# Tracing household transmission of SARS-CoV-2 in New Zealand using genomics

**DOI:** 10.1038/s44298-024-00032-6

**Published:** 2024-06-03

**Authors:** Lauren Jelley, Nayyereh Aminisani, Meaghan O’Neill, Tineke Jennings, Jordan Douglas, Srushti Utekar, Helen Johnston, David Welch, James Hadfield, SHIVERS Investigation Team, Nikki Turner, Tony Dowell, Annette Nesdale, Hazel C. Dobinson, Priscilla Campbell-Stokes, Michelle Balm, Cameron C. Grant, Karen Daniells, Peter McIntyre, Adrian Trenholme, Cass Byrnes, Ruth Seeds, Tim Wood, Megan Rensburg, Jort Cueto, Ernest Caballero, Joshma John, Emmanuel Penghulan, Danielle Currin, Mary Ryan, Andrea Bowers, Chor Ee Tan, Judy Bocacao, Wendy Gunn, Bryden Bird, Tegan Slater, Farjana Ahmed, Mai Anh Sam, Elaisa Glampe, Gabriella Davey, Joep de Ligt, David Winter, Nigel French, Paul G. Thomas, Richard J. Webby, Sue Huang, Jemma L. Geoghegan

**Affiliations:** 1 Institute of Environmental Science and Research, Wellington, New Zealand; 2 Department of Microbiology and Immunology, University of Otago, Dunedin, New Zealand; 3 Regional Public Health, Te Whatu Ora—Health New Zealand Capital, Coast and Hutt Valley, Wellington, New Zealand; 4 Centre for Computational Evolution, University of Auckland, Auckland, New Zealand; 5 Department of Physics, University of Auckland, Auckland, New Zealand; 6 School of Computer Science, University of Auckland, Auckland, New Zealand; 7 Fred Hutchinson Cancer Research Centre, Seattle, WA USA; 8 Tāwharau Ora/School of Veterinary Science, Massey University, Palmerston North, New Zealand; 9 Department of Host-Microbe Interactions, St Jude Children’s Research Hospital, Memphis, TN USA; 10 University of Auckland, Auckland, New Zealand; 11 University of Otago, Dunedin, New Zealand; 12 Te Whatu Ora—Health New Zealand Capital, Coast and Hutt Valley, Wellington, New Zealand; 13 Te Whatu Ora—Health New Zealand Counties Manukau, Auckland, New Zealand; 14 Te Whatu Ora—Health New Zealand Te Toka Tumai Auckland, Auckland, New Zealand

**Keywords:** Epidemiology, Virology

## Abstract

By early 2022, the highly transmissible Omicron variant of SARS-CoV-2 had spread across most of the world. For the first time since the pandemic began, New Zealand was experiencing high levels of community transmission of SARS-CoV-2. We enroled a cohort of households to better understand differences in transmission dynamics among subvariants of Omicron. We enroled 71 households, comprising 289 participants, and aimed to use viral genomes to gain a clearer understanding of variant-specific differences in epidemiological parameters affecting transmission dynamics. Approximately 80% of the households enroled experienced transmission of BA.2, while most of the remaining households had infections with BA.1 or BA.5. Using a logistic regression generalised linear mixed model, we found no difference in household secondary infection rate between Omicron subvariants BA.1, BA.2 and BA.5. Of the households recruited, the vast majority (92%) experienced a single chain of transmission with one inferred introduction. Further, we found that in 48% of the households studied, all household participants became infected following an index case. Most household participants tested positive within a week following an introduction, supporting the seven-day isolation requirement for household contacts that was in place in New Zealand at the time. By integrating genomic and epidemiological data, we show that viral transmission dynamics can be investigated with a higher level of granularity than with epidemiological data alone. Overall, households are a high risk setting for viral transmission in New Zealand.

## Introduction

During the first 18 months of the COVID-19 pandemic, New Zealand largely avoided community transmission of SARS-CoV-2^1^. New Zealand’s public health response used two main approaches for controlling community transmission of SARS-CoV-2. First, viral introductions into New Zealand were minimised via border restrictions, coupled with either managed isolation or quarantine for new arrivals dependent on their SARS-CoV-2 status upon entry^2^. Second, local transmission chains were rapidly extinguished by the use of national and regional lockdowns as well as the isolation of cases and quarantine of their contacts^3^. Due to these stringent measures, New Zealand, as of the 11 June 2021, had the lowest cumulative COVID-19 mortality rate in the OECD, which was ~5.2 deaths per million people^4^.

The arrival of Delta in August 2021^5^ however, showed that previously successful measures used to control community transmission of SARS-CoV-2 in New Zealand were much less effective against more transmissible variants^6^. Due to difficulties in controlling viral spread, coupled with high vaccination rates (by the end of 2021, approximately 92% of the eligible population had received a first dose of a COVID-19 vaccine^7,8^), New Zealand shifted from an elimination strategy to a mitigation approach. By early 2022, with the arrival of the highly transmissible Omicron variant^9^, coupled with less stringent public health restrictions^10^, New Zealand experienced high levels of community transmission of SARS-CoV-2 for the first time, with over 20,000 daily reported cases at the peak in early March 2022^11^. During the first quarter of 2022, Delta and Omicron (including both BA.1 and BA.2 subvariants) co-circulated in New Zealand, with Omicron rapidly surpassing Delta in frequency^9^. The sheer number of cases during the 2022 Omicron wave precluded the collection of detailed epidemiological information such as variant-specific patterns of infection. Close monitoring of the transmission dynamics of SARS-CoV-2 within households using both epidemiological data and viral genomics can help elucidate such patterns.

It has previously been suggested that households are a high risk setting for the transmission of SARS-CoV-2^12,13^, as they provide close contact and repeat exposure, amplifying the risk of viral transmission^14^. Therefore, households are an ideal setting to ascertain epidemiological and genomic parameters that affect rates of viral transmission^13,15–19^. These data have previously been used to infer secondary infection rates (SIR), which can be used to compare transmissibility of the different circulating subvariants. Until September 2022 (and during the time of this study), all household contacts of any index case in New Zealand were required to self-isolate for at least 7 days. Yet, how this quarantine requirement affected the risk of secondary infections within households is unknown. Further, we have little empirical data about temporal transmission dynamics within New Zealand households and whether the seven-day self-isolation requirement for household contacts was adequate.

In early 2022, we established a cohort study to examine the transmissibility of SARS-CoV-2 within New Zealand’s households. We aimed to use viral genomes to elucidate variant-specific differences in epidemiological parameters affecting transmission dynamics. We generated SARS-CoV-2 genomes from positive samples. Herein, we analysed a total of 71 households, made up of 289 participants, to investigate household transmission dynamics of SARS-CoV-2 in New Zealand.

## Results

**Fig. 1:**
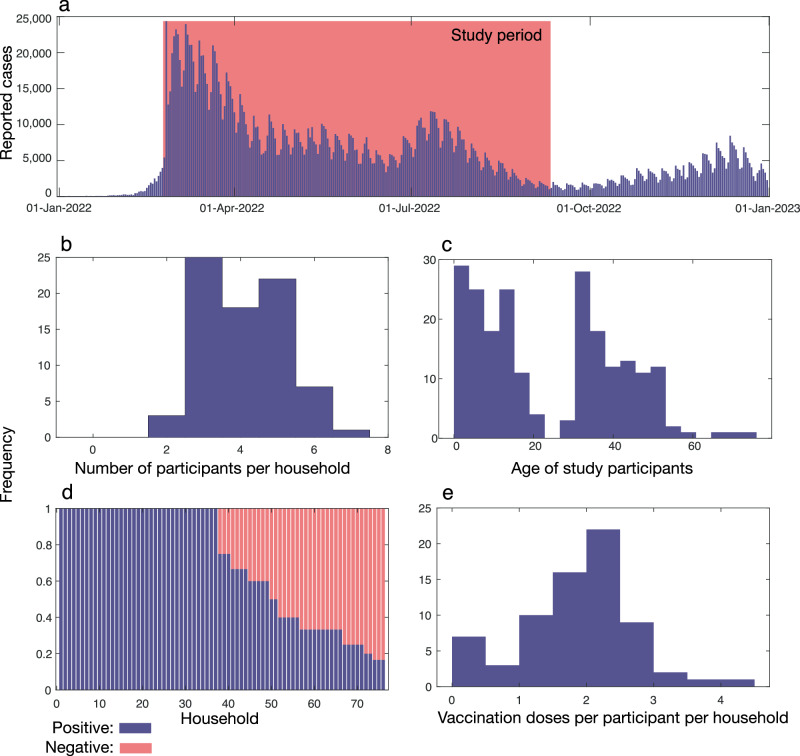
Timeline and demographics of the household transmission study and its participants. **a** Number of reported SARS-CoV-2 cases in the New Zealand community^43^ and the timing of this study (pink). **b** Number of participants per household. **c** Ages of participants. **d** Proportion of people per household who tested positive (purple) or negative (pink) for SARS-CoV-2. **e** Number of vaccinations per participant.

Between 7 February and 2 October 2022, 71 households, comprising 289 individuals, were enroled into the study (Fig. 1). Household size ranged from two to seven members, with a median of four (Fig. 1). The median age of all participants was 18 (range 0–76) and showed a bimodal distribution, reflecting both the requirement of households to have one member under 19 years old and the general makeup of nuclear families (Fig. 1c). The median age of participants in the first cluster was 8 (*n* = 147) while the median age in the second cluster was 39 (*n* = 142). Household participants included 166 females, 122 males and one identified as ‘other’. Most households (*n* = 57) followed a traditional nuclear family structure consisting of two parents and their children, while eight households were made up of single parent families as well as six multigenerational households.

During this study, 212 individual participants tested positive for SARS-CoV-2. Two participants tested positive for SARS-CoV-2 twice in two different transmission events in their household meaning that these were identified as reinfections. A total of 814 RT-PCR tests were positive from the 212 positive participants, including serial swabs from the same individuals. Of those who tested positive, the median age was 17. In *n* = 20 of the households, an index case was randomly assigned due to more than one participant testing positive on day zero and their viral genomes being indistinguishable. Household index cases were approximately evenly split among female (56%, *n* = 43) and male (44%, *n* = 34) participants, and among positive cases overall (58%, *n* = 124 female; 42%, *n* = 88 male). In 48% of households, all household participants tested positive for SARS-CoV-2 within the 28-day period.

**Fig. 2:**
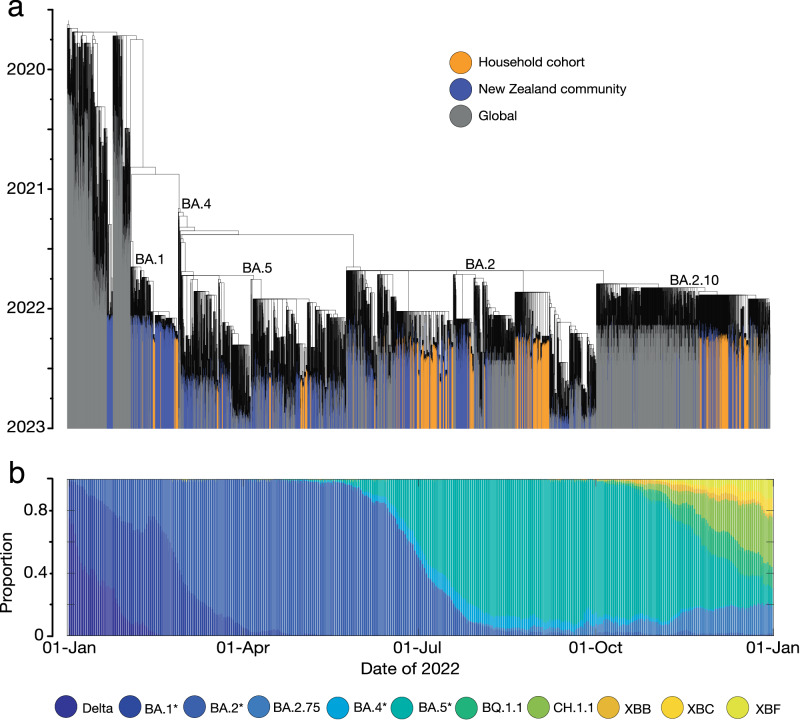
Phylogeny and genomic diversity of SARS-CoV-2 in New Zealand during 2022. **a** Maximum likelihood time-calibrated phylogenetic tree (black branches) showing the phylogenetic position of genomes generated within this household cohort study (orange bars), genomes from SARS-CoV-2 within the New Zealand community (purple bars) among a random selection of non-New Zealand SARS-CoV-2 genomes (grey bars). Major subvariants of Omicron are labelled. **b** Proportion of cases comprising different variants in New Zealand during 2022^43^.

From February to October 2022, several different Omicron subvariants circulated in New Zealand (Fig. 2) with the Delta wave diminishing around this time. From the 814 samples that tested positive for SARS-CoV-2 during this study, 603 viral genomes were successfully generated and their lineages were assigned using Pangolin^20^. The majority (82%) of genomes belonged to BA.2 (or progeny subvariants of BA.2*); the predominant subvariant in New Zealand during the study period, which circulated from March until early August 2022. Other subvariants present included BA.1*, BA.4*, BA.5* and recombinant XN (it was difficult to distinguish between sublineages below this parental level due to sequencing ambiguities). We found that viral genomes generated from this household cohort fell within New Zealand community lineages (Fig. 2).

Out of the 71 households recruited into this study, four households experienced two different transmission events, 28 or more days apart (Supplementary Fig. 1). In two of these households, the subvariant BA.2 circulated both times but infected different household members for each event. The other two households were due to different subvariants (BA.1 followed by BA.4.6, and BA.1 followed by BA.2). The households that experienced different subvariants of Omicron also included reinfections. Using genomic data, we found that two households had two different variants circulating within the household concurrently, meaning two separate sources of infection. In one five-member household, three individuals tested positive on the same day: two cases were identified as BA.2, while one was BA.5 (Supplementary Fig. 1). The BA.2 variant spread to the remaining two household members. The second household, also a five-member household, experienced both a BA.1 and BA.2 infection at the same time (Supplementary Fig. 1).

**Fig. 3:**
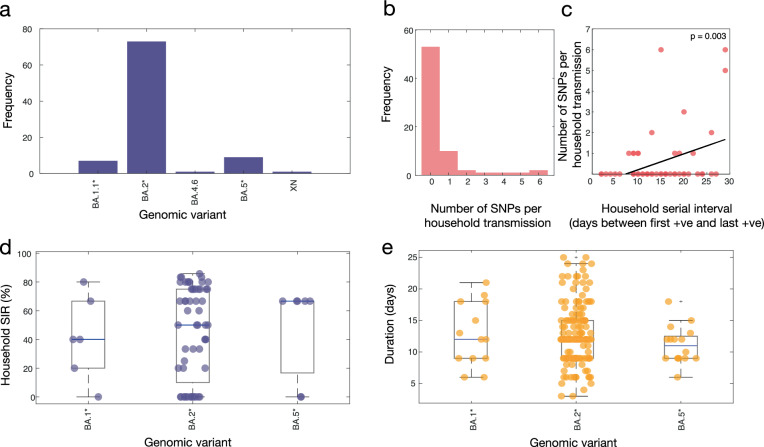
Omicron subvariants and genetic diversity within households. **a** Number of genomes by subvariants of Omicron. **b** Number of single nucleotide polymorphisms generated per household transmission. **c** Household serial interval (i.e. the number of days between the first positive and the last positive test in a household) versus the number of SNPs generated per household. **d** Estimated household secondary infection rate (SIR) per subvariant, including only those subvariants that infected more than one household. **e** Duration of testing positive by RT-PCR for each participant (days between first positive and first negative test) grouped by subvariant parental lineage, including only those subvariants with more than one household. Boxplots in (**d**) and (**e**) show the distribution of the lower and upper quartiles as well as the minimum and maximum values, where a blue horizontal line indicates the mean.

The majority (90%) of household transmission lineages generated little to no genetic diversity (i.e. 0−1 SNPs) within the consensus viral genomes inferred from each participant (Fig. 3). Household transmission events that generated the largest number of viral Single nucleotide polymorphism (SNPs) fell within the BA.2 subvariant. Viral genomes among three households accumulated 5–6 SNPs and a household infection duration of 15–28 days after the index case was identified. Despite this accumulation of SNPs, phylogenetic inference supports a single introduction, with the caveat of this large clonal outbreak in New Zealand at the time. The mean (and 95% confidence interval, CI) household SIR for subvariants BA.1, BA.2 and BA.5, was 55% (17–93%), 61% (50–71%), and 67% (38–95%), respectively (Fig. 3). The GLMMs provided no evidence of a difference in SIR between subvariants (*p* > 0.8 for all models including subvariant as a covariate). Households infected with BA.5 had an average of 0.65 doses (CI: 0.16–1.14) of the COVID-19 vaccine per household member, while BA.1 and BA.2 had an average of 1.8 (CI: 1.16–2.48) and 1.9 (CI: 1.71–2.01) doses per household member, respectively. We found no statistical association between household SIR and the number of vaccine doses per household member (Supplementary Table 2).

**Fig. 4:**
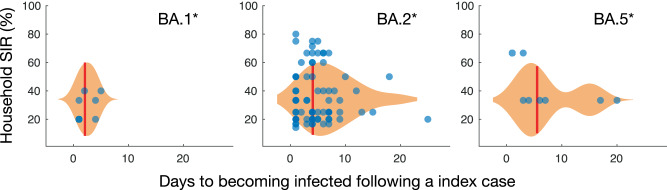
Number of days to becoming infected since the index case versus household secondary attack rate (SIR) for subvariants BA.1*, BA.2* and BA.5*. Violin plots show the density of points over the 28-day study period. The mean number of days for which participants tested positive following an index case is illustrated with a red line.

We compared the time (days) that household participants tested positive after an index case was identified, along with the household SIR between subvariants. Most household participants tested positive well within seven days after an index case with the mean number of days before becoming infected being 1.8, 3.3 and 5.7 days for BA.1, BA.2 and BA.5, respectively (Fig. 4). The 95% confidence interval for becoming infected following an index case was wider for BA.5 (2.6 - 8.8) compared to BA.1 (0.7–2.8) and BA.2 (2.6–4). Note that while households were only enroled for a maximum of 28 days, six households continued to have positive participants at the 28-day cut-off.

## Discussion

We recruited 71 households to better understand transmission dynamics of SARS-CoV-2. New Zealand’s first Omicron wave was dominated by BA.2, which meant that comparisons between subvariants within households were largely precluded as the number of households that experienced transmission with other Omicron subvariants was low in comparison. Nevertheless, while previous estimates of household SIR of Omicron variants were, on average, 38% (range 29–65%)^13,21–25^, we estimated an average household SIR to be as high as 71% for BA.5. Indeed, we found that in 75% of households infected with BA.5, all participants within the household became infected following an index case, excluding only one household due to the co-circulation of both BA.5 and BA.2. By comparison, the estimated average household SIR of both BA.1 and BA.2 were within the range described previously (55% and 61%, respectively), albeit at the higher end of this range and not significantly different from BA.5. It is worth noting that the relatively small sample sizes of BA.1 (*n* = 5 households) and BA.5 (*n* = 9 households), compared to BA.2 (*n* = 59 households) might explain these observed differences.

By the second quarter of 2022, Omicron subvariants with distinct transmissibility advantages over then-dominant BA.1 and BA.2 began to spread globally^26^. For example, BA.5, with the addition of two mutations, L452R and F486V, in the spike protein had a viral growth rate advantage over its predecessors and was found to dominate new introductions of SARS-CoV-2 into New Zealand^6,27^. It is noteworthy that even though New Zealand in general had high rates of vaccination against COVID-19, households infected with BA.5 had the lowest vaccine coverage with an average of 0.65 doses per household member. This is in contrast with an average of 1.8 and 1.9 doses per household member for BA.1 and BA.2, respectively. Overall, however, the relatively high household SIR among all Omicron subvariants estimated here suggests effective viral transmission even within a highly vaccinated cohort, validating the immune evasive properties of Omicron described previously^28–30^, particularly as this study took place prior the availability of bivalent vaccines in New Zealand.

With the exception of two households, our genomic data corroborated epidemiologically-identified household transmission clusters. Two households were infected with two different subvariants concurrently, allowing us to easily identify multiple sources of infection. On the one hand, it is likely that further households had more than one source of infection yet appeared to be genomically linked due to the largely clonal outbreak, especially of BA.2, in New Zealand at the time of the study. Yet, on the other hand, the seven-day self-isolation requirements for positive cases and their household contacts likely limited the number of separate introductions, meaning that most households (97%) experienced just a single source of infection. Indeed, the majority of viral genomes within households were identical within their respective consensus genomes. Among those household transmission clusters that generated considerable genetic diversity; the duration of their infections was long (between 15 to a maximum of 28 days following the identification of an index case).

Unlike many household transmission studies of SARS-CoV-2, we integrated both epidemiological data, such as assigning participants into clearly defined households, as well as genomic data, to accurately identify index cases and inform the overall direction of transmission within households. This study had several limitations. First, index cases were recruited only upon testing positive for SARS-CoV-2 and were asked to self-swab based on the number and severity of symptoms. This may cause the household SIR estimate to be lower since participants may have had an asymptomatic infection before the assumed index symptomatic case. Second, there is an inevitable time-lag between the reporting of a SARS-CoV-2 positive index case and enroling the remainder of the household in order to test them. This time-lag meant that many secondary cases tested positive on day three yet could have been infected earlier. These limitations could have been mitigated by enroling households before a SARS-CoV-2 infection occurred and by systematically testing all household participants for SARS-CoV-2 frequently, regardless of symptoms. By designing the study in this way, it would broaden the study population and therefore more accurately identify index cases, symptomatic and asymptomatic infections, as well as associated temporal dynamics. However, this study design would be logistically challenging. Finally, as mentioned previously, the dominance of BA.2 in New Zealand during the time of this study precluded robust comparisons among subvariants.

Overall, households in New Zealand, like in many countries^31–34^, are a high risk setting for SARS-CoV-2 transmission. We show that in New Zealand—where at the time of the study most population immunity was vaccine-derived—in half of all households studied, SARS-CoV-2 infected all household members and that reinfections within the study period were rare. Household transmission of SARS-CoV-2 typically occurred following just a single introduction and most secondary cases tested positive within just a few days of an index case, justifying the seven-day isolation requirement for household contacts that was in place at the time. We also found that the average household SIR in New Zealand was relatively high compared with other global estimates. While the requirement of household contacts to self-isolate following an index case might have increased the household SIR, direct comparisons with populations without such a requirement are difficult to make.

## Methods

### Ethics statement

All participants provided written consent allowing samples to be collected for research purposes. This study was approved by the New Zealand Health and Disability Ethics Committee (NTX/11/11/102).

### Study design

From 2021, the already established Southern Hemisphere Influenza Vaccine Effectiveness Research and Surveillance (SHIVERS) platform recruited households to be part of a household cohort to research influenza and SARS-CoV-2 transmission within households. This cohort, known as WellKiwi Household cohort, was the fourth iteration of the SHIVERS study (SHIVERS IV). For a household to be eligible to enrol, at least one household member was required to be under the age of 19, the household had to enrol for a minimum of two years and all members of the household had to agree to participate. Beginning in February 2022, participants in the Wellkiwi Household cohort received a weekly symptom survey in which they would report whether any of the household members were experiencing respiratory disease symptoms. Responses were triaged by the WellKiwi clinical team and participants would collect a nasal swab via self-swabbing. Swabs were couriered to the WHO National Influenza Centre at the Institute of Environmental Science and Research (ESR) and tested by the Clinical Virology Department for SARS-CoV-2 by RT-PCR. Recruitment into the SARS-CoV-2 household transmission study was based on a positive SARS-CoV-2 RT-PCR result. The household members of SARS-CoV-2 positive participants were then invited to participate in this sub-cohort study. Recruited participants completed a daily symptom survey and collected a nasal swab via self-swabbing every 3 days. Swabs were then couriered to the laboratory for testing. RT-PCR results were returned within a 24–48-h timeframe. The follow-up period for household members was defined by either the continuation of swabbing every three days until the participant had returned negative SARS-CoV-2 RT-PCR results twice in succession, or the household had participated for a total of 28 days. A transmission event was classified as the circulation of SARS-Co-2 within a household within a 28-day period. If transmission occurred more than 28 days after the last positive case within the household it was classified as a separate transmission event or reinfection.

### Viral RNA extraction and PCR

Viral RNA was extracted using the ZiXpress or Kingfisher Flex automated analysers, using the Thermofisher Scientific™ MagMax Viral/Pathogen Nucleic Acid Isolation Kit (A48310) and used as per manufacturer’s instructions. Nasopharyngeal samples collected from participants were tested for SARS-CoV-2 using the CDC Influenza SARS-CoV-2 (Flu SC2) Multiplex Assay and the Quantabio qScript XLT 1-step RT-qPCR ToughMix (95132-500), and confirmed using the CCDC primers for N gene and ORF1ab, and the Quantabio qScript XLT 1-step RT-qPCR ToughMix^TM^ (95132-500).

### Virus genomic sequencing

All household samples that tested positive for SARS-CoV-2 by rRT-PCR were then referred to the genomic sequencing department at the Institute of ESR for genome sequencing. In brief, sequencing was performed following the Midnight protocol v6^35^. This protocol contains a 1200-bp primer set for tiling the SARS-CoV-2 genome, as well as using the Oxford Nanopore Technologies R9.4 chemistry. Using a standardised pipeline (https://github.com/ESR-NZ/NZ_SARS-CoV-2_genomics), which is based on well-established bioinformatics pipelines (https://artic.network/ncov-2019/ncov2019-bioinformatics-sop.html; v1.2.1) consensus viral genomes were generated. These genomes were subject to quality testing and only genomes with fewer than 50% ambiguities were selected for further analysis.

### Genome sequence analysis

High-quality genomes were first designated lineages using Pangolin v4.0.6^20^. Sublineages were grouped by parent lineage due to sequence ambiguities in some genomes. Household cohort viral genomes were first aligned together with SARS-CoV-2 genomes sampled during 2022 from New Zealand (*n* = 2029) and the rest of the world (*n* = 1967) selected at random from GISAID^36^, as well as 408 global genomes sampled during 2020–2021 (see Supplementary Table 1 for all genome accession numbers). Genomes were aligned using NextAlign^37^, using Wuhan-Hu-1 (NC_045512.2) as a reference. A maximum likelihood time-calibrated phylogenetic tree was estimated using IQ-TREE2^38^, using the Hasegawa-Kishino-Yano (HKY + Γ) nucleotide substitution model^39^ (the best-fit model was determined by ModelFinder^40^), and branch support assessment using the ultrafast bootstrap method^41^.

SNP analysis for households was performed by first aligning genomes for a given household using NextAlign and summing the number of nucleotide differences. Households that showed two separate transmission events, or two different subvariants that circulated concurrently were aligned separately.

### Data analysis

An index case was defined as the participant from the Wellkiwi Household cohort who was the first to test positive for SARS-CoV-2 by RT-PCR upon which the household was then recruited into the household transmission study. In instances where more than one household member tested positive for the same subvariant on the same day (i.e. day zero), and the virus genome was genetically identical or an index could not be inferred from the phylogeny, index cases were randomly assigned. The household SIR was defined as the percentage of household members that tested positive following a positive test from the index case within 28 days and cases were linked to the same transmission chain using genomics (methods below). Statistical tests to compare household SIR among subvariants were performed using logistic regression generalised linear mixed models (GLMMs). It was assumed that secondary cases in households in which the same Omicron subvariant circulated and genomes were phylogenetically linked were from a single introduction following the index case. Households that had two different subvariants circulating concurrently, and those that had transmission events at vastly different times, were regarded as separate transmission events. For the GLMMs, household was included as a random effect, and the following variables were considered as potential confounders: number of household members; vaccination status; and age. Analyses were performed using the glmer command in the R package lme4^42^.

## Supplementary information

Supplementary Information

## Data Availability

SARS-CoV-2 genomes generated in this study are available online via GISAID under accession numbers: EPI_ISL_18565210-18565820.

## Code availability

The software used in the analysis for this manuscript: PANGO v4.0.6, NextAlign v.2.3.0 and IQTREE2. As well as the ARTIC bioinformatics pipeline available from (https://artic.network/ncov-2019/ncov2019-bioinformatics-sop.html; v1.2.1).
