# Supplementary material for: Tracing household transmission of SARS-CoV-2 in New Zealand using genomics
Source: Npj Viruses. 2024 Jun 3;2:21. doi: 10.1038/s44298-024-00032-6 (PMC11721343; doi:10.1038/s44298-024-00032-6)
Supplement: Supplementary file 1 — Supplementary Information [file 44298_2024_32_MOESM1_ESM.pdf]

Supplementary Table 1. Accessions for global SARS-CoV-2 genomes used in the phylogenomic analysis.

hCoV-19/Albania/244432/2021|EPI_ISL_16854868|2021-11-23
hCoV-19/Albania/249662/2021|EPI_ISL_16855263|2021-12-13
hCoV-19/Anhui/241/2020|EPI_ISL_16138319|2020-02-06
hCoV-19/Anhui/445/2020|EPI_ISL_1081310|2020-02-08
hCoV-19/Anhui/728/2020|EPI_ISL_16138296|2020-02-22
hCoV-19/Australia/NSW-ICPMR-30808/2022|EPI_ISL_14285457|2022-07-24
hCoV-19/Australia/NSW-ICPMR-31949/2022|EPI_ISL_14536640|2022-08-08
hCoV-19/Australia/NSW-ICPMR-31966/2022|EPI_ISL_14536657|2022-08-09
hCoV-19/Australia/NSW-ICPMR-32262/2022|EPI_ISL_14617315|2022-08-15
hCoV-19/Australia/NSW-ICPMR-32357/2022|EPI_ISL_14617410|2022-08-19
hCoV-19/Australia/NSW-ICPMR-32559/2022|EPI_ISL_14789031|2022-08-17
hCoV-19/Australia/NSW-ICPMR-32803/2022|EPI_ISL_14789275|2022-08-14
hCoV-19/Australia/NSW-ICPMR-32812/2022|EPI_ISL_14789284|2022-08-15
hCoV-19/Australia/NSW-ICPMR-33098/2022|EPI_ISL_14913138|2022-08-29
hCoV-19/Australia/NSW-ICPMR-33263/2022|EPI_ISL_14993314|2022-08-27
hCoV-19/Australia/NSW-ICPMR-33981/2022|EPI_ISL_15250271|2022-09-19
hCoV-19/Australia/NSW-ICPMR-34038/2022|EPI_ISL_15250308|2022-09-24
hCoV-19/Australia/NSW-ICPMR-34305/2022|EPI_ISL_15362258|2022-09-29
hCoV-19/Australia/NSW-ICPMR-36047/2022|EPI_ISL_15736413|2022-10-27
hCoV-19/Australia/NSW-ICPMR-37591/2022|EPI_ISL_15982389|2022-11-18
hCoV-19/Australia/NSW-ICPMR-37698/2022|EPI_ISL_15982496|2022-11-19
hCoV-19/Australia/NSW-ICPMR-37700/2022|EPI_ISL_15982498|2022-11-20
hCoV-19/Australia/NSW-ICPMR-37704/2022|EPI_ISL_15982502|2022-11-23
hCoV-19/Australia/NSW-ICPMR-37708/2022|EPI_ISL_15982506|2022-11-16
hCoV-19/Australia/NSW-ICPMR-37716/2022|EPI_ISL_15982514|2022-11-25
hCoV-19/Australia/NSW-ICPMR-37907/2022|EPI_ISL_16058831|2022-11-26
hCoV-19/Australia/NSW-ICPMR-37915/2022|EPI_ISL_16058839|2022-11-16
hCoV-19/Australia/NSW-ICPMR-37922/2022|EPI_ISL_16058846|2022-11-16
hCoV-19/Australia/NSW-ICPMR-38151/2022|EPI_ISL_16059075|2022-11-23
hCoV-19/Australia/NSW-ICPMR-38159/2022|EPI_ISL_16059083|2022-11-29
hCoV-19/Australia/NSW-ICPMR-38165/2022|EPI_ISL_16059089|2022-11-23
hCoV-19/Australia/NSW-ICPMR-38167/2022|EPI_ISL_16059091|2022-11-23
hCoV-19/Australia/NSW-ICPMR-38459/2022|EPI_ISL_16059383|2022-11-23
hCoV-19/Australia/NSW-ICPMR-38461/2022|EPI_ISL_16059385|2022-11-23
hCoV-19/Australia/NSW-ICPMR-38466/2022|EPI_ISL_16059390|2022-11-23
hCoV-19/Australia/NSW-ICPMR-38473/2022|EPI_ISL_16059397|2022-11-23
hCoV-19/Australia/NSW-ICPMR-38678/2022|EPI_ISL_16059602|2022-11-30
hCoV-19/Australia/NSW-ICPMR-38680/2022|EPI_ISL_16059604|2022-11-30
hCoV-19/Australia/NSW-ICPMR-38742/2022|EPI_ISL_16128448|2022-11-24
hCoV-19/Australia/NSW-ICPMR-38744/2022|EPI_ISL_16128449|2022-11-30

hCoV-19/Australia/NSW-ICPMR-38750/2022|EPI_ISL_16128452|2022-11-30
hCoV-19/Australia/NSW-ICPMR-38756/2022|EPI_ISL_16128454|2022-11-30
hCoV-19/Australia/NSW-ICPMR-38768/2022|EPI_ISL_16128462|2022-11-30
hCoV-19/Australia/NSW-ICPMR-38789/2022|EPI_ISL_16128465|2022-11-30
hCoV-19/Australia/NSW-ICPMR-38829/2022|EPI_ISL_16128480|2022-11-30
hCoV-19/Australia/NSW-ICPMR-39027/2022|EPI_ISL_16128530|2022-11-30
hCoV-19/Australia/NSW-SAVID-11632/2022|EPI_ISL_12666430|2022-04-13
hCoV-19/Australia/NSW-SAVID-12306/2022|EPI_ISL_13059297|2022-05-26
hCoV-19/Australia/NSW-SAVID-12367/2022|EPI_ISL_13199506|2022-05-29
hCoV-19/Australia/NSW-SAVID-12533/2022|EPI_ISL_13530585|2022-06-10
hCoV-19/Australia/NSW-SAVID-12551/2022|EPI_ISL_13530547|2022-06-10
hCoV-19/Australia/NSW-SAVID-14932/2022|EPI_ISL_15980446|2022-11-15
hCoV-19/Australia/NSW-SAVID-14951/2022|EPI_ISL_15980457|2022-11-15
hCoV-19/Australia/NSW-SAVID-14955/2022|EPI_ISL_15980460|2022-11-15
hCoV-19/Australia/NSW-SAVID-14965/2022|EPI_ISL_15980468|2022-11-16
hCoV-19/Australia/NSW-SAVID-14969/2022|EPI_ISL_15980472|2022-11-14
hCoV-19/Australia/NSW-SAVID-15042/2022|EPI_ISL_16051039|2022-11-15
hCoV-19/Australia/NSW680/2020|EPI_ISL_513323|2020-07-16
hCoV-19/Australia/QLD0x00C6AD/2022|EPI_ISL_10305533|2022-02-01
hCoV-19/Australia/QLD0x00CF43/2022|EPI_ISL_10973453|2022-02-16
hCoV-19/Australia/QLD0x00D7C0/2022|EPI_ISL_11632753|2022-03-02
hCoV-19/Australia/QLD0x00E071/2022|EPI_ISL_12135276|2022-04-12
hCoV-19/Australia/QLD0x00E2E8/2022|EPI_ISL_12386647|2022-04-12
hCoV-19/Australia/QLD0x00E315/2022|EPI_ISL_12386627|2022-04-13
hCoV-19/Australia/QLD0x00E740/2022|EPI_ISL_12721795|2022-05-03
hCoV-19/Australia/QLD0x00E8EC/2022|EPI_ISL_12807331|2022-05-03
hCoV-19/Australia/QLD0x00F435/2022|EPI_ISL_13417947|2022-06-11
hCoV-19/Australia/QLD0x010999/2022|EPI_ISL_14532977|2022-07-23
hCoV-19/Australia/QLD0x01099F/2022|EPI_ISL_14532992|2022-07-26
hCoV-19/Australia/QLD0x011261/2022|EPI_ISL_14916323|2022-09-02
hCoV-19/Australia/QLD0x012821/2022|EPI_ISL_16016493|2022-11-23
hCoV-19/Australia/QLD0x012822/2022|EPI_ISL_16016494|2022-11-26
hCoV-19/Australia/QLD0x012825/2022|EPI_ISL_16016498|2022-11-10
hCoV-19/Australia/QLD0x012826/2022|EPI_ISL_16016500|2022-11-09
hCoV-19/Australia/QLD0x012828/2022|EPI_ISL_16016504|2022-11-26
hCoV-19/Australia/QLD0x012889/2022|EPI_ISL_16016491|2022-11-25
hCoV-19/Australia/SA0091/2020|EPI_ISL_451126|2020-04-01
hCoV-19/Australia/VIC10540/2020|EPI_ISL_562173|2020-09-03
hCoV-19/Australia/VIC19500/2020|EPI_ISL_5299641|2020-08-05
hCoV-19/Australia/VIC328/2020|EPI_ISL_12846177|2020-03-17
hCoV-19/Australia/VIC4033/2020|EPI_ISL_519418|2020-07-11
hCoV-19/Australia/VIC67778/2022|EPI_ISL_14547843|2022-07-31
hCoV-19/Australia/VIC69258/2022|EPI_ISL_15118637|2022-08-21

hCoV-19/Australia/VIC69450/2022|EPI_ISL_15118806|2022-08-22
hCoV-19/Australia/WA1765/2022|EPI_ISL_10922810|2022-03-01
hCoV-19/Australia/WA1775/2022|EPI_ISL_10922820|2022-03-01
hCoV-19/Austria/AGES-150955/2022|EPI_ISL_14516173|2022-04-12
hCoV-19/Austria/AGES-161099/2022|EPI_ISL_14513928|2022-06-08
hCoV-19/Austria/AGES-172861/2022|EPI_ISL_14515525|2022-06-08
hCoV-19/Austria/AGES-172862/2022|EPI_ISL_14515526|2022-06-08
hCoV-19/Austria/AGES-172888/2022|EPI_ISL_14515547|2022-06-08
hCoV-19/Austria/AGES-177339/2022|EPI_ISL_14513784|2022-06-11
hCoV-19/Austria/LB-R00059-S116/2022|EPI_ISL_9852283|2022-02-03
hCoV-19/Austria/LB-R00059-S130/2022|EPI_ISL_9852297|2022-02-01
hCoV-19/Austria/LB-R00059-S159/2022|EPI_ISL_9852325|2022-02-03
hCoV-19/Austria/LB-R00059-S176/2022|EPI_ISL_9852342|2022-02-03
hCoV-19/Austria/LB-R00062-S017/2022|EPI_ISL_10580919|2022-02-16
hCoV-19/Austria/LB-R00062-S161/2022|EPI_ISL_10580958|2022-02-17
hCoV-19/Austria/LB-R00064-S101/2022|EPI_ISL_10896919|2022-03-01
hCoV-19/Austria/LB-R00064-S106/2022|EPI_ISL_10896924|2022-03-01
hCoV-19/Austria/LB-R00064-S109/2022|EPI_ISL_10896927|2022-03-01
hCoV-19/Austria/LB-R00064-S206/2022|EPI_ISL_10897021|2022-03-02
hCoV-19/Austria/LB-R00064-S230/2022|EPI_ISL_10897042|2022-03-02
hCoV-19/Austria/LB-R00066-S321/2022|EPI_ISL_11325699|2022-03-16
hCoV-19/Austria/LB-R00066-S348/2022|EPI_ISL_11325724|2022-03-16
hCoV-19/Austria/LB-R00066-S361/2022|EPI_ISL_11325737|2022-03-16
hCoV-19/Austria/LB-R00091-S066/2022|EPI_ISL_14063312|2022-07-20
hCoV-19/Austria/LB-R00092-S193/2022|EPI_ISL_14217517|2022-07-27
hCoV-19/Austria/LB-R00092-S211/2022|EPI_ISL_14217535|2022-07-27
hCoV-19/Austria/LB-R00096-S317/2022|EPI_ISL_14750112|2022-08-22
hCoV-19/Austria/LB-R00104-S251/2022|EPI_ISL_15259696|2022-09-27
hCoV-19/Austria/LB-R00108-S215/2022|EPI_ISL_15602742|2022-10-25
hCoV-19/Austria/LB-R00108-S297/2022|EPI_ISL_15602824|2022-10-25
hCoV-19/Austria/LB-R00108-S298/2022|EPI_ISL_15602825|2022-10-25
hCoV-19/Bahrain/341478738/2021|EPI_ISL_14132993|2021-02-08
hCoV-19/Bangladesh/ECHD_08985/2020|EPI_ISL_11811710|2020-08-03
hCoV-19/Bangladesh/ideSHi-Redwan-230-R/2020|EPI_ISL_16180632|2020-12-31
hCoV-19/Beijing/414/2022|EPI_ISL_13858976|2022-03-02
hCoV-19/Belgium/12215737601/2022|EPI_ISL_15385193|2022-09-26
hCoV-19/Belgium/AZKLINA122-050636/2022|EPI_ISL_10679388|2022-02-16
hCoV-19/Belgium/AZKLINA122-114320/2022|EPI_ISL_13214882|2022-05-25
hCoV-19/Belgium/AZKLINA122-142383/2022|EPI_ISL_14608063|2022-07-25
hCoV-19/Belgium/CHUNamur14087122/2022|EPI_ISL_11565142|2022-03-17
hCoV-19/Belgium/CHUNamur14124159/2022|EPI_ISL_12323600|2022-04-13
hCoV-19/Belgium/CHUNamur14149312/2022|EPI_ISL_12648037|2022-05-03
hCoV-19/Belgium/CHUNamur14195183/2022|EPI_ISL_13391878|2022-06-09

hCoV-19/Belgium/CHUNamur14197925/2022|EPI_ISL_13444983|2022-06-11

hCoV-19/Belgium/IPG-1673/2022|EPI_ISL_13421898|2022-06-11

hCoV-19/Belgium/Jessa_11-2223-004054ZOL/2022|EPI_ISL_13284649|2022-06-09

hCoV-19/Belgium/Jessa_11-2242-000831LKO/2022|EPI_ISL_15433747|2022-10-17

hCoV-19/Belgium/Jessa_55-2253-000421/2022|EPI_ISL_8455623|2022-01-02

hCoV-19/Belgium/rega-32509/2022|EPI_ISL_14621434|2022-02-16

hCoV-19/Belgium/rega-32639/2022|EPI_ISL_14621568|2022-02-16

hCoV-19/Belgium/rega-35838/2022|EPI_ISL_14630735|2022-03-17

hCoV-19/Belgium/rega-42602/2022|EPI_ISL_13389595|2022-05-29

hCoV-19/Belgium/rega-44576/2022|EPI_ISL_14728330|2022-07-22

hCoV-19/Belgium/rega-44715/2022|EPI_ISL_14749124|2022-07-27

hCoV-19/Belgium/rega-44990/2022|EPI_ISL_14732982|2022-08-05

hCoV-19/Belgium/rega-45831/2022|EPI_ISL_15425466|2022-09-30

hCoV-19/Belgium/Sciensano-LS-S1634/2020|EPI_ISL_13413323|2020-03-17

hCoV-19/Belgium/UGent-6127/2022|EPI_ISL_15158262|2022-08-30

hCoV-19/Belgium/ULB-IBC_CV8440465407/2022|EPI_ISL_10844711|2022-03-02

hCoV-19/Belgium/ULG-10364/2020|EPI_ISL_540531|2020-08-06

hCoV-19/Belgium/ULG-25830/2022|EPI_ISL_12219685|2022-04-12

hCoV-19/Belgium/ULG-26133/2022|EPI_ISL_12635764|2022-05-01

hCoV-19/Belgium/ULG-26574/2022|EPI_ISL_13216459|2022-05-27

hCoV-19/Belgium/ULG-26575/2022|EPI_ISL_13216460|2022-05-28

hCoV-19/Belgium/ULG-26627/2022|EPI_ISL_13251710|2022-05-27

hCoV-19/Belgium/ULG-27738/2022|EPI_ISL_14217281|2022-07-28

hCoV-19/Belgium/ULG-27985/2022|EPI_ISL_14436987|2022-08-08

hCoV-19/Belgium/ZNA_25387999/2022|EPI_ISL_12636900|2022-05-03

hCoV-19/Brazil/AM-8587/2021|EPI_ISL_16981400|2021-04-18

hCoV-19/Brazil/AM-L10_GATES-CD27228/2021|EPI_ISL_17298366|2021-08-04

hCoV-19/Brazil/BA-FIOCRUZ-PVM30175/2020|EPI_ISL_13407623|2020-10-21

hCoV-19/Brazil/BA-LACEN-119/2020|EPI_ISL_1583647|2020-06-17

hCoV-19/Brazil/BA-LACEN-BA1405-292302156/2022|EPI_ISL_11895217|2022-03-02

hCoV-19/Brazil/BA-VC4/2020|EPI_ISL_13838073|2020-10-20

hCoV-19/Brazil/CE-3961/2021|EPI_ISL_16981142|2021-01-18

hCoV-19/Brazil/CE-FIOCRUZ-00377/2021|EPI_ISL_3102518|2021-05-13

hCoV-19/Brazil/CE-IMTSP-CD8077/2021|EPI_ISL_14800269|2021-03-27

hCoV-19/Brazil/ES-LACENES-320648505/2021|EPI_ISL_15604651|2021-07-01

hCoV-19/Brazil/ES-LACENES-320866821/2021|EPI_ISL_15396197|2021-10-07

hCoV-19/Brazil/MG-FIOCRUZ-2746/2022|EPI_ISL_15257796|2022-08-05

hCoV-19/Brazil/MG-FIOCRUZ-3297/2022|EPI_ISL_15818252|2022-07-17

hCoV-19/Brazil/MG-FIOCRUZ-3483/2022|EPI_ISL_16050732|2022-11-18

hCoV-19/Brazil/MG-FIOCRUZ-3486/2022|EPI_ISL_16050735|2022-11-18

hCoV-19/Brazil/MG-FIOCRUZ-3490/2022|EPI_ISL_16050739|2022-11-18

hCoV-19/Brazil/MG-FIOCRUZ-3493/2022|EPI_ISL_16050742|2022-11-18

hCoV-19/Brazil/MG-FIOCRUZ-3510/2022|EPI_ISL_16050758|2022-11-21

hCoV-19/Brazil/PA-IEC-176944/2020|EPI_ISL_16893926|2020-12-14
hCoV-19/Brazil/PB-LaBiMol-040112M/2021|EPI_ISL_16898108|2021-12-01
hCoV-19/Brazil/PB-LaBiMol-043011L/2021|EPI_ISL_16897999|2021-11-29
hCoV-19/Brazil/PE-IMTSP-CD24995/2021|EPI_ISL_14801642|2021-10-27
hCoV-19/Brazil/PR-FIOCRUZ-34281/2020|EPI_ISL_8005604|2020-06-16
hCoV-19/Brazil/PR-IMTSP-CD10778/2021|EPI_ISL_16960581|2021-06-03
hCoV-19/Brazil/PR-IPEC_VIGCV19_FOZ_1371/2021|EPI_ISL_12426197|2021-06-21
hCoV-19/Brazil/PR-TOBR00086/2021|EPI_ISL_16030906|2021-11-03
hCoV-19/Brazil/RJ-COVID-19_UFRJ-48668/2021|EPI_ISL_16084113|2021-07-21
hCoV-19/Brazil/RJ-COVID-19_UFRJ-53495/2021|EPI_ISL_16084251|2021-09-01
hCoV-19/Brazil/RJ-COVID-19_UFRJ-63876/2022|EPI_ISL_16083665|2022-02-01
hCoV-19/Brazil/RJ-COVID-19_UFRJ-68392/2022|EPI_ISL_16083768|2022-07-06
hCoV-19/Brazil/RJ-COVID-19_UFRJ-69324/2022|EPI_ISL_16083790|2022-06-14
hCoV-19/Brazil/RJ-COVID-19_UFRJ-69596/2022|EPI_ISL_16083835|2022-06-21
hCoV-19/Brazil/RJ-LNN10228/2022|EPI_ISL_16007692|2022-10-27
hCoV-19/Brazil/RJ-LNN10231/2022|EPI_ISL_16007693|2022-10-28
hCoV-19/Brazil/RJ-LNN10317/2022|EPI_ISL_16007931|2022-11-07
hCoV-19/Brazil/RJ-Marica-15508P/2022|EPI_ISL_16084312|2022-04-30
hCoV-19/Brazil/RJ-Marica-15659W/2022|EPI_ISL_16084318|2022-05-10
hCoV-19/Brazil/RN_IMT_6.08/2020|EPI_ISL_12143787|2020-05-07
hCoV-19/Brazil/RS-UFCSPA-46476/2021|EPI_ISL_17475694|2021-09-07
hCoV-19/Brazil/SP-FMRP-1008/2021|EPI_ISL_16225501|2021-02-11
hCoV-19/Brazil/SP-FMRP-1507/2021|EPI_ISL_16354538|2021-06-18
hCoV-19/Brazil/SP-FMRP-1581/2021|EPI_ISL_16354611|2021-07-19
hCoV-19/Brazil/SP-FMRP-2082/2020|EPI_ISL_16355101|2020-06-22
hCoV-19/Brazil/SP-FMRP-433/2020|EPI_ISL_16224948|2020-08-12
hCoV-19/Brazil/SP-HIAE-ID2677/2021|EPI_ISL_17473991|2021-03-20
hCoV-19/Brazil/SP-IB_129769/2021|EPI_ISL_16371657|2021-08-30
hCoV-19/Brunei/7722010302/2022|EPI_ISL_14811753|2022-08-14
hCoV-19/Bulgaria/22BG-EU_017744_PI167/2022|EPI_ISL_13179324|2022-02-02
hCoV-19/Bulgaria/22BG-EU_018735_PI176/2022|EPI_ISL_13180166|2022-02-16
hCoV-19/Bulgaria/22BG-EU_019660_PI185/2022|EPI_ISL_13138659|2022-03-02
hCoV-19/Cambodia/939553/2022|EPI_ISL_14780363|2022-08-26
hCoV-19/Cambodia/g0817014/2022|EPI_ISL_14780395|2022-08-08
hCoV-19/Cambodia/g1029046/2022|EPI_ISL_16000880|2022-10-29
hCoV-19/Cambodia/g1110085/2022|EPI_ISL_16000881|2022-11-10
hCoV-19/Cambodia/g1114097/2022|EPI_ISL_16000883|2022-11-14
hCoV-19/Cambodia/g1115112/2022|EPI_ISL_16000884|2022-11-16
hCoV-19/Cambodia/g1116148/2022|EPI_ISL_16000886|2022-11-17
hCoV-19/Cambodia/g1124030/2022|EPI_ISL_16000888|2022-11-24
hCoV-19/Cambodia/VIR22-003363/2022|EPI_ISL_9696489|2022-02-02
hCoV-19/Cambodia/VIRU22-004156/2022|EPI_ISL_10068000|2022-02-05
hCoV-19/Cameroon/CV439/2020|EPI_ISL_845561|2020-11-19

hCoV-19/Canada/AB-ABPHL-18481/2021|EPI_ISL_2941469|2021-04-01
hCoV-19/Canada/AB-ABPHL-67080/2022|EPI_ISL_13103623|2022-05-01
hCoV-19/Canada/AB-ABPHL-68957/2022|EPI_ISL_13349353|2022-05-25
hCoV-19/Canada/AB-ABPHL-68983/2022|EPI_ISL_13349377|2022-05-28
hCoV-19/Canada/AB-ABPHL-70701/2022|EPI_ISL_13724833|2022-05-28
hCoV-19/Canada/AB-ABPHL-71136/2022|EPI_ISL_13725173|2022-06-12
hCoV-19/Canada/AB-ABPHL-71441/2022|EPI_ISL_13725429|2022-06-09
hCoV-19/Canada/AB-ABPHL-79535/2022|EPI_ISL_15278808|2022-08-30
hCoV-19/Canada/AB-ABPHL-85577/2022|EPI_ISL_15977904|2022-11-09
hCoV-19/Canada/AB-ABPHL-86007/2022|EPI_ISL_15978250|2022-11-12
hCoV-19/Canada/AB-ABPHL-86025/2022|EPI_ISL_15978265|2022-11-12
hCoV-19/Canada/BC-BCCDC-395186/2022|EPI_ISL_12381990|2022-03-16
hCoV-19/Canada/BC-BCCDC-406462/2020|EPI_ISL_12377866|2020-10-30
hCoV-19/Canada/BC-BCCDC-416927/2022|EPI_ISL_12662256|2022-04-13
hCoV-19/Canada/BC-BCCDC-418576/2022|EPI_ISL_12662520|2022-04-12
hCoV-19/Canada/BC-BCCDC-418697/2022|EPI_ISL_12662644|2022-04-12
hCoV-19/Canada/BC-BCCDC-418701/2022|EPI_ISL_12662648|2022-04-12
hCoV-19/Canada/BC-BCCDC-418791/2022|EPI_ISL_12662729|2022-04-13
hCoV-19/Canada/BC-BCCDC-418946/2022|EPI_ISL_12662855|2022-04-13
hCoV-19/Canada/BC-BCCDC-432235/2022|EPI_ISL_12968060|2022-05-02
hCoV-19/Canada/BC-BCCDC-432307/2022|EPI_ISL_12968126|2022-05-01
hCoV-19/Canada/BC-BCCDC-432398/2022|EPI_ISL_12968209|2022-05-02
hCoV-19/Canada/BC-BCCDC-432403/2022|EPI_ISL_12968213|2022-05-02
hCoV-19/Canada/BC-BCCDC-432406/2022|EPI_ISL_12968216|2022-05-01
hCoV-19/Canada/BC-BCCDC-432422/2022|EPI_ISL_12968231|2022-05-01
hCoV-19/Canada/BC-BCCDC-432477/2022|EPI_ISL_12968272|2022-05-02
hCoV-19/Canada/BC-BCCDC-432645/2022|EPI_ISL_12968419|2022-05-02
hCoV-19/Canada/BC-BCCDC-435200/2022|EPI_ISL_13317412|2022-05-03
hCoV-19/Canada/BC-BCCDC-435340/2022|EPI_ISL_13317516|2022-05-03
hCoV-19/Canada/BC-BCCDC-449954/2022|EPI_ISL_13411892|2022-05-25
hCoV-19/Canada/BC-BCCDC-450143/2022|EPI_ISL_13412010|2022-05-25
hCoV-19/Canada/BC-BCCDC-450193/2022|EPI_ISL_13412057|2022-05-27
hCoV-19/Canada/BC-BCCDC-450222/2022|EPI_ISL_13412079|2022-05-29
hCoV-19/Canada/BC-BCCDC-450274/2022|EPI_ISL_13412121|2022-05-26
hCoV-19/Canada/BC-BCCDC-450391/2022|EPI_ISL_13412200|2022-05-26
hCoV-19/Canada/BC-BCCDC-450481/2022|EPI_ISL_13412283|2022-05-27
hCoV-19/Canada/BC-BCCDC-450499/2022|EPI_ISL_13412297|2022-05-29
hCoV-19/Canada/BC-BCCDC-450506/2022|EPI_ISL_13412303|2022-05-28
hCoV-19/Canada/BC-BCCDC-450653/2022|EPI_ISL_13412392|2022-05-26
hCoV-19/Canada/BC-BCCDC-450661/2022|EPI_ISL_13412397|2022-05-27
hCoV-19/Canada/BC-BCCDC-455047/2022|EPI_ISL_13526333|2022-06-08
hCoV-19/Canada/BC-BCCDC-455158/2022|EPI_ISL_13526415|2022-06-09
hCoV-19/Canada/BC-BCCDC-455231/2022|EPI_ISL_13526451|2022-06-11

hCoV-19/Canada/BC-BCCDC-455252/2022|EPI_ISL_13526470|2022-06-09
hCoV-19/Canada/BC-BCCDC-455318/2022|EPI_ISL_13526516|2022-06-10
hCoV-19/Canada/BC-BCCDC-465396/2022|EPI_ISL_14760875|2022-06-09
hCoV-19/Canada/BC-BCCDC-465470/2022|EPI_ISL_14760917|2022-06-12
hCoV-19/Canada/BC-BCCDC-465530/2022|EPI_ISL_14760971|2022-06-11
hCoV-19/Canada/BC-BCCDC-474715/2022|EPI_ISL_14763946|2022-07-13
hCoV-19/Canada/BC-BCCDC-482226/2022|EPI_ISL_14765603|2022-07-21
hCoV-19/Canada/BC-BCCDC-482258/2022|EPI_ISL_14765626|2022-07-18
hCoV-19/Canada/BC-BCCDC-482584/2022|EPI_ISL_14765701|2022-07-23
hCoV-19/Canada/BC-BCCDC-499457/2022|EPI_ISL_15050063|2022-08-06
hCoV-19/Canada/BC-BCCDC-506081/2022|EPI_ISL_15051842|2022-08-28
hCoV-19/Canada/BC-BCCDC-506317/2022|EPI_ISL_15051965|2022-08-28
hCoV-19/Canada/BC-BCCDC-515562/2022|EPI_ISL_15184830|2022-09-01
hCoV-19/Canada/BC-BCCDC-535249/2022|EPI_ISL_15659982|2022-10-12
hCoV-19/Canada/BC-BCCDC-8608/2020|EPI_ISL_740850|2020-11-29
hCoV-19/Canada/ON-KHS-21-01154-v1/2021|EPI_ISL_13836370|2021-03-02
hCoV-19/Canada/ON-KHS-21-02282-v1/2021|EPI_ISL_13837278|2021-06-17
hCoV-19/Canada/ON-PHL-22-49999/2022|EPI_ISL_15690812|2022-10-31
hCoV-19/Canada/ON-S1922/2020|EPI_ISL_729330|2020-11-24
hCoV-19/Canada/QC-1nDJXW-488959081/2021|EPI_ISL_3207744|2021-05-14
hCoV-19/Canada/QC-1nIEOQ-7051284799/2020|EPI_ISL_7684747|2020-08-28
hCoV-19/Canada/QC-1nIFO-S60532021A/2020|EPI_ISL_7685781|2020-10-05
hCoV-19/Canada/QC-1nIUH-5524383248/2021|EPI_ISL_2975891|2021-04-21
hCoV-19/Canada/SK-NML-132341/2021|EPI_ISL_16160020|2021-04-23
hCoV-19/Canada/SK-NML-741/2020|EPI_ISL_2883739|2020-06-18
hCoV-19/Canary_Islands/CN-HUGCDN-02412554-08/2021|EPI_ISL_16215487|2021-08-10
hCoV-19/Canary_Islands/CN-HUNSC_ITER_150131920/2021|EPI_ISL_14073782|2021-02-14
hCoV-19/Canary_Islands/CN-HUNSC_ITER_900213334/2021|EPI_ISL_14091818|2021-03-24
hCoV-19/Canary_Islands/CN-HUNSC_ITER_900280991/2021|EPI_ISL_14100909|2021-06-30
hCoV-19/Chile/LL-ISPC-127242/2021|EPI_ISL_17028680|2021-07-16
hCoV-19/Chile/RM-263441/2020|EPI_ISL_1167914|2020-12-27
hCoV-19/Chile/RM-ISPC-197930/2021|EPI_ISL_17028963|2021-12-26
hCoV-19/Chile/RM-ISPC-81689/2021|EPI_ISL_17028371|2021-04-20
hCoV-19/Colombia/DC-INS-VG-25592/2022|EPI_ISL_15826554|2022-10-28
hCoV-19/Colombia/QUI-SYN-14503009123/2022|EPI_ISL_15977733|2022-04-05
hCoV-19/Colombia/VAC-CSB-31573352/2022|EPI_ISL_16131173|2022-08-14
hCoV-19/Colombia/VAC-LSG-22040100852-86/2022|EPI_ISL_15977711|2022-04-01
hCoV-19/Costa_Rica/INC-0185/2020|EPI_ISL_770005|2020-11-25
hCoV-19/Costa_Rica/INC-3992-751910/2022|EPI_ISL_12572734|2022-04-13
hCoV-19/Costa_Rica/INC-4332-754491/2022|EPI_ISL_12935301|2022-05-03
hCoV-19/Costa_Rica/INC-4825-757962/2022|EPI_ISL_13337891|2022-05-27
hCoV-19/Croatia/28461/2022|EPI_ISL_9975481|2022-02-03
hCoV-19/Croatia/28537/2022|EPI_ISL_9975932|2022-02-01

hCoV-19/Croatia/28710/2022|EPI_ISL_9975642|2022-02-01
hCoV-19/Croatia/28712/2022|EPI_ISL_9975644|2022-02-01
hCoV-19/Croatia/32626/2022|EPI_ISL_11342064|2022-03-02
hCoV-19/Croatia/32726/2022|EPI_ISL_11342183|2022-03-01
hCoV-19/Croatia/37050/2022|EPI_ISL_13032588|2022-05-03
hCoV-19/Croatia/37618/2022|EPI_ISL_13477837|2022-05-25
hCoV-19/Croatia/41113/2022|EPI_ISL_14589997|2022-08-06
hCoV-19/Croatia/42715/2022|EPI_ISL_15036444|2022-08-29
hCoV-19/Cyprus/BMV1082/2021|EPI_ISL_16372378|2021-04-27
hCoV-19/Cyprus/BMV167/2020|EPI_ISL_16372706|2020-12-31
hCoV-19/Cyprus/BMV2185/2021|EPI_ISL_16368788|2021-09-17
hCoV-19/Cyprus/BMV2335/2021|EPI_ISL_16372947|2021-10-05
hCoV-19/Cyprus/BMV362/2021|EPI_ISL_16373055|2021-01-28
hCoV-19/Cyprus/Cy145846/2020|EPI_ISL_1164725|2020-12-29
hCoV-19/Czech_Republic/CSQ6710/2022|EPI_ISL_10993666|2022-03-01
hCoV-19/Czech_Republic/CSQ9467/2022|EPI_ISL_14946134|2022-08-30
hCoV-19/Czech_Republic/FNHK-Ps-007003/2022|EPI_ISL_15813068|2022-10-17
hCoV-19/Czech_Republic/FNO-220470/2022|EPI_ISL_12785568|2022-05-03
hCoV-19/Czech_Republic/NRL_11156/2020|EPI_ISL_792691|2020-11-15
hCoV-19/Czech_Republic/NRL_s5487/2022|EPI_ISL_15058491|2022-09-02
hCoV-19/Czech_Republic/NRL_s7108/2022|EPI_ISL_16013868|2022-11-10
hCoV-19/Czech_Republic/UMTM467334/2022|EPI_ISL_12157001|2022-03-16
hCoV-19/Denmark/DCGC-298150/2022|EPI_ISL_8465307|2022-01-02
hCoV-19/Denmark/DCGC-298205/2022|EPI_ISL_8465322|2022-01-02
hCoV-19/Denmark/DCGC-298591/2022|EPI_ISL_8465418|2022-01-02
hCoV-19/Denmark/DCGC-298964/2022|EPI_ISL_8465513|2022-01-02
hCoV-19/Denmark/DCGC-301364/2022|EPI_ISL_8529718|2022-01-02
hCoV-19/Denmark/DCGC-301508/2022|EPI_ISL_8529806|2022-01-02
hCoV-19/Denmark/DCGC-304925/2022|EPI_ISL_8582139|2022-01-03
hCoV-19/Denmark/DCGC-306438/2022|EPI_ISL_8583434|2022-01-03
hCoV-19/Denmark/DCGC-306680/2022|EPI_ISL_8583616|2022-01-03
hCoV-19/Denmark/DCGC-316304/2022|EPI_ISL_8833220|2022-01-03
hCoV-19/Denmark/DCGC-351148/2022|EPI_ISL_9650695|2022-02-01
hCoV-19/Denmark/DCGC-351157/2022|EPI_ISL_9650704|2022-02-02
hCoV-19/Denmark/DCGC-351625/2022|EPI_ISL_9651697|2022-02-02
hCoV-19/Denmark/DCGC-351702/2022|EPI_ISL_9651800|2022-02-01
hCoV-19/Denmark/DCGC-351752/2022|EPI_ISL_9651849|2022-02-02
hCoV-19/Denmark/DCGC-351756/2022|EPI_ISL_9651853|2022-02-01
hCoV-19/Denmark/DCGC-352372/2022|EPI_ISL_9662188|2022-02-01
hCoV-19/Denmark/DCGC-353804/2022|EPI_ISL_9653933|2022-02-01
hCoV-19/Denmark/DCGC-354486/2022|EPI_ISL_9654707|2022-02-02
hCoV-19/Denmark/DCGC-355161/2022|EPI_ISL_9655626|2022-02-02
hCoV-19/Denmark/DCGC-355576/2022|EPI_ISL_9690243|2022-02-01

hCoV-19/Denmark/DCGC-355930/2022|EPI_ISL_9690946|2022-02-02
hCoV-19/Denmark/DCGC-355988/2022|EPI_ISL_9691049|2022-02-03
hCoV-19/Denmark/DCGC-356393/2022|EPI_ISL_9691736|2022-02-02
hCoV-19/Denmark/DCGC-356685/2022|EPI_ISL_9692167|2022-02-03
hCoV-19/Denmark/DCGC-356701/2022|EPI_ISL_9692196|2022-02-01
hCoV-19/Denmark/DCGC-356725/2022|EPI_ISL_9692240|2022-02-03
hCoV-19/Denmark/DCGC-357042/2022|EPI_ISL_9694012|2022-02-03
hCoV-19/Denmark/DCGC-357331/2022|EPI_ISL_9694681|2022-02-02
hCoV-19/Denmark/DCGC-357497/2022|EPI_ISL_9739535|2022-02-01
hCoV-19/Denmark/DCGC-357635/2022|EPI_ISL_9739879|2022-02-02
hCoV-19/Denmark/DCGC-357721/2022|EPI_ISL_9740129|2022-02-03
hCoV-19/Denmark/DCGC-357928/2022|EPI_ISL_9740835|2022-02-04
hCoV-19/Denmark/DCGC-357970/2022|EPI_ISL_9740963|2022-02-02
hCoV-19/Denmark/DCGC-357985/2022|EPI_ISL_9741010|2022-02-02
hCoV-19/Denmark/DCGC-358287/2022|EPI_ISL_9742034|2022-02-03
hCoV-19/Denmark/DCGC-358383/2022|EPI_ISL_9742251|2022-02-01
hCoV-19/Denmark/DCGC-358389/2022|EPI_ISL_9742269|2022-02-04
hCoV-19/Denmark/DCGC-358600/2022|EPI_ISL_9742991|2022-02-03
hCoV-19/Denmark/DCGC-358651/2022|EPI_ISL_9743146|2022-02-01
hCoV-19/Denmark/DCGC-358860/2022|EPI_ISL_9743735|2022-02-03
hCoV-19/Denmark/DCGC-359024/2022|EPI_ISL_9744216|2022-02-02
hCoV-19/Denmark/DCGC-359208/2022|EPI_ISL_9744970|2022-02-02
hCoV-19/Denmark/DCGC-359284/2022|EPI_ISL_9745200|2022-02-01
hCoV-19/Denmark/DCGC-359405/2022|EPI_ISL_9745582|2022-02-04
hCoV-19/Denmark/DCGC-359422/2022|EPI_ISL_9745632|2022-02-04
hCoV-19/Denmark/DCGC-359451/2022|EPI_ISL_9745719|2022-02-03
hCoV-19/Denmark/DCGC-359588/2022|EPI_ISL_9746130|2022-02-02
hCoV-19/Denmark/DCGC-359915/2022|EPI_ISL_9782044|2022-02-03
hCoV-19/Denmark/DCGC-359919/2022|EPI_ISL_9782060|2022-02-03
hCoV-19/Denmark/DCGC-359988/2022|EPI_ISL_9782439|2022-02-03
hCoV-19/Denmark/DCGC-360104/2022|EPI_ISL_9782861|2022-02-04
hCoV-19/Denmark/DCGC-360190/2022|EPI_ISL_9783183|2022-02-04
hCoV-19/Denmark/DCGC-360220/2022|EPI_ISL_9783303|2022-02-05
hCoV-19/Denmark/DCGC-360244/2022|EPI_ISL_9783400|2022-02-03
hCoV-19/Denmark/DCGC-360293/2022|EPI_ISL_9783565|2022-02-04
hCoV-19/Denmark/DCGC-360349/2022|EPI_ISL_9783791|2022-02-04
hCoV-19/Denmark/DCGC-360435/2022|EPI_ISL_9784102|2022-02-03
hCoV-19/Denmark/DCGC-360490/2022|EPI_ISL_9784290|2022-02-05
hCoV-19/Denmark/DCGC-360595/2022|EPI_ISL_9784581|2022-02-03
hCoV-19/Denmark/DCGC-360596/2022|EPI_ISL_9784582|2022-02-03
hCoV-19/Denmark/DCGC-360665/2022|EPI_ISL_9784824|2022-02-03
hCoV-19/Denmark/DCGC-360726/2022|EPI_ISL_9785057|2022-02-05
hCoV-19/Denmark/DCGC-360750/2022|EPI_ISL_9785156|2022-02-03

hCoV-19/Denmark/DCGC-361289/2022|EPI_ISL_9787525|2022-02-03
hCoV-19/Denmark/DCGC-361401/2022|EPI_ISL_9787998|2022-02-03
hCoV-19/Denmark/DCGC-361490/2022|EPI_ISL_9788318|2022-02-03
hCoV-19/Denmark/DCGC-361615/2022|EPI_ISL_9788773|2022-02-03
hCoV-19/Denmark/DCGC-361857/2022|EPI_ISL_9789429|2022-02-04
hCoV-19/Denmark/DCGC-361872/2022|EPI_ISL_9789469|2022-02-05
hCoV-19/Denmark/DCGC-361902/2022|EPI_ISL_9789551|2022-02-03
hCoV-19/Denmark/DCGC-362027/2022|EPI_ISL_9854816|2022-02-05
hCoV-19/Denmark/DCGC-362051/2022|EPI_ISL_9854856|2022-02-04
hCoV-19/Denmark/DCGC-362147/2022|EPI_ISL_9854951|2022-02-04
hCoV-19/Denmark/DCGC-362251/2022|EPI_ISL_9855115|2022-02-04
hCoV-19/Denmark/DCGC-362348/2022|EPI_ISL_9855212|2022-02-05
hCoV-19/Denmark/DCGC-362357/2022|EPI_ISL_9855221|2022-02-05
hCoV-19/Denmark/DCGC-362430/2022|EPI_ISL_9855748|2022-02-04
hCoV-19/Denmark/DCGC-362460/2022|EPI_ISL_9855778|2022-02-05
hCoV-19/Denmark/DCGC-362521/2022|EPI_ISL_9855837|2022-02-05
hCoV-19/Denmark/DCGC-362635/2022|EPI_ISL_9855950|2022-02-03
hCoV-19/Denmark/DCGC-362782/2022|EPI_ISL_9856094|2022-02-04
hCoV-19/Denmark/DCGC-362838/2022|EPI_ISL_9856148|2022-02-04
hCoV-19/Denmark/DCGC-362839/2022|EPI_ISL_9856149|2022-02-04
hCoV-19/Denmark/DCGC-362902/2022|EPI_ISL_9856284|2022-02-04
hCoV-19/Denmark/DCGC-362913/2022|EPI_ISL_9856296|2022-02-05
hCoV-19/Denmark/DCGC-363002/2022|EPI_ISL_9856385|2022-02-05
hCoV-19/Denmark/DCGC-363226/2022|EPI_ISL_9857227|2022-02-01
hCoV-19/Denmark/DCGC-363292/2022|EPI_ISL_9857404|2022-02-04
hCoV-19/Denmark/DCGC-363335/2022|EPI_ISL_9857446|2022-02-04
hCoV-19/Denmark/DCGC-363347/2022|EPI_ISL_9857458|2022-02-04
hCoV-19/Denmark/DCGC-363375/2022|EPI_ISL_9857566|2022-02-04
hCoV-19/Denmark/DCGC-363397/2022|EPI_ISL_9857587|2022-02-04
hCoV-19/Denmark/DCGC-363426/2022|EPI_ISL_9857616|2022-02-03
hCoV-19/Denmark/DCGC-363444/2022|EPI_ISL_9857633|2022-02-05
hCoV-19/Denmark/DCGC-363448/2022|EPI_ISL_9857637|2022-02-05
hCoV-19/Denmark/DCGC-363473/2022|EPI_ISL_9857661|2022-02-04
hCoV-19/Denmark/DCGC-363505/2022|EPI_ISL_9857693|2022-02-04
hCoV-19/Denmark/DCGC-363545/2022|EPI_ISL_9857733|2022-02-04
hCoV-19/Denmark/DCGC-363566/2022|EPI_ISL_9857754|2022-02-04
hCoV-19/Denmark/DCGC-363742/2022|EPI_ISL_9857925|2022-02-05
hCoV-19/Denmark/DCGC-363777/2022|EPI_ISL_9857960|2022-02-04
hCoV-19/Denmark/DCGC-363792/2022|EPI_ISL_9857975|2022-02-05
hCoV-19/Denmark/DCGC-363828/2022|EPI_ISL_9858010|2022-02-04
hCoV-19/Denmark/DCGC-363886/2022|EPI_ISL_9858068|2022-02-05
hCoV-19/Denmark/DCGC-363903/2022|EPI_ISL_9858188|2022-02-04
hCoV-19/Denmark/DCGC-363911/2022|EPI_ISL_9858196|2022-02-05

hCoV-19/Denmark/DCGC-363946/2022|EPI_ISL_9858237|2022-02-04
hCoV-19/Denmark/DCGC-364093/2022|EPI_ISL_9859486|2022-02-05
hCoV-19/Denmark/DCGC-364144/2022|EPI_ISL_9859536|2022-02-05
hCoV-19/Denmark/DCGC-365236/2022|EPI_ISL_9900518|2022-02-05
hCoV-19/Denmark/DCGC-365717/2022|EPI_ISL_9902542|2022-02-05
hCoV-19/Denmark/DCGC-365739/2022|EPI_ISL_9902616|2022-02-03
hCoV-19/Denmark/DCGC-366006/2022|EPI_ISL_9903497|2022-02-05
hCoV-19/Denmark/DCGC-366274/2022|EPI_ISL_9903990|2022-02-04
hCoV-19/Denmark/DCGC-366373/2022|EPI_ISL_9904088|2022-02-02
hCoV-19/Denmark/DCGC-366607/2022|EPI_ISL_9904430|2022-02-05
hCoV-19/Denmark/DCGC-366912/2022|EPI_ISL_9904724|2022-02-04
hCoV-19/Denmark/DCGC-366920/2022|EPI_ISL_9904732|2022-02-05
hCoV-19/Denmark/DCGC-366973/2022|EPI_ISL_9904784|2022-02-05
hCoV-19/Denmark/DCGC-368484/2022|EPI_ISL_10010962|2022-02-04
hCoV-19/Denmark/DCGC-369455/2022|EPI_ISL_10014049|2022-02-04
hCoV-19/Denmark/DCGC-369745/2022|EPI_ISL_10015015|2022-02-04
hCoV-19/Denmark/DCGC-371467/2022|EPI_ISL_10062170|2022-02-05
hCoV-19/Denmark/DCGC-377183/2022|EPI_ISL_10201127|2022-02-02
hCoV-19/Denmark/DCGC-377189/2022|EPI_ISL_10201144|2022-02-16
hCoV-19/Denmark/DCGC-377281/2022|EPI_ISL_10201424|2022-02-04
hCoV-19/Denmark/DCGC-377925/2022|EPI_ISL_10203674|2022-02-16
hCoV-19/Denmark/DCGC-380326/2022|EPI_ISL_10208979|2022-02-16
hCoV-19/Denmark/DCGC-380724/2022|EPI_ISL_10209868|2022-02-04
hCoV-19/Denmark/DCGC-380873/2022|EPI_ISL_10210047|2022-02-16
hCoV-19/Denmark/DCGC-381085/2022|EPI_ISL_10210845|2022-02-16
hCoV-19/Denmark/DCGC-382041/2022|EPI_ISL_10216265|2022-02-02
hCoV-19/Denmark/DCGC-382604/2022|EPI_ISL_10216823|2022-02-16
hCoV-19/Denmark/DCGC-383504/2022|EPI_ISL_10253004|2022-02-17
hCoV-19/Denmark/DCGC-383612/2022|EPI_ISL_10254043|2022-02-17
hCoV-19/Denmark/DCGC-383630/2022|EPI_ISL_10254071|2022-02-17
hCoV-19/Denmark/DCGC-383662/2022|EPI_ISL_10254103|2022-02-17
hCoV-19/Denmark/DCGC-383693/2022|EPI_ISL_10255134|2022-02-17
hCoV-19/Denmark/DCGC-383836/2022|EPI_ISL_10255409|2022-02-17
hCoV-19/Denmark/DCGC-384061/2022|EPI_ISL_10257589|2022-02-16
hCoV-19/Denmark/DCGC-384322/2022|EPI_ISL_10258020|2022-02-16
hCoV-19/Denmark/DCGC-384596/2022|EPI_ISL_10259036|2022-02-17
hCoV-19/Denmark/DCGC-384752/2022|EPI_ISL_10259377|2022-02-17
hCoV-19/Denmark/DCGC-384811/2022|EPI_ISL_10259489|2022-02-17
hCoV-19/Denmark/DCGC-384915/2022|EPI_ISL_10259773|2022-02-17
hCoV-19/Denmark/DCGC-384947/2022|EPI_ISL_10260111|2022-02-17
hCoV-19/Denmark/DCGC-385305/2022|EPI_ISL_10260936|2022-02-17
hCoV-19/Denmark/DCGC-385321/2022|EPI_ISL_10260963|2022-02-17
hCoV-19/Denmark/DCGC-385440/2022|EPI_ISL_10317915|2022-02-17

hCoV-19/Denmark/DCGC-386874/2022|EPI_ISL_10348925|2022-02-17
hCoV-19/Denmark/DCGC-387334/2022|EPI_ISL_10349750|2022-02-16
hCoV-19/Denmark/DCGC-387398/2022|EPI_ISL_10349872|2022-02-17
hCoV-19/Denmark/DCGC-387674/2022|EPI_ISL_10350406|2022-02-16
hCoV-19/Denmark/DCGC-387795/2022|EPI_ISL_10350634|2022-02-16
hCoV-19/Denmark/DCGC-388323/2022|EPI_ISL_10352855|2022-02-16
hCoV-19/Denmark/DCGC-388597/2022|EPI_ISL_10353335|2022-02-17
hCoV-19/Denmark/DCGC-388700/2022|EPI_ISL_10353506|2022-02-17
hCoV-19/Denmark/DCGC-391981/2022|EPI_ISL_10506346|2022-02-17
hCoV-19/Denmark/DCGC-392118/2022|EPI_ISL_10506598|2022-02-17
hCoV-19/Denmark/DCGC-393281/2022|EPI_ISL_10509751|2022-02-17
hCoV-19/Denmark/DCGC-393770/2022|EPI_ISL_10510939|2022-02-17
hCoV-19/Denmark/DCGC-393774/2022|EPI_ISL_10510943|2022-02-17
hCoV-19/Denmark/DCGC-394299/2022|EPI_ISL_10511541|2022-02-16
hCoV-19/Denmark/DCGC-395078/2022|EPI_ISL_10582271|2022-02-17
hCoV-19/Denmark/DCGC-405019/2022|EPI_ISL_10806069|2022-03-01
hCoV-19/Denmark/DCGC-405043/2022|EPI_ISL_10806093|2022-03-02
hCoV-19/Denmark/DCGC-405151/2022|EPI_ISL_10806201|2022-03-01
hCoV-19/Denmark/DCGC-405314/2022|EPI_ISL_10806362|2022-03-01
hCoV-19/Denmark/DCGC-405323/2022|EPI_ISL_10806371|2022-03-02
hCoV-19/Denmark/DCGC-405332/2022|EPI_ISL_10806380|2022-03-01
hCoV-19/Denmark/DCGC-405335/2022|EPI_ISL_10806383|2022-03-01
hCoV-19/Denmark/DCGC-405709/2022|EPI_ISL_10806912|2022-03-01
hCoV-19/Denmark/DCGC-405733/2022|EPI_ISL_10806936|2022-03-02
hCoV-19/Denmark/DCGC-405737/2022|EPI_ISL_10806940|2022-03-01
hCoV-19/Denmark/DCGC-405920/2022|EPI_ISL_10807212|2022-03-02
hCoV-19/Denmark/DCGC-406014/2022|EPI_ISL_10807305|2022-03-02
hCoV-19/Denmark/DCGC-406016/2022|EPI_ISL_10807307|2022-03-01
hCoV-19/Denmark/DCGC-406027/2022|EPI_ISL_10807318|2022-03-01
hCoV-19/Denmark/DCGC-406134/2022|EPI_ISL_10807505|2022-03-02
hCoV-19/Denmark/DCGC-406276/2022|EPI_ISL_10807646|2022-03-02
hCoV-19/Denmark/DCGC-406370/2022|EPI_ISL_10807739|2022-03-01
hCoV-19/Denmark/DCGC-406427/2022|EPI_ISL_10807797|2022-03-01
hCoV-19/Denmark/DCGC-406430/2022|EPI_ISL_10807814|2022-03-01
hCoV-19/Denmark/DCGC-406544/2022|EPI_ISL_10807928|2022-03-01
hCoV-19/Denmark/DCGC-406828/2022|EPI_ISL_10808310|2022-03-02
hCoV-19/Denmark/DCGC-406862/2022|EPI_ISL_10808342|2022-03-01
hCoV-19/Denmark/DCGC-406918/2022|EPI_ISL_10808398|2022-03-02
hCoV-19/Denmark/DCGC-406961/2022|EPI_ISL_10808441|2022-03-01
hCoV-19/Denmark/DCGC-407143/2022|EPI_ISL_10809138|2022-03-01
hCoV-19/Denmark/DCGC-407381/2022|EPI_ISL_10809558|2022-03-02
hCoV-19/Denmark/DCGC-407535/2022|EPI_ISL_10809752|2022-03-01
hCoV-19/Denmark/DCGC-407645/2022|EPI_ISL_10809861|2022-03-01

hCoV-19/Denmark/DCGC-407739/2022|EPI_ISL_10809982|2022-03-01
hCoV-19/Denmark/DCGC-407744/2022|EPI_ISL_10809987|2022-03-02
hCoV-19/Denmark/DCGC-407835/2022|EPI_ISL_10810078|2022-03-01
hCoV-19/Denmark/DCGC-408173/2022|EPI_ISL_10810581|2022-03-01
hCoV-19/Denmark/DCGC-408354/2022|EPI_ISL_10810781|2022-03-01
hCoV-19/Denmark/DCGC-408393/2022|EPI_ISL_10810820|2022-03-01
hCoV-19/Denmark/DCGC-408429/2022|EPI_ISL_10810855|2022-03-02
hCoV-19/Denmark/DCGC-408439/2022|EPI_ISL_10810865|2022-03-02
hCoV-19/Denmark/DCGC-408507/2022|EPI_ISL_10811017|2022-03-01
hCoV-19/Denmark/DCGC-408566/2022|EPI_ISL_10811076|2022-03-01
hCoV-19/Denmark/DCGC-408586/2022|EPI_ISL_10811096|2022-03-02
hCoV-19/Denmark/DCGC-408820/2022|EPI_ISL_10811325|2022-03-01
hCoV-19/Denmark/DCGC-409487/2022|EPI_ISL_10812152|2022-03-02
hCoV-19/Denmark/DCGC-409523/2022|EPI_ISL_10812188|2022-03-01
hCoV-19/Denmark/DCGC-409571/2022|EPI_ISL_10812298|2022-03-01
hCoV-19/Denmark/DCGC-409613/2022|EPI_ISL_10812339|2022-03-01
hCoV-19/Denmark/DCGC-409632/2022|EPI_ISL_10812358|2022-03-02
hCoV-19/Denmark/DCGC-409715/2022|EPI_ISL_10812809|2022-03-01
hCoV-19/Denmark/DCGC-409759/2022|EPI_ISL_10812853|2022-03-01
hCoV-19/Denmark/DCGC-409762/2022|EPI_ISL_10812856|2022-03-01
hCoV-19/Denmark/DCGC-409782/2022|EPI_ISL_10812876|2022-03-01
hCoV-19/Denmark/DCGC-409870/2022|EPI_ISL_10813050|2022-03-02
hCoV-19/Denmark/DCGC-409978/2022|EPI_ISL_10813158|2022-03-01
hCoV-19/Denmark/DCGC-409987/2022|EPI_ISL_10813167|2022-03-02
hCoV-19/Denmark/DCGC-410044/2022|EPI_ISL_10813234|2022-03-02
hCoV-19/Denmark/DCGC-410081/2022|EPI_ISL_10813271|2022-03-01
hCoV-19/Denmark/DCGC-410307/2022|EPI_ISL_10813844|2022-03-02
hCoV-19/Denmark/DCGC-410586/2022|EPI_ISL_10814417|2022-03-02
hCoV-19/Denmark/DCGC-410656/2022|EPI_ISL_10814486|2022-03-01
hCoV-19/Denmark/DCGC-410683/2022|EPI_ISL_10814513|2022-03-02
hCoV-19/Denmark/DCGC-410688/2022|EPI_ISL_10814518|2022-03-01
hCoV-19/Denmark/DCGC-410701/2022|EPI_ISL_10814530|2022-03-02
hCoV-19/Denmark/DCGC-410713/2022|EPI_ISL_10814542|2022-03-01
hCoV-19/Denmark/DCGC-412782/2022|EPI_ISL_10891124|2022-03-02
hCoV-19/Denmark/DCGC-413109/2022|EPI_ISL_10891623|2022-03-02
hCoV-19/Denmark/DCGC-413289/2022|EPI_ISL_10891808|2022-03-02
hCoV-19/Denmark/DCGC-413307/2022|EPI_ISL_10891826|2022-03-02
hCoV-19/Denmark/DCGC-413327/2022|EPI_ISL_10891846|2022-03-02
hCoV-19/Denmark/DCGC-413550/2022|EPI_ISL_10892115|2022-03-02
hCoV-19/Denmark/DCGC-413678/2022|EPI_ISL_10892340|2022-03-02
hCoV-19/Denmark/DCGC-413753/2022|EPI_ISL_10892415|2022-03-02
hCoV-19/Denmark/DCGC-413946/2022|EPI_ISL_10892611|2022-03-02
hCoV-19/Denmark/DCGC-414033/2022|EPI_ISL_10892796|2022-03-02

hCoV-19/Denmark/DCGC-414413/2022|EPI_ISL_10893175|2022-03-02
hCoV-19/Denmark/DCGC-414504/2022|EPI_ISL_10893267|2022-03-02
hCoV-19/Denmark/DCGC-414692/2022|EPI_ISL_10893457|2022-03-02
hCoV-19/Denmark/DCGC-414864/2022|EPI_ISL_10893637|2022-03-02
hCoV-19/Denmark/DCGC-414895/2022|EPI_ISL_10893669|2022-03-02
hCoV-19/Denmark/DCGC-415830/2022|EPI_ISL_10935095|2022-03-02
hCoV-19/Denmark/DCGC-415873/2022|EPI_ISL_10935209|2022-03-02
hCoV-19/Denmark/DCGC-416082/2022|EPI_ISL_10937907|2022-03-02
hCoV-19/Denmark/DCGC-420037/2022|EPI_ISL_11025672|2022-02-16
hCoV-19/Denmark/DCGC-424227/2022|EPI_ISL_11065443|2022-03-01
hCoV-19/Denmark/DCGC-434037/2022|EPI_ISL_11273211|2022-03-16
hCoV-19/Denmark/DCGC-434455/2022|EPI_ISL_11274514|2022-03-16
hCoV-19/Denmark/DCGC-434558/2022|EPI_ISL_11274747|2022-03-16
hCoV-19/Denmark/DCGC-434752/2022|EPI_ISL_11275445|2022-03-16
hCoV-19/Denmark/DCGC-435169/2022|EPI_ISL_11276778|2022-03-16
hCoV-19/Denmark/DCGC-435709/2022|EPI_ISL_11278580|2022-03-16
hCoV-19/Denmark/DCGC-435889/2022|EPI_ISL_11279246|2022-03-16
hCoV-19/Denmark/DCGC-436609/2022|EPI_ISL_11281523|2022-03-16
hCoV-19/Denmark/DCGC-437010/2022|EPI_ISL_11282704|2022-03-16
hCoV-19/Denmark/DCGC-437756/2022|EPI_ISL_11284977|2022-03-16
hCoV-19/Denmark/DCGC-437950/2022|EPI_ISL_11285545|2022-03-16
hCoV-19/Denmark/DCGC-437952/2022|EPI_ISL_11285547|2022-03-16
hCoV-19/Denmark/DCGC-438285/2022|EPI_ISL_11286470|2022-03-16
hCoV-19/Denmark/DCGC-439569/2022|EPI_ISL_11316964|2022-03-17
hCoV-19/Denmark/DCGC-439578/2022|EPI_ISL_11316974|2022-03-17
hCoV-19/Denmark/DCGC-439707/2022|EPI_ISL_11317102|2022-03-17
hCoV-19/Denmark/DCGC-439821/2022|EPI_ISL_11317216|2022-03-17
hCoV-19/Denmark/DCGC-439885/2022|EPI_ISL_11317622|2022-03-17
hCoV-19/Denmark/DCGC-440117/2022|EPI_ISL_11318448|2022-03-17
hCoV-19/Denmark/DCGC-440229/2022|EPI_ISL_11318559|2022-03-17
hCoV-19/Denmark/DCGC-440276/2022|EPI_ISL_11318606|2022-03-17
hCoV-19/Denmark/DCGC-440334/2022|EPI_ISL_11318664|2022-03-17
hCoV-19/Denmark/DCGC-440477/2022|EPI_ISL_11345215|2022-03-16
hCoV-19/Denmark/DCGC-440665/2022|EPI_ISL_11345410|2022-03-17
hCoV-19/Denmark/DCGC-440732/2022|EPI_ISL_11345476|2022-03-17
hCoV-19/Denmark/DCGC-440741/2022|EPI_ISL_11345485|2022-03-17
hCoV-19/Denmark/DCGC-440770/2022|EPI_ISL_11345514|2022-03-17
hCoV-19/Denmark/DCGC-440964/2022|EPI_ISL_11345707|2022-03-17
hCoV-19/Denmark/DCGC-440989/2022|EPI_ISL_11345732|2022-03-17
hCoV-19/Denmark/DCGC-441154/2022|EPI_ISL_11345897|2022-03-17
hCoV-19/Denmark/DCGC-441433/2022|EPI_ISL_11346585|2022-03-17
hCoV-19/Denmark/DCGC-441442/2022|EPI_ISL_11346594|2022-03-17
hCoV-19/Denmark/DCGC-441823/2022|EPI_ISL_11346974|2022-03-17

hCoV-19/Denmark/DCGC-441888/2022|EPI_ISL_11347101|2022-03-17
hCoV-19/Denmark/DCGC-442328/2022|EPI_ISL_11347606|2022-03-16
hCoV-19/Denmark/DCGC-442341/2022|EPI_ISL_11347619|2022-03-16
hCoV-19/Denmark/DCGC-442445/2022|EPI_ISL_11347723|2022-03-16
hCoV-19/Denmark/DCGC-442660/2022|EPI_ISL_11347935|2022-03-17
hCoV-19/Denmark/DCGC-442680/2022|EPI_ISL_11347955|2022-03-16
hCoV-19/Denmark/DCGC-442717/2022|EPI_ISL_11347992|2022-03-17
hCoV-19/Denmark/DCGC-442839/2022|EPI_ISL_11348171|2022-03-17
hCoV-19/Denmark/DCGC-442879/2022|EPI_ISL_11379167|2022-03-17
hCoV-19/Denmark/DCGC-443012/2022|EPI_ISL_11379299|2022-03-17
hCoV-19/Denmark/DCGC-443086/2022|EPI_ISL_11379373|2022-03-17
hCoV-19/Denmark/DCGC-443150/2022|EPI_ISL_11379437|2022-03-17
hCoV-19/Denmark/DCGC-443313/2022|EPI_ISL_11379599|2022-03-17
hCoV-19/Denmark/DCGC-443657/2022|EPI_ISL_11380042|2022-03-17
hCoV-19/Denmark/DCGC-443709/2022|EPI_ISL_11380095|2022-03-17
hCoV-19/Denmark/DCGC-443849/2022|EPI_ISL_11380377|2022-03-17
hCoV-19/Denmark/DCGC-444114/2022|EPI_ISL_11380642|2022-03-17
hCoV-19/Denmark/DCGC-444399/2022|EPI_ISL_11381044|2022-03-17
hCoV-19/Denmark/DCGC-444622/2022|EPI_ISL_11381372|2022-03-17
hCoV-19/Denmark/DCGC-444650/2022|EPI_ISL_11381400|2022-03-17
hCoV-19/Denmark/DCGC-444736/2022|EPI_ISL_11381485|2022-03-17
hCoV-19/Denmark/DCGC-444910/2022|EPI_ISL_11381659|2022-03-17
hCoV-19/Denmark/DCGC-444985/2022|EPI_ISL_11381733|2022-03-17
hCoV-19/Denmark/DCGC-445037/2022|EPI_ISL_11381785|2022-03-17
hCoV-19/Denmark/DCGC-446009/2022|EPI_ISL_11443126|2022-03-16
hCoV-19/Denmark/DCGC-446667/2022|EPI_ISL_11443836|2022-03-16
hCoV-19/Denmark/DCGC-447945/2022|EPI_ISL_11514788|2022-03-16
hCoV-19/Denmark/DCGC-447971/2022|EPI_ISL_11514813|2022-03-16
hCoV-19/Denmark/DCGC-448106/2022|EPI_ISL_11514945|2022-03-16
hCoV-19/Denmark/DCGC-448253/2022|EPI_ISL_11515281|2022-02-03
hCoV-19/Denmark/DCGC-448640/2022|EPI_ISL_11515774|2022-03-16
hCoV-19/Denmark/DCGC-448666/2022|EPI_ISL_11515800|2022-03-16
hCoV-19/Denmark/DCGC-448954/2022|EPI_ISL_11516211|2022-02-03
hCoV-19/Denmark/DCGC-449180/2022|EPI_ISL_11516470|2022-03-16
hCoV-19/Denmark/DCGC-449538/2022|EPI_ISL_11517175|2022-02-01
hCoV-19/Denmark/DCGC-449758/2022|EPI_ISL_11517696|2022-03-16
hCoV-19/Denmark/DCGC-449786/2022|EPI_ISL_11517751|2022-03-16
hCoV-19/Denmark/DCGC-449865/2022|EPI_ISL_11517866|2022-03-16
hCoV-19/Denmark/DCGC-449900/2022|EPI_ISL_11517901|2022-02-04
hCoV-19/Denmark/DCGC-450072/2022|EPI_ISL_11518072|2022-03-16
hCoV-19/Denmark/DCGC-450261/2022|EPI_ISL_11518260|2022-03-16
hCoV-19/Denmark/DCGC-450442/2022|EPI_ISL_11518441|2022-03-16
hCoV-19/Denmark/DCGC-450637/2022|EPI_ISL_11518641|2022-03-16

hCoV-19/Denmark/DCGC-451495/2022|EPI_ISL_11519583|2022-03-16
hCoV-19/Denmark/DCGC-451743/2022|EPI_ISL_11519952|2022-02-03
hCoV-19/Denmark/DCGC-480672/2022|EPI_ISL_12158079|2022-04-12
hCoV-19/Denmark/DCGC-480722/2022|EPI_ISL_12158164|2022-04-12
hCoV-19/Denmark/DCGC-480883/2022|EPI_ISL_12158494|2022-04-12
hCoV-19/Denmark/DCGC-481246/2022|EPI_ISL_12159256|2022-04-12
hCoV-19/Denmark/DCGC-481327/2022|EPI_ISL_12249247|2022-04-12
hCoV-19/Denmark/DCGC-481973/2022|EPI_ISL_12160455|2022-04-12
hCoV-19/Denmark/DCGC-482227/2022|EPI_ISL_12160843|2022-04-13
hCoV-19/Denmark/DCGC-482262/2022|EPI_ISL_12160902|2022-04-13
hCoV-19/Denmark/DCGC-482426/2022|EPI_ISL_12161212|2022-04-12
hCoV-19/Denmark/DCGC-482525/2022|EPI_ISL_12161408|2022-03-02
hCoV-19/Denmark/DCGC-482827/2022|EPI_ISL_12161926|2022-04-12
hCoV-19/Denmark/DCGC-483066/2022|EPI_ISL_12162164|2022-04-12
hCoV-19/Denmark/DCGC-483433/2022|EPI_ISL_12162558|2022-04-12
hCoV-19/Denmark/DCGC-483506/2022|EPI_ISL_12162630|2022-04-13
hCoV-19/Denmark/DCGC-483702/2022|EPI_ISL_12162826|2022-04-13
hCoV-19/Denmark/DCGC-483730/2022|EPI_ISL_12162854|2022-04-13
hCoV-19/Denmark/DCGC-483733/2022|EPI_ISL_12162857|2022-04-13
hCoV-19/Denmark/DCGC-483893/2022|EPI_ISL_12163016|2022-04-12
hCoV-19/Denmark/DCGC-483909/2022|EPI_ISL_12163032|2022-04-12
hCoV-19/Denmark/DCGC-484127/2022|EPI_ISL_12163249|2022-04-12
hCoV-19/Denmark/DCGC-484671/2022|EPI_ISL_12163870|2022-03-02
hCoV-19/Denmark/DCGC-485166/2022|EPI_ISL_12164358|2022-04-12
hCoV-19/Denmark/DCGC-485240/2022|EPI_ISL_12164431|2022-04-13
hCoV-19/Denmark/DCGC-485569/2022|EPI_ISL_12164836|2022-02-01
hCoV-19/Denmark/DCGC-485787/2022|EPI_ISL_12165053|2022-04-13
hCoV-19/Denmark/DCGC-486014/2022|EPI_ISL_12165279|2022-04-12
hCoV-19/Denmark/DCGC-486733/2022|EPI_ISL_12165990|2022-04-12
hCoV-19/Denmark/DCGC-486894/2022|EPI_ISL_12166149|2022-04-13
hCoV-19/Denmark/DCGC-487011/2022|EPI_ISL_12166265|2022-04-12
hCoV-19/Denmark/DCGC-488124/2022|EPI_ISL_12207708|2022-04-13
hCoV-19/Denmark/DCGC-488438/2022|EPI_ISL_12208018|2022-04-13
hCoV-19/Denmark/DCGC-488480/2022|EPI_ISL_12243955|2022-04-13
hCoV-19/Denmark/DCGC-489000/2022|EPI_ISL_12244472|2022-04-12
hCoV-19/Denmark/DCGC-489018/2022|EPI_ISL_12244491|2022-04-13
hCoV-19/Denmark/DCGC-489059/2022|EPI_ISL_12244532|2022-04-12
hCoV-19/Denmark/DCGC-489409/2022|EPI_ISL_12244880|2022-04-13
hCoV-19/Denmark/DCGC-489460/2022|EPI_ISL_12244931|2022-04-13
hCoV-19/Denmark/DCGC-490258/2022|EPI_ISL_12245725|2022-04-12
hCoV-19/Denmark/DCGC-490908/2022|EPI_ISL_12246373|2022-04-13
hCoV-19/Denmark/DCGC-490931/2022|EPI_ISL_12246396|2022-04-13
hCoV-19/Denmark/DCGC-491137/2022|EPI_ISL_12246600|2022-04-12

hCoV-19/Denmark/DCGC-491246/2022|EPI_ISL_12246710|2022-04-13
hCoV-19/Denmark/DCGC-491436/2022|EPI_ISL_12246898|2022-04-13
hCoV-19/Denmark/DCGC-491748/2022|EPI_ISL_12247208|2022-04-13
hCoV-19/Denmark/DCGC-491799/2022|EPI_ISL_12247258|2022-04-13
hCoV-19/Denmark/DCGC-491824/2022|EPI_ISL_12247283|2022-04-13
hCoV-19/Denmark/DCGC-492081/2022|EPI_ISL_12247537|2022-04-13
hCoV-19/Denmark/DCGC-492123/2022|EPI_ISL_12247579|2022-04-13
hCoV-19/Denmark/DCGC-492177/2022|EPI_ISL_12247633|2022-04-13
hCoV-19/Denmark/DCGC-492422/2022|EPI_ISL_12247875|2022-04-13
hCoV-19/Denmark/DCGC-492663/2022|EPI_ISL_12248115|2022-04-13
hCoV-19/Denmark/DCGC-492810/2022|EPI_ISL_12248261|2022-04-12
hCoV-19/Denmark/DCGC-493139/2022|EPI_ISL_12248587|2022-04-12
hCoV-19/Denmark/DCGC-493334/2022|EPI_ISL_12285778|2022-04-12
hCoV-19/Denmark/DCGC-493615/2022|EPI_ISL_12286671|2022-04-12
hCoV-19/Denmark/DCGC-493653/2022|EPI_ISL_12286748|2022-04-12
hCoV-19/Denmark/DCGC-493863/2022|EPI_ISL_12287012|2022-04-12
hCoV-19/Denmark/DCGC-494356/2022|EPI_ISL_12288128|2022-04-12
hCoV-19/Denmark/DCGC-494551/2022|EPI_ISL_12288725|2022-04-12
hCoV-19/Denmark/DCGC-494772/2022|EPI_ISL_12289112|2022-04-12
hCoV-19/Denmark/DCGC-494960/2022|EPI_ISL_12289588|2022-04-12
hCoV-19/Denmark/DCGC-495034/2022|EPI_ISL_12290224|2022-04-12
hCoV-19/Denmark/DCGC-495208/2022|EPI_ISL_12290458|2022-04-12
hCoV-19/Denmark/DCGC-495522/2022|EPI_ISL_12291574|2022-04-12
hCoV-19/Denmark/DCGC-495784/2022|EPI_ISL_12291836|2022-04-12
hCoV-19/Denmark/DCGC-495892/2022|EPI_ISL_12291944|2022-04-12
hCoV-19/Denmark/DCGC-506085/2022|EPI_ISL_12533955|2022-04-13
hCoV-19/Denmark/DCGC-506734/2022|EPI_ISL_12556839|2022-05-01
hCoV-19/Denmark/DCGC-507144/2022|EPI_ISL_12557667|2022-05-01
hCoV-19/Denmark/DCGC-507152/2022|EPI_ISL_12557675|2022-05-01
hCoV-19/Denmark/DCGC-507303/2022|EPI_ISL_12557973|2022-05-01
hCoV-19/Denmark/DCGC-508284/2022|EPI_ISL_12578982|2022-05-01
hCoV-19/Denmark/DCGC-508337/2022|EPI_ISL_12579035|2022-05-02
hCoV-19/Denmark/DCGC-508402/2022|EPI_ISL_12579100|2022-05-02
hCoV-19/Denmark/DCGC-508857/2022|EPI_ISL_12579547|2022-04-12
hCoV-19/Denmark/DCGC-508890/2022|EPI_ISL_12579580|2022-05-01
hCoV-19/Denmark/DCGC-508991/2022|EPI_ISL_12608003|2022-05-02
hCoV-19/Denmark/DCGC-509233/2022|EPI_ISL_12608244|2022-05-02
hCoV-19/Denmark/DCGC-509257/2022|EPI_ISL_12608268|2022-05-03
hCoV-19/Denmark/DCGC-509339/2022|EPI_ISL_12608349|2022-05-03
hCoV-19/Denmark/DCGC-509440/2022|EPI_ISL_12609222|2022-05-03
hCoV-19/Denmark/DCGC-509485/2022|EPI_ISL_12609267|2022-05-02
hCoV-19/Denmark/DCGC-509511/2022|EPI_ISL_12609293|2022-05-02
hCoV-19/Denmark/DCGC-509881/2022|EPI_ISL_12609746|2022-05-03

hCoV-19/Denmark/DCGC-509903/2022|EPI_ISL_12609942|2022-05-03
hCoV-19/Denmark/DCGC-509957/2022|EPI_ISL_12609996|2022-05-03
hCoV-19/Denmark/DCGC-510005/2022|EPI_ISL_12610041|2022-05-02
hCoV-19/Denmark/DCGC-510155/2022|EPI_ISL_12610295|2022-05-02
hCoV-19/Denmark/DCGC-510175/2022|EPI_ISL_12610316|2022-05-03
hCoV-19/Denmark/DCGC-511035/2022|EPI_ISL_12648353|2022-05-03
hCoV-19/Denmark/DCGC-511303/2022|EPI_ISL_12648639|2022-05-03
hCoV-19/Denmark/DCGC-511777/2022|EPI_ISL_12649108|2022-05-03
hCoV-19/Denmark/DCGC-511815/2022|EPI_ISL_12649146|2022-05-03
hCoV-19/Denmark/DCGC-514605/2022|EPI_ISL_12750705|2022-02-16
hCoV-19/Denmark/DCGC-514801/2022|EPI_ISL_12750896|2022-02-16
hCoV-19/Denmark/DCGC-514830/2022|EPI_ISL_12750924|2022-02-03
hCoV-19/Denmark/DCGC-518553/2022|EPI_ISL_12895841|2022-02-16
hCoV-19/Denmark/DCGC-520207/2022|EPI_ISL_12994380|2022-02-02
hCoV-19/Denmark/DCGC-521659/2022|EPI_ISL_13049243|2022-05-26
hCoV-19/Denmark/DCGC-521808/2022|EPI_ISL_13049388|2022-05-25
hCoV-19/Denmark/DCGC-521901/2022|EPI_ISL_13049480|2022-05-27
hCoV-19/Denmark/DCGC-521942/2022|EPI_ISL_13049521|2022-05-25
hCoV-19/Denmark/DCGC-522018/2022|EPI_ISL_13049594|2022-02-02
hCoV-19/Denmark/DCGC-522095/2022|EPI_ISL_13049670|2022-05-25
hCoV-19/Denmark/DCGC-522166/2022|EPI_ISL_13049739|2022-05-25
hCoV-19/Denmark/DCGC-522687/2022|EPI_ISL_13066904|2022-05-26
hCoV-19/Denmark/DCGC-522882/2022|EPI_ISL_13067098|2022-05-26
hCoV-19/Denmark/DCGC-522924/2022|EPI_ISL_13067139|2022-05-27
hCoV-19/Denmark/DCGC-523209/2022|EPI_ISL_13067424|2022-05-27
hCoV-19/Denmark/DCGC-523300/2022|EPI_ISL_13067515|2022-05-27
hCoV-19/Denmark/DCGC-523396/2022|EPI_ISL_13067611|2022-05-25
hCoV-19/Denmark/DCGC-523448/2022|EPI_ISL_13067663|2022-05-27
hCoV-19/Denmark/DCGC-523537/2022|EPI_ISL_13067751|2022-05-27
hCoV-19/Denmark/DCGC-523538/2022|EPI_ISL_13067752|2022-05-27
hCoV-19/Denmark/DCGC-523708/2022|EPI_ISL_13067922|2022-05-28
hCoV-19/Denmark/DCGC-523721/2022|EPI_ISL_13067934|2022-05-26
hCoV-19/Denmark/DCGC-523748/2022|EPI_ISL_13067960|2022-05-25
hCoV-19/Denmark/DCGC-523848/2022|EPI_ISL_13068060|2022-05-25
hCoV-19/Denmark/DCGC-523863/2022|EPI_ISL_13068075|2022-05-26
hCoV-19/Denmark/DCGC-523894/2022|EPI_ISL_13068106|2022-05-27
hCoV-19/Denmark/DCGC-523895/2022|EPI_ISL_13068107|2022-05-28
hCoV-19/Denmark/DCGC-523937/2022|EPI_ISL_13068149|2022-05-25
hCoV-19/Denmark/DCGC-524022/2022|EPI_ISL_13068234|2022-05-28
hCoV-19/Denmark/DCGC-524112/2022|EPI_ISL_13068323|2022-05-26
hCoV-19/Denmark/DCGC-524173/2022|EPI_ISL_13068384|2022-05-26
hCoV-19/Denmark/DCGC-524403/2022|EPI_ISL_13090526|2022-05-29
hCoV-19/Denmark/DCGC-524406/2022|EPI_ISL_13090529|2022-05-29

hCoV-19/Denmark/DCGC-524566/2022|EPI_ISL_13127661|2022-05-29
hCoV-19/Denmark/DCGC-524667/2022|EPI_ISL_13127762|2022-05-28
hCoV-19/Denmark/DCGC-524707/2022|EPI_ISL_13127802|2022-05-28
hCoV-19/Denmark/DCGC-524723/2022|EPI_ISL_13127818|2022-05-28
hCoV-19/Denmark/DCGC-524839/2022|EPI_ISL_13127934|2022-05-29
hCoV-19/Denmark/DCGC-524877/2022|EPI_ISL_13127972|2022-05-29
hCoV-19/Denmark/DCGC-526146/2022|EPI_ISL_13178869|2022-05-29
hCoV-19/Denmark/DCGC-527814/2022|EPI_ISL_13299055|2022-06-08
hCoV-19/Denmark/DCGC-528018/2022|EPI_ISL_13299286|2022-06-08
hCoV-19/Denmark/DCGC-528246/2022|EPI_ISL_13299512|2022-06-08
hCoV-19/Denmark/DCGC-528488/2022|EPI_ISL_13299752|2022-06-09
hCoV-19/Denmark/DCGC-528659/2022|EPI_ISL_13299922|2022-06-08
hCoV-19/Denmark/DCGC-528740/2022|EPI_ISL_13300003|2022-06-08
hCoV-19/Denmark/DCGC-529011/2022|EPI_ISL_13326947|2022-06-09
hCoV-19/Denmark/DCGC-529022/2022|EPI_ISL_13326958|2022-06-11
hCoV-19/Denmark/DCGC-529057/2022|EPI_ISL_13326995|2022-06-11
hCoV-19/Denmark/DCGC-529127/2022|EPI_ISL_13327065|2022-06-09
hCoV-19/Denmark/DCGC-529147/2022|EPI_ISL_13327085|2022-06-08
hCoV-19/Denmark/DCGC-529148/2022|EPI_ISL_13327086|2022-06-11
hCoV-19/Denmark/DCGC-529211/2022|EPI_ISL_13327149|2022-06-09
hCoV-19/Denmark/DCGC-529239/2022|EPI_ISL_13327177|2022-06-09
hCoV-19/Denmark/DCGC-529252/2022|EPI_ISL_13327190|2022-06-11
hCoV-19/Denmark/DCGC-529316/2022|EPI_ISL_13327702|2022-06-09
hCoV-19/Denmark/DCGC-529395/2022|EPI_ISL_13327782|2022-06-11
hCoV-19/Denmark/DCGC-529412/2022|EPI_ISL_13327799|2022-06-09
hCoV-19/Denmark/DCGC-529543/2022|EPI_ISL_13327931|2022-06-09
hCoV-19/Denmark/DCGC-529912/2022|EPI_ISL_13341814|2022-06-12
hCoV-19/Denmark/DCGC-530083/2022|EPI_ISL_13341985|2022-06-12
hCoV-19/Denmark/DCGC-530088/2022|EPI_ISL_13341990|2022-06-12
hCoV-19/Denmark/DCGC-530200/2022|EPI_ISL_13342285|2022-06-11
hCoV-19/Denmark/DCGC-530658/2022|EPI_ISL_13357618|2022-06-08
hCoV-19/Denmark/DCGC-537460/2022|EPI_ISL_13518702|2022-05-25
hCoV-19/Denmark/DCGC-544307/2022|EPI_ISL_13737228|2022-05-27
hCoV-19/Denmark/DCGC-551281/2022|EPI_ISL_14011647|2022-07-14
hCoV-19/Denmark/DCGC-559396/2022|EPI_ISL_14353348|2022-08-02
hCoV-19/Denmark/DCGC-563088/2022|EPI_ISL_14471805|2022-07-14
hCoV-19/Denmark/DCGC-564117/2022|EPI_ISL_14472827|2022-08-09
hCoV-19/Denmark/DCGC-567035/2022|EPI_ISL_14562959|2022-08-12
hCoV-19/Denmark/DCGC-569004/2022|EPI_ISL_14690579|2022-08-19
hCoV-19/Denmark/DCGC-569506/2022|EPI_ISL_14691075|2022-08-15
hCoV-19/Denmark/DCGC-569761/2022|EPI_ISL_14691328|2022-08-17
hCoV-19/Denmark/DCGC-570909/2022|EPI_ISL_14750773|2022-08-26
hCoV-19/Denmark/DCGC-571978/2022|EPI_ISL_14787399|2022-08-28

hCoV-19/Denmark/DCGC-574740/2022|EPI_ISL_14884028|2022-09-01
hCoV-19/Denmark/DCGC-575111/2022|EPI_ISL_14884400|2022-09-02
hCoV-19/Denmark/DCGC-575596/2022|EPI_ISL_14884887|2022-08-30
hCoV-19/Denmark/DCGC-576474/2022|EPI_ISL_14917238|2022-09-05
hCoV-19/Denmark/DCGC-584752/2022|EPI_ISL_15155136|2022-09-21
hCoV-19/Denmark/DCGC-584753/2022|EPI_ISL_15155137|2022-09-21
hCoV-19/Denmark/DCGC-584953/2022|EPI_ISL_15155337|2022-09-21
hCoV-19/Denmark/DCGC-585379/2022|EPI_ISL_15155761|2022-09-22
hCoV-19/Denmark/DCGC-585842/2022|EPI_ISL_15206532|2022-09-22
hCoV-19/Denmark/DCGC-586268/2022|EPI_ISL_15207123|2022-09-24
hCoV-19/Denmark/DCGC-586719/2022|EPI_ISL_15207669|2022-08-31
hCoV-19/Denmark/DCGC-586852/2022|EPI_ISL_15207802|2022-09-24
hCoV-19/Denmark/DCGC-586970/2022|EPI_ISL_15207919|2022-09-22
hCoV-19/Denmark/DCGC-586971/2022|EPI_ISL_15207920|2022-09-24
hCoV-19/Denmark/DCGC-587000/2022|EPI_ISL_15207949|2022-09-21
hCoV-19/Denmark/DCGC-587101/2022|EPI_ISL_15208050|2022-09-22
hCoV-19/Denmark/DCGC-587291/2022|EPI_ISL_15208239|2022-09-21
hCoV-19/Denmark/DCGC-587362/2022|EPI_ISL_15208310|2022-09-24
hCoV-19/Denmark/DCGC-587384/2022|EPI_ISL_15208332|2022-09-22
hCoV-19/Denmark/DCGC-587583/2022|EPI_ISL_15208531|2022-09-22
hCoV-19/Denmark/DCGC-587649/2022|EPI_ISL_15208597|2022-09-24
hCoV-19/Denmark/DCGC-587679/2022|EPI_ISL_15208627|2022-09-22
hCoV-19/Denmark/DCGC-588660/2022|EPI_ISL_15267223|2022-09-28
hCoV-19/Denmark/DCGC-590344/2022|EPI_ISL_15284210|2022-09-29
hCoV-19/Denmark/DCGC-594268/2022|EPI_ISL_15405711|2022-10-12
hCoV-19/Denmark/DCGC-596246/2022|EPI_ISL_15430582|2022-10-13
hCoV-19/Denmark/DCGC-596756/2022|EPI_ISL_15431091|2022-09-29
hCoV-19/Denmark/DCGC-596813/2022|EPI_ISL_15431148|2022-09-28
hCoV-19/Denmark/DCGC-597261/2022|EPI_ISL_15431593|2022-09-29
hCoV-19/Denmark/DCGC-597288/2022|EPI_ISL_15431620|2022-10-12
hCoV-19/Denmark/DCGC-597440/2022|EPI_ISL_15431771|2022-10-12
hCoV-19/Denmark/DCGC-598546/2022|EPI_ISL_15467251|2022-10-17
hCoV-19/Denmark/DCGC-600703/2022|EPI_ISL_15551856|2022-10-18
hCoV-19/Denmark/DCGC-605762/2022|EPI_ISL_15651248|2022-10-28
hCoV-19/Denmark/DCGC-606738/2022|EPI_ISL_15697748|2022-10-31
hCoV-19/Denmark/DCGC-616363/2022|EPI_ISL_15971573|2022-11-25
hCoV-19/Denmark/DCGC-616526/2022|EPI_ISL_15971761|2022-11-26
hCoV-19/Denmark/DCGC-616558/2022|EPI_ISL_15971793|2022-11-24
hCoV-19/Denmark/DCGC-617374/2022|EPI_ISL_15984076|2022-11-27
hCoV-19/Denmark/DCGC-617486/2022|EPI_ISL_15984188|2022-11-27
hCoV-19/Denmark/DCGC-617608/2022|EPI_ISL_15984309|2022-11-27
hCoV-19/Denmark/DCGC-617609/2022|EPI_ISL_15984310|2022-11-27
hCoV-19/Denmark/DCGC-620054/2022|EPI_ISL_16062340|2022-11-30

hCoV-19/Denmark/DCGC-620059/2022|EPI_ISL_16062345|2022-11-30
hCoV-19/Denmark/DCGC-620061/2022|EPI_ISL_16062347|2022-11-29
hCoV-19/Denmark/DCGC-620094/2022|EPI_ISL_16062380|2022-11-29
hCoV-19/Denmark/DCGC-620135/2022|EPI_ISL_16062421|2022-11-30
hCoV-19/Denmark/DCGC-620144/2022|EPI_ISL_16062430|2022-11-29
hCoV-19/Denmark/DCGC-620935/2022|EPI_ISL_16063216|2022-11-30
hCoV-19/Denmark/DCGC-620952/2022|EPI_ISL_16063233|2022-11-30
hCoV-19/Denmark/DCGC-620960/2022|EPI_ISL_16063241|2022-11-29
hCoV-19/Denmark/DCGC-620969/2022|EPI_ISL_16063250|2022-11-30
hCoV-19/Denmark/DCGC-620974/2022|EPI_ISL_16063255|2022-11-28
hCoV-19/Denmark/DCGC-621287/2022|EPI_ISL_16063567|2022-11-30
hCoV-19/Denmark/DCGC-621306/2022|EPI_ISL_16063586|2022-11-30
hCoV-19/Denmark/DCGC-621527/2022|EPI_ISL_16063807|2022-11-29
hCoV-19/Denmark/DCGC-621580/2022|EPI_ISL_16063859|2022-11-29
hCoV-19/Denmark/DCGC-621581/2022|EPI_ISL_16063860|2022-11-29
hCoV-19/Denmark/DCGC-621606/2022|EPI_ISL_16063885|2022-11-29
hCoV-19/DRC/2536/2020|EPI_ISL_437339|2020-04-15
hCoV-19/Ecuador/USFQ-105/2020|EPI_ISL_486842|2020-07-01
hCoV-19/Egypt/CPHL-EGY22595/2022|EPI_ISL_15670116|2022-08-29
hCoV-19/Egypt/CUNCI-HGC6I007/2020|EPI_ISL_479722|2020-06-02
hCoV-19/El_Salvador/INC-LNSP-258/2022|EPI_ISL_15185664|2022-07-15
hCoV-19/El_Salvador/INC-LNSP-300/2022|EPI_ISL_15185706|2022-07-18
hCoV-19/England/203690368/2020|EPI_ISL_534729|2020-09-05
hCoV-19/England/ALDP-9BE457/2020|EPI_ISL_567916|2020-09-10
hCoV-19/England/CAMB-785C8/2020|EPI_ISL_433753|2020-04-05
hCoV-19/England/CAMB-81535/2020|EPI_ISL_443104|2020-04-11
hCoV-19/England/CAMB-81F82/2020|EPI_ISL_438600|2020-04-21
hCoV-19/England/CAMC-B78BCB/2020|EPI_ISL_783376|2020-11-09
hCoV-19/England/LIVE-A0248/2020|EPI_ISL_472097|2020-06-03
hCoV-19/England/LSPA-32606760/2022|EPI_ISL_16071729|2022-11-29
hCoV-19/England/LSPA-3260688B/2022|EPI_ISL_16071741|2022-11-29
hCoV-19/England/LSPA-32606988/2022|EPI_ISL_16071721|2022-11-26
hCoV-19/England/LSPA-326069E2/2022|EPI_ISL_16071725|2022-11-29
hCoV-19/England/LSPA-32606AA3/2022|EPI_ISL_16071719|2022-11-29
hCoV-19/England/LSPA-32607D4F/2022|EPI_ISL_16072145|2022-11-30
hCoV-19/England/LSPA-3760B1C/2022|EPI_ISL_10550906|2022-02-17
hCoV-19/England/MILK-978A93/2020|EPI_ISL_550468|2020-08-01
hCoV-19/England/MILK-9A85C6/2020|EPI_ISL_550189|2020-09-02
hCoV-19/England/NOTT-119DB1/2020|EPI_ISL_741609|2020-11-03
hCoV-19/England/PHEC-1AFF7/2020|EPI_ISL_2737700|2020-02-13
hCoV-19/England/PHEC-1B22A/2020|EPI_ISL_2737734|2020-02-29
hCoV-19/England/PHEC-5X06FZ91/2022|EPI_ISL_11155578|2022-02-02
hCoV-19/England/PHEC-YY8EKUA/2022|EPI_ISL_15579918|2022-10-18

hCoV-19/England/PHEC-YY8EPUI/2022|EPI_ISL_15580010|2022-10-18
hCoV-19/England/PHEC-YY8GU95/2022|EPI_ISL_15580685|2022-10-13
hCoV-19/England/PHEC-YY8J8RA/2022|EPI_ISL_15630661|2022-10-18
hCoV-19/England/PHEC-YY8M7U9/2022|EPI_ISL_15760730|2022-10-31
hCoV-19/England/PHEC-YY8MZGY/2022|EPI_ISL_15760858|2022-10-28
hCoV-19/England/PHEC-YY8OC7T/2022|EPI_ISL_16109012|2022-11-29
hCoV-19/England/PHEC-YY8OCA5/2022|EPI_ISL_16109014|2022-11-29
hCoV-19/England/PHEC-YY8OCHU/2022|EPI_ISL_16109018|2022-11-29
hCoV-19/England/PHEC-YY8OCJ4/2022|EPI_ISL_16109020|2022-11-28
hCoV-19/England/PHEC-YY8OCNE/2022|EPI_ISL_16109022|2022-11-28
hCoV-19/England/PHEC-YY8ORWM/2022|EPI_ISL_16075056|2022-11-28
hCoV-19/England/PHEC-YY8ORYW/2022|EPI_ISL_16075058|2022-11-25
hCoV-19/England/PHEC-YY8OY4D/2022|EPI_ISL_16075062|2022-11-29
hCoV-19/England/PHEC-YY8OYA8/2022|EPI_ISL_16075066|2022-11-25
hCoV-19/England/PHEC-YY8OYH9/2022|EPI_ISL_16075071|2022-11-27
hCoV-19/England/PHEC-YY8QIJS/2022|EPI_ISL_16005129|2022-11-17
hCoV-19/England/PHEC-YY8QIKW/2022|EPI_ISL_16005130|2022-11-21
hCoV-19/England/PHEC-YY8QIM1/2022|EPI_ISL_16005131|2022-11-18
hCoV-19/England/PHEC-YY8QIQC/2022|EPI_ISL_16005134|2022-11-18
hCoV-19/England/PHEC-YY8QS6K/2022|EPI_ISL_16005140|2022-11-18
hCoV-19/England/PHEC-YY8QSHQ/2022|EPI_ISL_16005147|2022-11-20
hCoV-19/England/PHEC-YY8QSW6/2022|EPI_ISL_16005153|2022-11-19
hCoV-19/England/PHEC-YY8XJ6I/2022|EPI_ISL_16017550|2022-11-22
hCoV-19/England/PHEC-YY8XJTX/2022|EPI_ISL_16017555|2022-11-20
hCoV-19/England/PHEC-YY8XK7S/2022|EPI_ISL_16017561|2022-11-21
hCoV-19/England/PHEC-YY8XKHA/2022|EPI_ISL_16017568|2022-11-21
hCoV-19/England/PHEC-YY8Y7HD/2022|EPI_ISL_15386156|2022-09-30
hCoV-19/England/PHEC-YY8YAWA/2022|EPI_ISL_15386215|2022-09-28
hCoV-19/England/PHEC-YY8YKE9/2022|EPI_ISL_15386321|2022-09-29
hCoV-19/England/PHEC-YY8YU98/2022|EPI_ISL_15386493|2022-09-30
hCoV-19/England/PHEC-YYD51B1/2022|EPI_ISL_11877293|2022-03-16
hCoV-19/England/PHEC-YYD8H6S/2022|EPI_ISL_11223308|2022-03-02
hCoV-19/England/PHEC-YYDEOEP/2022|EPI_ISL_11224209|2022-03-01
hCoV-19/England/PHEC-YYDGAWO/2022|EPI_ISL_11224726|2022-03-01
hCoV-19/England/PHEC-YYDGOXD/2022|EPI_ISL_11225076|2022-03-02
hCoV-19/England/PHEC-YYDRAWW/2022|EPI_ISL_12581549|2022-03-02
hCoV-19/England/PHEC-YYDU57Y/2022|EPI_ISL_11655811|2022-03-16
hCoV-19/England/PHEC-YYDUI5K/2022|EPI_ISL_11657448|2022-03-16
hCoV-19/England/PHEC-YYDWN8R/2022|EPI_ISL_11658327|2022-03-16
hCoV-19/England/PHEC-YYFGPAT/2022|EPI_ISL_12705312|2022-05-03
hCoV-19/England/PHEC-YYFGPW3/2022|EPI_ISL_12705327|2022-05-02
hCoV-19/England/PHEC-YYFP41N/2022|EPI_ISL_13244023|2022-05-29
hCoV-19/England/PHEC-YYFPGRU/2022|EPI_ISL_13131450|2022-05-25

hCoV-19/England/PHEC-YYFQNUS/2022|EPI_ISL_13244216|2022-05-29
hCoV-19/England/PHEC-YYFTWSZ/2022|EPI_ISL_13475456|2022-06-11
hCoV-19/England/PHEC-YYFX7T7/2022|EPI_ISL_13421463|2022-06-08
hCoV-19/England/PHEC-YYFX9ZX/2022|EPI_ISL_13421520|2022-06-09
hCoV-19/England/PHEC-YYFXWTG/2022|EPI_ISL_13399890|2022-05-25
hCoV-19/England/PHEC-YYFXZTD/2022|EPI_ISL_13399916|2022-06-08
hCoV-19/England/PHEC-YYG1D55/2022|EPI_ISL_14459070|2022-08-03
hCoV-19/England/PHEC-YYG1N7A/2022|EPI_ISL_14459115|2022-07-28
hCoV-19/England/PHEC-YYG1RUK/2022|EPI_ISL_14459159|2022-08-02
hCoV-19/England/PHEC-YYG3CAK/2022|EPI_ISL_14950306|2022-08-29
hCoV-19/England/PHEC-YYG3CSQ/2022|EPI_ISL_14950317|2022-08-28
hCoV-19/England/PHEC-YYG3EIW/2022|EPI_ISL_14919282|2022-08-31
hCoV-19/England/PHEC-YYG3GR3/2022|EPI_ISL_14919335|2022-08-30
hCoV-19/England/PHEC-YYG3I9U/2022|EPI_ISL_14973529|2022-09-01
hCoV-19/England/PHEC-YYG3J7D/2022|EPI_ISL_14919343|2022-08-28
hCoV-19/England/PHEC-YYG3JO7/2022|EPI_ISL_14919355|2022-08-30
hCoV-19/England/PHEC-YYG3MO5/2022|EPI_ISL_14950360|2022-08-30
hCoV-19/England/PHEC-YYG3N5Q/2022|EPI_ISL_14919387|2022-08-28
hCoV-19/England/PHEC-YYG3NZS/2022|EPI_ISL_14919407|2022-08-28
hCoV-19/England/PHEC-YYG3U73/2022|EPI_ISL_14950435|2022-08-31
hCoV-19/England/PHEC-YYG3YTR/2022|EPI_ISL_14919490|2022-08-30
hCoV-19/England/PHEC-YYG48G1/2022|EPI_ISL_15020820|2022-09-04
hCoV-19/England/PHEC-YYG4CNI/2022|EPI_ISL_15020847|2022-09-05
hCoV-19/England/PHEC-YYG4E8X/2022|EPI_ISL_15020885|2022-09-02
hCoV-19/England/PHEC-YYG4G7R/2022|EPI_ISL_15020930|2022-08-31
hCoV-19/England/PHEC-YYG4IJ6/2022|EPI_ISL_15020964|2022-08-30
hCoV-19/England/PHEC-YYG4JNA/2022|EPI_ISL_15020985|2022-08-31
hCoV-19/England/PHEC-YYG4R6R/2022|EPI_ISL_15021131|2022-08-31
hCoV-19/England/PHEC-YYG4T3B/2022|EPI_ISL_15021154|2022-09-05
hCoV-19/England/PHEC-YYG5ISB/2022|EPI_ISL_15138820|2022-09-13
hCoV-19/England/PHEC-YYG5XZF/2022|EPI_ISL_15110405|2022-09-13
hCoV-19/England/PHEC-YYG87GD/2022|EPI_ISL_14171637|2022-07-15
hCoV-19/England/PHEC-YYG8ZM9/2022|EPI_ISL_14172126|2022-07-18
hCoV-19/England/PHEC-YYG9HD3/2022|EPI_ISL_15347999|2022-09-27
hCoV-19/England/PHEC-YYGA13R/2022|EPI_ISL_14858681|2022-08-26
hCoV-19/England/PHEC-YYGA1AG/2022|EPI_ISL_14858686|2022-08-26
hCoV-19/England/PHEC-YYGA1DT/2022|EPI_ISL_14858689|2022-08-26
hCoV-19/England/PHEC-YYGA3HZ/2022|EPI_ISL_14919502|2022-08-26
hCoV-19/England/PHEC-YYGA4GD/2022|EPI_ISL_14919525|2022-08-27
hCoV-19/England/PHEC-YYGA79P/2022|EPI_ISL_14919582|2022-08-28
hCoV-19/England/PHEC-YYGA7QO/2022|EPI_ISL_14919596|2022-08-28
hCoV-19/England/PHEC-YYGA8YN/2022|EPI_ISL_14812214|2022-08-23
hCoV-19/England/PHEC-YYGA9DR/2022|EPI_ISL_14919612|2022-08-27

hCoV-19/England/PHEC-YYGA9NG/2022|EPI_ISL_14919618|2022-08-26
hCoV-19/England/PHEC-YYGAJEO/2022|EPI_ISL_14822682|2022-08-11
hCoV-19/England/PHEC-YYGAKD3/2022|EPI_ISL_14822704|2022-08-23
hCoV-19/England/PHEC-YYGATNW/2022|EPI_ISL_14858786|2022-08-27
hCoV-19/England/PHEC-YYGH9BY/2022|EPI_ISL_15249229|2022-09-22
hCoV-19/England/PHEC-YYGHSIY/2022|EPI_ISL_15212178|2022-09-19
hCoV-19/England/PHEC-YYGI3FC/2022|EPI_ISL_14700103|2022-08-14
hCoV-19/England/PHEC-YYGINE7/2022|EPI_ISL_14651193|2022-08-12
hCoV-19/England/PHEC-YYGIYA6/2022|EPI_ISL_14618049|2022-08-12
hCoV-19/England/PHEC-YYGJ1XA/2022|EPI_ISL_14234478|2022-07-21
hCoV-19/England/PHEC-YYGJN1B/2022|EPI_ISL_14212354|2022-07-13
hCoV-19/England/PHEC-YYGM4JA/2022|EPI_ISL_14300168|2022-07-23
hCoV-19/England/PHEC-YYGMOYW/2022|EPI_ISL_14300609|2022-07-20
hCoV-19/England/PHEC-YYGMXIK/2022|EPI_ISL_14300825|2022-07-20
hCoV-19/England/PHEC-YYGO4C8/2022|EPI_ISL_14431071|2022-07-28
hCoV-19/England/PHEC-YYGPC6Z/2022|EPI_ISL_14301142|2022-07-24
hCoV-19/England/PHEC-YYGR36H/2022|EPI_ISL_14090014|2022-07-14
hCoV-19/England/PHEC-YYGS1TA/2022|EPI_ISL_14700471|2022-08-17
hCoV-19/England/PHEC-YYGSCJX/2022|EPI_ISL_14700602|2022-08-16
hCoV-19/England/PHEC-YYGSNH1/2022|EPI_ISL_14700848|2022-08-15
hCoV-19/England/PHEC-YYGT3N4/2022|EPI_ISL_14459222|2022-07-30
hCoV-19/England/PHEC-YYGTRK9/2022|EPI_ISL_14459721|2022-07-28
hCoV-19/England/PHEC-YYGU1HE/2022|EPI_ISL_14571493|2022-08-05
hCoV-19/England/PHEC-YYGU9M6/2022|EPI_ISL_14571626|2022-08-08
hCoV-19/England/PHEC-YYGUEMI/2022|EPI_ISL_14543345|2022-08-05
hCoV-19/England/PHEC-YYGUJWB/2022|EPI_ISL_14543400|2022-07-31
hCoV-19/England/PHEC-YYGUKUN/2022|EPI_ISL_14543422|2022-08-06
hCoV-19/England/PHEC-YYGUMF6/2022|EPI_ISL_14543437|2022-08-05
hCoV-19/England/PHEC-YYGWH9S/2022|EPI_ISL_14618216|2022-08-10
hCoV-19/England/PHEC-YYGX45M/2022|EPI_ISL_14389665|2022-07-26
hCoV-19/England/PHEC-YYGXSOF/2022|EPI_ISL_14389847|2022-07-26
hCoV-19/England/PHEC-YYGXSR6/2022|EPI_ISL_14324137|2022-07-28
hCoV-19/England/PHEC-YYGZ3JY/2022|EPI_ISL_14812407|2022-08-22
hCoV-19/England/PHEC-YYGZ5PS/2022|EPI_ISL_14812460|2022-08-22
hCoV-19/England/PHEC-YYGZG31/2022|EPI_ISL_14772146|2022-08-17
hCoV-19/England/PHEC-YYGZGAW/2022|EPI_ISL_14772152|2022-08-06
hCoV-19/England/PHEC-YYGZGSA/2022|EPI_ISL_14772163|2022-08-06
hCoV-19/England/PHEC-YYGZH1K/2022|EPI_ISL_14812540|2022-08-23
hCoV-19/England/PHEC-YYGZQAC/2022|EPI_ISL_14786702|2022-08-22
hCoV-19/England/PHEC-YYGZSC7/2022|EPI_ISL_14812619|2022-08-18
hCoV-19/England/PHEC-YYGZUFQ/2022|EPI_ISL_14812670|2022-08-20
hCoV-19/England/PHEC-YYNOCQI/2022|EPI_ISL_10183908|2022-02-02
hCoV-19/England/PHEC-YYRZ3JN/2022|EPI_ISL_12345980|2022-04-12

hCoV-19/England/QEUH-9616D5/2020|EPI_ISL_537112|2020-08-03

hCoV-19/England/QEUH-96E6D8/2020|EPI_ISL_530400|2020-08-17

hCoV-19/Estonia/16028328/2022|EPI_ISL_10798679|2022-02-03

hCoV-19/Estonia/19070821/2022|EPI_ISL_9649788|2022-01-03

hCoV-19/Estonia/RC0548241/2022|EPI_ISL_11739519|2022-02-17

hCoV-19/Estonia/RC0685135/2022|EPI_ISL_12449969|2022-04-12

hCoV-19/Estonia/RC0699688/2022|EPI_ISL_13029431|2022-05-02

hCoV-19/Finland/THL-202202476/2022|EPI_ISL_9672782|2022-01-03

hCoV-19/Finland/THL-202209104/2022|EPI_ISL_11813230|2022-03-01

hCoV-19/Finland/THL-202209486/2022|EPI_ISL_12624890|2022-02-03

hCoV-19/France/ARA-CFD220063859501/2022|EPI_ISL_16064116|2022-11-30

hCoV-19/France/ARA-CFD700000323525/2022|EPI_ISL_16008160|2022-11-16

hCoV-19/France/ARA-CFD700000325231/2022|EPI_ISL_16008161|2022-11-16

hCoV-19/France/ARA-CFD700000325324/2022|EPI_ISL_16008162|2022-11-21

hCoV-19/France/ARA-GENBIO-14293261273/2022|EPI_ISL_15968444|2022-11-22

hCoV-19/France/ARA-HCL022198320401/2022|EPI_ISL_16056100|2022-11-26

hCoV-19/France/ARA-HCL022198608201/2022|EPI_ISL_16056121|2022-11-28

hCoV-19/France/ARA-HCL022198698301/2022|EPI_ISL_16056126|2022-11-21

hCoV-19/France/ARA-HCL722004322301/2022|EPI_ISL_16056220|2022-11-21

hCoV-19/France/ARA-SC723/2020|EPI_ISL_779817|2020-11-15

hCoV-19/France/BFC-BFC-478CL8721031913/2022|EPI_ISL_12248903|2022-04-13

hCoV-19/France/BFC-CHUD-720AN8721591259/2022|EPI_ISL_13514020|2022-06-08

hCoV-19/France/BRE-ChuBr-2205551098/2022|EPI_ISL_13300448|2022-05-25

hCoV-19/France/BRE-IPP56001/2022|EPI_ISL_15973201|2022-11-21

hCoV-19/France/CVL-CERBAHC-01435076/2022|EPI_ISL_9470034|2022-01-03

hCoV-19/France/HDF-IPP47913/2022|EPI_ISL_14439355|2022-08-02

hCoV-19/France/HDF-IPP51214/2022|EPI_ISL_15271195|2022-09-26

hCoV-19/France/IDF-HCL990000378924/2021|EPI_ISL_16467459|2021-06-14

hCoV-19/France/NAQ-CHUP-2202011492/2022|EPI_ISL_11567883|2022-02-01

hCoV-19/France/NAQ-CHUP-2202041078/2022|EPI_ISL_11564530|2022-02-04

hCoV-19/France/NAQ-CHUP-2202050765/2022|EPI_ISL_11564527|2022-02-05

hCoV-19/France/NAQ-CHUP-2203161805/2022|EPI_ISL_12029501|2022-03-17

hCoV-19/France/NAQ-IPP49676/2022|EPI_ISL_14951679|2022-08-29

hCoV-19/France/OCC-ChuMtp-99223101962/2022|EPI_ISL_15969421|2022-11-06

hCoV-19/France/OCC-ChuMtp-99223133867/2022|EPI_ISL_15969438|2022-11-09

hCoV-19/France/PAC-CERBAHC-01436120/2022|EPI_ISL_10314487|2022-01-03

hCoV-19/France/PAC-IHU-12269_Nova1/2020|EPI_ISL_2659910|2020-05-05

hCoV-19/Fujian/BD-VIR-SPE-0782/2021|EPI_ISL_15775813|2021-09-12

hCoV-19/Germany/BB-RKI-I-1040124/2022|EPI_ISL_15990822|2022-11-25

hCoV-19/Germany/BB-RKI-I-1040135/2022|EPI_ISL_15990832|2022-11-26

hCoV-19/Germany/BB-RKI-I-1040147/2022|EPI_ISL_15990842|2022-11-24

hCoV-19/Germany/BB-RKI-I-1040155/2022|EPI_ISL_15990850|2022-11-25

hCoV-19/Germany/BB-RKI-I-1040160/2022|EPI_ISL_15990855|2022-11-25

hCoV-19/Germany/BB-RKI-I-1050402/2021|EPI_ISL_16144611|2021-02-09
hCoV-19/Germany/BB-RKI-I-189283/2021|EPI_ISL_3120722|2021-05-04
hCoV-19/Germany/BB-RKI-I-683171/2022|EPI_ISL_11669323|2022-03-17
hCoV-19/Germany/BB-RKI-I-683774/2022|EPI_ISL_11670769|2022-03-16
hCoV-19/Germany/BB-RKI-I-845140/2022|EPI_ISL_13238841|2022-05-27
hCoV-19/Germany/BB-RKI-I-845147/2022|EPI_ISL_13238847|2022-05-27
hCoV-19/Germany/BB-RKI-I-919745/2022|EPI_ISL_14381353|2022-07-19
hCoV-19/Germany/BE-ChVir-LB-220211-4639/2022|EPI_ISL_9975602|2022-02-02
hCoV-19/Germany/BE-ChVir-LB-220225-8586/2022|EPI_ISL_10707805|2022-02-17
hCoV-19/Germany/BE-RKI-I-787657/2022|EPI_ISL_12677068|2022-05-01
hCoV-19/Germany/BW-RKI-I-026865/2020|EPI_ISL_1157079|2020-12-13
hCoV-19/Germany/BW-RKI-I-1035311/2022|EPI_ISL_15986743|2022-11-17
hCoV-19/Germany/BW-RKI-I-1035313/2022|EPI_ISL_15986745|2022-11-17
hCoV-19/Germany/BW-RKI-I-1035314/2022|EPI_ISL_15986746|2022-11-17
hCoV-19/Germany/BW-RKI-I-1035316/2022|EPI_ISL_15986748|2022-11-17
hCoV-19/Germany/BW-RKI-I-1035318/2022|EPI_ISL_15986750|2022-11-17
hCoV-19/Germany/BW-RKI-I-1035319/2022|EPI_ISL_15986751|2022-11-17
hCoV-19/Germany/BW-RKI-I-1035320/2022|EPI_ISL_15986752|2022-11-17
hCoV-19/Germany/BW-RKI-I-1040173/2022|EPI_ISL_15990868|2022-11-24
hCoV-19/Germany/BW-RKI-I-1043707/2022|EPI_ISL_16042724|2022-11-29
hCoV-19/Germany/BW-RKI-I-1043740/2022|EPI_ISL_16042750|2022-11-28
hCoV-19/Germany/BW-RKI-I-1043978/2022|EPI_ISL_16042978|2022-11-24
hCoV-19/Germany/BW-RKI-I-527240/2022|EPI_ISL_10019569|2022-02-03
hCoV-19/Germany/BW-RKI-I-527376/2022|EPI_ISL_10020193|2022-02-04
hCoV-19/Germany/BW-RKI-I-561776/2022|EPI_ISL_10299577|2022-02-16
hCoV-19/Germany/BW-RKI-I-572753/2022|EPI_ISL_10741293|2022-02-16
hCoV-19/Germany/BW-RKI-I-580062/2022|EPI_ISL_10756255|2022-02-03
hCoV-19/Germany/BW-RKI-I-580828/2022|EPI_ISL_10757596|2022-02-03
hCoV-19/Germany/BW-RKI-I-580930/2022|EPI_ISL_10757733|2022-02-03
hCoV-19/Germany/BW-RKI-I-581004/2022|EPI_ISL_10758199|2022-02-03
hCoV-19/Germany/BW-RKI-I-581162/2022|EPI_ISL_10758775|2022-02-03
hCoV-19/Germany/BW-RKI-I-581481/2022|EPI_ISL_10760410|2022-02-03
hCoV-19/Germany/BW-RKI-I-602201/2022|EPI_ISL_10917780|2022-03-01
hCoV-19/Germany/BW-RKI-I-602317/2022|EPI_ISL_10917850|2022-03-01
hCoV-19/Germany/BW-RKI-I-602364/2022|EPI_ISL_10917881|2022-03-01
hCoV-19/Germany/BW-RKI-I-602491/2022|EPI_ISL_10917959|2022-03-02
hCoV-19/Germany/BW-RKI-I-602519/2022|EPI_ISL_10917979|2022-03-02
hCoV-19/Germany/BW-RKI-I-602778/2022|EPI_ISL_10918137|2022-03-02
hCoV-19/Germany/BW-RKI-I-602972/2022|EPI_ISL_10918268|2022-03-01
hCoV-19/Germany/BW-RKI-I-603004/2022|EPI_ISL_10918290|2022-03-01
hCoV-19/Germany/BW-RKI-I-603242/2022|EPI_ISL_10918447|2022-03-01
hCoV-19/Germany/BW-RKI-I-606140/2022|EPI_ISL_10920536|2022-03-02
hCoV-19/Germany/BW-RKI-I-611107/2022|EPI_ISL_11452677|2022-02-17

hCoV-19/Germany/BW-RKI-I-611139/2022|EPI_ISL_11452709|2022-02-17
hCoV-19/Germany/BW-RKI-I-611622/2022|EPI_ISL_11453245|2022-02-17
hCoV-19/Germany/BW-RKI-I-615447/2022|EPI_ISL_11456533|2022-02-17
hCoV-19/Germany/BW-RKI-I-615503/2022|EPI_ISL_11456636|2022-02-17
hCoV-19/Germany/BW-RKI-I-615535/2022|EPI_ISL_11456694|2022-02-17
hCoV-19/Germany/BW-RKI-I-615579/2022|EPI_ISL_11456762|2022-02-17
hCoV-19/Germany/BW-RKI-I-615800/2022|EPI_ISL_11456998|2022-02-17
hCoV-19/Germany/BW-RKI-I-616004/2022|EPI_ISL_11457297|2022-02-17
hCoV-19/Germany/BW-RKI-I-616108/2022|EPI_ISL_11457599|2022-02-17
hCoV-19/Germany/BW-RKI-I-616119/2022|EPI_ISL_11457612|2022-02-17
hCoV-19/Germany/BW-RKI-I-616215/2022|EPI_ISL_11457722|2022-02-17
hCoV-19/Germany/BW-RKI-I-616282/2022|EPI_ISL_11457794|2022-02-17
hCoV-19/Germany/BW-RKI-I-616451/2022|EPI_ISL_11457991|2022-02-17
hCoV-19/Germany/BW-RKI-I-621437/2022|EPI_ISL_11464198|2022-03-01
hCoV-19/Germany/BW-RKI-I-637337/2022|EPI_ISL_11480864|2022-02-17
hCoV-19/Germany/BW-RKI-I-665456/2022|EPI_ISL_11637838|2022-03-16
hCoV-19/Germany/BW-RKI-I-683444/2022|EPI_ISL_11670429|2022-03-16
hCoV-19/Germany/BW-RKI-I-685089/2022|EPI_ISL_11672580|2022-03-16
hCoV-19/Germany/BW-RKI-I-687463/2022|EPI_ISL_11675772|2022-03-17
hCoV-19/Germany/BW-RKI-I-687467/2022|EPI_ISL_11675776|2022-03-17
hCoV-19/Germany/BW-RKI-I-712573/2022|EPI_ISL_12025352|2022-03-17
hCoV-19/Germany/BW-RKI-I-712646/2022|EPI_ISL_12025431|2022-03-17
hCoV-19/Germany/BW-RKI-I-712699/2022|EPI_ISL_12025482|2022-03-17
hCoV-19/Germany/BW-RKI-I-712707/2022|EPI_ISL_12025490|2022-03-17
hCoV-19/Germany/BW-RKI-I-712710/2022|EPI_ISL_12025493|2022-03-17
hCoV-19/Germany/BW-RKI-I-712807/2022|EPI_ISL_12025659|2022-03-17
hCoV-19/Germany/BW-RKI-I-739503/2022|EPI_ISL_12346111|2022-04-12
hCoV-19/Germany/BW-RKI-I-740957/2022|EPI_ISL_12349173|2022-04-13
hCoV-19/Germany/BW-RKI-I-741048/2022|EPI_ISL_12349383|2022-04-13
hCoV-19/Germany/BW-RKI-I-745269/2022|EPI_ISL_12355348|2022-04-13
hCoV-19/Germany/BW-RKI-I-745385/2022|EPI_ISL_12355482|2022-04-12
hCoV-19/Germany/BW-RKI-I-745431/2022|EPI_ISL_12355536|2022-04-12
hCoV-19/Germany/BW-RKI-I-746338/2022|EPI_ISL_12356957|2022-04-13
hCoV-19/Germany/BW-RKI-I-747828/2022|EPI_ISL_12358328|2022-04-13
hCoV-19/Germany/BW-RKI-I-748078/2022|EPI_ISL_12358591|2022-04-12
hCoV-19/Germany/BW-RKI-I-748168/2022|EPI_ISL_12358676|2022-04-13
hCoV-19/Germany/BW-RKI-I-750548/2022|EPI_ISL_12362277|2022-04-12
hCoV-19/Germany/BW-RKI-I-750658/2022|EPI_ISL_12362406|2022-04-12
hCoV-19/Germany/BW-RKI-I-750729/2022|EPI_ISL_12362510|2022-04-12
hCoV-19/Germany/BW-RKI-I-751027/2022|EPI_ISL_12362591|2022-04-12
hCoV-19/Germany/BW-RKI-I-755804/2022|EPI_ISL_12367152|2022-04-13
hCoV-19/Germany/BW-RKI-I-777142/2022|EPI_ISL_12667885|2022-05-02
hCoV-19/Germany/BW-RKI-I-784889/2022|EPI_ISL_12674713|2022-05-02

hCoV-19/Germany/BW-RKI-I-785318/2022|EPI_ISL_12675067|2022-05-02
hCoV-19/Germany/BW-RKI-I-785345/2022|EPI_ISL_12675091|2022-05-03
hCoV-19/Germany/BW-RKI-I-785975/2022|EPI_ISL_12675506|2022-05-03
hCoV-19/Germany/BW-RKI-I-786044/2022|EPI_ISL_12675572|2022-05-02
hCoV-19/Germany/BW-RKI-I-786268/2022|EPI_ISL_12675789|2022-05-02
hCoV-19/Germany/BW-RKI-I-786358/2022|EPI_ISL_12675876|2022-05-02
hCoV-19/Germany/BW-RKI-I-786519/2022|EPI_ISL_12676030|2022-05-02
hCoV-19/Germany/BW-RKI-I-789533/2022|EPI_ISL_12678284|2022-05-03
hCoV-19/Germany/BW-RKI-I-790642/2022|EPI_ISL_12729101|2022-05-03
hCoV-19/Germany/BW-RKI-I-800527/2022|EPI_ISL_12813375|2022-05-03
hCoV-19/Germany/BW-RKI-I-808783/2022|EPI_ISL_12820079|2022-05-03
hCoV-19/Germany/BW-RKI-I-809196/2022|EPI_ISL_12820472|2022-05-03
hCoV-19/Germany/BW-RKI-I-809258/2022|EPI_ISL_12820532|2022-05-02
hCoV-19/Germany/BW-RKI-I-809300/2022|EPI_ISL_12820571|2022-05-02
hCoV-19/Germany/BW-RKI-I-825559/2022|EPI_ISL_13003816|2022-05-02
hCoV-19/Germany/BW-RKI-I-825972/2022|EPI_ISL_13004214|2022-05-03
hCoV-19/Germany/BW-RKI-I-842138/2022|EPI_ISL_13235852|2022-05-27
hCoV-19/Germany/BW-RKI-I-848627/2022|EPI_ISL_13241545|2022-05-26
hCoV-19/Germany/BW-RKI-I-849165/2022|EPI_ISL_13244609|2022-05-27
hCoV-19/Germany/BW-RKI-I-849172/2022|EPI_ISL_13244617|2022-05-29
hCoV-19/Germany/BW-RKI-I-849180/2022|EPI_ISL_13244625|2022-05-28
hCoV-19/Germany/BW-RKI-I-849370/2022|EPI_ISL_13244830|2022-05-28
hCoV-19/Germany/BW-RKI-I-851321/2022|EPI_ISL_13246293|2022-05-29
hCoV-19/Germany/BW-RKI-I-852888/2022|EPI_ISL_13247322|2022-05-25
hCoV-19/Germany/BW-RKI-I-853153/2022|EPI_ISL_13247577|2022-05-28
hCoV-19/Germany/BW-RKI-I-853194/2022|EPI_ISL_13247616|2022-05-28
hCoV-19/Germany/BW-RKI-I-867550/2022|EPI_ISL_13381335|2022-06-09
hCoV-19/Germany/BW-RKI-I-873833/2022|EPI_ISL_13386725|2022-06-08
hCoV-19/Germany/BW-RKI-I-874751/2022|EPI_ISL_13387840|2022-06-12
hCoV-19/Germany/BW-RKI-I-919374/2022|EPI_ISL_14381003|2022-07-14
hCoV-19/Germany/BW-RKI-I-927222/2022|EPI_ISL_14401350|2022-07-20
hCoV-19/Germany/BW-RKI-I-927234/2022|EPI_ISL_14401362|2022-07-21
hCoV-19/Germany/BW-RKI-I-934782/2022|EPI_ISL_14410768|2022-07-28
hCoV-19/Germany/BW-RKI-I-947000/2022|EPI_ISL_14431965|2022-08-03
hCoV-19/Germany/BW-RKI-I-950120/2022|EPI_ISL_14519183|2022-08-06
hCoV-19/Germany/BW-RKI-I-954050/2022|EPI_ISL_14523604|2022-08-11
hCoV-19/Germany/BW-RKI-I-959953/2022|EPI_ISL_14696088|2022-08-17
hCoV-19/Germany/BW-RKI-I-997326/2022|EPI_ISL_15444670|2022-09-29
hCoV-19/Germany/BY-MVP-000014407/2022|EPI_ISL_12567686|2022-04-12
hCoV-19/Germany/BY-MVP-000016079/2022|EPI_ISL_14471243|2022-05-02
hCoV-19/Germany/BY-RKI-I-214344/2021|EPI_ISL_17299696|2021-08-24
hCoV-19/Germany/BY-RKI-I-430748/2021|EPI_ISL_17300521|2021-12-28
hCoV-19/Germany/BY-RKI-I-687037/2022|EPI_ISL_11675312|2022-03-17

hCoV-19/Germany/BY-RKI-I-777051/2022|EPI_ISL_12667800|2022-02-04
hCoV-19/Germany/BY-RKI-I-808789/2022|EPI_ISL_12820085|2022-05-03
hCoV-19/Germany/BY-RKI-I-809014/2022|EPI_ISL_12820299|2022-05-03
hCoV-19/Germany/BY-RKI-I-874523/2022|EPI_ISL_13387649|2022-06-10
hCoV-19/Germany/HB-RKI-I-1053212/2021|EPI_ISL_16146985|2021-03-30
hCoV-19/Germany/HE-RKI-I-765610/2022|EPI_ISL_12519792|2022-04-12
hCoV-19/Germany/HE-RKI-I-798147/2022|EPI_ISL_12737400|2022-05-02
hCoV-19/Germany/HE-RKI-I-798349/2022|EPI_ISL_12737566|2022-05-03
hCoV-19/Germany/HE-RKI-I-798396/2022|EPI_ISL_12737601|2022-05-03
hCoV-19/Germany/HH-LIV-p3143/2020|EPI_ISL_16871444|2020-12-25
hCoV-19/Germany/HH-LIV-p3495/2020|EPI_ISL_16871441|2020-12-19
hCoV-19/Germany/HH-RKI-I-1063177/2021|EPI_ISL_16156190|2021-09-13
hCoV-19/Germany/HH-RKI-I-528180/2022|EPI_ISL_10020862|2022-02-03
hCoV-19/Germany/HH-RKI-I-528219/2022|EPI_ISL_10020901|2022-02-01
hCoV-19/Germany/HH-RKI-I-750296/2022|EPI_ISL_12361873|2022-04-13
hCoV-19/Germany/HH-RKI-I-880842/2022|EPI_ISL_13909756|2022-06-12
hCoV-19/Germany/MV-RKI-I-554457/2022|EPI_ISL_10294200|2022-02-03
hCoV-19/Germany/MV-RKI-I-586800/2022|EPI_ISL_10904369|2022-02-16
hCoV-19/Germany/MV-RKI-I-621392/2022|EPI_ISL_11464132|2022-03-02
hCoV-19/Germany/MV-RKI-I-665875/2022|EPI_ISL_11638886|2022-03-17
hCoV-19/Germany/MV-RKI-I-682802/2022|EPI_ISL_11668797|2022-03-16
hCoV-19/Germany/MV-RKI-I-742952/2022|EPI_ISL_12353530|2022-04-12
hCoV-19/Germany/MV-RKI-I-785941/2022|EPI_ISL_12675475|2022-05-03
hCoV-19/Germany/MV-UMG-20214100760/2021|EPI_ISL_17303047|2021-04-30
hCoV-19/Germany/NI-RKI-I-1047853/2020|EPI_ISL_16140894|2020-11-25
hCoV-19/Germany/NI-RKI-I-846522/2022|EPI_ISL_13240016|2022-05-25
hCoV-19/Germany/NI-RKI-I-846528/2022|EPI_ISL_13240022|2022-05-25
hCoV-19/Germany/NW-HHU-26759/2022|EPI_ISL_12879524|2022-05-03
hCoV-19/Germany/NW-HHU-7578/2021|EPI_ISL_3115769|2021-05-19
hCoV-19/Germany/NW-KRO-621/2020|EPI_ISL_2870125|2020-06-19
hCoV-19/Germany/NW-RKI-I-1023137/2022|EPI_ISL_15738533|2022-09-21
hCoV-19/Germany/NW-RKI-I-1025255/2022|EPI_ISL_15798293|2022-10-26
hCoV-19/Germany/NW-RKI-I-1043368/2022|EPI_ISL_16042408|2022-11-24
hCoV-19/Germany/NW-RKI-I-1043610/2022|EPI_ISL_16042632|2022-11-24
hCoV-19/Germany/NW-RKI-I-1045935/2021|EPI_ISL_16139822|2021-01-12
hCoV-19/Germany/NW-RKI-I-323177/2020|EPI_ISL_6463433|2020-08-11
hCoV-19/Germany/NW-RKI-I-507635/2022|EPI_ISL_9724674|2022-02-02
hCoV-19/Germany/NW-RKI-I-507640/2022|EPI_ISL_9724688|2022-02-02
hCoV-19/Germany/NW-RKI-I-508582/2022|EPI_ISL_9726461|2022-02-01
hCoV-19/Germany/NW-RKI-I-529437/2022|EPI_ISL_10022607|2022-02-02
hCoV-19/Germany/NW-RKI-I-531361/2022|EPI_ISL_10024271|2022-02-03
hCoV-19/Germany/NW-RKI-I-610897/2022|EPI_ISL_11452420|2022-03-02
hCoV-19/Germany/NW-RKI-I-610912/2022|EPI_ISL_11452426|2022-03-02

hCoV-19/Germany/NW-RKI-I-664165/2022|EPI_ISL_11620759|2022-03-16
hCoV-19/Germany/NW-RKI-I-665737/2022|EPI_ISL_11638574|2022-03-17
hCoV-19/Germany/NW-RKI-I-672671/2022|EPI_ISL_11655377|2022-03-17
hCoV-19/Germany/NW-RKI-I-672674/2022|EPI_ISL_11655382|2022-03-16
hCoV-19/Germany/NW-RKI-I-705492/2022|EPI_ISL_12016229|2022-03-02
hCoV-19/Germany/NW-RKI-I-737727/2022|EPI_ISL_12343018|2022-04-12
hCoV-19/Germany/NW-RKI-I-737759/2022|EPI_ISL_12343096|2022-04-12
hCoV-19/Germany/NW-RKI-I-737874/2022|EPI_ISL_12343371|2022-04-13
hCoV-19/Germany/NW-RKI-I-739546/2022|EPI_ISL_12346275|2022-04-12
hCoV-19/Germany/NW-RKI-I-767084/2022|EPI_ISL_12521233|2022-02-03
hCoV-19/Germany/NW-RKI-I-767155/2022|EPI_ISL_12521299|2022-02-03
hCoV-19/Germany/NW-RKI-I-785066/2022|EPI_ISL_12674883|2022-05-02
hCoV-19/Germany/NW-RKI-I-785104/2022|EPI_ISL_12674921|2022-05-02
hCoV-19/Germany/NW-RKI-I-785420/2022|EPI_ISL_12675163|2022-05-03
hCoV-19/Germany/NW-RKI-I-789568/2022|EPI_ISL_12678319|2022-05-02
hCoV-19/Germany/NW-RKI-I-789609/2022|EPI_ISL_12678369|2022-05-03
hCoV-19/Germany/NW-RKI-I-789641/2022|EPI_ISL_12678407|2022-05-03
hCoV-19/Germany/NW-RKI-I-789670/2022|EPI_ISL_12678443|2022-05-02
hCoV-19/Germany/NW-RKI-I-789676/2022|EPI_ISL_12678451|2022-05-02
hCoV-19/Germany/NW-RKI-I-789690/2022|EPI_ISL_12678470|2022-05-02
hCoV-19/Germany/NW-RKI-I-789708/2022|EPI_ISL_12678493|2022-05-02
hCoV-19/Germany/NW-RKI-I-800495/2022|EPI_ISL_12813346|2022-05-03
hCoV-19/Germany/NW-RKI-I-845455/2022|EPI_ISL_13239136|2022-05-27
hCoV-19/Germany/NW-RKI-I-845461/2022|EPI_ISL_13239142|2022-05-27
hCoV-19/Germany/NW-RKI-I-849349/2022|EPI_ISL_13244812|2022-05-25
hCoV-19/Germany/NW-RKI-I-869691/2022|EPI_ISL_13382882|2022-06-08
hCoV-19/Germany/NW-RKI-I-970606/2022|EPI_ISL_14979607|2022-07-25
hCoV-19/Germany/NW-RKI-I-984080/2022|EPI_ISL_15147710|2022-08-16
hCoV-19/Germany/NW-RKI-I-991330/2022|EPI_ISL_15266310|2022-09-29
hCoV-19/Germany/NW-RKI-I-992373/2022|EPI_ISL_15439548|2022-08-30
hCoV-19/Germany/RP-RKI-I-645955/2022|EPI_ISL_11595841|2022-03-01
hCoV-19/Germany/RP-RKI-I-654032/2022|EPI_ISL_11612052|2022-03-02
hCoV-19/Germany/RP-RKI-I-847782/2022|EPI_ISL_13240766|2022-05-28
hCoV-19/Germany/RP-RKI-I-851406/2022|EPI_ISL_13246374|2022-05-28
hCoV-19/Germany/RP-RKI-I-851415/2022|EPI_ISL_13246381|2022-05-28
hCoV-19/Germany/RP-RKI-I-851490/2022|EPI_ISL_13246441|2022-05-26
hCoV-19/Germany/RP-RKI-I-851493/2022|EPI_ISL_13246444|2022-05-28
hCoV-19/Germany/RP-RKI-I-851494/2022|EPI_ISL_13246445|2022-05-28
hCoV-19/Germany/RP-RKI-I-851533/2022|EPI_ISL_13246480|2022-05-28
hCoV-19/Germany/RP-RKI-I-851539/2022|EPI_ISL_13246486|2022-05-26
hCoV-19/Germany/RP-RKI-I-851580/2022|EPI_ISL_13246517|2022-05-28
hCoV-19/Germany/RP-RKI-I-851617/2022|EPI_ISL_13246544|2022-05-26
hCoV-19/Germany/RP-RKI-I-851731/2022|EPI_ISL_13246634|2022-05-26

hCoV-19/Germany/RP-RKI-I-872914/2022|EPI_ISL_13386050|2022-06-11

hCoV-19/Germany/RP-RKI-I-873108/2022|EPI_ISL_13386178|2022-06-11

hCoV-19/Germany/RP-RKI-I-885121/2022|EPI_ISL_13915784|2022-06-11

hCoV-19/Germany/SH-ChVir20054/2020|EPI_ISL_729417|2020-11-06

hCoV-19/Germany/SH-RKI-I-1052688/2021|EPI_ISL_16146648|2021-12-20

hCoV-19/Germany/SH-RKI-I-848420/2022|EPI_ISL_13241266|2022-05-25

hCoV-19/Germany/SH-RKI-I-869571/2022|EPI_ISL_13382767|2022-06-08

hCoV-19/Germany/SN-RKI-I-588988/2022|EPI_ISL_10905551|2022-02-16

hCoV-19/Germany/SN-RKI-I-589285/2022|EPI_ISL_10906135|2022-02-16

hCoV-19/Germany/SN-RKI-I-637433/2022|EPI_ISL_11480928|2022-03-01

hCoV-19/Germany/SN-RKI-I-680530/2022|EPI_ISL_11666582|2022-03-16

hCoV-19/Germany/SN-RKI-I-683125/2022|EPI_ISL_11669277|2022-03-17

hCoV-19/Germany/SN-RKI-I-809823/2022|EPI_ISL_12821069|2022-05-03

hCoV-19/Germany/ST-MD11663/2022|EPI_ISL_15806360|2022-09-30

hCoV-19/Germany/ST-MD12180/2022|EPI_ISL_15806693|2022-10-17

hCoV-19/Germany/ST-MD12401/2022|EPI_ISL_15806814|2022-10-26

hCoV-19/Germany/ST-MD12441/2022|EPI_ISL_15806850|2022-10-28

hCoV-19/Germany/ST-MD3844/2021|EPI_ISL_3087699|2021-05-20

hCoV-19/Germany/ST-RKI-I-588486/2022|EPI_ISL_10905360|2022-02-03

hCoV-19/Germany/ST-RKI-I-714174/2022|EPI_ISL_12026815|2022-03-17

hCoV-19/Germany/ST-RKI-I-737624/2022|EPI_ISL_12342749|2022-04-12

hCoV-19/Germany/ST-RKI-I-765898/2022|EPI_ISL_12520167|2022-03-17

hCoV-19/Germany/TH-RKI-I-1062987/2021|EPI_ISL_16156016|2021-10-14

hCoV-19/Germany/TH-RKI-I-605762/2022|EPI_ISL_10920166|2022-03-01

hCoV-19/Germany/un-C1316/2021|EPI_ISL_15589793|2021-06-22

hCoV-19/Germany/un-C2495/2021|EPI_ISL_15590109|2021-09-20

hCoV-19/Germany/un-C2805/2021|EPI_ISL_15590297|2021-10-18

hCoV-19/Germany/un-C680/2021|EPI_ISL_15590667|2021-03-29

hCoV-19/Ghana/UHAS-AG301/2021|EPI_ISL_15887000|2021-03-05

hCoV-19/Greece/26110/2022|EPI_ISL_13904535|2022-06-09

hCoV-19/Guangdong/20SF616/2020|EPI_ISL_428465|2020-01-24

hCoV-19/Guangdong/20SF812/2020|EPI_ISL_428475|2020-01-28

hCoV-19/Guangdong/20SF822/2020|EPI_ISL_428477|2020-01-28

hCoV-19/Guinea/LFHVG-G0463/2020|EPI_ISL_9569806|2020-08-02

hCoV-19/Haiti/34916/2020|EPI_ISL_15279672|2020-05-18

hCoV-19/Hangzhou/HZCDC0091/2020|EPI_ISL_421228|2020-01-21

hCoV-19/Hong_Kong/HK-HKPU-PU22MM416851/2022|EPI_ISL_12418097|2022-02-03

hCoV-19/Hong_Kong/HK-HKPU-PU22MV046976/2022|EPI_ISL_15680566|2022-08-29

hCoV-19/Hong_Kong/HK-HKPU-PU22MV672094/2022|EPI_ISL_15680216|2022-08-22

hCoV-19/Hong_Kong/HK-HKPU-PU22TM231167/2022|EPI_ISL_12418258|2022-02-04

hCoV-19/Hong_Kong/HK-HKPU-PU22TM231168/2022|EPI_ISL_12418255|2022-02-04

hCoV-19/Hong_Kong/HK-HKPU-PU22TM232621/2022|EPI_ISL_12418125|2022-02-04

hCoV-19/Hong_Kong/HK-HKPU-PUH077636535/2022|EPI_ISL_15679204|2022-07-13

hCoV-19/Hong_Kong/HK-HKPU-PUH10426558A/2022|EPI_ISL_15679235|2022-07-25
hCoV-19/Hong_Kong/HK-HKPU-PUN38384806A/2022|EPI_ISL_15679477|2022-08-22
hCoV-19/Hong_Kong/HK-HKPU-PUN384775465/2022|EPI_ISL_15684233|2022-07-15
hCoV-19/Hong_Kong/HK-HKPU-PUN510294475/2022|EPI_ISL_15680340|2022-08-11
hCoV-19/Hong_Kong/HK-HKPU-PUN512526985/2022|EPI_ISL_13140346|2022-05-03
hCoV-19/Hong_Kong/HK-HKPU-PUN513475713/2022|EPI_ISL_15680110|2022-07-27
hCoV-19/Hong_Kong/HK-HKPU-PUN516976675/2022|EPI_ISL_15680472|2022-08-15
hCoV-19/Hong_Kong/HK-HKPU-PUN517415421/2022|EPI_ISL_15683975|2022-07-14
hCoV-19/Hong_Kong/HK-HKPU-PUN518309579/2022|EPI_ISL_15680343|2022-08-11
hCoV-19/Hong_Kong/HK-HKPU-PUN520421593/2022|EPI_ISL_15680619|2022-08-29
hCoV-19/Hong_Kong/HK-HKPU-PUN523363187/2022|EPI_ISL_15679346|2022-08-05
hCoV-19/Hong_Kong/HK-HKPU-PUN525037570/2022|EPI_ISL_15680332|2022-08-10
hCoV-19/Hong_Kong/HK-HKPU-PUN525248326/2022|EPI_ISL_15679086|2022-08-05
hCoV-19/Hong_Kong/HK-HKPU-PUN525328915/2022|EPI_ISL_15683681|2022-07-14
hCoV-19/Hong_Kong/HK-HKPU-PUN525930054/2022|EPI_ISL_15684517|2022-07-22
hCoV-19/Hong_Kong/HK-HKPU-PUN526617198/2022|EPI_ISL_15683665|2022-07-26
hCoV-19/Hong_Kong/HK-HKPU-PUN683497912/2022|EPI_ISL_15680554|2022-08-22
hCoV-19/Hong_Kong/HK-HKPU-PUN683862343/2022|EPI_ISL_15679648|2022-08-30
hCoV-19/Hong_Kong/HK-HKPU-PUN683986301/2022|EPI_ISL_15683607|2022-07-18
hCoV-19/Hong_Kong/HK-HKPU-PUN684482293/2022|EPI_ISL_15683600|2022-07-21
hCoV-19/Hong_Kong/HK-HKPU-PUN684774103/2022|EPI_ISL_15680306|2022-08-09
hCoV-19/Hong_Kong/HK-HKPU-PUN684804517/2022|EPI_ISL_15683587|2022-07-25
hCoV-19/Hong_Kong/HK-HKPU-PUU039450685/2022|EPI_ISL_13696493|2022-06-08
hCoV-19/Hong_Kong/HKPU-00040/2020|EPI_ISL_2710814|2020-02-10
hCoV-19/Hong_Kong/HKPU-00048/2020|EPI_ISL_2710831|2020-02-11
hCoV-19/Hong_Kong/HKU-011/2020|EPI_ISL_16347501|2020-12-04
hCoV-19/Hong_Kong/HKU-902b/2020|EPI_ISL_434564|2020-01-24
hCoV-19/Hunan/HN-CIDC-P5/2020|EPI_ISL_539334|2020-01-25
hCoV-19/Iceland/3588/2020|EPI_ISL_827280|2020-11-01
hCoV-19/Iceland/DCGN-0007899/2021|EPI_ISL_16934494|2021-12-03
hCoV-19/Iceland/L-1736/2022|EPI_ISL_15284434|2022-09-29
hCoV-19/Iceland/L-565/2022|EPI_ISL_14047660|2022-07-19
hCoV-19/India/AN-ICMR-NIV-INSACOG-GSEQ-8526/2022|EPI_ISL_9419116|2022-01-01
hCoV-19/India/AP-CCMB-CIC4384/2022|EPI_ISL_10989508|2022-02-02
hCoV-19/India/AP-CCMB-CIC4402/2022|EPI_ISL_10989690|2022-02-02
hCoV-19/India/AP-CCMB-CIC4432/2022|EPI_ISL_10989451|2022-02-04
hCoV-19/India/AP-CCMB-CIC4458/2022|EPI_ISL_10989653|2022-02-05
hCoV-19/India/AP-CCMB-CIC4550/2022|EPI_ISL_10989790|2022-02-03
hCoV-19/India/AP-CCMB-CIC4614/2022|EPI_ISL_10989463|2022-02-04
hCoV-19/India/AP-CCMB-CIC4669/2022|EPI_ISL_10989769|2022-02-02
hCoV-19/India/AP-CCMB-CIC6310/2022|EPI_ISL_11228666|2022-02-17
hCoV-19/India/AP-CCMB-CID4069/2022|EPI_ISL_14910455|2022-08-02
hCoV-19/India/AS-DIB-238610/2021|EPI_ISL_17599412|2021-04-23

hCoV-19/India/CG-AIIMSR-VRDL-INSACOG-0709/2022|EPI_ISL_14668609|2022-07-14

hCoV-19/India/CG-AIIMSR-VRDL-INSACOG-0752/2022|EPI_ISL_14668610|2022-07-30

hCoV-19/India/CT-INSACOG/NII-1354/2022|EPI_ISL_15754346|2022-08-08

hCoV-19/India/DL-AIIMS10108/2020|EPI_ISL_17463912|2020-06-08

hCoV-19/India/GJ-GBRC118/2020|EPI_ISL_451163|2020-05-02

hCoV-19/India/GJ-GBRC120/2020|EPI_ISL_455017|2020-05-02

hCoV-19/India/GJ-INSACOG-GBRC6968/2022|EPI_ISL_13539260|2022-06-09

hCoV-19/India/GJ-INSACOG-GBRC7237/2022|EPI_ISL_13697054|2022-05-03

hCoV-19/India/GJ-INSACOG-GBRC7426/2022|EPI_ISL_13711258|2022-06-10

hCoV-19/India/GJ-INSACOG-GBRC8851/2022|EPI_ISL_15074041|2022-08-15

hCoV-19/India/GJ-INSACOG-GBRC9700/2022|EPI_ISL_15762492|2022-10-26

hCoV-19/India/GJ-INSACOG-GBRC9715/2022|EPI_ISL_15762507|2022-10-27

hCoV-19/India/HR-701791765/2022|EPI_ISL_11030545|2022-03-02

hCoV-19/India/HR-763446532/2022|EPI_ISL_14819026|2022-08-10

hCoV-19/India/HR-763446593/2022|EPI_ISL_14819029|2022-08-10

hCoV-19/India/HR-763446720/2022|EPI_ISL_14819031|2022-08-10

hCoV-19/India/HR-764429917/2022|EPI_ISL_14841679|2022-08-13

hCoV-19/India/HR-764874530/2022|EPI_ISL_14841687|2022-08-14

hCoV-19/India/JH-ICMR-NIV-INSACOG-SEQ-2592/2020|EPI_ISL_15038483|2020-10-30

hCoV-19/India/JK-759100813/2022|EPI_ISL_15192540|2022-07-26

hCoV-19/India/JK-759173457/2022|EPI_ISL_14841717|2022-07-27

hCoV-19/India/KA-20128_S99_R1_001/2022|EPI_ISL_15655729|2022-08-02

hCoV-19/India/KA-CBR-1402HAV041/2022|EPI_ISL_10306567|2022-02-03

hCoV-19/India/MH-GMCA-ICMR-INSACOG-C635478/2022|EPI_ISL_14923287|2022-08-31

hCoV-19/India/MH-ICMR-12371/2022|EPI_ISL_15074126|2022-08-30

hCoV-19/India/MH-ICMR-NIV-INSACOG-G-11624/2022|EPI_ISL_14861572|2022-07-21

hCoV-19/India/MH-ICMR-NIV-INSACOG-G-11860/2022|EPI_ISL_14405493|2022-06-09

hCoV-19/India/MH-ICMR-NIV-INSACOG-G-11962/2022|EPI_ISL_14405587|2022-06-11

hCoV-19/India/MH-ICMR-NIV-INSACOG-GSEQ-12362/2022|EPI_ISL_15854682|2022-08-21

hCoV-19/India/MH-ICMR-NIV-INSACOG-GSEQ-12426/2022|EPI_ISL_15854819|2022-09-02

hCoV-19/India/MH-ICMR-NIV-INSACOG-GSEQ-9790/2022|EPI_ISL_10523137|2022-02-02

hCoV-19/India/MH-ICMR-NIV-INSACOG-NCOV-22340/2022|EPI_ISL_9419120|2022-01-01

hCoV-19/India/MH-ICMR-NIV-INSACOG-NIV2221702/2022|EPI_ISL_14861471|2022-07-22

hCoV-19/India/MH-ICMR-NIV-INSACOG-NIV2222990/2022|EPI_ISL_15348888|2022-08-21

hCoV-19/India/MH-ICMR-NIV-INSACOG-NIVSARI-22-2150/2022|EPI_ISL_15348911|2022-08-29

hCoV-19/India/MH-INSACOG-CSIR-NEERI1296/2022|EPI_ISL_11427707|2022-02-16

hCoV-19/India/MH-INSACOG-CSIR-NEERI1791/2022|EPI_ISL_13093996|2022-05-28

hCoV-19/India/MH-INSACOG-CSIR-NEERI2409/2022|EPI_ISL_14567018|2022-07-16

hCoV-19/India/MH-INSACOG-CSIR-NEERI798/2022|EPI_ISL_10264128|2022-02-02

hCoV-19/India/MH-KasturbaMCGM-ICMR-INSACOG-WG4132/2022|EPI_ISL_13537276|2022-05-28

hCoV-19/India/MH-KasturbaMCGM-ICMR-INSACOG-WG4741/2022|EPI_ISL_14355869|2022-07-27

hCoV-19/India/MH-KasturbaMCGM-ICMR-INSACOG-WG4745/2022|EPI_ISL_14355873|2022-07-21

hCoV-19/India/MH-NCDC-2909361/2021|EPI_ISL_2955915|2021-04-06

hCoV-19/India/MP-AIIMS_B-ICMR-INSACOG-WGS-20/2022|EPI_ISL_10018642|2022-02-02

hCoV-19/India/MZ-INSACOG-1526500096041/2022|EPI_ISL_12737815|2022-02-05

hCoV-19/India/OR-ILSGS18497/2022|EPI_ISL_14228597|2022-07-24

hCoV-19/India/OR-ILSGS18907/2022|EPI_ISL_14601821|2022-07-29

hCoV-19/India/OR-ILSGS18929/2022|EPI_ISL_14601842|2022-07-28

hCoV-19/India/OR-ILSGS19107/2022|EPI_ISL_14601979|2022-08-05

hCoV-19/India/OR-ILSGS19217/2022|EPI_ISL_15153185|2022-08-08

hCoV-19/India/OR-ILSGS19403/2022|EPI_ISL_15153286|2022-09-02

hCoV-19/India/OR-RMRC_B-ICMR-INSACOG-BBS425799/2022|EPI_ISL_14357356|2022-07-13

hCoV-19/India/OR-RMRC_B-ICMR-INSACOG-BBS429142/2022|EPI_ISL_14814432|2022-08-18

hCoV-19/India/OR-RMRC_B-ICMR-INSACOG-BBS429588/2022|EPI_ISL_14987846|2022-08-26

hCoV-19/India/OR-RMRC_B-ICMR-INSACOG-BBS429644/2022|EPI_ISL_14987854|2022-08-27

hCoV-19/India/OR-RMRC_B-ICMR-INSACOG-BBS429661/2022|EPI_ISL_14987856|2022-08-29

hCoV-19/India/PB-VRDLGMCPTA-ICMR-INSACOG-670456868/2022|EPI_ISL_14147894|2022-02-02

hCoV-19/India/RJ-INSACOG-AIIMSJ-146841/2021|EPI_ISL_14806574|2021-06-17

hCoV-19/India/RJ-SMS-ICMR-INSACOG-TS-8031/2022|EPI_ISL_10943926|2022-02-03

hCoV-19/India/RJ-SMS-ICMR-INSACOG-TS-8055/2022|EPI_ISL_10943962|2022-02-03

hCoV-19/India/TG-CCMB-X42/2020|EPI_ISL_528854|2020-07-14

hCoV-19/India/TG-CDFD-E130074/2021|EPI_ISL_17608957|2021-12-24

hCoV-19/India/TG-GMCH-ICMR-INSACOG-311432/2022|EPI_ISL_8527529|2022-01-03

hCoV-19/India/TG-GMCH-ICMR-INSACOG-312616/2022|EPI_ISL_8527578|2022-01-04

hCoV-19/India/TN-CBR-CL1827/2021|EPI_ISL_14607807|2021-06-23

hCoV-19/India/TN-CDFD-O-98/2022|EPI_ISL_15469896|2022-10-12

hCoV-19/India/TN-ICMR-INSACOG-SPHL-2139/2021|EPI_ISL_15251354|2021-09-27

hCoV-19/India/TN-ICMR-INSACOG-SPHL1300/2021|EPI_ISL_15003409|2021-02-09

hCoV-19/India/TN-ICMR-NIRT-INSACOG-177/2020|EPI_ISL_2100191|2020-05-19

hCoV-19/India/TN-INSACOG-CBR-S-10688/2021|EPI_ISL_14680068|2021-10-18

hCoV-19/India/WB-IICB-002/2020|EPI_ISL_569860|2020-09-10

hCoV-19/Indonesia/JI-ITD-59570NT/2022|EPI_ISL_16027474|2022-11-10

hCoV-19/Indonesia/JK-FK_UNMUL-WGS-0029/2022|EPI_ISL_15783942|2022-09-29

hCoV-19/Indonesia/JK-NIHRD-MI.22.13790/2022|EPI_ISL_9843675|2022-02-01

hCoV-19/Indonesia/JK-NIHRD-WGS-22-35168/2022|EPI_ISL_16073718|2022-11-24

hCoV-19/Indonesia/KI-FK_UNMUL-10343/2021|EPI_ISL_16003686|2021-02-10

hCoV-19/Indonesia/RI-GS-PAMKI-GSILAB-1099136/2022|EPI_ISL_14470966|2022-07-25

hCoV-19/Iran/49110/2021|EPI_ISL_15755102|2021-03-31

hCoV-19/Iran/708/2020|EPI_ISL_15754873|2020-02-23

hCoV-19/Iran/A13316/2020|EPI_ISL_15755066|2020-03-29

hCoV-19/Iran/AG2451/2021|EPI_ISL_15755173|2021-08-15

hCoV-19/Iran/Mashhad-NIC-25mo-16/2022|EPI_ISL_14806946|2022-08-11

hCoV-19/Iran/Mashhad-NIC-6sh-42/2022|EPI_ISL_15013507|2022-08-16

hCoV-19/Iran/NIC-22sh-9/2022|EPI_ISL_15295470|2022-08-26

hCoV-19/Iran/Sari-NIC-6sh-31/2022|EPI_ISL_15013499|2022-07-29

hCoV-19/Iran/Tehran-NIC-46/2022|EPI_ISL_10083932|2022-02-05

hCoV-19/Iran/Tehran-NIC-9mo-1/2022|EPI_ISL_14445618|2022-07-30

hCoV-19/Iran/Yasuj-NIC-1sh-38/2022|EPI_ISL_14951893|2022-07-31

hCoV-19/Ireland/CW-NVRL-S22IRL00314972/2022|EPI_ISL_13445965|2022-05-01

hCoV-19/Ireland/D-FAU83736_052/2022|EPI_ISL_16097997|2022-11-19

hCoV-19/Ireland/D-NVRL-G22IRL30390/2022|EPI_ISL_13503028|2022-05-27

hCoV-19/Ireland/D-NVRL-S22IRL00298444/2022|EPI_ISL_13123599|2022-04-13

hCoV-19/Ireland/D-NVRL-S22IRL00314843/2022|EPI_ISL_13446071|2022-05-01

hCoV-19/Ireland/LH-NVRL-S22IRL00298022/2022|EPI_ISL_13123896|2022-04-12

hCoV-19/Ireland/MH-NVRL-S22IRL00314903/2022|EPI_ISL_13446416|2022-05-01

hCoV-19/Ireland/TA-NVRL-S22IRL00314983/2022|EPI_ISL_13446460|2022-05-01

hCoV-19/Israel/2089375/2020|EPI_ISL_447372|2020-04-01

hCoV-19/Israel/SMC-7093315/2022|EPI_ISL_14161323|2022-07-20

hCoV-19/Italy/CAM-TIGEM-IZSM-COLLI-23216/2022|EPI_ISL_8544448|2022-01-01

hCoV-19/Italy/CAM-TIGEM-IZSM-COLLI-26229/2022|EPI_ISL_10243836|2022-02-01

hCoV-19/Italy/CAM-TIGEM-IZSM-COLLI-26387/2022|EPI_ISL_10244185|2022-02-01

hCoV-19/Italy/CAM-TIGEM-IZSM-COLLI-35588/2022|EPI_ISL_14186299|2022-07-20

hCoV-19/Italy/EMR_AUSLRomagna_C023-22-24/2022|EPI_ISL_10736925|2022-03-01

hCoV-19/Italy/EMR_AUSLRomagna_C038-22-12/2022|EPI_ISL_12356138|2022-04-12

hCoV-19/Italy/EMR_AUSLRomagna_C039-22-39/2022|EPI_ISL_12355824|2022-04-13

hCoV-19/Italy/EMR_AUSLRomagna_C040-22-07/2022|EPI_ISL_12355628|2022-04-13

hCoV-19/Italy/EMR_AUSLRomagna_C043-22-39/2022|EPI_ISL_12540457|2022-05-01

hCoV-19/Italy/EMR_AUSLRomagna_C052-22-13/2022|EPI_ISL_13113748|2022-05-25

hCoV-19/Italy/EMR_AUSLRomagna_C053-22-26/2022|EPI_ISL_13113714|2022-05-25

hCoV-19/Italy/EMR_AUSLRomagna_C054-22-07/2022|EPI_ISL_13113693|2022-05-26

hCoV-19/Italy/EMR_AUSLRomagna_C110-22-24/2022|EPI_ISL_15630302|2022-10-26

hCoV-19/Italy/EMR-IZSLER-2022-148800-014-01/2022|EPI_ISL_12605965|2022-05-03

hCoV-19/Italy/EMR-IZSLER-2022-253193-021-01/2022|EPI_ISL_14585737|2022-08-02

hCoV-19/Italy/FVG-PN-64043843/2022|EPI_ISL_12685445|2022-05-03

hCoV-19/Italy/LAZ-AMC-2201034102-DS/2022|EPI_ISL_9353350|2022-01-03

hCoV-19/Italy/LAZ-AMC-220302477-DS/2022|EPI_ISL_11108643|2022-03-02

hCoV-19/Italy/LAZ-AMC-220316896-DS/2022|EPI_ISL_11799531|2022-03-16

hCoV-19/Italy/LAZ-FPG-2021-31/2021|EPI_ISL_14932560|2021-02-12

hCoV-19/Italy/LAZ-OPBG_4/2020|EPI_ISL_7671720|2020-10-01

hCoV-19/Italy/LOM_80019092BAS/2022|EPI_ISL_12565292|2022-05-03

hCoV-19/Italy/LOM_ASSTMonza_6000045999_20220302105400/2022|EPI_ISL_10865024|2022-03-02

hCoV-19/Italy/LOM_ASSTMonza_6000086016_20220503071000/2022|EPI_ISL_12563714|2022-05-03

hCoV-19/Italy/LOM_ASSTMonza_630631_20210604080400/2021|EPI_ISL_12897575|2021-06-04

hCoV-19/Italy/LOM_Sacco_Var_R08502/2022|EPI_ISL_12240067|2022-04-13

hCoV-19/Italy/LOM-UniMI-L278/2020|EPI_ISL_542242|2020-02-27

hCoV-19/Italy/PAB_SABES_1900889912/2022|EPI_ISL_11557108|2022-03-01

hCoV-19/Italy/PIE_IRCC_15881462/2022|EPI_ISL_11349230|2022-03-16

hCoV-19/Italy/TAA-PAB_SABES_1900709473/2021|EPI_ISL_16260002|2021-11-03

hCoV-19/Italy/TUS-AOUC-470/2022|EPI_ISL_12790166|2022-04-12

hCoV-19/Italy/TUS-AOUC-539/2022|EPI_ISL_12790098|2022-05-03

hCoV-19/Italy/TUS-AOUC-974/2022|EPI_ISL_15512658|2022-07-26

hCoV-19/Italy/TUS-C44/2020|EPI_ISL_738147|2020-02-25

hCoV-19/Italy/VEN-SR-29-B/2020|EPI_ISL_1064080|2020-02-26

hCoV-19/Japan/FK0157/2020|EPI_ISL_10342160|2020-10-22

hCoV-19/Japan/HiroFH218/2021|EPI_ISL_14442299|2021-10-10

hCoV-19/Japan/IC-11277/2022|EPI_ISL_13217712|2022-05-26

hCoV-19/Japan/IC-9269/2022|EPI_ISL_12807057|2022-05-03

hCoV-19/Japan/NO575700044/2021|EPI_ISL_17377042|2021-09-01

hCoV-19/Japan/OO1116A3796/2021|EPI_ISL_17376758|2021-08-12

hCoV-19/Japan/OPHO-Hb57-176/2022|EPI_ISL_13048316|2022-05-25

hCoV-19/Japan/OPHO-Hb71-382/2022|EPI_ISL_13952114|2022-07-14

hCoV-19/Japan/PG-0199/2020|EPI_ISL_684139|2020-02-27

hCoV-19/Japan/PG-0376/2020|EPI_ISL_480046|2020-02-22

hCoV-19/Japan/PG-169771/2020|EPI_ISL_8110946|2020-05-07

hCoV-19/Japan/PG-257672/2022|EPI_ISL_14113416|2022-05-29

hCoV-19/Japan/PG-405871/2022|EPI_ISL_16073440|2022-11-24

hCoV-19/Japan/PG-58098/2021|EPI_ISL_3183863|2021-05-20

hCoV-19/Japan/PG-59196/2021|EPI_ISL_3198240|2021-05-01

hCoV-19/Japan/PG-60621/2020|EPI_ISL_2885247|2020-08-09

hCoV-19/Japan/PG-63979/2021|EPI_ISL_3194183|2021-05-06

hCoV-19/Japan/PG-66039/2021|EPI_ISL_3187968|2021-05-18

hCoV-19/Japan/PG-70021/2021|EPI_ISL_3192274|2021-05-22

hCoV-19/Japan/TKYkbm14118/2022|EPI_ISL_12076246|2022-03-17

hCoV-19/Japan/TKYkbm19164/2022|EPI_ISL_13091884|2022-04-13

hCoV-19/Japan/TKYkbm39425/2022|EPI_ISL_14718689|2022-07-14

hCoV-19/Japan/TKYmbc13081/2022|EPI_ISL_15834806|2022-09-22

hCoV-19/Japan/TKYnat0014/2022|EPI_ISL_8919721|2022-01-01

hCoV-19/Japan/TKYnat0795/2022|EPI_ISL_13414232|2022-04-12

hCoV-19/Japan/TKYnat1172/2022|EPI_ISL_13512629|2022-05-25

hCoV-19/Japan/TKYnat1192/2022|EPI_ISL_13512647|2022-05-25

hCoV-19/Japan/TKYnat1320/2022|EPI_ISL_13530979|2022-05-26

hCoV-19/Japan/TKYnat2301/2022|EPI_ISL_13843369|2022-06-09

hCoV-19/Japan/TKYnat2415/2022|EPI_ISL_13843528|2022-06-12

hCoV-19/Japan/TKYnat6052/2022|EPI_ISL_14971144|2022-08-17

hCoV-19/Jiangsu/JS03/2020|EPI_ISL_411953|2020-01-24

hCoV-19/Kenya/MTMC_27_274/2021|EPI_ISL_17425665|2021-11-17

hCoV-19/Laos/LOMWRU-0998/2021|EPI_ISL_16700080|2021-09-24

hCoV-19/Latvia/2202011160/2022|EPI_ISL_13629245|2022-02-03

hCoV-19/Latvia/2202012250/2022|EPI_ISL_13628574|2022-02-03

hCoV-19/Latvia/2202013426/2022|EPI_ISL_13629288|2022-02-03

hCoV-19/Latvia/2202014435/2022|EPI_ISL_13630001|2022-02-04

hCoV-19/Latvia/2203000728/2022|EPI_ISL_13629537|2022-03-01

hCoV-19/Latvia/2203005094/2022|EPI_ISL_13628322|2022-03-02
hCoV-19/Latvia/22070220NO/2022|EPI_ISL_13628326|2022-02-05
hCoV-19/Lebanon/5578/2021|EPI_ISL_13829925|2021-02-09
hCoV-19/Lebanon/LAU-R97/2020|EPI_ISL_8311758|2020-01-10
hCoV-19/Lebanon/ML11_S47/2020|EPI_ISL_12470092|2020-04-03
hCoV-19/Libya/421067095/2021|EPI_ISL_16648345|2021-02-19
hCoV-19/Liechtenstein/FL-Risch-1117R14914/2022|EPI_ISL_16027481|2022-11-17
hCoV-19/Lithuania/LTU000_INMEDICA_1255286/2022|EPI_ISL_11826952|2022-03-17
hCoV-19/Lithuania/LTU000_KUL_5345028/2022|EPI_ISL_10316164|2022-02-02
hCoV-19/Lithuania/LTU000_KUL_5360050/2022|EPI_ISL_10851399|2022-02-17
hCoV-19/Luxembourg/LNS0492430/2022|EPI_ISL_12837218|2022-05-03
hCoV-19/Luxembourg/LNS1261847/2022|EPI_ISL_12451580|2022-02-16
hCoV-19/Luxembourg/LNS1297458/2022|EPI_ISL_12445875|2022-03-17
hCoV-19/Luxembourg/LNS1903878/2022|EPI_ISL_9853676|2022-02-02
hCoV-19/Luxembourg/LNS2470897/2021|EPI_ISL_3145395|2021-05-05
hCoV-19/Luxembourg/LNS2542993/2022|EPI_ISL_9853417|2022-02-05
hCoV-19/Luxembourg/LNS3591928/2022|EPI_ISL_13667643|2022-06-09
hCoV-19/Luxembourg/LNS4423374/2022|EPI_ISL_12452832|2022-04-12
hCoV-19/Luxembourg/LNS5093647/2022|EPI_ISL_12451792|2022-03-02
hCoV-19/Luxembourg/LNS5739154/2020|EPI_ISL_744288|2020-11-14
hCoV-19/Luxembourg/LNS5820934/2022|EPI_ISL_12837543|2022-05-02
hCoV-19/Luxembourg/LNS6843203/2022|EPI_ISL_12446234|2022-04-12
hCoV-19/Luxembourg/LNS7719337/2022|EPI_ISL_15354777|2022-09-26
hCoV-19/Luxembourg/LNS8271882/2022|EPI_ISL_14759792|2022-08-16
hCoV-19/Luxembourg/LNS9139202/2022|EPI_ISL_12703017|2022-04-13
hCoV-19/Malaysia/IMR_OS2369/2022|EPI_ISL_14116955|2022-06-10
hCoV-19/Malaysia/IMR_OS5532/2022|EPI_ISL_15314364|2022-05-26
hCoV-19/Malaysia/IMR_OS5675/2022|EPI_ISL_15348226|2022-06-08
hCoV-19/Malaysia/IMR_WC193755/2022|EPI_ISL_12942583|2022-03-16
hCoV-19/Malaysia/IMR_WC194740/2022|EPI_ISL_12891167|2022-03-16
hCoV-19/Malaysia/UiTM2085/2022|EPI_ISL_14687490|2022-07-30
hCoV-19/Malaysia/UiTM2339/2022|EPI_ISL_16121013|2022-11-18
hCoV-19/Malaysia/UiTM2344/2022|EPI_ISL_16121016|2022-11-21
hCoV-19/Malaysia/UiTM2354/2022|EPI_ISL_16121022|2022-11-16
hCoV-19/Malaysia/UNIMAS-GHML2002/2022|EPI_ISL_16030569|2022-11-13
hCoV-19/Mauritius/52473/2022|EPI_ISL_15908963|2022-09-30
hCoV-19/Mauritius/54147/2022|EPI_ISL_16097247|2022-10-29
hCoV-19/Mauritius/54178/2022|EPI_ISL_16097255|2022-10-29
hCoV-19/Mauritius/54268/2022|EPI_ISL_16097260|2022-10-31
hCoV-19/Mauritius/54335/2022|EPI_ISL_16097272|2022-11-01
hCoV-19/Mauritius/8730/2022|EPI_ISL_13665201|2022-02-04
hCoV-19/Mexico/CAM_IBT_IMSS_9169/2022|EPI_ISL_14806412|2022-08-04
hCoV-19/Mexico/CHH_CIAD-IMSS_01034008/2022|EPI_ISL_14901900|2022-08-22

hCoV-19/Mexico/CMX_INCMNSZ_CS-156307/2021|EPI_ISL_13984218|2021-02-04

hCoV-19/Mexico/CMX-13/2020|EPI_ISL_9433299|2020-09-04

hCoV-19/Mexico/CMX-94/2020|EPI_ISL_12625129|2020-09-10

hCoV-19/Mexico/CMX-INER-IBT-1150/2020|EPI_ISL_6316739|2020-07-09

hCoV-19/Mexico/CMX-INMEGEN-04-05-154/2020|EPI_ISL_4219889|2020-05-24

hCoV-19/Mexico/CMX-INMEGEN-04-07-30/2020|EPI_ISL_4219951|2020-06-05

hCoV-19/Mexico/CMX-INMEGEN-04-07-312/2020|EPI_ISL_4220077|2020-08-21

hCoV-19/Mexico/CMX-UNAM-1_4597/2021|EPI_ISL_16872202|2021-11-29

hCoV-19/Mexico/CMX-UNAM-4_4499/2021|EPI_ISL_16872058|2021-12-16

hCoV-19/Mexico/PUE_CIAD-IMSS_202201104193/2022|EPI_ISL_15310316|2022-09-22

hCoV-19/Mexico/VER_LANGEBIO_IMSS_10154/2022|EPI_ISL_14711601|2022-08-05

hCoV-19/Mexico/YUC_CIAD-IMSS_202201020296/2022|EPI_ISL_15932649|2022-10-25

hCoV-19/Montenegro/CO-00892_MNE000_1889462202/2022|EPI_ISL_14490197|2022-05-02

hCoV-19/Morocco/INH-108/2020|EPI_ISL_4899903|2020-02-02

hCoV-19/Mozambique/INS-PCS0025076/2022|EPI_ISL_15808020|2022-08-23

hCoV-19/Mozambique/MZ-L-IBV-97035972/2021|EPI_ISL_17075853|2021-07-01

hCoV-19/Mozambique/MZ-L-IBV-97036099/2021|EPI_ISL_17075946|2021-08-12

hCoV-19/Nepal/MTMC_05_069/2021|EPI_ISL_17425633|2021-11-21

hCoV-19/Nepal/NPHL-S-1556/2022|EPI_ISL_13388862|2022-05-02

hCoV-19/Nepal/NPHL-S-1754/2022|EPI_ISL_14569490|2022-07-21

hCoV-19/Nepal/NPHL-S-1780/2022|EPI_ISL_14807701|2022-08-16

hCoV-19/Netherlands/FR-RIVM-111984/2022|EPI_ISL_15177346|2022-08-29

hCoV-19/Netherlands/GE-EMC-450/2020|EPI_ISL_801450|2020-11-26

hCoV-19/Netherlands/GE-RIVM-103508/2022|EPI_ISL_13760687|2022-06-11

hCoV-19/Netherlands/GR-UMCG-MMB_5188/2022|EPI_ISL_12252277|2022-04-13

hCoV-19/Netherlands/GR-UMCG-SH_0109/2021|EPI_ISL_15165134|2021-02-11

hCoV-19/Netherlands/NB-EMC-905/2020|EPI_ISL_12683861|2020-07-07

hCoV-19/Netherlands/NH-AUMC-020866/2022|EPI_ISL_13132076|2022-05-27

hCoV-19/Netherlands/NH-AUMC-033093/2022|EPI_ISL_14913574|2022-08-30

hCoV-19/Netherlands/NH-AUMC-034366/2022|EPI_ISL_16073511|2022-11-28

hCoV-19/Netherlands/NH-AUMC-034412/2022|EPI_ISL_16073540|2022-11-28

hCoV-19/Netherlands/NH-RIVM-107420/2022|EPI_ISL_14239800|2022-07-14

hCoV-19/Netherlands/NH-RIVM-111759/2022|EPI_ISL_15112703|2022-08-26

hCoV-19/Netherlands/NH-RIVM-113291/2022|EPI_ISL_15216060|2022-09-19

hCoV-19/Netherlands/UT-RIVM-108249/2022|EPI_ISL_14702607|2022-07-22

hCoV-19/Netherlands/UT-RIVM-91546/2020|EPI_ISL_11119594|2020-10-16

hCoV-19/Netherlands/ZH-EMC-346/2020|EPI_ISL_523687|2020-07-31

hCoV-19/Netherlands/ZH-LUMC-2241/2022|EPI_ISL_12339483|2022-04-13

hCoV-19/Netherlands/ZH-LUMC-2565/2022|EPI_ISL_14093480|2022-05-29

hCoV-19/Netherlands/ZH-MZ-17/2020|EPI_ISL_15769735|2020-12-01

hCoV-19/Netherlands/ZH-RIVM-103492/2022|EPI_ISL_13760850|2022-06-10

hCoV-19/Netherlands/ZH-RIVM-46029/2021|EPI_ISL_3138103|2021-05-07

hCoV-19/Netherlands/ZH-RIVM-95276/2022|EPI_ISL_11840841|2022-02-01

hCoV-19/New_Zealand/22CH0104/2022|EPI_ISL_8648592|2022-01-04

hCoV-19/New_Zealand/22CH0128/2022|EPI_ISL_8648536|2022-01-05

hCoV-19/New_Zealand/22CH0153/2022|EPI_ISL_8825386|2022-01-01

hCoV-19/New_Zealand/22CH0159/2022|EPI_ISL_8825559|2022-01-02

hCoV-19/New_Zealand/22CH0161/2022|EPI_ISL_8825527|2022-01-03

hCoV-19/New_Zealand/22CH0173/2022|EPI_ISL_8825542|2022-01-07

hCoV-19/New_Zealand/22CH0266/2022|EPI_ISL_8825519|2022-01-09

hCoV-19/New_Zealand/22CH0267/2022|EPI_ISL_8826268|2022-01-09

hCoV-19/New_Zealand/22CH0272/2022|EPI_ISL_8826335|2022-01-10

hCoV-19/New_Zealand/22CH0312/2022|EPI_ISL_8825357|2022-01-11

hCoV-19/New_Zealand/22CH0353/2022|EPI_ISL_8826280|2022-01-13

hCoV-19/New_Zealand/22CH0359/2022|EPI_ISL_8826306|2022-01-15

hCoV-19/New_Zealand/22CH0361/2022|EPI_ISL_8825341|2022-01-15

hCoV-19/New_Zealand/22CH0415/2022|EPI_ISL_9210381|2022-01-17

hCoV-19/New_Zealand/22CH0429/2022|EPI_ISL_9210007|2022-01-17

hCoV-19/New_Zealand/22CH0430/2022|EPI_ISL_9210848|2022-01-17

hCoV-19/New_Zealand/22CH0454/2022|EPI_ISL_9209834|2022-01-16

hCoV-19/New_Zealand/22CH0464/2022|EPI_ISL_9210547|2022-01-15

hCoV-19/New_Zealand/22CH0478/2022|EPI_ISL_9210475|2022-01-15

hCoV-19/New_Zealand/22CH0497/2022|EPI_ISL_9210720|2022-01-15

hCoV-19/New_Zealand/22CH0506/2022|EPI_ISL_9209469|2022-01-12

hCoV-19/New_Zealand/22CH0511/2022|EPI_ISL_9209520|2022-01-13

hCoV-19/New_Zealand/22CH0519/2022|EPI_ISL_9210429|2022-01-11

hCoV-19/New_Zealand/22CH0520/2022|EPI_ISL_9210048|2022-01-13

hCoV-19/New_Zealand/22CH0532/2022|EPI_ISL_9209368|2022-01-12

hCoV-19/New_Zealand/22CH0542/2022|EPI_ISL_9209367|2022-01-11

hCoV-19/New_Zealand/22CH0543/2022|EPI_ISL_9209177|2022-01-11

hCoV-19/New_Zealand/22CH0566/2022|EPI_ISL_9210891|2022-01-22

hCoV-19/New_Zealand/22CH0573/2022|EPI_ISL_9210898|2022-01-23

hCoV-19/New_Zealand/22CH0595/2022|EPI_ISL_9210424|2022-01-02

hCoV-19/New_Zealand/22CH0596/2022|EPI_ISL_9209551|2022-01-02

hCoV-19/New_Zealand/22CH0597/2022|EPI_ISL_9209387|2022-01-02

hCoV-19/New_Zealand/22CH0614/2022|EPI_ISL_9209577|2022-01-24

hCoV-19/New_Zealand/22CH0620/2022|EPI_ISL_9210583|2022-01-24

hCoV-19/New_Zealand/22CH0635/2022|EPI_ISL_9434152|2022-01-25

hCoV-19/New_Zealand/22CH0675/2022|EPI_ISL_9636709|2022-01-31

hCoV-19/New_Zealand/22CH0689/2022|EPI_ISL_9636706|2022-01-31

hCoV-19/New_Zealand/22CH0692/2022|EPI_ISL_9636642|2022-01-31

hCoV-19/New_Zealand/22CH0697/2022|EPI_ISL_9636790|2022-02-01

hCoV-19/New_Zealand/22CH0722/2022|EPI_ISL_9636641|2022-01-28

hCoV-19/New_Zealand/22CH0757/2022|EPI_ISL_9636578|2022-02-02

hCoV-19/New_Zealand/22CH0761/2022|EPI_ISL_9636760|2022-02-02

hCoV-19/New_Zealand/22CH0763/2022|EPI_ISL_9636788|2022-02-02

hCoV-19/New_Zealand/22CH0768/2022|EPI_ISL_9636789|2022-02-02
hCoV-19/New_Zealand/22CH0770/2022|EPI_ISL_9636748|2022-02-02
hCoV-19/New_Zealand/22CH0777/2022|EPI_ISL_9636637|2022-01-29
hCoV-19/New_Zealand/22CH0779/2022|EPI_ISL_9671855|2022-01-28
hCoV-19/New_Zealand/22CH0786/2022|EPI_ISL_9671861|2022-01-27
hCoV-19/New_Zealand/22CH0799/2022|EPI_ISL_10188480|2022-02-03
hCoV-19/New_Zealand/22CH0801/2022|EPI_ISL_10188540|2022-02-03
hCoV-19/New_Zealand/22CH0815/2022|EPI_ISL_9636674|2022-01-25
hCoV-19/New_Zealand/22CH0816/2022|EPI_ISL_9636554|2022-01-25
hCoV-19/New_Zealand/22CH0818/2022|EPI_ISL_9636492|2022-01-25
hCoV-19/New_Zealand/22CH0821/2022|EPI_ISL_9636596|2022-01-25
hCoV-19/New_Zealand/22CH0846/2022|EPI_ISL_10188657|2022-02-04
hCoV-19/New_Zealand/22CH0851/2022|EPI_ISL_10188093|2022-02-04
hCoV-19/New_Zealand/22CH0856/2022|EPI_ISL_10188748|2022-02-04
hCoV-19/New_Zealand/22CH0871/2022|EPI_ISL_10188510|2022-02-07
hCoV-19/New_Zealand/22CH0886/2022|EPI_ISL_10188003|2022-02-06
hCoV-19/New_Zealand/22CH0900/2022|EPI_ISL_10188558|2022-02-04
hCoV-19/New_Zealand/22CH0902/2022|EPI_ISL_10188044|2022-02-03
hCoV-19/New_Zealand/22CH0904/2022|EPI_ISL_10188068|2022-02-02
hCoV-19/New_Zealand/22CH0908/2022|EPI_ISL_10188299|2022-02-07
hCoV-19/New_Zealand/22CH0910/2022|EPI_ISL_10188747|2022-02-09
hCoV-19/New_Zealand/22CH0914/2022|EPI_ISL_10188750|2022-02-10
hCoV-19/New_Zealand/22CH0915/2022|EPI_ISL_10188751|2022-02-10
hCoV-19/New_Zealand/22CH0926/2022|EPI_ISL_10188014|2022-02-10
hCoV-19/New_Zealand/22CH0927/2022|EPI_ISL_10188169|2022-02-10
hCoV-19/New_Zealand/22CH0931/2022|EPI_ISL_10188513|2022-02-11
hCoV-19/New_Zealand/22CH0933/2022|EPI_ISL_10188755|2022-02-11
hCoV-19/New_Zealand/22CH0957/2022|EPI_ISL_10188711|2022-02-14
hCoV-19/New_Zealand/22CH0961/2022|EPI_ISL_10186731|2022-02-14
hCoV-19/New_Zealand/22CH0963/2022|EPI_ISL_10188605|2022-02-13
hCoV-19/New_Zealand/22CH0964/2022|EPI_ISL_10188662|2022-02-13
hCoV-19/New_Zealand/22CH0965/2022|EPI_ISL_10186708|2022-02-14
hCoV-19/New_Zealand/22CH0969/2022|EPI_ISL_10188732|2022-02-12
hCoV-19/New_Zealand/22CH0982/2022|EPI_ISL_10188683|2022-02-11
hCoV-19/New_Zealand/22CH0996/2022|EPI_ISL_10188720|2022-02-11
hCoV-19/New_Zealand/22CH0999/2022|EPI_ISL_10188053|2022-02-10
hCoV-19/New_Zealand/22CH1001/2022|EPI_ISL_10188054|2022-02-10
hCoV-19/New_Zealand/22CH1006/2022|EPI_ISL_10188384|2022-02-12
hCoV-19/New_Zealand/22CH1014/2022|EPI_ISL_10188087|2022-02-12
hCoV-19/New_Zealand/22CH1022/2022|EPI_ISL_10188638|2022-02-12
hCoV-19/New_Zealand/22CH1034/2022|EPI_ISL_10188435|2022-02-11
hCoV-19/New_Zealand/22CH1036/2022|EPI_ISL_10188661|2022-02-11
hCoV-19/New_Zealand/22CH1042/2022|EPI_ISL_10188233|2022-02-15

hCoV-19/New_Zealand/22CH1107/2022|EPI_ISL_10188512|2022-02-12
hCoV-19/New_Zealand/22CH1130/2022|EPI_ISL_10188224|2022-02-13
hCoV-19/New_Zealand/22CH1134/2022|EPI_ISL_10188571|2022-02-13
hCoV-19/New_Zealand/22CH1141/2022|EPI_ISL_10626516|2022-02-13
hCoV-19/New_Zealand/22CH1154/2022|EPI_ISL_10626184|2022-02-16
hCoV-19/New_Zealand/22CH1165/2022|EPI_ISL_10626181|2022-02-17
hCoV-19/New_Zealand/22CH1176/2022|EPI_ISL_10626252|2022-02-17
hCoV-19/New_Zealand/22CH1190/2022|EPI_ISL_10626573|2022-02-18
hCoV-19/New_Zealand/22CH1195/2022|EPI_ISL_10626205|2022-02-15
hCoV-19/New_Zealand/22CH1196/2022|EPI_ISL_10626416|2022-02-15
hCoV-19/New_Zealand/22CH1218/2022|EPI_ISL_10626304|2022-02-21
hCoV-19/New_Zealand/22CH1226/2022|EPI_ISL_10626301|2022-02-17
hCoV-19/New_Zealand/22CH1236/2022|EPI_ISL_10626455|2022-02-20
hCoV-19/New_Zealand/22CH1239/2022|EPI_ISL_10626182|2022-02-20
hCoV-19/New_Zealand/22CH1247/2022|EPI_ISL_10626213|2022-02-22
hCoV-19/New_Zealand/22CH1258/2022|EPI_ISL_10626211|2022-02-23
hCoV-19/New_Zealand/22CH1270/2022|EPI_ISL_10626039|2022-02-15
hCoV-19/New_Zealand/22CH1290/2022|EPI_ISL_10626350|2022-02-20
hCoV-19/New_Zealand/22CH1336/2022|EPI_ISL_10626397|2022-02-28
hCoV-19/New_Zealand/22CH1357/2022|EPI_ISL_11017777|2022-02-23
hCoV-19/New_Zealand/22CH1360/2022|EPI_ISL_11017756|2022-02-22
hCoV-19/New_Zealand/22CH1387/2022|EPI_ISL_11017886|2022-02-28
hCoV-19/New_Zealand/22CH1415/2022|EPI_ISL_11017717|2022-02-28
hCoV-19/New_Zealand/22CH1465/2022|EPI_ISL_11017792|2022-03-04
hCoV-19/New_Zealand/22CH1474/2022|EPI_ISL_11017716|2022-03-05
hCoV-19/New_Zealand/22CH1480/2022|EPI_ISL_11017783|2022-03-06
hCoV-19/New_Zealand/22CH1481/2022|EPI_ISL_11017633|2022-03-06
hCoV-19/New_Zealand/22CH1482/2022|EPI_ISL_11017576|2022-03-06
hCoV-19/New_Zealand/22CH1489/2022|EPI_ISL_11017868|2022-03-07
hCoV-19/New_Zealand/22CH1498/2022|EPI_ISL_11866238|2022-03-07
hCoV-19/New_Zealand/22CH1502/2022|EPI_ISL_11866077|2022-03-07
hCoV-19/New_Zealand/22CH1525/2022|EPI_ISL_11017924|2022-03-06
hCoV-19/New_Zealand/22CH1526/2022|EPI_ISL_11017894|2022-03-01
hCoV-19/New_Zealand/22CH1536/2022|EPI_ISL_11331513|2022-03-04
hCoV-19/New_Zealand/22CH1537/2022|EPI_ISL_11017809|2022-03-04
hCoV-19/New_Zealand/22CH1540/2022|EPI_ISL_11017584|2022-03-04
hCoV-19/New_Zealand/22CH1544/2022|EPI_ISL_11017617|2022-03-04
hCoV-19/New_Zealand/22CH1549/2022|EPI_ISL_11017871|2022-03-04
hCoV-19/New_Zealand/22CH1562/2022|EPI_ISL_11331669|2022-03-05
hCoV-19/New_Zealand/22CH1569/2022|EPI_ISL_11331559|2022-03-06
hCoV-19/New_Zealand/22CH1574/2022|EPI_ISL_11331619|2022-03-06
hCoV-19/New_Zealand/22CH1596/2022|EPI_ISL_11866165|2022-03-07
hCoV-19/New_Zealand/22CH1598/2022|EPI_ISL_11017852|2022-03-05

hCoV-19/New_Zealand/22CH1617/2022|EPI_ISL_11017658|2022-03-04
hCoV-19/New_Zealand/22CH1629/2022|EPI_ISL_11331832|2022-03-09
hCoV-19/New_Zealand/22CH1636/2022|EPI_ISL_11331644|2022-03-09
hCoV-19/New_Zealand/22CH1642/2022|EPI_ISL_11331843|2022-03-10
hCoV-19/New_Zealand/22CH1644/2022|EPI_ISL_11331367|2022-03-09
hCoV-19/New_Zealand/22CH1669/2022|EPI_ISL_11331376|2022-03-07
hCoV-19/New_Zealand/22CH1676/2022|EPI_ISL_11331768|2022-03-09
hCoV-19/New_Zealand/22CH1687/2022|EPI_ISL_11331624|2022-03-09
hCoV-19/New_Zealand/22CH1693/2022|EPI_ISL_11331770|2022-03-08
hCoV-19/New_Zealand/22CH1707/2022|EPI_ISL_11331515|2022-03-10
hCoV-19/New_Zealand/22CH1709/2022|EPI_ISL_11331353|2022-03-10
hCoV-19/New_Zealand/22CH1730/2022|EPI_ISL_11331553|2022-03-11
hCoV-19/New_Zealand/22CH1746/2022|EPI_ISL_11331497|2022-03-12
hCoV-19/New_Zealand/22CH1758/2022|EPI_ISL_11331760|2022-03-13
hCoV-19/New_Zealand/22CH1778/2022|EPI_ISL_11331495|2022-03-13
hCoV-19/New_Zealand/22CH1795/2022|EPI_ISL_11331687|2022-03-14
hCoV-19/New_Zealand/22CH1826/2022|EPI_ISL_11331350|2022-03-10
hCoV-19/New_Zealand/22CH1959/2022|EPI_ISL_11331853|2022-03-17
hCoV-19/New_Zealand/22CH1969/2022|EPI_ISL_11331528|2022-03-17
hCoV-19/New_Zealand/22CH2032/2022|EPI_ISL_11331787|2022-03-20
hCoV-19/New_Zealand/22CH2085/2022|EPI_ISL_11866014|2022-03-22
hCoV-19/New_Zealand/22CH2100/2022|EPI_ISL_11865943|2022-03-22
hCoV-19/New_Zealand/22CH2188/2022|EPI_ISL_11866283|2022-03-17
hCoV-19/New_Zealand/22CH2201/2022|EPI_ISL_11865688|2022-03-18
hCoV-19/New_Zealand/22CH2241/2022|EPI_ISL_11866196|2022-03-21
hCoV-19/New_Zealand/22CH2325/2022|EPI_ISL_11865889|2022-03-28
hCoV-19/New_Zealand/22CH2331/2022|EPI_ISL_11866038|2022-03-28
hCoV-19/New_Zealand/22CH2333/2022|EPI_ISL_11865941|2022-03-28
hCoV-19/New_Zealand/22CH2404/2022|EPI_ISL_11866256|2022-03-27
hCoV-19/New_Zealand/22CH2424/2022|EPI_ISL_11865808|2022-03-28
hCoV-19/New_Zealand/22CH2497/2022|EPI_ISL_11866145|2022-04-01
hCoV-19/New_Zealand/22CH2505/2022|EPI_ISL_11865648|2022-04-01
hCoV-19/New_Zealand/22CH2515/2022|EPI_ISL_11865914|2022-04-02
hCoV-19/New_Zealand/22CH2520/2022|EPI_ISL_11865748|2022-04-02
hCoV-19/New_Zealand/22CH2524/2022|EPI_ISL_11865783|2022-04-02
hCoV-19/New_Zealand/22CH2527/2022|EPI_ISL_11865805|2022-04-02
hCoV-19/New_Zealand/22CH2545/2022|EPI_ISL_12546431|2022-04-04
hCoV-19/New_Zealand/22CH2560/2022|EPI_ISL_12545604|2022-04-04
hCoV-19/New_Zealand/22CH2578/2022|EPI_ISL_12545883|2022-04-01
hCoV-19/New_Zealand/22CH2582/2022|EPI_ISL_12546002|2022-04-03
hCoV-19/New_Zealand/22CH2584/2022|EPI_ISL_12545049|2022-04-01
hCoV-19/New_Zealand/22CH2611/2022|EPI_ISL_12545864|2022-04-05
hCoV-19/New_Zealand/22CH2623/2022|EPI_ISL_12545012|2022-04-05

hCoV-19/New_Zealand/22CH2625/2022|EPI_ISL_12546430|2022-04-05
hCoV-19/New_Zealand/22CH2636/2022|EPI_ISL_12545506|2022-04-03
hCoV-19/New_Zealand/22CH2648/2022|EPI_ISL_12545149|2022-04-04
hCoV-19/New_Zealand/22CH2649/2022|EPI_ISL_12546502|2022-04-05
hCoV-19/New_Zealand/22CH2650/2022|EPI_ISL_12545126|2022-04-03
hCoV-19/New_Zealand/22CH2654/2022|EPI_ISL_12545708|2022-04-06
hCoV-19/New_Zealand/22CH2656/2022|EPI_ISL_12546452|2022-04-06
hCoV-19/New_Zealand/22CH2658/2022|EPI_ISL_12546396|2022-04-06
hCoV-19/New_Zealand/22CH2663/2022|EPI_ISL_12545098|2022-04-06
hCoV-19/New_Zealand/22CH2668/2022|EPI_ISL_12545383|2022-04-06
hCoV-19/New_Zealand/22CH2690/2022|EPI_ISL_12545189|2022-04-05
hCoV-19/New_Zealand/22CH2697/2022|EPI_ISL_12546449|2022-04-05
hCoV-19/New_Zealand/22CH2701/2022|EPI_ISL_12546054|2022-04-06
hCoV-19/New_Zealand/22CH2713/2022|EPI_ISL_12545728|2022-04-06
hCoV-19/New_Zealand/22CH2727/2022|EPI_ISL_12546385|2022-04-07
hCoV-19/New_Zealand/22CH2730/2022|EPI_ISL_12546460|2022-04-07
hCoV-19/New_Zealand/22CH2739/2022|EPI_ISL_12546371|2022-04-08
hCoV-19/New_Zealand/22CH2764/2022|EPI_ISL_12545226|2022-04-09
hCoV-19/New_Zealand/22CH2769/2022|EPI_ISL_12545252|2022-04-09
hCoV-19/New_Zealand/22CH2779/2022|EPI_ISL_12545718|2022-04-10
hCoV-19/New_Zealand/22CH2789/2022|EPI_ISL_12545366|2022-04-11
hCoV-19/New_Zealand/22CH2793/2022|EPI_ISL_12545244|2022-04-11
hCoV-19/New_Zealand/22CH2797/2022|EPI_ISL_12546123|2022-04-11
hCoV-19/New_Zealand/22CH2804/2022|EPI_ISL_12546432|2022-04-11
hCoV-19/New_Zealand/22CH2807/2022|EPI_ISL_12546370|2022-04-12
hCoV-19/New_Zealand/22CH2812/2022|EPI_ISL_12545651|2022-04-07
hCoV-19/New_Zealand/22CH2817/2022|EPI_ISL_12545317|2022-04-08
hCoV-19/New_Zealand/22CH2827/2022|EPI_ISL_12546252|2022-04-09
hCoV-19/New_Zealand/22CH2832/2022|EPI_ISL_12546479|2022-04-08
hCoV-19/New_Zealand/22CH2836/2022|EPI_ISL_12546331|2022-04-11
hCoV-19/New_Zealand/22CH2840/2022|EPI_ISL_12546006|2022-04-12
hCoV-19/New_Zealand/22CH2848/2022|EPI_ISL_12544940|2022-04-11
hCoV-19/New_Zealand/22CH2851/2022|EPI_ISL_12546466|2022-04-09
hCoV-19/New_Zealand/22CH2873/2022|EPI_ISL_12546293|2022-04-12
hCoV-19/New_Zealand/22CH2875/2022|EPI_ISL_12546475|2022-04-13
hCoV-19/New_Zealand/22CH2886/2022|EPI_ISL_12545648|2022-04-13
hCoV-19/New_Zealand/22CH2890/2022|EPI_ISL_12546078|2022-04-14
hCoV-19/New_Zealand/22CH2901/2022|EPI_ISL_12545974|2022-04-11
hCoV-19/New_Zealand/22CH2915/2022|EPI_ISL_12546259|2022-04-12
hCoV-19/New_Zealand/22CH2918/2022|EPI_ISL_12544966|2022-04-12
hCoV-19/New_Zealand/22CH2919/2022|EPI_ISL_12544933|2022-04-12
hCoV-19/New_Zealand/22CH2923/2022|EPI_ISL_12544986|2022-04-13
hCoV-19/New_Zealand/22CH2924/2022|EPI_ISL_12545255|2022-04-15

hCoV-19/New_Zealand/22CH2929/2022|EPI_ISL_12545612|2022-04-14
hCoV-19/New_Zealand/22CH2933/2022|EPI_ISL_12545731|2022-04-14
hCoV-19/New_Zealand/22CH2934/2022|EPI_ISL_12545437|2022-04-14
hCoV-19/New_Zealand/22CH2944/2022|EPI_ISL_12545761|2022-04-15
hCoV-19/New_Zealand/22CH2949/2022|EPI_ISL_12545624|2022-04-15
hCoV-19/New_Zealand/22CH2955/2022|EPI_ISL_12545710|2022-04-16
hCoV-19/New_Zealand/22CH2958/2022|EPI_ISL_12545836|2022-04-16
hCoV-19/New_Zealand/22CH2963/2022|EPI_ISL_12545725|2022-04-16
hCoV-19/New_Zealand/22CH2983/2022|EPI_ISL_12546359|2022-04-17
hCoV-19/New_Zealand/22CH2987/2022|EPI_ISL_12545969|2022-04-18
hCoV-19/New_Zealand/22CH2996/2022|EPI_ISL_12546480|2022-04-18
hCoV-19/New_Zealand/22CH3003/2022|EPI_ISL_12545293|2022-04-19
hCoV-19/New_Zealand/22CH3008/2022|EPI_ISL_12544939|2022-04-19
hCoV-19/New_Zealand/22CH3013/2022|EPI_ISL_12546505|2022-04-19
hCoV-19/New_Zealand/22CH3017/2022|EPI_ISL_12545364|2022-04-19
hCoV-19/New_Zealand/22CH3019/2022|EPI_ISL_12545266|2022-04-16
hCoV-19/New_Zealand/22CH3031/2022|EPI_ISL_12545124|2022-04-13
hCoV-19/New_Zealand/22CH3042/2022|EPI_ISL_12545550|2022-04-14
hCoV-19/New_Zealand/22CH3043/2022|EPI_ISL_12546100|2022-04-15
hCoV-19/New_Zealand/22CH3046/2022|EPI_ISL_12545575|2022-04-14
hCoV-19/New_Zealand/22CH3093/2022|EPI_ISL_12545950|2022-04-20
hCoV-19/New_Zealand/22CH3099/2022|EPI_ISL_12545997|2022-04-20
hCoV-19/New_Zealand/22CH3103/2022|EPI_ISL_12545837|2022-04-21
hCoV-19/New_Zealand/22CH3109/2022|EPI_ISL_12546424|2022-04-19
hCoV-19/New_Zealand/22CH3113/2022|EPI_ISL_12545331|2022-04-19
hCoV-19/New_Zealand/22CH3134/2022|EPI_ISL_12546086|2022-04-19
hCoV-19/New_Zealand/22CH3135/2022|EPI_ISL_12545726|2022-04-19
hCoV-19/New_Zealand/22CH3137/2022|EPI_ISL_12545121|2022-04-19
hCoV-19/New_Zealand/22CH3151/2022|EPI_ISL_12545904|2022-04-21
hCoV-19/New_Zealand/22CH3152/2022|EPI_ISL_12546301|2022-04-21
hCoV-19/New_Zealand/22CH3155/2022|EPI_ISL_12545933|2022-04-21
hCoV-19/New_Zealand/22CH3158/2022|EPI_ISL_12545808|2022-04-21
hCoV-19/New_Zealand/22CH3166/2022|EPI_ISL_12545005|2022-04-22
hCoV-19/New_Zealand/22CH3170/2022|EPI_ISL_12545527|2022-04-22
hCoV-19/New_Zealand/22CH3172/2022|EPI_ISL_12546003|2022-04-21
hCoV-19/New_Zealand/22CH3179/2022|EPI_ISL_12545324|2022-04-20
hCoV-19/New_Zealand/22CH3181/2022|EPI_ISL_12545941|2022-04-20
hCoV-19/New_Zealand/22CH3184/2022|EPI_ISL_12546443|2022-04-20
hCoV-19/New_Zealand/22CH3185/2022|EPI_ISL_12545824|2022-04-20
hCoV-19/New_Zealand/22CH3216/2022|EPI_ISL_12545302|2022-04-24
hCoV-19/New_Zealand/22CH3217/2022|EPI_ISL_12545983|2022-04-14
hCoV-19/New_Zealand/22CH3219/2022|EPI_ISL_12545494|2022-04-19
hCoV-19/New_Zealand/22CH3224/2022|EPI_ISL_12546412|2022-04-24

hCoV-19/New_Zealand/22CH3227/2022|EPI_ISL_12546418|2022-04-24
hCoV-19/New_Zealand/22CH3251/2022|EPI_ISL_12545442|2022-04-21
hCoV-19/New_Zealand/22CH3257/2022|EPI_ISL_12545945|2022-04-21
hCoV-19/New_Zealand/22CH3281/2022|EPI_ISL_12546112|2022-04-24
hCoV-19/New_Zealand/22CH3298/2022|EPI_ISL_12546192|2022-04-27
hCoV-19/New_Zealand/22CH3324/2022|EPI_ISL_12544990|2022-04-26
hCoV-19/New_Zealand/22CH3343/2022|EPI_ISL_12546066|2022-04-26
hCoV-19/New_Zealand/22CH3369/2022|EPI_ISL_12545072|2022-04-28
hCoV-19/New_Zealand/22CH3383/2022|EPI_ISL_12545802|2022-04-27
hCoV-19/New_Zealand/22CH3399/2022|EPI_ISL_12545910|2022-04-29
hCoV-19/New_Zealand/22CH3412/2022|EPI_ISL_12546474|2022-04-29
hCoV-19/New_Zealand/22CH3420/2022|EPI_ISL_12545254|2022-04-30
hCoV-19/New_Zealand/22CH3435/2022|EPI_ISL_12545953|2022-05-01
hCoV-19/New_Zealand/22CH3447/2022|EPI_ISL_12546035|2022-04-28
hCoV-19/New_Zealand/22CH3469/2022|EPI_ISL_12545388|2022-04-28
hCoV-19/New_Zealand/22CH3497/2022|EPI_ISL_12805751|2022-05-02
hCoV-19/New_Zealand/22CH3523/2022|EPI_ISL_12545516|2022-05-02
hCoV-19/New_Zealand/22CH3531/2022|EPI_ISL_12805368|2022-05-02
hCoV-19/New_Zealand/22CH3546/2022|EPI_ISL_12805699|2022-05-02
hCoV-19/New_Zealand/22CH3552/2022|EPI_ISL_12805986|2022-05-03
hCoV-19/New_Zealand/22CH3560/2022|EPI_ISL_12805772|2022-05-03
hCoV-19/New_Zealand/22CH3566/2022|EPI_ISL_12806277|2022-05-03
hCoV-19/New_Zealand/22CH3579/2022|EPI_ISL_12806213|2022-05-04
hCoV-19/New_Zealand/22CH3581/2022|EPI_ISL_12805567|2022-05-04
hCoV-19/New_Zealand/22CH3584/2022|EPI_ISL_12805891|2022-05-04
hCoV-19/New_Zealand/22CH3591/2022|EPI_ISL_12805769|2022-05-04
hCoV-19/New_Zealand/22CH3593/2022|EPI_ISL_12805893|2022-05-02
hCoV-19/New_Zealand/22CH3599/2022|EPI_ISL_12805670|2022-05-02
hCoV-19/New_Zealand/22CH3608/2022|EPI_ISL_12805712|2022-05-01
hCoV-19/New_Zealand/22CH3609/2022|EPI_ISL_12805514|2022-05-02
hCoV-19/New_Zealand/22CH3619/2022|EPI_ISL_12805921|2022-05-03
hCoV-19/New_Zealand/22CH3637/2022|EPI_ISL_12805391|2022-05-03
hCoV-19/New_Zealand/22CH3640/2022|EPI_ISL_12806145|2022-05-04
hCoV-19/New_Zealand/22CH3651/2022|EPI_ISL_12805759|2022-05-05
hCoV-19/New_Zealand/22CH3654/2022|EPI_ISL_12805577|2022-05-05
hCoV-19/New_Zealand/22CH3656/2022|EPI_ISL_12805710|2022-05-05
hCoV-19/New_Zealand/22CH3668/2022|EPI_ISL_12805409|2022-05-05
hCoV-19/New_Zealand/22CH3698/2022|EPI_ISL_12805396|2022-05-07
hCoV-19/New_Zealand/22CH3699/2022|EPI_ISL_12805870|2022-05-07
hCoV-19/New_Zealand/22CH3706/2022|EPI_ISL_12805867|2022-05-08
hCoV-19/New_Zealand/22CH3712/2022|EPI_ISL_12805423|2022-05-09
hCoV-19/New_Zealand/22CH3716/2022|EPI_ISL_12806168|2022-05-05
hCoV-19/New_Zealand/22CH3727/2022|EPI_ISL_12805542|2022-05-09

hCoV-19/New_Zealand/22CH3737/2022|EPI_ISL_12805757|2022-05-09
hCoV-19/New_Zealand/22CH3741/2022|EPI_ISL_12806208|2022-05-09
hCoV-19/New_Zealand/22CH3749/2022|EPI_ISL_12806284|2022-05-10
hCoV-19/New_Zealand/22CH3774/2022|EPI_ISL_12805490|2022-05-06
hCoV-19/New_Zealand/22CH3789/2022|EPI_ISL_12805990|2022-05-08
hCoV-19/New_Zealand/22CH3808/2022|EPI_ISL_12805516|2022-05-09
hCoV-19/New_Zealand/22CH3812/2022|EPI_ISL_12805643|2022-05-10
hCoV-19/New_Zealand/22CH3813/2022|EPI_ISL_12805999|2022-05-10
hCoV-19/New_Zealand/22CH3849/2022|EPI_ISL_12806235|2022-05-09
hCoV-19/New_Zealand/22CH3866/2022|EPI_ISL_12806045|2022-05-12
hCoV-19/New_Zealand/22CH3867/2022|EPI_ISL_12805586|2022-05-11
hCoV-19/New_Zealand/22CH3872/2022|EPI_ISL_12806279|2022-05-11
hCoV-19/New_Zealand/22CH3882/2022|EPI_ISL_12805723|2022-05-11
hCoV-19/New_Zealand/22CH3892/2022|EPI_ISL_12805738|2022-05-12
hCoV-19/New_Zealand/22CH3903/2022|EPI_ISL_12805394|2022-05-11
hCoV-19/New_Zealand/22CH3925/2022|EPI_ISL_12805468|2022-05-10
hCoV-19/New_Zealand/22CH3927/2022|EPI_ISL_12806127|2022-05-11
hCoV-19/New_Zealand/22CH3956/2022|EPI_ISL_12805877|2022-05-15
hCoV-19/New_Zealand/22CH3988/2022|EPI_ISL_12805942|2022-05-12
hCoV-19/New_Zealand/22CH3991/2022|EPI_ISL_12805826|2022-05-11
hCoV-19/New_Zealand/22CH4003/2022|EPI_ISL_13437872|2022-05-16
hCoV-19/New_Zealand/22CH4037/2022|EPI_ISL_13437117|2022-05-11
hCoV-19/New_Zealand/22CH4042/2022|EPI_ISL_13438652|2022-05-14
hCoV-19/New_Zealand/22CH4044/2022|EPI_ISL_13438257|2022-05-15
hCoV-19/New_Zealand/22CH4048/2022|EPI_ISL_13438135|2022-05-17
hCoV-19/New_Zealand/22CH4050/2022|EPI_ISL_13437725|2022-05-17
hCoV-19/New_Zealand/22CH4051/2022|EPI_ISL_13438419|2022-05-17
hCoV-19/New_Zealand/22CH4066/2022|EPI_ISL_13437980|2022-05-17
hCoV-19/New_Zealand/22CH4072/2022|EPI_ISL_13437958|2022-05-18
hCoV-19/New_Zealand/22CH4079/2022|EPI_ISL_13438541|2022-05-15
hCoV-19/New_Zealand/22CH4084/2022|EPI_ISL_13438170|2022-05-16
hCoV-19/New_Zealand/22CH4095/2022|EPI_ISL_13437425|2022-05-14
hCoV-19/New_Zealand/22CH4102/2022|EPI_ISL_13438973|2022-05-18
hCoV-19/New_Zealand/22CH4108/2022|EPI_ISL_13438469|2022-05-12
hCoV-19/New_Zealand/22CH4117/2022|EPI_ISL_13436660|2022-05-17
hCoV-19/New_Zealand/22CH4125/2022|EPI_ISL_13438181|2022-05-16
hCoV-19/New_Zealand/22CH4132/2022|EPI_ISL_13438865|2022-05-16
hCoV-19/New_Zealand/22CH4139/2022|EPI_ISL_13437536|2022-05-17
hCoV-19/New_Zealand/22CH4142/2022|EPI_ISL_13436799|2022-05-18
hCoV-19/New_Zealand/22CH4166/2022|EPI_ISL_13436869|2022-05-19
hCoV-19/New_Zealand/22CH4168/2022|EPI_ISL_13437165|2022-05-19
hCoV-19/New_Zealand/22CH4169/2022|EPI_ISL_13437513|2022-05-19
hCoV-19/New_Zealand/22CH4177/2022|EPI_ISL_13438082|2022-05-19

hCoV-19/New_Zealand/22CH4191/2022|EPI_ISL_13437906|2022-05-21
hCoV-19/New_Zealand/22CH4194/2022|EPI_ISL_13437239|2022-05-20
hCoV-19/New_Zealand/22CH4198/2022|EPI_ISL_13437749|2022-05-20
hCoV-19/New_Zealand/22CH4203/2022|EPI_ISL_13437001|2022-05-20
hCoV-19/New_Zealand/22CH4235/2022|EPI_ISL_13437982|2022-05-19
hCoV-19/New_Zealand/22CH4238/2022|EPI_ISL_13438603|2022-05-19
hCoV-19/New_Zealand/22CH4241/2022|EPI_ISL_13437811|2022-05-21
hCoV-19/New_Zealand/22CH4244/2022|EPI_ISL_13437878|2022-05-19
hCoV-19/New_Zealand/22CH4247/2022|EPI_ISL_13438916|2022-05-19
hCoV-19/New_Zealand/22CH4248/2022|EPI_ISL_13438827|2022-05-20
hCoV-19/New_Zealand/22CH4251/2022|EPI_ISL_13438292|2022-05-22
hCoV-19/New_Zealand/22CH4253/2022|EPI_ISL_13438032|2022-05-23
hCoV-19/New_Zealand/22CH4263/2022|EPI_ISL_13436777|2022-05-22
hCoV-19/New_Zealand/22CH4267/2022|EPI_ISL_13436977|2022-05-23
hCoV-19/New_Zealand/22CH4275/2022|EPI_ISL_13437246|2022-05-18
hCoV-19/New_Zealand/22CH4293/2022|EPI_ISL_13437062|2022-05-22
hCoV-19/New_Zealand/22CH4301/2022|EPI_ISL_13764163|2022-05-22
hCoV-19/New_Zealand/22CH4324/2022|EPI_ISL_13437627|2022-05-22
hCoV-19/New_Zealand/22CH4337/2022|EPI_ISL_13437178|2022-05-24
hCoV-19/New_Zealand/22CH4361/2022|EPI_ISL_13436987|2022-05-23
hCoV-19/New_Zealand/22CH4363/2022|EPI_ISL_13438819|2022-05-24
hCoV-19/New_Zealand/22CH4393/2022|EPI_ISL_13437077|2022-05-25
hCoV-19/New_Zealand/22CH4394/2022|EPI_ISL_13436838|2022-05-25
hCoV-19/New_Zealand/22CH4396/2022|EPI_ISL_13437422|2022-05-23
hCoV-19/New_Zealand/22CH4398/2022|EPI_ISL_13437198|2022-05-25
hCoV-19/New_Zealand/22CH4402/2022|EPI_ISL_13437496|2022-05-24
hCoV-19/New_Zealand/22CH4450/2022|EPI_ISL_13437263|2022-05-27
hCoV-19/New_Zealand/22CH4475/2022|EPI_ISL_13437086|2022-05-29
hCoV-19/New_Zealand/22CH4478/2022|EPI_ISL_13438543|2022-05-29
hCoV-19/New_Zealand/22CH4479/2022|EPI_ISL_13436964|2022-05-29
hCoV-19/New_Zealand/22CH4481/2022|EPI_ISL_13438037|2022-05-30
hCoV-19/New_Zealand/22CH4485/2022|EPI_ISL_13438161|2022-05-27
hCoV-19/New_Zealand/22CH4502/2022|EPI_ISL_13437901|2022-05-25
hCoV-19/New_Zealand/22CH4505/2022|EPI_ISL_13437559|2022-05-29
hCoV-19/New_Zealand/22CH4507/2022|EPI_ISL_13437764|2022-05-27
hCoV-19/New_Zealand/22CH4510/2022|EPI_ISL_13438368|2022-05-28
hCoV-19/New_Zealand/22CH4512/2022|EPI_ISL_13437576|2022-05-28
hCoV-19/New_Zealand/22CH4513/2022|EPI_ISL_13437142|2022-05-27
hCoV-19/New_Zealand/22CH4516/2022|EPI_ISL_13436768|2022-05-29
hCoV-19/New_Zealand/22CH4520/2022|EPI_ISL_13437027|2022-05-28
hCoV-19/New_Zealand/22CH4530/2022|EPI_ISL_13437530|2022-05-30
hCoV-19/New_Zealand/22CH4532/2022|EPI_ISL_13437622|2022-05-31
hCoV-19/New_Zealand/22CH4535/2022|EPI_ISL_13438106|2022-05-31

hCoV-19/New_Zealand/22CH4540/2022|EPI_ISL_13437819|2022-05-30
hCoV-19/New_Zealand/22CH4541/2022|EPI_ISL_13437768|2022-05-30
hCoV-19/New_Zealand/22CH4544/2022|EPI_ISL_13438489|2022-05-26
hCoV-19/New_Zealand/22CH4559/2022|EPI_ISL_13438501|2022-05-31
hCoV-19/New_Zealand/22CH4560/2022|EPI_ISL_13438542|2022-05-31
hCoV-19/New_Zealand/22CH4592/2022|EPI_ISL_13436898|2022-05-30
hCoV-19/New_Zealand/22CH4593/2022|EPI_ISL_13436814|2022-05-30
hCoV-19/New_Zealand/22CH4595/2022|EPI_ISL_13436726|2022-05-27
hCoV-19/New_Zealand/22CH4605/2022|EPI_ISL_13438083|2022-05-30
hCoV-19/New_Zealand/22CH4607/2022|EPI_ISL_13438558|2022-05-31
hCoV-19/New_Zealand/22CH4608/2022|EPI_ISL_13438907|2022-05-31
hCoV-19/New_Zealand/22CH4612/2022|EPI_ISL_13436937|2022-05-31
hCoV-19/New_Zealand/22CH4631/2022|EPI_ISL_13437511|2022-06-01
hCoV-19/New_Zealand/22CH4633/2022|EPI_ISL_13436739|2022-06-02
hCoV-19/New_Zealand/22CH4664/2022|EPI_ISL_13436774|2022-06-03
hCoV-19/New_Zealand/22CH4677/2022|EPI_ISL_13438679|2022-06-01
hCoV-19/New_Zealand/22CH4690/2022|EPI_ISL_13437784|2022-05-31
hCoV-19/New_Zealand/22CH4694/2022|EPI_ISL_13437653|2022-06-02
hCoV-19/New_Zealand/22CH4696/2022|EPI_ISL_13437687|2022-06-01
hCoV-19/New_Zealand/22CH4703/2022|EPI_ISL_13438533|2022-06-02
hCoV-19/New_Zealand/22CH4709/2022|EPI_ISL_13438809|2022-06-01
hCoV-19/New_Zealand/22CH4720/2022|EPI_ISL_13438744|2022-06-01
hCoV-19/New_Zealand/22CH4759/2022|EPI_ISL_13437029|2022-06-01
hCoV-19/New_Zealand/22CH4760/2022|EPI_ISL_13438046|2022-06-01
hCoV-19/New_Zealand/22CH4771/2022|EPI_ISL_13437411|2022-06-05
hCoV-19/New_Zealand/22CH4799/2022|EPI_ISL_13436962|2022-06-03
hCoV-19/New_Zealand/22CH4819/2022|EPI_ISL_13437036|2022-06-07
hCoV-19/New_Zealand/22CH4820/2022|EPI_ISL_13437465|2022-06-07
hCoV-19/New_Zealand/22CH4823/2022|EPI_ISL_13437624|2022-06-07
hCoV-19/New_Zealand/22CH4859/2022|EPI_ISL_13436676|2022-06-06
hCoV-19/New_Zealand/22CH4947/2022|EPI_ISL_13437780|2022-06-10
hCoV-19/New_Zealand/22CH4992/2022|EPI_ISL_13436952|2022-06-08
hCoV-19/New_Zealand/22CH4995/2022|EPI_ISL_13438142|2022-06-08
hCoV-19/New_Zealand/22CH5020/2022|EPI_ISL_13437935|2022-06-08
hCoV-19/New_Zealand/22CH5071/2022|EPI_ISL_13437668|2022-06-09
hCoV-19/New_Zealand/22CH5085/2022|EPI_ISL_13437479|2022-06-16
hCoV-19/New_Zealand/22CH5158/2022|EPI_ISL_13437510|2022-06-14
hCoV-19/New_Zealand/22CH5350/2022|EPI_ISL_13763820|2022-06-17
hCoV-19/New_Zealand/22CH5409/2022|EPI_ISL_13765195|2022-06-21
hCoV-19/New_Zealand/22CH5448/2022|EPI_ISL_13765445|2022-06-22
hCoV-19/New_Zealand/22CH5487/2022|EPI_ISL_13765037|2022-06-24
hCoV-19/New_Zealand/22CH5522/2022|EPI_ISL_13764379|2022-06-22
hCoV-19/New_Zealand/22CH5524/2022|EPI_ISL_13765184|2022-06-23

hCoV-19/New_Zealand/22CH5540/2022|EPI_ISL_13764097|2022-06-23
hCoV-19/New_Zealand/22CH5602/2022|EPI_ISL_13764588|2022-06-27
hCoV-19/New_Zealand/22CH5605/2022|EPI_ISL_13764935|2022-06-22
hCoV-19/New_Zealand/22CH5612/2022|EPI_ISL_13765488|2022-06-22
hCoV-19/New_Zealand/22CH5615/2022|EPI_ISL_13765485|2022-06-22
hCoV-19/New_Zealand/22CH5622/2022|EPI_ISL_13765374|2022-06-23
hCoV-19/New_Zealand/22CH5642/2022|EPI_ISL_13765193|2022-06-25
hCoV-19/New_Zealand/22CH5652/2022|EPI_ISL_13765300|2022-06-26
hCoV-19/New_Zealand/22CH5662/2022|EPI_ISL_13764657|2022-06-23
hCoV-19/New_Zealand/22CH5705/2022|EPI_ISL_13764091|2022-06-29
hCoV-19/New_Zealand/22CH5773/2022|EPI_ISL_13763644|2022-06-29
hCoV-19/New_Zealand/22CH5792/2022|EPI_ISL_13765052|2022-06-28
hCoV-19/New_Zealand/22CH5793/2022|EPI_ISL_13765238|2022-06-27
hCoV-19/New_Zealand/22CH5837/2022|EPI_ISL_13763983|2022-06-30
hCoV-19/New_Zealand/22CH5876/2022|EPI_ISL_13765434|2022-07-01
hCoV-19/New_Zealand/22CH5961/2022|EPI_ISL_13764918|2022-07-03
hCoV-19/New_Zealand/22CH5986/2022|EPI_ISL_13765546|2022-07-04
hCoV-19/New_Zealand/22CH5988/2022|EPI_ISL_13765540|2022-07-04
hCoV-19/New_Zealand/22CH5997/2022|EPI_ISL_13764221|2022-07-05
hCoV-19/New_Zealand/22CH6042/2022|EPI_ISL_13765293|2022-07-01
hCoV-19/New_Zealand/22CH6050/2022|EPI_ISL_13763683|2022-07-04
hCoV-19/New_Zealand/22CH6119/2022|EPI_ISL_13763761|2022-07-05
hCoV-19/New_Zealand/22CH6245/2022|EPI_ISL_13765361|2022-07-06
hCoV-19/New_Zealand/22CH6247/2022|EPI_ISL_13764632|2022-07-06
hCoV-19/New_Zealand/22CH6251/2022|EPI_ISL_13763951|2022-07-06
hCoV-19/New_Zealand/22CH6286/2022|EPI_ISL_13765211|2022-07-06
hCoV-19/New_Zealand/22CH6293/2022|EPI_ISL_13764115|2022-07-06
hCoV-19/New_Zealand/22CH6329/2022|EPI_ISL_13763869|2022-07-07
hCoV-19/New_Zealand/22CV0106/2022|EPI_ISL_8648571|2022-01-03
hCoV-19/New_Zealand/22CV0109/2022|EPI_ISL_8648657|2022-01-03
hCoV-19/New_Zealand/22CV0134/2022|EPI_ISL_8825504|2022-01-04
hCoV-19/New_Zealand/22CV0140/2022|EPI_ISL_8826292|2022-01-04
hCoV-19/New_Zealand/22CV0155/2022|EPI_ISL_8826181|2022-01-04
hCoV-19/New_Zealand/22CV0159/2022|EPI_ISL_8826131|2022-01-04
hCoV-19/New_Zealand/22CV0252/2022|EPI_ISL_8826351|2022-01-05
hCoV-19/New_Zealand/22CV0326/2022|EPI_ISL_8648753|2022-01-07
hCoV-19/New_Zealand/22CV0373/2022|EPI_ISL_8826061|2022-01-07
hCoV-19/New_Zealand/22CV0377/2022|EPI_ISL_8825499|2022-01-08
hCoV-19/New_Zealand/22CV0379/2022|EPI_ISL_8825478|2022-01-08
hCoV-19/New_Zealand/22CV0383/2022|EPI_ISL_8825482|2022-01-08
hCoV-19/New_Zealand/22CV0417/2022|EPI_ISL_8826200|2022-01-10
hCoV-19/New_Zealand/22CV0444/2022|EPI_ISL_8825508|2022-01-12
hCoV-19/New_Zealand/22CV0468/2022|EPI_ISL_8826155|2022-01-13

hCoV-19/New_Zealand/22CV0476/2022|EPI_ISL_8826366|2022-01-13
hCoV-19/New_Zealand/22CV0503/2022|EPI_ISL_9209489|2022-01-14
hCoV-19/New_Zealand/22CV0512/2022|EPI_ISL_9210796|2022-01-16
hCoV-19/New_Zealand/22CV0518/2022|EPI_ISL_9209384|2022-01-16
hCoV-19/New_Zealand/22CV0522/2022|EPI_ISL_9210513|2022-01-16
hCoV-19/New_Zealand/22CV0534/2022|EPI_ISL_9210841|2022-01-18
hCoV-19/New_Zealand/22CV0543/2022|EPI_ISL_9210857|2022-01-18
hCoV-19/New_Zealand/22CV0633/2022|EPI_ISL_9210335|2022-01-14
hCoV-19/New_Zealand/22CV0635/2022|EPI_ISL_9209666|2022-01-16
hCoV-19/New_Zealand/22CV0637/2022|EPI_ISL_9210258|2022-01-16
hCoV-19/New_Zealand/22CV0638/2022|EPI_ISL_9209993|2022-01-17
hCoV-19/New_Zealand/22CV0646/2022|EPI_ISL_9210410|2022-01-19
hCoV-19/New_Zealand/22CV0745/2022|EPI_ISL_9209190|2022-01-21
hCoV-19/New_Zealand/22CV0759/2022|EPI_ISL_9210632|2022-01-18
hCoV-19/New_Zealand/22CV0776/2022|EPI_ISL_9434120|2022-01-23
hCoV-19/New_Zealand/22CV0791/2022|EPI_ISL_9434143|2022-01-23
hCoV-19/New_Zealand/22CV0801/2022|EPI_ISL_9434059|2022-01-23
hCoV-19/New_Zealand/22CV0814/2022|EPI_ISL_9209452|2022-01-21
hCoV-19/New_Zealand/22CV0835/2022|EPI_ISL_9434101|2022-01-24
hCoV-19/New_Zealand/22CV0843/2022|EPI_ISL_9433957|2022-01-24
hCoV-19/New_Zealand/22CV0888/2022|EPI_ISL_10188600|2022-01-26
hCoV-19/New_Zealand/22CV0905/2022|EPI_ISL_9636514|2022-01-24
hCoV-19/New_Zealand/22CV0922/2022|EPI_ISL_10188577|2022-01-26
hCoV-19/New_Zealand/22CV0932/2022|EPI_ISL_9636533|2022-01-25
hCoV-19/New_Zealand/22CV0957/2022|EPI_ISL_9636560|2022-01-26
hCoV-19/New_Zealand/22CV0958/2022|EPI_ISL_9636655|2022-01-26
hCoV-19/New_Zealand/22CV0990/2022|EPI_ISL_9636580|2022-01-26
hCoV-19/New_Zealand/22CV1002/2022|EPI_ISL_9636613|2022-01-26
hCoV-19/New_Zealand/22CV1037/2022|EPI_ISL_10188175|2022-01-27
hCoV-19/New_Zealand/22CV1057/2022|EPI_ISL_9636586|2022-01-27
hCoV-19/New_Zealand/22CV1089/2022|EPI_ISL_9636512|2022-01-26
hCoV-19/New_Zealand/22CV1103/2022|EPI_ISL_9433830|2022-01-29
hCoV-19/New_Zealand/22CV1105/2022|EPI_ISL_9434039|2022-01-29
hCoV-19/New_Zealand/22CV1132/2022|EPI_ISL_9636594|2022-01-27
hCoV-19/New_Zealand/22CV1136/2022|EPI_ISL_9636646|2022-01-27
hCoV-19/New_Zealand/22CV1147/2022|EPI_ISL_10188166|2022-01-28
hCoV-19/New_Zealand/22CV1149/2022|EPI_ISL_9636671|2022-01-28
hCoV-19/New_Zealand/22CV1190/2022|EPI_ISL_9636505|2022-01-28
hCoV-19/New_Zealand/22CV1289/2022|EPI_ISL_9636508|2022-01-28
hCoV-19/New_Zealand/22CV1295/2022|EPI_ISL_9636552|2022-01-28
hCoV-19/New_Zealand/22CV1319/2022|EPI_ISL_10188745|2022-01-29
hCoV-19/New_Zealand/22CV1386/2022|EPI_ISL_10188737|2022-01-27
hCoV-19/New_Zealand/22CV1408/2022|EPI_ISL_10188602|2022-01-29

hCoV-19/New_Zealand/22CV1417/2022|EPI_ISL_10188362|2022-01-29
hCoV-19/New_Zealand/22CV1429/2022|EPI_ISL_10188402|2022-01-30
hCoV-19/New_Zealand/22CV1432/2022|EPI_ISL_10187979|2022-01-30
hCoV-19/New_Zealand/22CV1435/2022|EPI_ISL_10187706|2022-01-31
hCoV-19/New_Zealand/22CV1443/2022|EPI_ISL_10188047|2022-01-31
hCoV-19/New_Zealand/22CV1452/2022|EPI_ISL_10187909|2022-01-31
hCoV-19/New_Zealand/22CV1467/2022|EPI_ISL_10188400|2022-01-31
hCoV-19/New_Zealand/22CV1485/2022|EPI_ISL_10188511|2022-01-31
hCoV-19/New_Zealand/22CV1515/2022|EPI_ISL_10188365|2022-02-01
hCoV-19/New_Zealand/22CV1530/2022|EPI_ISL_10188354|2022-02-01
hCoV-19/New_Zealand/22CV1534/2022|EPI_ISL_10187683|2022-02-01
hCoV-19/New_Zealand/22CV1543/2022|EPI_ISL_10188383|2022-02-01
hCoV-19/New_Zealand/22CV1553/2022|EPI_ISL_10188081|2022-01-31
hCoV-19/New_Zealand/22CV1563/2022|EPI_ISL_10188413|2022-02-02
hCoV-19/New_Zealand/22CV1623/2022|EPI_ISL_10188455|2022-02-03
hCoV-19/New_Zealand/22CV1629/2022|EPI_ISL_10188252|2022-02-03
hCoV-19/New_Zealand/22CV1637/2022|EPI_ISL_10188556|2022-02-05
hCoV-19/New_Zealand/22CV1639/2022|EPI_ISL_10188690|2022-02-05
hCoV-19/New_Zealand/22CV1648/2022|EPI_ISL_10188498|2022-02-05
hCoV-19/New_Zealand/22CV1663/2022|EPI_ISL_10188385|2022-02-05
hCoV-19/New_Zealand/22CV1680/2022|EPI_ISL_10188414|2022-02-06
hCoV-19/New_Zealand/22CV1683/2022|EPI_ISL_10187811|2022-02-07
hCoV-19/New_Zealand/22CV1695/2022|EPI_ISL_10188586|2022-02-06
hCoV-19/New_Zealand/22CV1703/2022|EPI_ISL_10188272|2022-02-05
hCoV-19/New_Zealand/22CV1754/2022|EPI_ISL_10188448|2022-02-07
hCoV-19/New_Zealand/22CV1765/2022|EPI_ISL_10187761|2022-02-07
hCoV-19/New_Zealand/22CV1773/2022|EPI_ISL_10187643|2022-02-08
hCoV-19/New_Zealand/22CV1775/2022|EPI_ISL_10188352|2022-02-08
hCoV-19/New_Zealand/22CV1788/2022|EPI_ISL_10188415|2022-02-08
hCoV-19/New_Zealand/22CV1794/2022|EPI_ISL_10187698|2022-02-09
hCoV-19/New_Zealand/22CV1804/2022|EPI_ISL_10187967|2022-02-09
hCoV-19/New_Zealand/22CV1816/2022|EPI_ISL_10188440|2022-02-08
hCoV-19/New_Zealand/22CV1826/2022|EPI_ISL_10188350|2022-02-09
hCoV-19/New_Zealand/22CV1830/2022|EPI_ISL_10188444|2022-02-09
hCoV-19/New_Zealand/22CV1835/2022|EPI_ISL_10188375|2022-02-09
hCoV-19/New_Zealand/22CV1842/2022|EPI_ISL_10188090|2022-02-09
hCoV-19/New_Zealand/22CV1861/2022|EPI_ISL_10188094|2022-02-06
hCoV-19/New_Zealand/22CV1871/2022|EPI_ISL_10187869|2022-02-10
hCoV-19/New_Zealand/22CV1880/2022|EPI_ISL_10188051|2022-02-10
hCoV-19/New_Zealand/22CV1882/2022|EPI_ISL_10188293|2022-02-10
hCoV-19/New_Zealand/22CV1909/2022|EPI_ISL_10187837|2022-02-09
hCoV-19/New_Zealand/22CV1928/2022|EPI_ISL_10188348|2022-02-11
hCoV-19/New_Zealand/22CV1963/2022|EPI_ISL_11865832|2022-02-12

hCoV-19/New_Zealand/22CV1967/2022|EPI_ISL_11865925|2022-02-12
hCoV-19/New_Zealand/22CV1974/2022|EPI_ISL_10188482|2022-02-14
hCoV-19/New_Zealand/22CV2002/2022|EPI_ISL_10188335|2022-02-15
hCoV-19/New_Zealand/22CV2004/2022|EPI_ISL_10626210|2022-02-15
hCoV-19/New_Zealand/22CV2012/2022|EPI_ISL_10626334|2022-02-14
hCoV-19/New_Zealand/22CV2031/2022|EPI_ISL_10188546|2022-02-14
hCoV-19/New_Zealand/22CV2052/2022|EPI_ISL_10188452|2022-02-13
hCoV-19/New_Zealand/22CV2072/2022|EPI_ISL_10188103|2022-02-14
hCoV-19/New_Zealand/22CV2089/2022|EPI_ISL_10186720|2022-02-14
hCoV-19/New_Zealand/22CV2121/2022|EPI_ISL_10626202|2022-02-15
hCoV-19/New_Zealand/22CV2204/2022|EPI_ISL_10626542|2022-02-13
hCoV-19/New_Zealand/22CV2238/2022|EPI_ISL_10626197|2022-02-16
hCoV-19/New_Zealand/22CV2243/2022|EPI_ISL_10626385|2022-02-16
hCoV-19/New_Zealand/22CV2253/2022|EPI_ISL_10626040|2022-02-18
hCoV-19/New_Zealand/22CV2269/2022|EPI_ISL_10626421|2022-02-18
hCoV-19/New_Zealand/22CV2274/2022|EPI_ISL_10626406|2022-02-18
hCoV-19/New_Zealand/22CV2278/2022|EPI_ISL_10626362|2022-02-18
hCoV-19/New_Zealand/22CV2288/2022|EPI_ISL_10626410|2022-02-18
hCoV-19/New_Zealand/22CV2298/2022|EPI_ISL_10626390|2022-02-17
hCoV-19/New_Zealand/22CV2309/2022|EPI_ISL_10626166|2022-02-18
hCoV-19/New_Zealand/22CV2384/2022|EPI_ISL_10626277|2022-02-21
hCoV-19/New_Zealand/22CV2394/2022|EPI_ISL_10626446|2022-02-22
hCoV-19/New_Zealand/22CV2405/2022|EPI_ISL_10626072|2022-02-21
hCoV-19/New_Zealand/22CV2411/2022|EPI_ISL_10626293|2022-02-21
hCoV-19/New_Zealand/22CV2451/2022|EPI_ISL_10626497|2022-02-22
hCoV-19/New_Zealand/22CV2466/2022|EPI_ISL_11017929|2022-02-23
hCoV-19/New_Zealand/22CV2478/2022|EPI_ISL_11017920|2022-02-23
hCoV-19/New_Zealand/22CV2484/2022|EPI_ISL_11017684|2022-02-23
hCoV-19/New_Zealand/22CV2501/2022|EPI_ISL_11017695|2022-02-25
hCoV-19/New_Zealand/22CV2503/2022|EPI_ISL_11017839|2022-02-25
hCoV-19/New_Zealand/22CV2523/2022|EPI_ISL_11017838|2022-02-28
hCoV-19/New_Zealand/22CV2534/2022|EPI_ISL_11017765|2022-02-23
hCoV-19/New_Zealand/22CV2536/2022|EPI_ISL_11017864|2022-02-23
hCoV-19/New_Zealand/22CV2540/2022|EPI_ISL_11017872|2022-02-23
hCoV-19/New_Zealand/22CV2546/2022|EPI_ISL_11017738|2022-03-02
hCoV-19/New_Zealand/22CV2557/2022|EPI_ISL_11017666|2022-03-02
hCoV-19/New_Zealand/22CV2593/2022|EPI_ISL_11017921|2022-02-24
hCoV-19/New_Zealand/22CV2596/2022|EPI_ISL_11017591|2022-02-24
hCoV-19/New_Zealand/22CV2619/2022|EPI_ISL_11017606|2022-03-03
hCoV-19/New_Zealand/22CV2625/2022|EPI_ISL_11017806|2022-03-03
hCoV-19/New_Zealand/22CV2646/2022|EPI_ISL_11017724|2022-02-24
hCoV-19/New_Zealand/22CV2647/2022|EPI_ISL_11017731|2022-02-24
hCoV-19/New_Zealand/22CV2705/2022|EPI_ISL_11331345|2022-03-08

hCoV-19/New_Zealand/22CV2740/2022|EPI_ISL_11331658|2022-03-07
hCoV-19/New_Zealand/22CV2761/2022|EPI_ISL_11331510|2022-03-08
hCoV-19/New_Zealand/22CV2777/2022|EPI_ISL_11331355|2022-03-09
hCoV-19/New_Zealand/22CV2787/2022|EPI_ISL_11331382|2022-03-10
hCoV-19/New_Zealand/22CV2795/2022|EPI_ISL_11331482|2022-03-10
hCoV-19/New_Zealand/22CV2803/2022|EPI_ISL_11331563|2022-03-11
hCoV-19/New_Zealand/22CV2812/2022|EPI_ISL_11331335|2022-03-13
hCoV-19/New_Zealand/22CV2826/2022|EPI_ISL_11331595|2022-03-12
hCoV-19/New_Zealand/22CV2847/2022|EPI_ISL_11331420|2022-03-11
hCoV-19/New_Zealand/22CV2858/2022|EPI_ISL_11331731|2022-03-14
hCoV-19/New_Zealand/22CV2865/2022|EPI_ISL_11331614|2022-03-04
hCoV-19/New_Zealand/22CV2882/2022|EPI_ISL_11331530|2022-03-06
hCoV-19/New_Zealand/22CV2885/2022|EPI_ISL_11331591|2022-03-03
hCoV-19/New_Zealand/22CV2902/2022|EPI_ISL_11331385|2022-03-06
hCoV-19/New_Zealand/22CV2904/2022|EPI_ISL_11331846|2022-03-06
hCoV-19/New_Zealand/22CV2906/2022|EPI_ISL_11331588|2022-03-07
hCoV-19/New_Zealand/22CV2934/2022|EPI_ISL_11331637|2022-03-09
hCoV-19/New_Zealand/22CV2936/2022|EPI_ISL_11331737|2022-03-10
hCoV-19/New_Zealand/22CV2998/2022|EPI_ISL_11331322|2022-03-16
hCoV-19/New_Zealand/22CV3028/2022|EPI_ISL_11331366|2022-03-17
hCoV-19/New_Zealand/22CV3033/2022|EPI_ISL_11331643|2022-03-18
hCoV-19/New_Zealand/22CV3042/2022|EPI_ISL_11331727|2022-03-08
hCoV-19/New_Zealand/22CV3046/2022|EPI_ISL_11331441|2022-03-07
hCoV-19/New_Zealand/22CV3052/2022|EPI_ISL_11331361|2022-03-17
hCoV-19/New_Zealand/22CV3059/2022|EPI_ISL_11865927|2022-03-17
hCoV-19/New_Zealand/22CV3064/2022|EPI_ISL_11866135|2022-03-20
hCoV-19/New_Zealand/22CV3073/2022|EPI_ISL_11866184|2022-03-20
hCoV-19/New_Zealand/22CV3074/2022|EPI_ISL_11866277|2022-03-20
hCoV-19/New_Zealand/22CV3076/2022|EPI_ISL_11865844|2022-03-21
hCoV-19/New_Zealand/22CV3080/2022|EPI_ISL_11331605|2022-03-21
hCoV-19/New_Zealand/22CV3082/2022|EPI_ISL_11331592|2022-03-21
hCoV-19/New_Zealand/22CV3096/2022|EPI_ISL_11865872|2022-03-18
hCoV-19/New_Zealand/22CV3100/2022|EPI_ISL_11865653|2022-03-16
hCoV-19/New_Zealand/22CV3103/2022|EPI_ISL_11865830|2022-03-19
hCoV-19/New_Zealand/22CV3112/2022|EPI_ISL_11866107|2022-03-08
hCoV-19/New_Zealand/22CV3205/2022|EPI_ISL_11865960|2022-03-21
hCoV-19/New_Zealand/22CV3208/2022|EPI_ISL_11866081|2022-03-21
hCoV-19/New_Zealand/22CV3213/2022|EPI_ISL_11866060|2022-02-20
hCoV-19/New_Zealand/22CV3225/2022|EPI_ISL_11865721|2022-02-26
hCoV-19/New_Zealand/22CV3227/2022|EPI_ISL_11865912|2022-03-05
hCoV-19/New_Zealand/22CV3239/2022|EPI_ISL_11865939|2022-03-23
hCoV-19/New_Zealand/22CV3265/2022|EPI_ISL_11865768|2022-03-24
hCoV-19/New_Zealand/22CV3277/2022|EPI_ISL_11866119|2022-03-19

hCoV-19/New_Zealand/22CV3300/2022|EPI_ISL_11866099|2022-03-18
hCoV-19/New_Zealand/22CV3303/2022|EPI_ISL_11865675|2022-03-23
hCoV-19/New_Zealand/22CV3307/2022|EPI_ISL_11866045|2022-02-23
hCoV-19/New_Zealand/22CV3314/2022|EPI_ISL_11866121|2022-03-02
hCoV-19/New_Zealand/22CV3324/2022|EPI_ISL_11866055|2022-03-19
hCoV-19/New_Zealand/22CV3326/2022|EPI_ISL_11866013|2022-03-01
hCoV-19/New_Zealand/22CV3327/2022|EPI_ISL_11865881|2022-03-03
hCoV-19/New_Zealand/22CV3328/2022|EPI_ISL_11865937|2022-03-15
hCoV-19/New_Zealand/22CV3330/2022|EPI_ISL_11865736|2022-03-19
hCoV-19/New_Zealand/22CV3335/2022|EPI_ISL_11865886|2022-03-26
hCoV-19/New_Zealand/22CV3359/2022|EPI_ISL_11865902|2022-03-26
hCoV-19/New_Zealand/22CV3387/2022|EPI_ISL_11866136|2022-03-18
hCoV-19/New_Zealand/22CV3388/2022|EPI_ISL_11865976|2022-03-17
hCoV-19/New_Zealand/22CV3392/2022|EPI_ISL_11865910|2022-02-28
hCoV-19/New_Zealand/22CV3429/2022|EPI_ISL_11865826|2022-03-15
hCoV-19/New_Zealand/22CV3446/2022|EPI_ISL_11866147|2022-03-28
hCoV-19/New_Zealand/22CV3449/2022|EPI_ISL_12545335|2022-03-29
hCoV-19/New_Zealand/22CV3458/2022|EPI_ISL_11866015|2022-03-24
hCoV-19/New_Zealand/22CV3477/2022|EPI_ISL_11866108|2022-03-10
hCoV-19/New_Zealand/22CV3482/2022|EPI_ISL_11865903|2022-03-21
hCoV-19/New_Zealand/22CV3506/2022|EPI_ISL_11865978|2022-04-02
hCoV-19/New_Zealand/22CV3508/2022|EPI_ISL_11865900|2022-04-01
hCoV-19/New_Zealand/22CV3525/2022|EPI_ISL_11865921|2022-03-31
hCoV-19/New_Zealand/22CV3526/2022|EPI_ISL_11865940|2022-04-01
hCoV-19/New_Zealand/22CV3538/2022|EPI_ISL_12545484|2022-03-25
hCoV-19/New_Zealand/22CV3569/2022|EPI_ISL_12544954|2022-03-25
hCoV-19/New_Zealand/22CV3590/2022|EPI_ISL_12545822|2022-04-05
hCoV-19/New_Zealand/22CV3595/2022|EPI_ISL_12545184|2022-04-07
hCoV-19/New_Zealand/22CV3600/2022|EPI_ISL_12545426|2022-04-05
hCoV-19/New_Zealand/22CV3607/2022|EPI_ISL_12546044|2022-04-05
hCoV-19/New_Zealand/22CV3608/2022|EPI_ISL_12545258|2022-04-05
hCoV-19/New_Zealand/22CV3610/2022|EPI_ISL_12545174|2022-04-04
hCoV-19/New_Zealand/22CV3640/2022|EPI_ISL_12546104|2022-04-05
hCoV-19/New_Zealand/22CV3643/2022|EPI_ISL_12545645|2022-04-07
hCoV-19/New_Zealand/22CV3645/2022|EPI_ISL_12546021|2022-04-06
hCoV-19/New_Zealand/22CV3681/2022|EPI_ISL_12545584|2022-04-06
hCoV-19/New_Zealand/22CV3701/2022|EPI_ISL_12545723|2022-04-08
hCoV-19/New_Zealand/22CV3703/2022|EPI_ISL_12546234|2022-04-02
hCoV-19/New_Zealand/22CV3733/2022|EPI_ISL_12545052|2022-03-19
hCoV-19/New_Zealand/22CV3740/2022|EPI_ISL_12546207|2022-04-08
hCoV-19/New_Zealand/22CV3742/2022|EPI_ISL_12545756|2022-04-09
hCoV-19/New_Zealand/22CV3750/2022|EPI_ISL_12545552|2022-04-10
hCoV-19/New_Zealand/22CV3758/2022|EPI_ISL_12545178|2022-03-18

hCoV-19/New_Zealand/22CV3764/2022|EPI_ISL_12545819|2022-04-12
hCoV-19/New_Zealand/22CV3778/2022|EPI_ISL_12545022|2022-04-08
hCoV-19/New_Zealand/22CV3809/2022|EPI_ISL_12545925|2022-03-08
hCoV-19/New_Zealand/22CV3818/2022|EPI_ISL_12545362|2022-04-06
hCoV-19/New_Zealand/22CV3823/2022|EPI_ISL_12546477|2022-04-04
hCoV-19/New_Zealand/22CV3828/2022|EPI_ISL_12546332|2022-04-08
hCoV-19/New_Zealand/22CV3832/2022|EPI_ISL_12545694|2022-04-11
hCoV-19/New_Zealand/22CV3834/2022|EPI_ISL_12545867|2022-04-14
hCoV-19/New_Zealand/22CV3845/2022|EPI_ISL_12546366|2022-04-13
hCoV-19/New_Zealand/22CV3852/2022|EPI_ISL_12545092|2022-04-15
hCoV-19/New_Zealand/22CV3876/2022|EPI_ISL_12545956|2022-04-18
hCoV-19/New_Zealand/22CV3882/2022|EPI_ISL_12545501|2022-04-16
hCoV-19/New_Zealand/22CV3886/2022|EPI_ISL_12546061|2022-04-15
hCoV-19/New_Zealand/22CV3889/2022|EPI_ISL_12546499|2022-04-18
hCoV-19/New_Zealand/22CV3890/2022|EPI_ISL_12545260|2022-04-13
hCoV-19/New_Zealand/22CV3905/2022|EPI_ISL_12545795|2022-04-17
hCoV-19/New_Zealand/22CV3911/2022|EPI_ISL_12545982|2022-04-19
hCoV-19/New_Zealand/22CV3925/2022|EPI_ISL_12545833|2022-04-11
hCoV-19/New_Zealand/22CV3932/2022|EPI_ISL_12546115|2022-04-12
hCoV-19/New_Zealand/22CV3947/2022|EPI_ISL_12545602|2022-04-18
hCoV-19/New_Zealand/22CV3956/2022|EPI_ISL_12545397|2022-04-20
hCoV-19/New_Zealand/22CV3957/2022|EPI_ISL_12545281|2022-04-21
hCoV-19/New_Zealand/22CV3968/2022|EPI_ISL_12545917|2022-04-19
hCoV-19/New_Zealand/22CV3993/2022|EPI_ISL_12545154|2022-04-21
hCoV-19/New_Zealand/22CV3994/2022|EPI_ISL_12546186|2022-04-15
hCoV-19/New_Zealand/22CV3999/2022|EPI_ISL_12545312|2022-04-22
hCoV-19/New_Zealand/22CV4006/2022|EPI_ISL_12544953|2022-04-23
hCoV-19/New_Zealand/22CV4120/2022|EPI_ISL_12545053|2022-04-23
hCoV-19/New_Zealand/22CV4139/2022|EPI_ISL_12546166|2022-04-25
hCoV-19/New_Zealand/22CV4246/2022|EPI_ISL_12545574|2022-04-27
hCoV-19/New_Zealand/22CV4253/2022|EPI_ISL_12545964|2022-04-27
hCoV-19/New_Zealand/22CV4254/2022|EPI_ISL_12546235|2022-04-27
hCoV-19/New_Zealand/22CV4255/2022|EPI_ISL_12545265|2022-04-27
hCoV-19/New_Zealand/22CV4258/2022|EPI_ISL_12546339|2022-04-27
hCoV-19/New_Zealand/22CV4261/2022|EPI_ISL_12805588|2022-04-19
hCoV-19/New_Zealand/22CV4266/2022|EPI_ISL_13764975|2022-04-21
hCoV-19/New_Zealand/22CV4277/2022|EPI_ISL_12805534|2022-04-11
hCoV-19/New_Zealand/22CV4300/2022|EPI_ISL_12546150|2022-04-26
hCoV-19/New_Zealand/22CV4319/2022|EPI_ISL_12545430|2022-04-23
hCoV-19/New_Zealand/22CV4322/2022|EPI_ISL_12545139|2022-04-28
hCoV-19/New_Zealand/22CV4354/2022|EPI_ISL_12545113|2022-04-28
hCoV-19/New_Zealand/22CV4368/2022|EPI_ISL_12805666|2022-04-28
hCoV-19/New_Zealand/22CV4384/2022|EPI_ISL_12806159|2022-04-19

hCoV-19/New_Zealand/22CV4411/2022|EPI_ISL_12805875|2022-04-30
hCoV-19/New_Zealand/22CV4426/2022|EPI_ISL_12806217|2022-04-24
hCoV-19/New_Zealand/22CV4427/2022|EPI_ISL_12805780|2022-04-24
hCoV-19/New_Zealand/22CV4449/2022|EPI_ISL_12805949|2022-05-02
hCoV-19/New_Zealand/22CV4450/2022|EPI_ISL_12805914|2022-05-02
hCoV-19/New_Zealand/22CV4469/2022|EPI_ISL_12805831|2022-05-01
hCoV-19/New_Zealand/22CV4470/2022|EPI_ISL_12806005|2022-05-02
hCoV-19/New_Zealand/22CV4483/2022|EPI_ISL_12805818|2022-05-04
hCoV-19/New_Zealand/22CV4484/2022|EPI_ISL_12805693|2022-05-04
hCoV-19/New_Zealand/22CV4495/2022|EPI_ISL_12806210|2022-05-04
hCoV-19/New_Zealand/22CV4496/2022|EPI_ISL_12806174|2022-05-04
hCoV-19/New_Zealand/22CV4535/2022|EPI_ISL_12806272|2022-05-05
hCoV-19/New_Zealand/22CV4538/2022|EPI_ISL_12806239|2022-05-05
hCoV-19/New_Zealand/22CV4543/2022|EPI_ISL_12805913|2022-05-01
hCoV-19/New_Zealand/22CV4571/2022|EPI_ISL_12805473|2022-05-06
hCoV-19/New_Zealand/22CV4582/2022|EPI_ISL_12805495|2022-05-05
hCoV-19/New_Zealand/22CV4592/2022|EPI_ISL_12805726|2022-05-09
hCoV-19/New_Zealand/22CV4605/2022|EPI_ISL_12805433|2022-05-07
hCoV-19/New_Zealand/22CV4612/2022|EPI_ISL_12806117|2022-05-09
hCoV-19/New_Zealand/22CV4615/2022|EPI_ISL_12805430|2022-05-09
hCoV-19/New_Zealand/22CV4617/2022|EPI_ISL_12805768|2022-05-10
hCoV-19/New_Zealand/22CV4618/2022|EPI_ISL_12805970|2022-05-10
hCoV-19/New_Zealand/22CV4622/2022|EPI_ISL_12806076|2022-05-05
hCoV-19/New_Zealand/22CV4635/2022|EPI_ISL_12806256|2022-05-10
hCoV-19/New_Zealand/22CV4636/2022|EPI_ISL_12805835|2022-05-10
hCoV-19/New_Zealand/22CV4639/2022|EPI_ISL_12805578|2022-05-10
hCoV-19/New_Zealand/22CV4643/2022|EPI_ISL_12805906|2022-05-10
hCoV-19/New_Zealand/22CV4650/2022|EPI_ISL_12806197|2022-05-09
hCoV-19/New_Zealand/22CV4653/2022|EPI_ISL_12805506|2022-05-06
hCoV-19/New_Zealand/22CV4659/2022|EPI_ISL_12806049|2022-05-09
hCoV-19/New_Zealand/22CV4692/2022|EPI_ISL_12805971|2022-05-11
hCoV-19/New_Zealand/22CV4693/2022|EPI_ISL_12806204|2022-05-09
hCoV-19/New_Zealand/22CV4697/2022|EPI_ISL_12805454|2022-05-11
hCoV-19/New_Zealand/22CV4712/2022|EPI_ISL_12805940|2022-05-12
hCoV-19/New_Zealand/22CV4722/2022|EPI_ISL_12806156|2022-05-12
hCoV-19/New_Zealand/22CV4754/2022|EPI_ISL_13436868|2022-05-16
hCoV-19/New_Zealand/22CV4759/2022|EPI_ISL_13437063|2022-05-16
hCoV-19/New_Zealand/22CV4761/2022|EPI_ISL_13436822|2022-05-16
hCoV-19/New_Zealand/22CV4765/2022|EPI_ISL_13438519|2022-05-12
hCoV-19/New_Zealand/22CV4771/2022|EPI_ISL_13438968|2022-05-16
hCoV-19/New_Zealand/22CV4780/2022|EPI_ISL_13438683|2022-05-14
hCoV-19/New_Zealand/22CV4784/2022|EPI_ISL_13437683|2022-05-13
hCoV-19/New_Zealand/22CV4789/2022|EPI_ISL_13437099|2022-05-13

hCoV-19/New_Zealand/22CV4793/2022|EPI_ISL_13437225|2022-05-13
hCoV-19/New_Zealand/22CV4799/2022|EPI_ISL_13437024|2022-05-15
hCoV-19/New_Zealand/22CV4857/2022|EPI_ISL_13437938|2022-05-15
hCoV-19/New_Zealand/22CV4866/2022|EPI_ISL_13437131|2022-05-17
hCoV-19/New_Zealand/22CV4870/2022|EPI_ISL_13437609|2022-05-18
hCoV-19/New_Zealand/22CV4874/2022|EPI_ISL_13438884|2022-05-18
hCoV-19/New_Zealand/22CV4877/2022|EPI_ISL_13436762|2022-05-18
hCoV-19/New_Zealand/22CV4879/2022|EPI_ISL_13437900|2022-05-18
hCoV-19/New_Zealand/22CV4889/2022|EPI_ISL_13438972|2022-05-18
hCoV-19/New_Zealand/22CV4891/2022|EPI_ISL_13437919|2022-05-18
hCoV-19/New_Zealand/22CV4896/2022|EPI_ISL_13437326|2022-05-17
hCoV-19/New_Zealand/22CV4899/2022|EPI_ISL_13438743|2022-05-18
hCoV-19/New_Zealand/22CV4904/2022|EPI_ISL_13437050|2022-05-19
hCoV-19/New_Zealand/22CV4905/2022|EPI_ISL_13437375|2022-05-19
hCoV-19/New_Zealand/22CV4924/2022|EPI_ISL_13437452|2022-05-17
hCoV-19/New_Zealand/22CV4931/2022|EPI_ISL_13438264|2022-05-19
hCoV-19/New_Zealand/22CV4932/2022|EPI_ISL_13437192|2022-05-19
hCoV-19/New_Zealand/22CV4935/2022|EPI_ISL_13437814|2022-05-19
hCoV-19/New_Zealand/22CV4941/2022|EPI_ISL_13438979|2022-05-17
hCoV-19/New_Zealand/22CV4979/2022|EPI_ISL_13438791|2022-05-21
hCoV-19/New_Zealand/22CV4986/2022|EPI_ISL_13438718|2022-05-23
hCoV-19/New_Zealand/22CV4988/2022|EPI_ISL_13437133|2022-05-23
hCoV-19/New_Zealand/22CV4993/2022|EPI_ISL_13438645|2022-05-23
hCoV-19/New_Zealand/22CV4999/2022|EPI_ISL_13437961|2022-05-22
hCoV-19/New_Zealand/22CV5007/2022|EPI_ISL_13436929|2022-05-23
hCoV-19/New_Zealand/22CV5032/2022|EPI_ISL_13437843|2022-05-24
hCoV-19/New_Zealand/22CV5037/2022|EPI_ISL_13437879|2022-05-25
hCoV-19/New_Zealand/22CV5038/2022|EPI_ISL_13438912|2022-05-24
hCoV-19/New_Zealand/22CV5042/2022|EPI_ISL_13438222|2022-05-25
hCoV-19/New_Zealand/22CV5053/2022|EPI_ISL_13437514|2022-05-25
hCoV-19/New_Zealand/22CV5061/2022|EPI_ISL_13437894|2022-05-23
hCoV-19/New_Zealand/22CV5077/2022|EPI_ISL_13437527|2022-05-25
hCoV-19/New_Zealand/22CV5081/2022|EPI_ISL_13436903|2022-05-25
hCoV-19/New_Zealand/22CV5083/2022|EPI_ISL_13438438|2022-05-25
hCoV-19/New_Zealand/22CV5087/2022|EPI_ISL_13438349|2022-05-26
hCoV-19/New_Zealand/22CV5110/2022|EPI_ISL_13438380|2022-05-23
hCoV-19/New_Zealand/22CV5114/2022|EPI_ISL_13437672|2022-05-25
hCoV-19/New_Zealand/22CV5118/2022|EPI_ISL_13437903|2022-05-25
hCoV-19/New_Zealand/22CV5145/2022|EPI_ISL_13437560|2022-05-28
hCoV-19/New_Zealand/22CV5158/2022|EPI_ISL_13438267|2022-05-29
hCoV-19/New_Zealand/22CV5172/2022|EPI_ISL_13438344|2022-05-30
hCoV-19/New_Zealand/22CV5178/2022|EPI_ISL_13437214|2022-05-30
hCoV-19/New_Zealand/22CV5179/2022|EPI_ISL_13438959|2022-05-30

hCoV-19/New_Zealand/22CV5187/2022|EPI_ISL_13438569|2022-05-31
hCoV-19/New_Zealand/22CV5198/2022|EPI_ISL_13438680|2022-05-22
hCoV-19/New_Zealand/22CV5204/2022|EPI_ISL_13438232|2022-05-27
hCoV-19/New_Zealand/22CV5216/2022|EPI_ISL_13437207|2022-05-30
hCoV-19/New_Zealand/22CV5225/2022|EPI_ISL_13438838|2022-05-26
hCoV-19/New_Zealand/22CV5232/2022|EPI_ISL_13437529|2022-05-30
hCoV-19/New_Zealand/22CV5242/2022|EPI_ISL_13437802|2022-05-30
hCoV-19/New_Zealand/22CV5253/2022|EPI_ISL_13438220|2022-05-31
hCoV-19/New_Zealand/22CV5256/2022|EPI_ISL_13438589|2022-05-31
hCoV-19/New_Zealand/22CV5284/2022|EPI_ISL_13437253|2022-06-03
hCoV-19/New_Zealand/22CV5293/2022|EPI_ISL_13438490|2022-06-04
hCoV-19/New_Zealand/22CV5322/2022|EPI_ISL_13436775|2022-06-04
hCoV-19/New_Zealand/22CV5331/2022|EPI_ISL_13438560|2022-06-07
hCoV-19/New_Zealand/22CV5356/2022|EPI_ISL_13437651|2022-06-07
hCoV-19/New_Zealand/22CV5365/2022|EPI_ISL_13437639|2022-06-05
hCoV-19/New_Zealand/22CV5388/2022|EPI_ISL_13437950|2022-06-06
hCoV-19/New_Zealand/22CV5400/2022|EPI_ISL_13439002|2022-06-02
hCoV-19/New_Zealand/22CV5403/2022|EPI_ISL_13438832|2022-05-31
hCoV-19/New_Zealand/22CV5456/2022|EPI_ISL_13437573|2022-06-08
hCoV-19/New_Zealand/22CV5460/2022|EPI_ISL_13438034|2022-06-08
hCoV-19/New_Zealand/22CV5520/2022|EPI_ISL_13438888|2022-06-09
hCoV-19/New_Zealand/22CV5543/2022|EPI_ISL_13437245|2022-06-11
hCoV-19/New_Zealand/22CV5547/2022|EPI_ISL_13437956|2022-06-11
hCoV-19/New_Zealand/22CV5618/2022|EPI_ISL_13438573|2022-06-10
hCoV-19/New_Zealand/22CV5657/2022|EPI_ISL_13765400|2022-06-11
hCoV-19/New_Zealand/22CV5663/2022|EPI_ISL_13438568|2022-06-12
hCoV-19/New_Zealand/22CV5693/2022|EPI_ISL_13436970|2022-06-11
hCoV-19/New_Zealand/22CV5759/2022|EPI_ISL_13436802|2022-06-13
hCoV-19/New_Zealand/22CV5781/2022|EPI_ISL_13765314|2022-06-13
hCoV-19/New_Zealand/22CV5802/2022|EPI_ISL_13438506|2022-06-14
hCoV-19/New_Zealand/22CV5825/2022|EPI_ISL_13437091|2022-06-13
hCoV-19/New_Zealand/22CV5833/2022|EPI_ISL_13438191|2022-06-08
hCoV-19/New_Zealand/22CV5952/2022|EPI_ISL_13764814|2022-06-14
hCoV-19/New_Zealand/22CV5960/2022|EPI_ISL_13763899|2022-06-14
hCoV-19/New_Zealand/22CV6033/2022|EPI_ISL_13764402|2022-06-16
hCoV-19/New_Zealand/22CV6080/2022|EPI_ISL_13764655|2022-06-14
hCoV-19/New_Zealand/22CV6093/2022|EPI_ISL_13764501|2022-06-14
hCoV-19/New_Zealand/22CV6136/2022|EPI_ISL_13763742|2022-06-17
hCoV-19/New_Zealand/22CV6162/2022|EPI_ISL_13763846|2022-06-15
hCoV-19/New_Zealand/22CV6179/2022|EPI_ISL_13764220|2022-06-18
hCoV-19/New_Zealand/22CV6200/2022|EPI_ISL_13764903|2022-06-17
hCoV-19/New_Zealand/22CV6270/2022|EPI_ISL_13764817|2022-06-17
hCoV-19/New_Zealand/22CV6395/2022|EPI_ISL_13765272|2022-06-19

hCoV-19/New_Zealand/22CV6490/2022|EPI_ISL_13764187|2022-06-19
hCoV-19/New_Zealand/22CV6506/2022|EPI_ISL_13764058|2022-06-16
hCoV-19/New_Zealand/22CV6521/2022|EPI_ISL_13765342|2022-06-17
hCoV-19/New_Zealand/22CV6575/2022|EPI_ISL_13764821|2022-06-21
hCoV-19/New_Zealand/22CV6687/2022|EPI_ISL_13765308|2022-06-20
hCoV-19/New_Zealand/22CV6719/2022|EPI_ISL_13763806|2022-06-21
hCoV-19/New_Zealand/22CV6721/2022|EPI_ISL_13764387|2022-06-21
hCoV-19/New_Zealand/22CV6725/2022|EPI_ISL_13764947|2022-06-21
hCoV-19/New_Zealand/22CV6820/2022|EPI_ISL_13763745|2022-06-22
hCoV-19/New_Zealand/22CV6864/2022|EPI_ISL_13764847|2022-06-26
hCoV-19/New_Zealand/22CV6872/2022|EPI_ISL_13765068|2022-05-22
hCoV-19/New_Zealand/22CV6889/2022|EPI_ISL_13765170|2022-05-22
hCoV-19/New_Zealand/22CV6897/2022|EPI_ISL_13765060|2022-05-22
hCoV-19/New_Zealand/22CV6901/2022|EPI_ISL_13764179|2022-05-22
hCoV-19/New_Zealand/22CV6921/2022|EPI_ISL_13765482|2022-05-22
hCoV-19/New_Zealand/22CV6922/2022|EPI_ISL_13764289|2022-05-22
hCoV-19/New_Zealand/22CV6993/2022|EPI_ISL_13763977|2022-06-21
hCoV-19/New_Zealand/22CV6996/2022|EPI_ISL_13763787|2022-06-21
hCoV-19/New_Zealand/22CV7003/2022|EPI_ISL_13764649|2022-06-22
hCoV-19/New_Zealand/22CV7065/2022|EPI_ISL_13764279|2022-06-26
hCoV-19/New_Zealand/22CV7096/2022|EPI_ISL_13764923|2022-06-23
hCoV-19/New_Zealand/22CV7154/2022|EPI_ISL_13764393|2022-06-27
hCoV-19/New_Zealand/22CV7248/2022|EPI_ISL_13764241|2022-06-28
hCoV-19/New_Zealand/22CV7262/2022|EPI_ISL_13764270|2022-06-22
hCoV-19/New_Zealand/22CV7274/2022|EPI_ISL_13765364|2022-06-23
hCoV-19/New_Zealand/22CV7345/2022|EPI_ISL_13765122|2022-06-25
hCoV-19/New_Zealand/22CV7420/2022|EPI_ISL_13763973|2022-06-26
hCoV-19/New_Zealand/22KV0020/2022|EPI_ISL_9210052|2022-01-17
hCoV-19/New_Zealand/22KV0026/2022|EPI_ISL_9209169|2022-01-17
hCoV-19/New_Zealand/22KV0042/2022|EPI_ISL_9210065|2022-01-16
hCoV-19/New_Zealand/22KV0090/2022|EPI_ISL_9636739|2022-01-19
hCoV-19/New_Zealand/22KV0095/2022|EPI_ISL_9636652|2022-01-19
hCoV-19/New_Zealand/22KV0155/2022|EPI_ISL_9636575|2022-01-19
hCoV-19/New_Zealand/22KV0161/2022|EPI_ISL_9671812|2022-01-19
hCoV-19/New_Zealand/22KV0218/2022|EPI_ISL_9636771|2022-01-21
hCoV-19/New_Zealand/22MV0061/2022|EPI_ISL_8648497|2022-01-01
hCoV-19/New_Zealand/22MV0065/2022|EPI_ISL_8648633|2022-01-01
hCoV-19/New_Zealand/22MV0068/2022|EPI_ISL_8648557|2022-01-01
hCoV-19/New_Zealand/22MV0075/2022|EPI_ISL_8648560|2022-01-02
hCoV-19/New_Zealand/22MV0076/2022|EPI_ISL_8648501|2022-01-02
hCoV-19/New_Zealand/22MV0086/2022|EPI_ISL_8648625|2022-01-02
hCoV-19/New_Zealand/22MV0106/2022|EPI_ISL_8648746|2022-01-01
hCoV-19/New_Zealand/22MV0107/2022|EPI_ISL_8648591|2022-01-01

hCoV-19/New_Zealand/22MV0122/2022|EPI_ISL_8648492|2022-01-04
hCoV-19/New_Zealand/22MV0216/2022|EPI_ISL_8826194|2022-01-05
hCoV-19/New_Zealand/22MV0222/2022|EPI_ISL_8648505|2022-01-06
hCoV-19/New_Zealand/22MV0226/2022|EPI_ISL_8648750|2022-01-03
hCoV-19/New_Zealand/22MV0276/2022|EPI_ISL_8826215|2022-01-05
hCoV-19/New_Zealand/22MV0300/2022|EPI_ISL_8826129|2022-01-07
hCoV-19/New_Zealand/22MV0335/2022|EPI_ISL_8826132|2022-01-10
hCoV-19/New_Zealand/22MV0337/2022|EPI_ISL_8825534|2022-01-10
hCoV-19/New_Zealand/22MV0339/2022|EPI_ISL_8826121|2022-01-10
hCoV-19/New_Zealand/22MV0344/2022|EPI_ISL_8825403|2022-01-07
hCoV-19/New_Zealand/22MV0348/2022|EPI_ISL_8825511|2022-01-05
hCoV-19/New_Zealand/22MV0382/2022|EPI_ISL_8826186|2022-01-10
hCoV-19/New_Zealand/22MV0442/2022|EPI_ISL_8825569|2022-01-11
hCoV-19/New_Zealand/22MV0447/2022|EPI_ISL_8825570|2022-01-12
hCoV-19/New_Zealand/22MV0483/2022|EPI_ISL_8826368|2022-01-09
hCoV-19/New_Zealand/22MV0488/2022|EPI_ISL_8825554|2022-01-09
hCoV-19/New_Zealand/22MV0532/2022|EPI_ISL_9209430|2022-01-18
hCoV-19/New_Zealand/22MV0555/2022|EPI_ISL_9209705|2022-01-18
hCoV-19/New_Zealand/22MV0567/2022|EPI_ISL_9209749|2022-01-22
hCoV-19/New_Zealand/22MV0572/2022|EPI_ISL_9209909|2022-01-20
hCoV-19/New_Zealand/22MV0575/2022|EPI_ISL_9210440|2022-01-20
hCoV-19/New_Zealand/22MV0583/2022|EPI_ISL_9210183|2022-01-21
hCoV-19/New_Zealand/22MV0586/2022|EPI_ISL_9210613|2022-01-18
hCoV-19/New_Zealand/22MV0597/2022|EPI_ISL_9210700|2022-01-19
hCoV-19/New_Zealand/22MV0624/2022|EPI_ISL_9210013|2022-01-24
hCoV-19/New_Zealand/22MV0630/2022|EPI_ISL_9210079|2022-01-25
hCoV-19/New_Zealand/22MV0670/2022|EPI_ISL_9433986|2022-01-26
hCoV-19/New_Zealand/22MV0672/2022|EPI_ISL_9434080|2022-01-27
hCoV-19/New_Zealand/22MV0694/2022|EPI_ISL_9434034|2022-01-28
hCoV-19/New_Zealand/22MV0700/2022|EPI_ISL_9433903|2022-01-28
hCoV-19/New_Zealand/22MV0770/2022|EPI_ISL_10188416|2022-02-01
hCoV-19/New_Zealand/22MV0779/2022|EPI_ISL_10188601|2022-02-02
hCoV-19/New_Zealand/22MV0785/2022|EPI_ISL_10188283|2022-02-02
hCoV-19/New_Zealand/22MV0796/2022|EPI_ISL_10188516|2022-02-02
hCoV-19/New_Zealand/22MV0797/2022|EPI_ISL_10188481|2022-02-02
hCoV-19/New_Zealand/22MV0832/2022|EPI_ISL_10188549|2022-02-02
hCoV-19/New_Zealand/22MV0836/2022|EPI_ISL_10188617|2022-02-03
hCoV-19/New_Zealand/22MV0840/2022|EPI_ISL_10188427|2022-02-08
hCoV-19/New_Zealand/22MV0851/2022|EPI_ISL_10188692|2022-02-09
hCoV-19/New_Zealand/22MV0861/2022|EPI_ISL_10188261|2022-02-09
hCoV-19/New_Zealand/22MV0870/2022|EPI_ISL_10187822|2022-02-10
hCoV-19/New_Zealand/22MV0872/2022|EPI_ISL_10188432|2022-02-09
hCoV-19/New_Zealand/22MV0886/2022|EPI_ISL_10187938|2022-02-10

hCoV-19/New_Zealand/22MV0888/2022|EPI_ISL_10188080|2022-02-10
hCoV-19/New_Zealand/22MV0890/2022|EPI_ISL_10188735|2022-02-10
hCoV-19/New_Zealand/22MV0897/2022|EPI_ISL_10187744|2022-02-16
hCoV-19/New_Zealand/22MV0940/2022|EPI_ISL_10626493|2022-02-16
hCoV-19/New_Zealand/22MV0959/2022|EPI_ISL_10626043|2022-02-15
hCoV-19/New_Zealand/22MV0962/2022|EPI_ISL_10626339|2022-02-17
hCoV-19/New_Zealand/22MV0967/2022|EPI_ISL_10626393|2022-02-15
hCoV-19/New_Zealand/22MV1003/2022|EPI_ISL_10626101|2022-02-19
hCoV-19/New_Zealand/22MV1017/2022|EPI_ISL_10626273|2022-02-20
hCoV-19/New_Zealand/22MV1027/2022|EPI_ISL_10626094|2022-02-21
hCoV-19/New_Zealand/22MV1050/2022|EPI_ISL_10626486|2022-02-22
hCoV-19/New_Zealand/22MV1057/2022|EPI_ISL_10626196|2022-02-15
hCoV-19/New_Zealand/22MV1059/2022|EPI_ISL_10626195|2022-02-18
hCoV-19/New_Zealand/22MV1068/2022|EPI_ISL_10626095|2022-02-20
hCoV-19/New_Zealand/22MV1075/2022|EPI_ISL_10626414|2022-02-21
hCoV-19/New_Zealand/22MV1076/2022|EPI_ISL_11017719|2022-02-28
hCoV-19/New_Zealand/22MV1101/2022|EPI_ISL_11017613|2022-02-26
hCoV-19/New_Zealand/22MV1125/2022|EPI_ISL_11017586|2022-03-01
hCoV-19/New_Zealand/22MV1130/2022|EPI_ISL_11017874|2022-02-27
hCoV-19/New_Zealand/22MV1134/2022|EPI_ISL_11017938|2022-03-01
hCoV-19/New_Zealand/22MV1142/2022|EPI_ISL_11017789|2022-02-23
hCoV-19/New_Zealand/22MV1161/2022|EPI_ISL_11017569|2022-02-24
hCoV-19/New_Zealand/22MV1204/2022|EPI_ISL_11017833|2022-02-22
hCoV-19/New_Zealand/22MV1205/2022|EPI_ISL_11017885|2022-02-28
hCoV-19/New_Zealand/22MV1221/2022|EPI_ISL_11331656|2022-02-23
hCoV-19/New_Zealand/22MV1252/2022|EPI_ISL_11331425|2022-02-25
hCoV-19/New_Zealand/22MV1266/2022|EPI_ISL_11331838|2022-02-28
hCoV-19/New_Zealand/22MV1269/2022|EPI_ISL_11331540|2022-03-14
hCoV-19/New_Zealand/22MV1273/2022|EPI_ISL_11331675|2022-02-17
hCoV-19/New_Zealand/22MV1287/2022|EPI_ISL_11331533|2022-03-08
hCoV-19/New_Zealand/22MV1291/2022|EPI_ISL_11331450|2022-02-27
hCoV-19/New_Zealand/22MV1308/2022|EPI_ISL_11331440|2022-03-14
hCoV-19/New_Zealand/22MV1324/2022|EPI_ISL_11331582|2022-03-16
hCoV-19/New_Zealand/22MV1334/2022|EPI_ISL_11331539|2022-03-15
hCoV-19/New_Zealand/22MV1347/2022|EPI_ISL_11331836|2022-03-18
hCoV-19/New_Zealand/22MV1350/2022|EPI_ISL_11866025|2022-03-18
hCoV-19/New_Zealand/22MV1402/2022|EPI_ISL_11866049|2022-03-12
hCoV-19/New_Zealand/22MV1404/2022|EPI_ISL_11865958|2022-03-14
hCoV-19/New_Zealand/22MV1413/2022|EPI_ISL_11866006|2022-03-15
hCoV-19/New_Zealand/22MV1457/2022|EPI_ISL_11866273|2022-03-24
hCoV-19/New_Zealand/22MV1471/2022|EPI_ISL_11865678|2022-03-19
hCoV-19/New_Zealand/22MV1513/2022|EPI_ISL_11866287|2022-03-19
hCoV-19/New_Zealand/22MV1518/2022|EPI_ISL_11866034|2022-03-20

hCoV-19/New_Zealand/22MV1630/2022|EPI_ISL_11865853|2022-03-29
hCoV-19/New_Zealand/22MV1639/2022|EPI_ISL_11865694|2022-03-30
hCoV-19/New_Zealand/22MV1646/2022|EPI_ISL_11866268|2022-03-23
hCoV-19/New_Zealand/22MV1654/2022|EPI_ISL_11866231|2022-03-30
hCoV-19/New_Zealand/22MV1669/2022|EPI_ISL_11865747|2022-03-30
hCoV-19/New_Zealand/22MV1699/2022|EPI_ISL_12546227|2022-04-01
hCoV-19/New_Zealand/22MV1703/2022|EPI_ISL_12546197|2022-04-01
hCoV-19/New_Zealand/22MV1711/2022|EPI_ISL_12545185|2022-04-01
hCoV-19/New_Zealand/22MV1713/2022|EPI_ISL_12545936|2022-04-01
hCoV-19/New_Zealand/22MV1723/2022|EPI_ISL_12545013|2022-03-22
hCoV-19/New_Zealand/22MV1750/2022|EPI_ISL_12546403|2022-03-25
hCoV-19/New_Zealand/22MV1797/2022|EPI_ISL_12545432|2022-03-31
hCoV-19/New_Zealand/22MV1808/2022|EPI_ISL_12546173|2022-04-01
hCoV-19/New_Zealand/22MV1855/2022|EPI_ISL_12545325|2022-04-14
hCoV-19/New_Zealand/22MV1875/2022|EPI_ISL_12546522|2022-04-18
hCoV-19/New_Zealand/22MV1911/2022|EPI_ISL_12545236|2022-04-04
hCoV-19/New_Zealand/22MV1954/2022|EPI_ISL_12545165|2022-04-09
hCoV-19/New_Zealand/22MV1960/2022|EPI_ISL_12546075|2022-04-13
hCoV-19/New_Zealand/22MV1972/2022|EPI_ISL_12545525|2022-04-20
hCoV-19/New_Zealand/22MV1975/2022|EPI_ISL_12545112|2022-03-01
hCoV-19/New_Zealand/22MV1985/2022|EPI_ISL_12545429|2022-04-13
hCoV-19/New_Zealand/22MV2017/2022|EPI_ISL_12545600|2022-04-18
hCoV-19/New_Zealand/22MV2019/2022|EPI_ISL_12545610|2022-04-19
hCoV-19/New_Zealand/22MV2024/2022|EPI_ISL_12545239|2022-04-16
hCoV-19/New_Zealand/22MV2040/2022|EPI_ISL_12545177|2022-04-19
hCoV-19/New_Zealand/22MV2072/2022|EPI_ISL_12544973|2022-04-24
hCoV-19/New_Zealand/22MV2089/2022|EPI_ISL_12546125|2022-04-11
hCoV-19/New_Zealand/22MV2094/2022|EPI_ISL_12545019|2022-04-16
hCoV-19/New_Zealand/22MV2097/2022|EPI_ISL_12546245|2022-04-12
hCoV-19/New_Zealand/22MV2107/2022|EPI_ISL_12545390|2022-04-19
hCoV-19/New_Zealand/22MV2117/2022|EPI_ISL_12546124|2022-04-20
hCoV-19/New_Zealand/22MV2123/2022|EPI_ISL_12545853|2022-04-26
hCoV-19/New_Zealand/22MV2132/2022|EPI_ISL_12545890|2022-04-21
hCoV-19/New_Zealand/22MV2137/2022|EPI_ISL_12546121|2022-04-20
hCoV-19/New_Zealand/22MV2164/2022|EPI_ISL_12545585|2022-04-14
hCoV-19/New_Zealand/22MV2179/2022|EPI_ISL_12546164|2022-04-22
hCoV-19/New_Zealand/22MV2191/2022|EPI_ISL_12545230|2022-04-27
hCoV-19/New_Zealand/22MV2192/2022|EPI_ISL_12546212|2022-04-13
hCoV-19/New_Zealand/22MV2205/2022|EPI_ISL_12545965|2022-04-30
hCoV-19/New_Zealand/22MV2213/2022|EPI_ISL_12544944|2022-04-20
hCoV-19/New_Zealand/22MV2231/2022|EPI_ISL_12545620|2022-05-01
hCoV-19/New_Zealand/22MV2250/2022|EPI_ISL_12546283|2022-05-02
hCoV-19/New_Zealand/22MV2254/2022|EPI_ISL_12546090|2022-05-02

hCoV-19/New_Zealand/22MV2257/2022|EPI_ISL_12545939|2022-05-01
hCoV-19/New_Zealand/22MV2262/2022|EPI_ISL_12546270|2022-05-03
hCoV-19/New_Zealand/22MV2273/2022|EPI_ISL_12805626|2022-04-30
hCoV-19/New_Zealand/22MV2285/2022|EPI_ISL_12805528|2022-04-26
hCoV-19/New_Zealand/22MV2286/2022|EPI_ISL_12805622|2022-04-27
hCoV-19/New_Zealand/22MV2297/2022|EPI_ISL_12805745|2022-04-28
hCoV-19/New_Zealand/22MV2318/2022|EPI_ISL_12806162|2022-05-03
hCoV-19/New_Zealand/22MV2329/2022|EPI_ISL_12805727|2022-05-02
hCoV-19/New_Zealand/22MV2332/2022|EPI_ISL_12806215|2022-05-02
hCoV-19/New_Zealand/22MV2356/2022|EPI_ISL_12805553|2022-05-04
hCoV-19/New_Zealand/22MV2363/2022|EPI_ISL_12806086|2022-05-05
hCoV-19/New_Zealand/22MV2370/2022|EPI_ISL_12805644|2022-05-05
hCoV-19/New_Zealand/22MV2379/2022|EPI_ISL_12806191|2022-05-05
hCoV-19/New_Zealand/22MV2393/2022|EPI_ISL_12805581|2022-05-08
hCoV-19/New_Zealand/22MV2399/2022|EPI_ISL_12805466|2022-05-03
hCoV-19/New_Zealand/22MV2406/2022|EPI_ISL_12805412|2022-05-06
hCoV-19/New_Zealand/22MV2414/2022|EPI_ISL_12806231|2022-05-06
hCoV-19/New_Zealand/22MV2430/2022|EPI_ISL_12805777|2022-05-03
hCoV-19/New_Zealand/22MV2434/2022|EPI_ISL_12806056|2022-05-09
hCoV-19/New_Zealand/22MV2443/2022|EPI_ISL_12806253|2022-05-10
hCoV-19/New_Zealand/22MV2448/2022|EPI_ISL_12806097|2022-05-10
hCoV-19/New_Zealand/22MV2453/2022|EPI_ISL_12805933|2022-05-05
hCoV-19/New_Zealand/22MV2460/2022|EPI_ISL_12806317|2022-05-09
hCoV-19/New_Zealand/22MV2470/2022|EPI_ISL_12806172|2022-05-10
hCoV-19/New_Zealand/22MV2482/2022|EPI_ISL_12805969|2022-05-10
hCoV-19/New_Zealand/22MV2490/2022|EPI_ISL_12806052|2022-05-11
hCoV-19/New_Zealand/22MV2503/2022|EPI_ISL_12805941|2022-05-13
hCoV-19/New_Zealand/22MV2523/2022|EPI_ISL_13437866|2022-05-16
hCoV-19/New_Zealand/22MV2526/2022|EPI_ISL_13437650|2022-05-13
hCoV-19/New_Zealand/22MV2527/2022|EPI_ISL_13437996|2022-05-15
hCoV-19/New_Zealand/22MV2529/2022|EPI_ISL_13437176|2022-05-15
hCoV-19/New_Zealand/22MV2539/2022|EPI_ISL_13437641|2022-05-13
hCoV-19/New_Zealand/22MV2547/2022|EPI_ISL_13437368|2022-05-17
hCoV-19/New_Zealand/22MV2561/2022|EPI_ISL_13437094|2022-05-16
hCoV-19/New_Zealand/22MV2565/2022|EPI_ISL_13437498|2022-05-16
hCoV-19/New_Zealand/22MV2567/2022|EPI_ISL_13436982|2022-05-16
hCoV-19/New_Zealand/22MV2581/2022|EPI_ISL_13438113|2022-05-17
hCoV-19/New_Zealand/22MV2582/2022|EPI_ISL_13437857|2022-05-17
hCoV-19/New_Zealand/22MV2590/2022|EPI_ISL_13437169|2022-05-17
hCoV-19/New_Zealand/22MV2592/2022|EPI_ISL_13438375|2022-05-16
hCoV-19/New_Zealand/22MV2603/2022|EPI_ISL_13438821|2022-05-13
hCoV-19/New_Zealand/22MV2607/2022|EPI_ISL_13438624|2022-05-13
hCoV-19/New_Zealand/22MV2615/2022|EPI_ISL_13438561|2022-05-17

hCoV-19/New_Zealand/22MV2629/2022|EPI_ISL_13437369|2022-05-16
hCoV-19/New_Zealand/22MV2633/2022|EPI_ISL_13438706|2022-05-19
hCoV-19/New_Zealand/22MV2636/2022|EPI_ISL_13436919|2022-05-18
hCoV-19/New_Zealand/22MV2639/2022|EPI_ISL_13438058|2022-05-18
hCoV-19/New_Zealand/22MV2645/2022|EPI_ISL_13438547|2022-05-18
hCoV-19/New_Zealand/22MV2652/2022|EPI_ISL_13438348|2022-05-20
hCoV-19/New_Zealand/22MV2654/2022|EPI_ISL_13438808|2022-05-20
hCoV-19/New_Zealand/22MV2661/2022|EPI_ISL_13438494|2022-05-21
hCoV-19/New_Zealand/22MV2664/2022|EPI_ISL_13438594|2022-05-18
hCoV-19/New_Zealand/22MV2677/2022|EPI_ISL_13437928|2022-05-19
hCoV-19/New_Zealand/22MV2682/2022|EPI_ISL_13437521|2022-05-20
hCoV-19/New_Zealand/22MV2686/2022|EPI_ISL_13437538|2022-05-20
hCoV-19/New_Zealand/22MV2693/2022|EPI_ISL_13438152|2022-05-20
hCoV-19/New_Zealand/22MV2699/2022|EPI_ISL_13437817|2022-05-17
hCoV-19/New_Zealand/22MV2701/2022|EPI_ISL_13437317|2022-05-21
hCoV-19/New_Zealand/22MV2703/2022|EPI_ISL_13437329|2022-05-20
hCoV-19/New_Zealand/22MV2706/2022|EPI_ISL_13437508|2022-05-19
hCoV-19/New_Zealand/22MV2712/2022|EPI_ISL_13436736|2022-05-21
hCoV-19/New_Zealand/22MV2713/2022|EPI_ISL_13438096|2022-05-23
hCoV-19/New_Zealand/22MV2732/2022|EPI_ISL_13438030|2022-05-25
hCoV-19/New_Zealand/22MV2740/2022|EPI_ISL_13437402|2022-05-23
hCoV-19/New_Zealand/22MV2743/2022|EPI_ISL_13438799|2022-05-22
hCoV-19/New_Zealand/22MV2775/2022|EPI_ISL_13438774|2022-05-25
hCoV-19/New_Zealand/22MV2782/2022|EPI_ISL_13438988|2022-05-25
hCoV-19/New_Zealand/22MV2784/2022|EPI_ISL_13437701|2022-05-25
hCoV-19/New_Zealand/22MV2787/2022|EPI_ISL_13438651|2022-05-24
hCoV-19/New_Zealand/22MV2791/2022|EPI_ISL_13436832|2022-05-24
hCoV-19/New_Zealand/22MV2800/2022|EPI_ISL_13438824|2022-05-26
hCoV-19/New_Zealand/22MV2802/2022|EPI_ISL_13437266|2022-05-26
hCoV-19/New_Zealand/22MV2816/2022|EPI_ISL_13436758|2022-05-28
hCoV-19/New_Zealand/22MV2822/2022|EPI_ISL_13436923|2022-05-28
hCoV-19/New_Zealand/22MV2828/2022|EPI_ISL_13437500|2022-05-28
hCoV-19/New_Zealand/22MV2832/2022|EPI_ISL_13438075|2022-05-27
hCoV-19/New_Zealand/22MV2855/2022|EPI_ISL_13437135|2022-05-25
hCoV-19/New_Zealand/22MV2957/2022|EPI_ISL_13438393|2022-06-02
hCoV-19/New_Zealand/22MV3002/2022|EPI_ISL_13437831|2022-06-01
hCoV-19/New_Zealand/22MV3006/2022|EPI_ISL_13437999|2022-06-05
hCoV-19/New_Zealand/22MV3011/2022|EPI_ISL_13438704|2022-06-04
hCoV-19/New_Zealand/22MV3028/2022|EPI_ISL_13438661|2022-05-30
hCoV-19/New_Zealand/22MV3040/2022|EPI_ISL_13437623|2022-06-08
hCoV-19/New_Zealand/22MV3087/2022|EPI_ISL_13437765|2022-06-07
hCoV-19/New_Zealand/22MV3109/2022|EPI_ISL_13437596|2022-06-09
hCoV-19/New_Zealand/22MV3112/2022|EPI_ISL_13438421|2022-06-09

hCoV-19/New_Zealand/22MV3138/2022|EPI_ISL_13438403|2022-06-10
hCoV-19/New_Zealand/22MV3154/2022|EPI_ISL_13438091|2022-06-11
hCoV-19/New_Zealand/22MV3162/2022|EPI_ISL_13438771|2022-06-10
hCoV-19/New_Zealand/22MV3183/2022|EPI_ISL_13437949|2022-06-11
hCoV-19/New_Zealand/22MV3200/2022|EPI_ISL_13436753|2022-06-13
hCoV-19/New_Zealand/22MV3203/2022|EPI_ISL_13438659|2022-06-13
hCoV-19/New_Zealand/22MV3218/2022|EPI_ISL_13764794|2022-06-14
hCoV-19/New_Zealand/22MV3235/2022|EPI_ISL_13436789|2022-06-13
hCoV-19/New_Zealand/22MV3251/2022|EPI_ISL_13437472|2022-06-16
hCoV-19/New_Zealand/22MV3267/2022|EPI_ISL_13437020|2022-06-15
hCoV-19/New_Zealand/22MV3272/2022|EPI_ISL_13764199|2022-06-15
hCoV-19/New_Zealand/22MV3330/2022|EPI_ISL_13765081|2022-06-17
hCoV-19/New_Zealand/22MV3348/2022|EPI_ISL_13763901|2022-06-17
hCoV-19/New_Zealand/22MV3370/2022|EPI_ISL_13764420|2022-06-22
hCoV-19/New_Zealand/22MV3410/2022|EPI_ISL_13765381|2022-06-23
hCoV-19/New_Zealand/22MV3470/2022|EPI_ISL_13764567|2022-06-28
hCoV-19/New_Zealand/22MV3518/2022|EPI_ISL_13764410|2022-07-03
hCoV-19/New_Zealand/22MV3519/2022|EPI_ISL_13764913|2022-07-03
hCoV-19/New_Zealand/22MV3526/2022|EPI_ISL_13763943|2022-07-05
hCoV-19/New_Zealand/22XA0008/2022|EPI_ISL_14634604|2022-07-08
hCoV-19/New_Zealand/22XA0040/2022|EPI_ISL_14634616|2022-07-09
hCoV-19/New_Zealand/22XA0083/2022|EPI_ISL_14632755|2022-07-09
hCoV-19/New_Zealand/22XA0087/2022|EPI_ISL_14632757|2022-07-09
hCoV-19/New_Zealand/22XA0091/2022|EPI_ISL_14632752|2022-07-09
hCoV-19/New_Zealand/22XA0182/2022|EPI_ISL_14634697|2022-07-11
hCoV-19/New_Zealand/22XA0208/2022|EPI_ISL_14637163|2022-07-11
hCoV-19/New_Zealand/22XA0225/2022|EPI_ISL_14636742|2022-07-11
hCoV-19/New_Zealand/22XA0230/2022|EPI_ISL_14632760|2022-07-10
hCoV-19/New_Zealand/22XA0243/2022|EPI_ISL_14637166|2022-07-12
hCoV-19/New_Zealand/22XA0248/2022|EPI_ISL_14634800|2022-07-13
hCoV-19/New_Zealand/22XA0268/2022|EPI_ISL_14634790|2022-07-13
hCoV-19/New_Zealand/22XA0272/2022|EPI_ISL_14634793|2022-07-13
hCoV-19/New_Zealand/22XA0274/2022|EPI_ISL_14634798|2022-07-13
hCoV-19/New_Zealand/22XA0279/2022|EPI_ISL_14634805|2022-07-13
hCoV-19/New_Zealand/22XA0280/2022|EPI_ISL_14634772|2022-07-13
hCoV-19/New_Zealand/22XA0290/2022|EPI_ISL_14634811|2022-07-14
hCoV-19/New_Zealand/22XA0325/2022|EPI_ISL_14637175|2022-07-15
hCoV-19/New_Zealand/22XA0414/2022|EPI_ISL_14632774|2022-07-15
hCoV-19/New_Zealand/22XA0434/2022|EPI_ISL_14632799|2022-07-16
hCoV-19/New_Zealand/22XA0468/2022|EPI_ISL_14632823|2022-07-17
hCoV-19/New_Zealand/22XA0509/2022|EPI_ISL_14637040|2022-07-18
hCoV-19/New_Zealand/22XA0545/2022|EPI_ISL_14637392|2022-07-19
hCoV-19/New_Zealand/22XA0595/2022|EPI_ISL_14634952|2022-07-20

hCoV-19/New_Zealand/22XA0606/2022|EPI_ISL_14634973|2022-07-23
hCoV-19/New_Zealand/22XA0613/2022|EPI_ISL_14634965|2022-07-23
hCoV-19/New_Zealand/22XA0630/2022|EPI_ISL_14636846|2022-07-23
hCoV-19/New_Zealand/22XA0669/2022|EPI_ISL_14632837|2022-07-18
hCoV-19/New_Zealand/22XA0678/2022|EPI_ISL_14634977|2022-07-24
hCoV-19/New_Zealand/22XA0809/2022|EPI_ISL_14637240|2022-07-25
hCoV-19/New_Zealand/22XA0827/2022|EPI_ISL_14632896|2022-07-25
hCoV-19/New_Zealand/22XA0864/2022|EPI_ISL_14632919|2022-07-26
hCoV-19/New_Zealand/22XA0870/2022|EPI_ISL_14636872|2022-07-27
hCoV-19/New_Zealand/22XA0884/2022|EPI_ISL_14636873|2022-07-27
hCoV-19/New_Zealand/22XA0901/2022|EPI_ISL_14633285|2022-07-26
hCoV-19/New_Zealand/22XA0906/2022|EPI_ISL_14633291|2022-07-27
hCoV-19/New_Zealand/22XA0998/2022|EPI_ISL_14637364|2022-07-30
hCoV-19/New_Zealand/22XA1022/2022|EPI_ISL_14632955|2022-07-30
hCoV-19/New_Zealand/22XA1050/2022|EPI_ISL_14632976|2022-07-31
hCoV-19/New_Zealand/22XA1070/2022|EPI_ISL_14637103|2022-07-31
hCoV-19/New_Zealand/22XA1074/2022|EPI_ISL_14637258|2022-08-01
hCoV-19/New_Zealand/22XA1082/2022|EPI_ISL_14635153|2022-07-31
hCoV-19/New_Zealand/22XA1084/2022|EPI_ISL_14635155|2022-07-31
hCoV-19/New_Zealand/22XA1111/2022|EPI_ISL_14635134|2022-07-31
hCoV-19/New_Zealand/22XA1142/2022|EPI_ISL_14632989|2022-08-01
hCoV-19/New_Zealand/22XA1143/2022|EPI_ISL_14632991|2022-08-01
hCoV-19/New_Zealand/22XA1153/2022|EPI_ISL_14633004|2022-08-01
hCoV-19/New_Zealand/22XA1169/2022|EPI_ISL_14633342|2022-08-01
hCoV-19/New_Zealand/22XA1178/2022|EPI_ISL_14633341|2022-08-01
hCoV-19/New_Zealand/22XA1186/2022|EPI_ISL_14633350|2022-08-02
hCoV-19/New_Zealand/22XA1190/2022|EPI_ISL_14633353|2022-08-02
hCoV-19/New_Zealand/22XA1212/2022|EPI_ISL_14637387|2022-08-02
hCoV-19/New_Zealand/22XA1284/2022|EPI_ISL_14637265|2022-08-04
hCoV-19/New_Zealand/22XA1330/2022|EPI_ISL_14637276|2022-08-06
hCoV-19/New_Zealand/22XA1344/2022|EPI_ISL_14637115|2022-08-05
hCoV-19/New_Zealand/22XA1351/2022|EPI_ISL_14633381|2022-08-07
hCoV-19/New_Zealand/22XA1353/2022|EPI_ISL_14633383|2022-08-07
hCoV-19/New_Zealand/22XA1373/2022|EPI_ISL_14635251|2022-08-07
hCoV-19/New_Zealand/22XA1404/2022|EPI_ISL_14633042|2022-08-07
hCoV-19/New_Zealand/22XA1434/2022|EPI_ISL_14635259|2022-08-08
hCoV-19/New_Zealand/22XA1442/2022|EPI_ISL_14635274|2022-08-08
hCoV-19/New_Zealand/22XA1464/2022|EPI_ISL_14633405|2022-08-08
hCoV-19/New_Zealand/22XA1471/2022|EPI_ISL_14633420|2022-08-08
hCoV-19/New_Zealand/22XA1473/2022|EPI_ISL_14633406|2022-08-08
hCoV-19/New_Zealand/22XA1493/2022|EPI_ISL_14633390|2022-08-08
hCoV-19/New_Zealand/22XA1510/2022|EPI_ISL_14636957|2022-08-08
hCoV-19/New_Zealand/22XA1527/2022|EPI_ISL_14633060|2022-08-08

hCoV-19/New_Zealand/22XA1533/2022|EPI_ISL_14633055|2022-08-08
hCoV-19/New_Zealand/22XA1536/2022|EPI_ISL_14633047|2022-08-08
hCoV-19/New_Zealand/22XA1553/2022|EPI_ISL_14637126|2022-08-08
hCoV-19/New_Zealand/22XA1581/2022|EPI_ISL_14636968|2022-08-09
hCoV-19/New_Zealand/22XA1606/2022|EPI_ISL_14633063|2022-08-09
hCoV-19/New_Zealand/22XA1652/2022|EPI_ISL_14633466|2022-08-10
hCoV-19/New_Zealand/22XA1668/2022|EPI_ISL_14633072|2022-08-10
hCoV-19/New_Zealand/22XA1708/2022|EPI_ISL_14635339|2022-08-14
hCoV-19/New_Zealand/22XA1714/2022|EPI_ISL_14633473|2022-08-13
hCoV-19/New_Zealand/22XA1717/2022|EPI_ISL_14633475|2022-08-13
hCoV-19/New_Zealand/22XA1729/2022|EPI_ISL_14637018|2022-08-14
hCoV-19/New_Zealand/22XA1740/2022|EPI_ISL_14637023|2022-08-14
hCoV-19/New_Zealand/22XA1763/2022|EPI_ISL_14633492|2022-08-14
hCoV-19/New_Zealand/22XA1782/2022|EPI_ISL_14633090|2022-08-14
hCoV-19/New_Zealand/22XA1824/2022|EPI_ISL_14635360|2022-08-15
hCoV-19/New_Zealand/22XA1827/2022|EPI_ISL_14635361|2022-08-15
hCoV-19/New_Zealand/22XA1834/2022|EPI_ISL_14637366|2022-08-15
hCoV-19/New_Zealand/22XA1835/2022|EPI_ISL_14637381|2022-08-15
hCoV-19/New_Zealand/22XA1865/2022|EPI_ISL_15278016|2022-08-16
hCoV-19/New_Zealand/22XA1885/2022|EPI_ISL_15277994|2022-08-15
hCoV-19/New_Zealand/22XA1886/2022|EPI_ISL_15277995|2022-08-15
hCoV-19/New_Zealand/22XA1895/2022|EPI_ISL_15275591|2022-08-16
hCoV-19/New_Zealand/22XA1915/2022|EPI_ISL_15275907|2022-08-16
hCoV-19/New_Zealand/22XA1922/2022|EPI_ISL_15278317|2022-08-16
hCoV-19/New_Zealand/22XA1924/2022|EPI_ISL_15277272|2022-08-17
hCoV-19/New_Zealand/22XA1927/2022|EPI_ISL_15278037|2022-08-17
hCoV-19/New_Zealand/22XA1938/2022|EPI_ISL_15278029|2022-08-17
hCoV-19/New_Zealand/22XA1965/2022|EPI_ISL_15275599|2022-08-17
hCoV-19/New_Zealand/22XA1983/2022|EPI_ISL_15278388|2022-08-17
hCoV-19/New_Zealand/22XA1987/2022|EPI_ISL_15278399|2022-08-18
hCoV-19/New_Zealand/22XA2007/2022|EPI_ISL_15278052|2022-08-20
hCoV-19/New_Zealand/22XA2065/2022|EPI_ISL_15278408|2022-08-20
hCoV-19/New_Zealand/22XA2073/2022|EPI_ISL_15276867|2022-08-21
hCoV-19/New_Zealand/22XA2086/2022|EPI_ISL_15276877|2022-08-22
hCoV-19/New_Zealand/22XA2097/2022|EPI_ISL_15278415|2022-08-22
hCoV-19/New_Zealand/22XA2181/2022|EPI_ISL_15275963|2022-08-22
hCoV-19/New_Zealand/22XA2187/2022|EPI_ISL_15275970|2022-08-22
hCoV-19/New_Zealand/22XA2208/2022|EPI_ISL_15275982|2022-08-23
hCoV-19/New_Zealand/22XA2216/2022|EPI_ISL_15275987|2022-08-22
hCoV-19/New_Zealand/22XA2250/2022|EPI_ISL_15278427|2022-08-23
hCoV-19/New_Zealand/22XA2253/2022|EPI_ISL_15278424|2022-08-22
hCoV-19/New_Zealand/22XA2272/2022|EPI_ISL_15275649|2022-08-23
hCoV-19/New_Zealand/22XA2276/2022|EPI_ISL_15275661|2022-08-23

hCoV-19/New_Zealand/22XA2312/2022|EPI_ISL_15278109|2022-08-25
hCoV-19/New_Zealand/22XA2338/2022|EPI_ISL_15276019|2022-08-27
hCoV-19/New_Zealand/22XA2346/2022|EPI_ISL_15277332|2022-08-27
hCoV-19/New_Zealand/22XA2370/2022|EPI_ISL_15278445|2022-08-27
hCoV-19/New_Zealand/22XA2383/2022|EPI_ISL_15278587|2022-08-27
hCoV-19/New_Zealand/22XA2386/2022|EPI_ISL_15278122|2022-08-28
hCoV-19/New_Zealand/22XA2390/2022|EPI_ISL_15278116|2022-08-28
hCoV-19/New_Zealand/22XA2402/2022|EPI_ISL_15275679|2022-08-28
hCoV-19/New_Zealand/22XA2403/2022|EPI_ISL_15275684|2022-08-28
hCoV-19/New_Zealand/22XA2453/2022|EPI_ISL_15276043|2022-08-29
hCoV-19/New_Zealand/22XA2462/2022|EPI_ISL_15278461|2022-08-29
hCoV-19/New_Zealand/22XA2469/2022|EPI_ISL_15278463|2022-08-29
hCoV-19/New_Zealand/22XA2471/2022|EPI_ISL_15278467|2022-08-29
hCoV-19/New_Zealand/22XA2474/2022|EPI_ISL_15275696|2022-08-29
hCoV-19/New_Zealand/22XA2488/2022|EPI_ISL_15278330|2022-08-29
hCoV-19/New_Zealand/22XA2512/2022|EPI_ISL_15276078|2022-08-30
hCoV-19/New_Zealand/22XA2517/2022|EPI_ISL_15276083|2022-08-30
hCoV-19/New_Zealand/22XA2520/2022|EPI_ISL_15276082|2022-08-30
hCoV-19/New_Zealand/22XA2527/2022|EPI_ISL_15278468|2022-08-30
hCoV-19/New_Zealand/22XA2560/2022|EPI_ISL_15276087|2022-08-31
hCoV-19/New_Zealand/22XA2561/2022|EPI_ISL_15276088|2022-08-31
hCoV-19/New_Zealand/22XA2601/2022|EPI_ISL_15276093|2022-09-03
hCoV-19/New_Zealand/22XA2604/2022|EPI_ISL_15276100|2022-09-03
hCoV-19/New_Zealand/22XA2610/2022|EPI_ISL_15278478|2022-09-04
hCoV-19/New_Zealand/22XA2616/2022|EPI_ISL_15277287|2022-09-03
hCoV-19/New_Zealand/22XA2620/2022|EPI_ISL_15278206|2022-09-03
hCoV-19/New_Zealand/22XA2632/2022|EPI_ISL_15275702|2022-08-30
hCoV-19/New_Zealand/22XA2634/2022|EPI_ISL_15275700|2022-08-30
hCoV-19/New_Zealand/22XA2670/2022|EPI_ISL_15278215|2022-09-04
hCoV-19/New_Zealand/22XA2708/2022|EPI_ISL_15278224|2022-09-05
hCoV-19/New_Zealand/22XA2794/2022|EPI_ISL_15278339|2022-09-05
hCoV-19/New_Zealand/22XA2868/2022|EPI_ISL_15278494|2022-09-07
hCoV-19/New_Zealand/22XA2870/2022|EPI_ISL_15278496|2022-09-07
hCoV-19/New_Zealand/22XA2875/2022|EPI_ISL_15275739|2022-09-07
hCoV-19/New_Zealand/22XA2950/2022|EPI_ISL_15278268|2022-09-12
hCoV-19/New_Zealand/22XA2960/2022|EPI_ISL_15276183|2022-09-12
hCoV-19/New_Zealand/22XA2967/2022|EPI_ISL_15276169|2022-09-12
hCoV-19/New_Zealand/22XA2969/2022|EPI_ISL_15276176|2022-09-12
hCoV-19/New_Zealand/22XA3001/2022|EPI_ISL_15278503|2022-09-12
hCoV-19/New_Zealand/22XA3009/2022|EPI_ISL_15276193|2022-09-13
hCoV-19/New_Zealand/22XA3043/2022|EPI_ISL_15278516|2022-09-13
hCoV-19/New_Zealand/22XA3047/2022|EPI_ISL_15276199|2022-09-14
hCoV-19/New_Zealand/22XA3134/2022|EPI_ISL_15278533|2022-09-18

hCoV-19/New_Zealand/22XA3135/2022|EPI_ISL_15278602|2022-09-17
hCoV-19/New_Zealand/22XA3137/2022|EPI_ISL_15278535|2022-09-18
hCoV-19/New_Zealand/22XA3153/2022|EPI_ISL_15275792|2022-09-17
hCoV-19/New_Zealand/22XA3170/2022|EPI_ISL_15276217|2022-09-19
hCoV-19/New_Zealand/22XA3229/2022|EPI_ISL_15275810|2022-09-20
hCoV-19/New_Zealand/22XA3230/2022|EPI_ISL_15275811|2022-09-20
hCoV-19/New_Zealand/22XA3245/2022|EPI_ISL_15276228|2022-09-20
hCoV-19/New_Zealand/22XA3278/2022|EPI_ISL_15276234|2022-09-21
hCoV-19/New_Zealand/22XA3299/2022|EPI_ISL_15277309|2022-09-27
hCoV-19/New_Zealand/22XA3332/2022|EPI_ISL_15323382|2022-09-27
hCoV-19/New_Zealand/22XA3339/2022|EPI_ISL_15323644|2022-09-28
hCoV-19/New_Zealand/22XA3348/2022|EPI_ISL_15322601|2022-09-27
hCoV-19/New_Zealand/22XA3350/2022|EPI_ISL_15322592|2022-09-27
hCoV-19/New_Zealand/22XA3366/2022|EPI_ISL_15323801|2022-09-27
hCoV-19/New_Zealand/22XA3380/2022|EPI_ISL_15323656|2022-09-28
hCoV-19/New_Zealand/22XA3430/2022|EPI_ISL_15322693|2022-10-03
hCoV-19/New_Zealand/22XA3434/2022|EPI_ISL_15323659|2022-10-01
hCoV-19/New_Zealand/22XA3442/2022|EPI_ISL_15323370|2022-10-01
hCoV-19/New_Zealand/22XA3475/2022|EPI_ISL_15323792|2022-10-03
hCoV-19/New_Zealand/22XA3501/2022|EPI_ISL_15322515|2022-10-03
hCoV-19/New_Zealand/22XA3505/2022|EPI_ISL_15322714|2022-10-04
hCoV-19/New_Zealand/22XA3512/2022|EPI_ISL_15323695|2022-10-04
hCoV-19/New_Zealand/22XA3518/2022|EPI_ISL_15323701|2022-10-04
hCoV-19/New_Zealand/22XA3559/2022|EPI_ISL_15955914|2022-10-07
hCoV-19/New_Zealand/22XA3580/2022|EPI_ISL_15957622|2022-10-08
hCoV-19/New_Zealand/22XA3606/2022|EPI_ISL_15955504|2022-10-08
hCoV-19/New_Zealand/22XA3611/2022|EPI_ISL_15955502|2022-10-08
hCoV-19/New_Zealand/22XA3648/2022|EPI_ISL_15955517|2022-10-10
hCoV-19/New_Zealand/22XA3652/2022|EPI_ISL_15955516|2022-10-10
hCoV-19/New_Zealand/22XA3655/2022|EPI_ISL_15959005|2022-10-10
hCoV-19/New_Zealand/22XA3676/2022|EPI_ISL_15959012|2022-10-12
hCoV-19/New_Zealand/22XA3719/2022|EPI_ISL_15958594|2022-10-12
hCoV-19/New_Zealand/22XA3730/2022|EPI_ISL_15955945|2022-10-12
hCoV-19/New_Zealand/22XA3789/2022|EPI_ISL_15958603|2022-10-13
hCoV-19/New_Zealand/22XA3819/2022|EPI_ISL_15957821|2022-10-15
hCoV-19/New_Zealand/22XA3833/2022|EPI_ISL_15955544|2022-10-14
hCoV-19/New_Zealand/22XA3863/2022|EPI_ISL_15958616|2022-10-16
hCoV-19/New_Zealand/22XA3915/2022|EPI_ISL_15955964|2022-10-17
hCoV-19/New_Zealand/22XA3936/2022|EPI_ISL_15955981|2022-10-18
hCoV-19/New_Zealand/22XA3950/2022|EPI_ISL_15957080|2022-10-18
hCoV-19/New_Zealand/22XA3951/2022|EPI_ISL_15957078|2022-10-18
hCoV-19/New_Zealand/22XA3964/2022|EPI_ISL_15959031|2022-10-17
hCoV-19/New_Zealand/22XA3966/2022|EPI_ISL_15959026|2022-10-17

hCoV-19/New_Zealand/22XA4007/2022|EPI_ISL_15958054|2022-10-18
hCoV-19/New_Zealand/22XA4011/2022|EPI_ISL_15958033|2022-10-18
hCoV-19/New_Zealand/22XA4045/2022|EPI_ISL_15958633|2022-10-19
hCoV-19/New_Zealand/22XA4056/2022|EPI_ISL_15955573|2022-10-18
hCoV-19/New_Zealand/22XA4067/2022|EPI_ISL_15958914|2022-10-19
hCoV-19/New_Zealand/22XA4087/2022|EPI_ISL_15957834|2022-10-22
hCoV-19/New_Zealand/22XA4093/2022|EPI_ISL_15958658|2022-10-21
hCoV-19/New_Zealand/22XA4104/2022|EPI_ISL_15958664|2022-10-22
hCoV-19/New_Zealand/22XA4141/2022|EPI_ISL_15955595|2022-10-21
hCoV-19/New_Zealand/22XA4142/2022|EPI_ISL_15955600|2022-10-21
hCoV-19/New_Zealand/22XA4147/2022|EPI_ISL_15955608|2022-10-22
hCoV-19/New_Zealand/22XA4149/2022|EPI_ISL_15955605|2022-10-22
hCoV-19/New_Zealand/22XA4182/2022|EPI_ISL_15956023|2022-10-25
hCoV-19/New_Zealand/22XA4206/2022|EPI_ISL_15957846|2022-10-25
hCoV-19/New_Zealand/22XA4209/2022|EPI_ISL_15958920|2022-10-21
hCoV-19/New_Zealand/22XA4217/2022|EPI_ISL_15958924|2022-10-24
hCoV-19/New_Zealand/22XA4233/2022|EPI_ISL_15955618|2022-10-24
hCoV-19/New_Zealand/22XA4234/2022|EPI_ISL_15955617|2022-10-24
hCoV-19/New_Zealand/22XA4263/2022|EPI_ISL_15959050|2022-10-25
hCoV-19/New_Zealand/22XA4280/2022|EPI_ISL_15955625|2022-10-25
hCoV-19/New_Zealand/22XA4302/2022|EPI_ISL_15959053|2022-10-27
hCoV-19/New_Zealand/22XA4310/2022|EPI_ISL_15959056|2022-10-26
hCoV-19/New_Zealand/22XA4313/2022|EPI_ISL_15957851|2022-10-26
hCoV-19/New_Zealand/22XA4335/2022|EPI_ISL_15958690|2022-10-27
hCoV-19/New_Zealand/22XA4344/2022|EPI_ISL_15957862|2022-10-28
hCoV-19/New_Zealand/22XA4363/2022|EPI_ISL_15957867|2022-10-29
hCoV-19/New_Zealand/22XA4390/2022|EPI_ISL_15955648|2022-10-28
hCoV-19/New_Zealand/22XA4397/2022|EPI_ISL_15955641|2022-10-28
hCoV-19/New_Zealand/22XA4409/2022|EPI_ISL_15955660|2022-10-29
hCoV-19/New_Zealand/22XA4449/2022|EPI_ISL_15959066|2022-10-27
hCoV-19/New_Zealand/22XA4455/2022|EPI_ISL_15959074|2022-10-30
hCoV-19/New_Zealand/22XA4466/2022|EPI_ISL_15958942|2022-10-28
hCoV-19/New_Zealand/22XA4545/2022|EPI_ISL_15957879|2022-11-01
hCoV-19/New_Zealand/22XA4546/2022|EPI_ISL_15957877|2022-11-01
hCoV-19/New_Zealand/22XA4554/2022|EPI_ISL_15955673|2022-10-31
hCoV-19/New_Zealand/22XA4563/2022|EPI_ISL_15958947|2022-11-01
hCoV-19/New_Zealand/22XA4566/2022|EPI_ISL_15955677|2022-11-01
hCoV-19/New_Zealand/22XA4595/2022|EPI_ISL_15959093|2022-11-03
hCoV-19/New_Zealand/22XA4610/2022|EPI_ISL_15956079|2022-11-02
hCoV-19/New_Zealand/22XA4632/2022|EPI_ISL_15955688|2022-11-02
hCoV-19/New_Zealand/22XA4633/2022|EPI_ISL_15955689|2022-11-02
hCoV-19/New_Zealand/22XA4639/2022|EPI_ISL_15955683|2022-11-02
hCoV-19/New_Zealand/22XA4705/2022|EPI_ISL_15958958|2022-11-04

hCoV-19/New_Zealand/22XA4729/2022|EPI_ISL_15956094|2022-11-04
hCoV-19/New_Zealand/22XA4744/2022|EPI_ISL_15959116|2022-11-06
hCoV-19/New_Zealand/22XA4771/2022|EPI_ISL_15958747|2022-11-06
hCoV-19/New_Zealand/22XA4777/2022|EPI_ISL_15958009|2022-11-07
hCoV-19/New_Zealand/22XA4810/2022|EPI_ISL_15958012|2022-11-07
hCoV-19/New_Zealand/22XA4811/2022|EPI_ISL_15957706|2022-11-08
hCoV-19/New_Zealand/22XA4839/2022|EPI_ISL_15958022|2022-11-09
hCoV-19/New_Zealand/22XA4843/2022|EPI_ISL_15957711|2022-11-09
hCoV-19/New_Zealand/22XA4854/2022|EPI_ISL_15958025|2022-11-09
hCoV-19/New_Zealand/22XA4857/2022|EPI_ISL_15958967|2022-11-08
hCoV-19/New_Zealand/22XA4861/2022|EPI_ISL_15958878|2022-11-06
hCoV-19/New_Zealand/22XA4871/2022|EPI_ISL_15956127|2022-11-07
hCoV-19/New_Zealand/22XA4876/2022|EPI_ISL_15956118|2022-11-07
hCoV-19/New_Zealand/22XA4877/2022|EPI_ISL_15956121|2022-11-07
hCoV-19/New_Zealand/22XA4938/2022|EPI_ISL_15959141|2022-11-09
hCoV-19/New_Zealand/22XA4952/2022|EPI_ISL_15955730|2022-11-09
hCoV-19/New_Zealand/22XA5063/2022|EPI_ISL_15957917|2022-11-11
hCoV-19/New_Zealand/22XA5074/2022|EPI_ISL_15957724|2022-11-12
hCoV-19/New_Zealand/22XA5148/2022|EPI_ISL_15957937|2022-11-14
hCoV-19/New_Zealand/22XA5159/2022|EPI_ISL_15956178|2022-11-13
hCoV-19/New_Zealand/22XA5177/2022|EPI_ISL_15956183|2022-11-14
hCoV-19/New_Zealand/22XA5207/2022|EPI_ISL_15957943|2022-11-15
hCoV-19/New_Zealand/22XA5220/2022|EPI_ISL_15957732|2022-11-14
hCoV-19/New_Zealand/22XA5241/2022|EPI_ISL_15958814|2022-11-14
hCoV-19/New_Zealand/22XA5249/2022|EPI_ISL_15957751|2022-11-16
hCoV-19/New_Zealand/22XA5281/2022|EPI_ISL_15956208|2022-11-16
hCoV-19/New_Zealand/22XA5308/2022|EPI_ISL_15956210|2022-11-16
hCoV-19/New_Zealand/22XA5330/2022|EPI_ISL_15956237|2022-11-18
hCoV-19/New_Zealand/22XA5335/2022|EPI_ISL_15956241|2022-11-19
hCoV-19/New_Zealand/22XA5343/2022|EPI_ISL_15958837|2022-11-17
hCoV-19/New_Zealand/22XA5346/2022|EPI_ISL_15958836|2022-11-17
hCoV-19/New_Zealand/22XA5359/2022|EPI_ISL_15957974|2022-11-18
hCoV-19/New_Zealand/22XA5367/2022|EPI_ISL_15958849|2022-11-18
hCoV-19/New_Zealand/22XA5395/2022|EPI_ISL_15959183|2022-11-18
hCoV-19/New_Zealand/22XA5418/2022|EPI_ISL_15957772|2022-11-18
hCoV-19/New_Zealand/22XA5468/2022|EPI_ISL_15957789|2022-11-21
hCoV-19/New_Zealand/22XA5479/2022|EPI_ISL_15957795|2022-11-22
hCoV-19/New_Zealand/22XA5521/2022|EPI_ISL_15955813|2022-11-20
hCoV-19/New_Zealand/22XA5537/2022|EPI_ISL_15955826|2022-11-22
hCoV-19/New_Zealand/22XA5553/2022|EPI_ISL_15956266|2022-11-22
hCoV-19/New_Zealand/22XA5612/2022|EPI_ISL_16050561|2022-11-23
hCoV-19/New_Zealand/22XA5632/2022|EPI_ISL_16050564|2022-11-24
hCoV-19/New_Zealand/22XA5669/2022|EPI_ISL_16050283|2022-11-24

hCoV-19/New_Zealand/22XA5674/2022|EPI_ISL_16050292|2022-11-24
hCoV-19/New_Zealand/22XA5688/2022|EPI_ISL_16050284|2022-11-25
hCoV-19/New_Zealand/22XA5700/2022|EPI_ISL_16050721|2022-11-24
hCoV-19/New_Zealand/22XA5714/2022|EPI_ISL_16050533|2022-11-25
hCoV-19/New_Zealand/22XA5752/2022|EPI_ISL_16050724|2022-11-25
hCoV-19/New_Zealand/22XA5756/2022|EPI_ISL_16050726|2022-11-25
hCoV-19/New_Zealand/22XA5777/2022|EPI_ISL_16050546|2022-11-28
hCoV-19/New_Zealand/22XA5779/2022|EPI_ISL_16050538|2022-11-27
hCoV-19/New_Zealand/22XA5845/2022|EPI_ISL_16050316|2022-11-28
hCoV-19/New_Zealand/22XA5846/2022|EPI_ISL_16050221|2022-11-25
hCoV-19/New_Zealand/22XA5853/2022|EPI_ISL_16050232|2022-11-28
hCoV-19/New_Zealand/22XA5859/2022|EPI_ISL_16050239|2022-11-28
hCoV-19/New_Zealand/22XA5884/2022|EPI_ISL_16050711|2022-11-28
hCoV-19/New_Zealand/22XA5933/2022|EPI_ISL_16435450|2022-11-30
hCoV-19/New_Zealand/22XA5994/2022|EPI_ISL_16436022|2022-11-30
hCoV-19/New_Zealand/22XA6001/2022|EPI_ISL_16436024|2022-11-30
hCoV-19/New_Zealand/22XA6035/2022|EPI_ISL_16434053|2022-11-30
hCoV-19/New_Zealand/22XA6054/2022|EPI_ISL_16436241|2022-11-30
hCoV-19/New_Zealand/22XA6082/2022|EPI_ISL_16434076|2022-12-02
hCoV-19/New_Zealand/22XA6087/2022|EPI_ISL_16434079|2022-12-02
hCoV-19/New_Zealand/22XA6116/2022|EPI_ISL_16435467|2022-12-02
hCoV-19/New_Zealand/22XA6163/2022|EPI_ISL_16435480|2022-12-04
hCoV-19/New_Zealand/22XA6168/2022|EPI_ISL_16436052|2022-12-04
hCoV-19/New_Zealand/22XA6195/2022|EPI_ISL_16433771|2022-12-03
hCoV-19/New_Zealand/22XA6210/2022|EPI_ISL_16433783|2022-12-04
hCoV-19/New_Zealand/22XA6257/2022|EPI_ISL_16434112|2022-12-05
hCoV-19/New_Zealand/22XA6315/2022|EPI_ISL_16433792|2022-12-05
hCoV-19/New_Zealand/22XA6329/2022|EPI_ISL_16436377|2022-12-06
hCoV-19/New_Zealand/22XA6369/2022|EPI_ISL_16436383|2022-12-07
hCoV-19/New_Zealand/22XA6379/2022|EPI_ISL_16436255|2022-12-06
hCoV-19/New_Zealand/22XA6399/2022|EPI_ISL_16435502|2022-12-06
hCoV-19/New_Zealand/22XA6401/2022|EPI_ISL_16435366|2022-12-06
hCoV-19/New_Zealand/22XA6403/2022|EPI_ISL_16435367|2022-12-07
hCoV-19/New_Zealand/22XA6422/2022|EPI_ISL_16435370|2022-12-07
hCoV-19/New_Zealand/22XA6478/2022|EPI_ISL_16435512|2022-12-08
hCoV-19/New_Zealand/22XA6491/2022|EPI_ISL_16436390|2022-12-08
hCoV-19/New_Zealand/22XA6502/2022|EPI_ISL_16436394|2022-12-09
hCoV-19/New_Zealand/22XA6515/2022|EPI_ISL_16436397|2022-12-10
hCoV-19/New_Zealand/22XA6517/2022|EPI_ISL_16436399|2022-12-10
hCoV-19/New_Zealand/22XA6518/2022|EPI_ISL_16436396|2022-12-10
hCoV-19/New_Zealand/22XA6529/2022|EPI_ISL_16436106|2022-12-08
hCoV-19/New_Zealand/22XA6559/2022|EPI_ISL_16436113|2022-12-09
hCoV-19/New_Zealand/22XA6573/2022|EPI_ISL_16435533|2022-12-10

hCoV-19/New_Zealand/22XA6582/2022|EPI_ISL_16436123|2022-12-10
hCoV-19/New_Zealand/22XA6591/2022|EPI_ISL_16436286|2022-12-09
hCoV-19/New_Zealand/22XA6613/2022|EPI_ISL_16434197|2022-12-12
hCoV-19/New_Zealand/22XA6620/2022|EPI_ISL_16434201|2022-12-12
hCoV-19/New_Zealand/22XA6625/2022|EPI_ISL_16434205|2022-12-12
hCoV-19/New_Zealand/22XA6635/2022|EPI_ISL_16436411|2022-12-12
hCoV-19/New_Zealand/22XA6775/2022|EPI_ISL_16435406|2022-12-14
hCoV-19/New_Zealand/22XA6835/2022|EPI_ISL_16434215|2022-12-14
hCoV-19/New_Zealand/22XA6839/2022|EPI_ISL_16434219|2022-12-14
hCoV-19/New_Zealand/22XA6888/2022|EPI_ISL_16436155|2022-12-14
hCoV-19/New_Zealand/22XA6900/2022|EPI_ISL_16435416|2022-12-15
hCoV-19/New_Zealand/22XA6962/2022|EPI_ISL_16435575|2022-12-16
hCoV-19/New_Zealand/22XA6978/2022|EPI_ISL_16436182|2022-12-16
hCoV-19/New_Zealand/22XA6993/2022|EPI_ISL_16436189|2022-12-17
hCoV-19/New_Zealand/22XA7014/2022|EPI_ISL_16436167|2022-12-15
hCoV-19/New_Zealand/22XA7062/2022|EPI_ISL_16433907|2022-12-17
hCoV-19/New_Zealand/22XA7077/2022|EPI_ISL_16433917|2022-12-18
hCoV-19/New_Zealand/22XA7120/2022|EPI_ISL_16436226|2022-12-19
hCoV-19/New_Zealand/22XA7137/2022|EPI_ISL_16434257|2022-12-18
hCoV-19/New_Zealand/22XA7167/2022|EPI_ISL_16436203|2022-12-19
hCoV-19/New_Zealand/22YA0011/2022|EPI_ISL_14633507|2022-07-08
hCoV-19/New_Zealand/22YA0016/2022|EPI_ISL_14633514|2022-07-09
hCoV-19/New_Zealand/22YA0043/2022|EPI_ISL_14635655|2022-07-10
hCoV-19/New_Zealand/22YA0050/2022|EPI_ISL_14636729|2022-07-10
hCoV-19/New_Zealand/22YA0206/2022|EPI_ISL_14634729|2022-07-12
hCoV-19/New_Zealand/22YA0225/2022|EPI_ISL_14634758|2022-07-12
hCoV-19/New_Zealand/22YA0253/2022|EPI_ISL_14635445|2022-07-13
hCoV-19/New_Zealand/22YA0255/2022|EPI_ISL_14636707|2022-07-13
hCoV-19/New_Zealand/22YA0262/2022|EPI_ISL_14633573|2022-07-11
hCoV-19/New_Zealand/22YA0311/2022|EPI_ISL_14636749|2022-07-12
hCoV-19/New_Zealand/22YA0314/2022|EPI_ISL_14636755|2022-07-12
hCoV-19/New_Zealand/22YA0346/2022|EPI_ISL_14634564|2022-07-12
hCoV-19/New_Zealand/22YA0348/2022|EPI_ISL_14634563|2022-07-12
hCoV-19/New_Zealand/22YA0382/2022|EPI_ISL_14633538|2022-07-10
hCoV-19/New_Zealand/22YA0385/2022|EPI_ISL_14634849|2022-07-16
hCoV-19/New_Zealand/22YA0388/2022|EPI_ISL_14634838|2022-07-16
hCoV-19/New_Zealand/22YA0409/2022|EPI_ISL_14634815|2022-07-15
hCoV-19/New_Zealand/22YA0450/2022|EPI_ISL_14636581|2022-07-13
hCoV-19/New_Zealand/22YA0464/2022|EPI_ISL_14636506|2022-07-10
hCoV-19/New_Zealand/22YA0470/2022|EPI_ISL_14635556|2022-07-12
hCoV-19/New_Zealand/22YA0472/2022|EPI_ISL_14635555|2022-07-12
hCoV-19/New_Zealand/22YA0505/2022|EPI_ISL_14633729|2022-07-16
hCoV-19/New_Zealand/22YA0513/2022|EPI_ISL_14634870|2022-07-17

hCoV-19/New_Zealand/22YA0551/2022|EPI_ISL_14635401|2022-07-16
hCoV-19/New_Zealand/22YA0620/2022|EPI_ISL_14633740|2022-07-18
hCoV-19/New_Zealand/22YA0641/2022|EPI_ISL_14636585|2022-07-13
hCoV-19/New_Zealand/22YA0659/2022|EPI_ISL_14636472|2022-07-15
hCoV-19/New_Zealand/22YA0691/2022|EPI_ISL_14636693|2022-07-19
hCoV-19/New_Zealand/22YA0704/2022|EPI_ISL_14636467|2022-07-18
hCoV-19/New_Zealand/22YA0755/2022|EPI_ISL_14633830|2022-07-19
hCoV-19/New_Zealand/22YA0760/2022|EPI_ISL_14633844|2022-07-19
hCoV-19/New_Zealand/22YA0772/2022|EPI_ISL_14633810|2022-07-19
hCoV-19/New_Zealand/22YA0774/2022|EPI_ISL_14636829|2022-07-20
hCoV-19/New_Zealand/22YA0792/2022|EPI_ISL_14635586|2022-07-19
hCoV-19/New_Zealand/22YA0823/2022|EPI_ISL_14633200|2022-07-19
hCoV-19/New_Zealand/22YA0836/2022|EPI_ISL_14633188|2022-07-19
hCoV-19/New_Zealand/22YA0859/2022|EPI_ISL_14632862|2022-07-23
hCoV-19/New_Zealand/22YA0864/2022|EPI_ISL_14632868|2022-07-23
hCoV-19/New_Zealand/22YA0875/2022|EPI_ISL_14633913|2022-07-23
hCoV-19/New_Zealand/22YA0890/2022|EPI_ISL_14633899|2022-07-23
hCoV-19/New_Zealand/22YA0895/2022|EPI_ISL_14633903|2022-07-23
hCoV-19/New_Zealand/22YA1004/2022|EPI_ISL_14632885|2022-07-24
hCoV-19/New_Zealand/22YA1013/2022|EPI_ISL_14636674|2022-07-25
hCoV-19/New_Zealand/22YA1016/2022|EPI_ISL_14636550|2022-07-26
hCoV-19/New_Zealand/22YA1029/2022|EPI_ISL_14634016|2022-07-25
hCoV-19/New_Zealand/22YA1036/2022|EPI_ISL_14634005|2022-07-25
hCoV-19/New_Zealand/22YA1066/2022|EPI_ISL_14636857|2022-07-25
hCoV-19/New_Zealand/22YA1242/2022|EPI_ISL_14634060|2022-07-26
hCoV-19/New_Zealand/22YA1255/2022|EPI_ISL_14633983|2022-07-25
hCoV-19/New_Zealand/22YA1280/2022|EPI_ISL_14635426|2022-07-31
hCoV-19/New_Zealand/22YA1291/2022|EPI_ISL_14633300|2022-07-30
hCoV-19/New_Zealand/22YA1302/2022|EPI_ISL_14633309|2022-07-30
hCoV-19/New_Zealand/22YA1336/2022|EPI_ISL_14635507|2022-08-01
hCoV-19/New_Zealand/22YA1367/2022|EPI_ISL_14634152|2022-07-31
hCoV-19/New_Zealand/22YA1370/2022|EPI_ISL_14635156|2022-08-01
hCoV-19/New_Zealand/22YA1392/2022|EPI_ISL_14635178|2022-08-01
hCoV-19/New_Zealand/22YA1420/2022|EPI_ISL_14636678|2022-08-02
hCoV-19/New_Zealand/22YA1434/2022|EPI_ISL_14634207|2022-08-01
hCoV-19/New_Zealand/22YA1453/2022|EPI_ISL_14634244|2022-08-02
hCoV-19/New_Zealand/22YA1463/2022|EPI_ISL_14634202|2022-08-01
hCoV-19/New_Zealand/22YA1473/2022|EPI_ISL_14636932|2022-08-02
hCoV-19/New_Zealand/22YA1525/2022|EPI_ISL_14635503|2022-08-03
hCoV-19/New_Zealand/22YA1553/2022|EPI_ISL_14636624|2022-08-01
hCoV-19/New_Zealand/22YA1556/2022|EPI_ISL_14636563|2022-08-01
hCoV-19/New_Zealand/22YA1557/2022|EPI_ISL_14636565|2022-08-01
hCoV-19/New_Zealand/22YA1564/2022|EPI_ISL_14634310|2022-08-03

hCoV-19/New_Zealand/22YA1676/2022|EPI_ISL_14635494|2022-08-08
hCoV-19/New_Zealand/22YA1738/2022|EPI_ISL_14636681|2022-08-08
hCoV-19/New_Zealand/22YA1749/2022|EPI_ISL_14634348|2022-08-08
hCoV-19/New_Zealand/22YA1812/2022|EPI_ISL_14637299|2022-08-10
hCoV-19/New_Zealand/22YA1837/2022|EPI_ISL_14636526|2022-08-09
hCoV-19/New_Zealand/22YA1844/2022|EPI_ISL_14636649|2022-08-09
hCoV-19/New_Zealand/22YA1860/2022|EPI_ISL_14634472|2022-08-10
hCoV-19/New_Zealand/22YA1899/2022|EPI_ISL_14635308|2022-08-10
hCoV-19/New_Zealand/22YA1968/2022|EPI_ISL_14637015|2022-08-13
hCoV-19/New_Zealand/22YA1970/2022|EPI_ISL_14637010|2022-08-13
hCoV-19/New_Zealand/22YA1980/2022|EPI_ISL_14635609|2022-08-10
hCoV-19/New_Zealand/22YA1998/2022|EPI_ISL_14634361|2022-08-08
hCoV-19/New_Zealand/22YA2015/2022|EPI_ISL_14634521|2022-08-14
hCoV-19/New_Zealand/22YA2092/2022|EPI_ISL_15275882|2022-08-15
hCoV-19/New_Zealand/22YA2142/2022|EPI_ISL_15276261|2022-08-15
hCoV-19/New_Zealand/22YA2168/2022|EPI_ISL_15276796|2022-08-16
hCoV-19/New_Zealand/22YA2178/2022|EPI_ISL_15276805|2022-08-16
hCoV-19/New_Zealand/22YA2185/2022|EPI_ISL_15276812|2022-08-16
hCoV-19/New_Zealand/22YA2192/2022|EPI_ISL_15276820|2022-08-17
hCoV-19/New_Zealand/22YA2220/2022|EPI_ISL_15277891|2022-08-17
hCoV-19/New_Zealand/22YA2223/2022|EPI_ISL_15276269|2022-08-16
hCoV-19/New_Zealand/22YA2224/2022|EPI_ISL_15276275|2022-08-16
hCoV-19/New_Zealand/22YA2225/2022|EPI_ISL_15276274|2022-08-16
hCoV-19/New_Zealand/22YA2236/2022|EPI_ISL_15276327|2022-08-17
hCoV-19/New_Zealand/22YA2237/2022|EPI_ISL_15276342|2022-08-17
hCoV-19/New_Zealand/22YA2337/2022|EPI_ISL_15278061|2022-08-21
hCoV-19/New_Zealand/22YA2371/2022|EPI_ISL_15276412|2022-08-22
hCoV-19/New_Zealand/22YA2421/2022|EPI_ISL_15276425|2022-08-23
hCoV-19/New_Zealand/22YA2430/2022|EPI_ISL_15276442|2022-08-23
hCoV-19/New_Zealand/22YA2473/2022|EPI_ISL_15276922|2022-08-24
hCoV-19/New_Zealand/22YA2481/2022|EPI_ISL_15276929|2022-08-24
hCoV-19/New_Zealand/22YA2496/2022|EPI_ISL_15277911|2022-08-23
hCoV-19/New_Zealand/22YA2534/2022|EPI_ISL_15276945|2022-08-27
hCoV-19/New_Zealand/22YA2575/2022|EPI_ISL_15276530|2022-08-29
hCoV-19/New_Zealand/22YA2597/2022|EPI_ISL_15956990|2022-08-29
hCoV-19/New_Zealand/22YA2606/2022|EPI_ISL_15276962|2022-08-29
hCoV-19/New_Zealand/22YA2647/2022|EPI_ISL_15277200|2022-08-31
hCoV-19/New_Zealand/22YA2651/2022|EPI_ISL_15276555|2022-08-30
hCoV-19/New_Zealand/22YA2653/2022|EPI_ISL_15276553|2022-08-30
hCoV-19/New_Zealand/22YA2755/2022|EPI_ISL_15276991|2022-08-31
hCoV-19/New_Zealand/22YA2769/2022|EPI_ISL_15277154|2022-09-03
hCoV-19/New_Zealand/22YA2787/2022|EPI_ISL_15277003|2022-09-03
hCoV-19/New_Zealand/22YA2789/2022|EPI_ISL_15277005|2022-09-03

hCoV-19/New_Zealand/22YA2792/2022|EPI_ISL_15277008|2022-09-03
hCoV-19/New_Zealand/22YA2824/2022|EPI_ISL_15277897|2022-09-05
hCoV-19/New_Zealand/22YA2827/2022|EPI_ISL_15276614|2022-09-05
hCoV-19/New_Zealand/22YA2916/2022|EPI_ISL_15277848|2022-09-05
hCoV-19/New_Zealand/22YA2925/2022|EPI_ISL_15277852|2022-09-06
hCoV-19/New_Zealand/22YA2935/2022|EPI_ISL_15277236|2022-09-08
hCoV-19/New_Zealand/22YA2959/2022|EPI_ISL_15277056|2022-09-07
hCoV-19/New_Zealand/22YA2960/2022|EPI_ISL_15277057|2022-09-08
hCoV-19/New_Zealand/22YA2999/2022|EPI_ISL_15277071|2022-09-12
hCoV-19/New_Zealand/22YA3012/2022|EPI_ISL_15276687|2022-09-11
hCoV-19/New_Zealand/22YA3015/2022|EPI_ISL_15276683|2022-09-10
hCoV-19/New_Zealand/22YA3016/2022|EPI_ISL_15276680|2022-09-10
hCoV-19/New_Zealand/22YA3054/2022|EPI_ISL_15277072|2022-09-12
hCoV-19/New_Zealand/22YA3060/2022|EPI_ISL_15276723|2022-09-12
hCoV-19/New_Zealand/22YA3113/2022|EPI_ISL_15277249|2022-09-18
hCoV-19/New_Zealand/22YA3124/2022|EPI_ISL_15277090|2022-09-17
hCoV-19/New_Zealand/22YA3125/2022|EPI_ISL_15277091|2022-09-17
hCoV-19/New_Zealand/22YA3144/2022|EPI_ISL_15277096|2022-09-18
hCoV-19/New_Zealand/22YA3187/2022|EPI_ISL_15277104|2022-09-20
hCoV-19/New_Zealand/22YA3194/2022|EPI_ISL_15276779|2022-09-21
hCoV-19/New_Zealand/22YA3200/2022|EPI_ISL_15276778|2022-09-21
hCoV-19/New_Zealand/22YA3223/2022|EPI_ISL_15277352|2022-09-22
hCoV-19/New_Zealand/22YA3265/2022|EPI_ISL_15277908|2022-09-27
hCoV-19/New_Zealand/22YA3266/2022|EPI_ISL_15277251|2022-09-27
hCoV-19/New_Zealand/22YA3267/2022|EPI_ISL_15277171|2022-09-27
hCoV-19/New_Zealand/22YA3278/2022|EPI_ISL_15322963|2022-09-27
hCoV-19/New_Zealand/22YA3288/2022|EPI_ISL_15322992|2022-09-28
hCoV-19/New_Zealand/22YA3313/2022|EPI_ISL_15323353|2022-09-30
hCoV-19/New_Zealand/22YA3334/2022|EPI_ISL_15323023|2022-10-02
hCoV-19/New_Zealand/22YA3338/2022|EPI_ISL_15323036|2022-10-03
hCoV-19/New_Zealand/22YA3374/2022|EPI_ISL_15958535|2022-10-05
hCoV-19/New_Zealand/22YA3388/2022|EPI_ISL_15956997|2022-10-04
hCoV-19/New_Zealand/22YA3390/2022|EPI_ISL_15956999|2022-10-05
hCoV-19/New_Zealand/22YA3412/2022|EPI_ISL_15956315|2022-10-09
hCoV-19/New_Zealand/22YA3476/2022|EPI_ISL_15956346|2022-10-11
hCoV-19/New_Zealand/22YA3502/2022|EPI_ISL_15957041|2022-10-12
hCoV-19/New_Zealand/22YA3510/2022|EPI_ISL_15956368|2022-10-13
hCoV-19/New_Zealand/22YA3517/2022|EPI_ISL_15957039|2022-10-12
hCoV-19/New_Zealand/22YA3524/2022|EPI_ISL_15957490|2022-10-13
hCoV-19/New_Zealand/22YA3529/2022|EPI_ISL_15956385|2022-10-13
hCoV-19/New_Zealand/22YA3568/2022|EPI_ISL_15957046|2022-10-13
hCoV-19/New_Zealand/22YA3585/2022|EPI_ISL_15957061|2022-10-16
hCoV-19/New_Zealand/22YA3593/2022|EPI_ISL_15956401|2022-10-15

hCoV-19/New_Zealand/22YA3621/2022|EPI_ISL_15956453|2022-10-19
hCoV-19/New_Zealand/22YA3651/2022|EPI_ISL_15956486|2022-10-21
hCoV-19/New_Zealand/22YA3709/2022|EPI_ISL_15957124|2022-10-22
hCoV-19/New_Zealand/22YA3715/2022|EPI_ISL_15957130|2022-10-23
hCoV-19/New_Zealand/22YA3721/2022|EPI_ISL_15957091|2022-10-19
hCoV-19/New_Zealand/22YA3727/2022|EPI_ISL_15957104|2022-10-20
hCoV-19/New_Zealand/22YA3732/2022|EPI_ISL_15957103|2022-10-19
hCoV-19/New_Zealand/22YA3742/2022|EPI_ISL_15956525|2022-10-25
hCoV-19/New_Zealand/22YA3743/2022|EPI_ISL_15956526|2022-10-25
hCoV-19/New_Zealand/22YA3748/2022|EPI_ISL_15957454|2022-10-25
hCoV-19/New_Zealand/22YA3845/2022|EPI_ISL_15957152|2022-10-26
hCoV-19/New_Zealand/22YA3846/2022|EPI_ISL_15957153|2022-10-26
hCoV-19/New_Zealand/22YA3857/2022|EPI_ISL_15957580|2022-10-27
hCoV-19/New_Zealand/22YA3867/2022|EPI_ISL_15957549|2022-10-29
hCoV-19/New_Zealand/22YA3873/2022|EPI_ISL_15956614|2022-10-30
hCoV-19/New_Zealand/22YA3919/2022|EPI_ISL_15957169|2022-10-28
hCoV-19/New_Zealand/22YA3952/2022|EPI_ISL_15957188|2022-10-30
hCoV-19/New_Zealand/22YA3966/2022|EPI_ISL_15956574|2022-10-27
hCoV-19/New_Zealand/22YA4011/2022|EPI_ISL_15956663|2022-11-01
hCoV-19/New_Zealand/22YA4014/2022|EPI_ISL_15956658|2022-11-01
hCoV-19/New_Zealand/22YA4099/2022|EPI_ISL_15958480|2022-11-04
hCoV-19/New_Zealand/22YA4114/2022|EPI_ISL_15956747|2022-11-06
hCoV-19/New_Zealand/22YA4117/2022|EPI_ISL_15956757|2022-11-07
hCoV-19/New_Zealand/22YA4142/2022|EPI_ISL_15957241|2022-11-05
hCoV-19/New_Zealand/22YA4148/2022|EPI_ISL_15957520|2022-11-08
hCoV-19/New_Zealand/22YA4151/2022|EPI_ISL_15957600|2022-11-08
hCoV-19/New_Zealand/22YA4159/2022|EPI_ISL_15956774|2022-11-07
hCoV-19/New_Zealand/22YA4161/2022|EPI_ISL_15956773|2022-11-07
hCoV-19/New_Zealand/22YA4174/2022|EPI_ISL_15957263|2022-11-07
hCoV-19/New_Zealand/22YA4185/2022|EPI_ISL_15957279|2022-11-08
hCoV-19/New_Zealand/22YA4217/2022|EPI_ISL_15957293|2022-11-09
hCoV-19/New_Zealand/22YA4237/2022|EPI_ISL_15956817|2022-11-10
hCoV-19/New_Zealand/22YA4253/2022|EPI_ISL_15957295|2022-11-09
hCoV-19/New_Zealand/22YA4258/2022|EPI_ISL_15957299|2022-11-09
hCoV-19/New_Zealand/22YA4273/2022|EPI_ISL_15957468|2022-11-10
hCoV-19/New_Zealand/22YA4291/2022|EPI_ISL_15957302|2022-11-10
hCoV-19/New_Zealand/22YA4332/2022|EPI_ISL_15958484|2022-11-10
hCoV-19/New_Zealand/22YA4333/2022|EPI_ISL_15958483|2022-11-10
hCoV-19/New_Zealand/22YA4379/2022|EPI_ISL_15956893|2022-11-15
hCoV-19/New_Zealand/22YA4390/2022|EPI_ISL_15957349|2022-11-14
hCoV-19/New_Zealand/22YA4420/2022|EPI_ISL_15958541|2022-11-15
hCoV-19/New_Zealand/22YA4428/2022|EPI_ISL_15957501|2022-11-16
hCoV-19/New_Zealand/22YA4435/2022|EPI_ISL_15957372|2022-11-16

hCoV-19/New_Zealand/22YA4437/2022|EPI_ISL_15957374|2022-11-16
hCoV-19/New_Zealand/22YA4438/2022|EPI_ISL_15957375|2022-11-16
hCoV-19/New_Zealand/22YA4457/2022|EPI_ISL_15957541|2022-11-18
hCoV-19/New_Zealand/22YA4572/2022|EPI_ISL_15957397|2022-11-19
hCoV-19/New_Zealand/22YA4578/2022|EPI_ISL_15957402|2022-11-19
hCoV-19/New_Zealand/22YA4620/2022|EPI_ISL_15958533|2022-11-21
hCoV-19/New_Zealand/22YA4632/2022|EPI_ISL_15957475|2022-11-23
hCoV-19/New_Zealand/22YA4639/2022|EPI_ISL_15957419|2022-11-21
hCoV-19/New_Zealand/22YA4643/2022|EPI_ISL_15957423|2022-11-21
hCoV-19/New_Zealand/22YA4662/2022|EPI_ISL_15957442|2022-11-22
hCoV-19/New_Zealand/22YA4663/2022|EPI_ISL_15957443|2022-11-22
hCoV-19/New_Zealand/22YA4667/2022|EPI_ISL_15957447|2022-11-22
hCoV-19/New_Zealand/22YA4675/2022|EPI_ISL_16050342|2022-11-22
hCoV-19/New_Zealand/22YA4677/2022|EPI_ISL_16050352|2022-11-23
hCoV-19/New_Zealand/22YA4690/2022|EPI_ISL_16050393|2022-11-22
hCoV-19/New_Zealand/22YA4696/2022|EPI_ISL_16050392|2022-11-22
hCoV-19/New_Zealand/22YA4709/2022|EPI_ISL_16050365|2022-11-23
hCoV-19/New_Zealand/22YA4714/2022|EPI_ISL_16050346|2022-11-23
hCoV-19/New_Zealand/22YA4718/2022|EPI_ISL_16050591|2022-11-22
hCoV-19/New_Zealand/22YA4772/2022|EPI_ISL_16050430|2022-11-24
hCoV-19/New_Zealand/22YA4793/2022|EPI_ISL_16050450|2022-11-25
hCoV-19/New_Zealand/22YA4799/2022|EPI_ISL_16050456|2022-11-26
hCoV-19/New_Zealand/22YA4820/2022|EPI_ISL_16050419|2022-11-23
hCoV-19/New_Zealand/22YA4903/2022|EPI_ISL_16434791|2022-11-28
hCoV-19/New_Zealand/22YA4925/2022|EPI_ISL_16434279|2022-11-29
hCoV-19/New_Zealand/22YA4941/2022|EPI_ISL_16434801|2022-11-29
hCoV-19/New_Zealand/22YA4959/2022|EPI_ISL_16435307|2022-12-01
hCoV-19/New_Zealand/22YA4970/2022|EPI_ISL_16435624|2022-11-30
hCoV-19/New_Zealand/22YA5085/2022|EPI_ISL_16436349|2022-12-01
hCoV-19/New_Zealand/22YA5092/2022|EPI_ISL_16435252|2022-12-05
hCoV-19/New_Zealand/22YA5097/2022|EPI_ISL_16435288|2022-12-05
hCoV-19/New_Zealand/22YA5121/2022|EPI_ISL_16434852|2022-12-01
hCoV-19/New_Zealand/22YA5146/2022|EPI_ISL_16434880|2022-12-04
hCoV-19/New_Zealand/22YA5167/2022|EPI_ISL_16434893|2022-12-05
hCoV-19/New_Zealand/22YA5181/2022|EPI_ISL_16434909|2022-12-05
hCoV-19/New_Zealand/22YA5193/2022|EPI_ISL_16434841|2022-12-01
hCoV-19/New_Zealand/22YA5210/2022|EPI_ISL_16434903|2022-12-05
hCoV-19/New_Zealand/22YA5234/2022|EPI_ISL_16434365|2022-12-02
hCoV-19/New_Zealand/22YA5314/2022|EPI_ISL_16434942|2022-12-07
hCoV-19/New_Zealand/22YA5330/2022|EPI_ISL_16434938|2022-12-07
hCoV-19/New_Zealand/22YA5332/2022|EPI_ISL_16434940|2022-12-07
hCoV-19/New_Zealand/22YA5335/2022|EPI_ISL_16434945|2022-12-07
hCoV-19/New_Zealand/22YA5358/2022|EPI_ISL_16435265|2022-12-10

hCoV-19/New_Zealand/22YA5364/2022|EPI_ISL_16435294|2022-12-09
hCoV-19/New_Zealand/22YA5370/2022|EPI_ISL_16435267|2022-12-10
hCoV-19/New_Zealand/22YA5405/2022|EPI_ISL_16436005|2022-12-07
hCoV-19/New_Zealand/22YA5414/2022|EPI_ISL_16434534|2022-12-08
hCoV-19/New_Zealand/22YA5427/2022|EPI_ISL_16434503|2022-12-07
hCoV-19/New_Zealand/22YA5550/2022|EPI_ISL_16434981|2022-12-09
hCoV-19/New_Zealand/22YA5557/2022|EPI_ISL_16434989|2022-12-09
hCoV-19/New_Zealand/22YA5574/2022|EPI_ISL_16435006|2022-12-11
hCoV-19/New_Zealand/22YA5610/2022|EPI_ISL_16435260|2022-12-13
hCoV-19/New_Zealand/22YA5705/2022|EPI_ISL_16435057|2022-12-13
hCoV-19/New_Zealand/22YA5724/2022|EPI_ISL_16435093|2022-12-14
hCoV-19/New_Zealand/22YA5725/2022|EPI_ISL_16435094|2022-12-14
hCoV-19/New_Zealand/22YA5737/2022|EPI_ISL_16435028|2022-12-12
hCoV-19/New_Zealand/22YA5811/2022|EPI_ISL_16435090|2022-12-14
hCoV-19/New_Zealand/22YA5824/2022|EPI_ISL_16435110|2022-12-15
hCoV-19/New_Zealand/22YA5835/2022|EPI_ISL_16434717|2022-12-15
hCoV-19/New_Zealand/22YA5839/2022|EPI_ISL_16434747|2022-12-16
hCoV-19/New_Zealand/22YA5848/2022|EPI_ISL_16434729|2022-12-16
hCoV-19/New_Zealand/22YA5928/2022|EPI_ISL_16435168|2022-12-17
hCoV-19/New_Zealand/22YA5961/2022|EPI_ISL_16434767|2022-12-19
hCoV-19/New_Zealand/22YA5985/2022|EPI_ISL_16435104|2022-12-15
hCoV-19/New_Zealand/22YA6003/2022|EPI_ISL_16435149|2022-12-16
hCoV-19/New_Zealand/22YA6011/2022|EPI_ISL_16435160|2022-12-16
hCoV-19/New_Zealand/22YA6029/2022|EPI_ISL_16435181|2022-12-17
hCoV-19/New_Zealand/22ZA0003/2022|EPI_ISL_14635799|2022-07-08
hCoV-19/New_Zealand/22ZA0011/2022|EPI_ISL_14635800|2022-07-09
hCoV-19/New_Zealand/22ZA0019/2022|EPI_ISL_14635809|2022-07-08
hCoV-19/New_Zealand/22ZA0036/2022|EPI_ISL_14635726|2022-07-09
hCoV-19/New_Zealand/22ZA0040/2022|EPI_ISL_14635729|2022-07-09
hCoV-19/New_Zealand/22ZA0067/2022|EPI_ISL_14633519|2022-07-09
hCoV-19/New_Zealand/22ZA0088/2022|EPI_ISL_14634645|2022-07-10
hCoV-19/New_Zealand/22ZA0103/2022|EPI_ISL_14634641|2022-07-10
hCoV-19/New_Zealand/22ZA0119/2022|EPI_ISL_14634659|2022-07-10
hCoV-19/New_Zealand/22ZA0134/2022|EPI_ISL_14633566|2022-07-11
hCoV-19/New_Zealand/22ZA0137/2022|EPI_ISL_14633583|2022-07-11
hCoV-19/New_Zealand/22ZA0153/2022|EPI_ISL_14633535|2022-07-10
hCoV-19/New_Zealand/22ZA0166/2022|EPI_ISL_14633590|2022-07-11
hCoV-19/New_Zealand/22ZA0174/2022|EPI_ISL_14633556|2022-07-11
hCoV-19/New_Zealand/22ZA0186/2022|EPI_ISL_14635835|2022-07-11
hCoV-19/New_Zealand/22ZA0199/2022|EPI_ISL_14635838|2022-07-12
hCoV-19/New_Zealand/22ZA0286/2022|EPI_ISL_14635760|2022-07-13
hCoV-19/New_Zealand/22ZA0313/2022|EPI_ISL_14633608|2022-07-12
hCoV-19/New_Zealand/22ZA0347/2022|EPI_ISL_14633649|2022-07-13

hCoV-19/New_Zealand/22ZA0386/2022|EPI_ISL_14633708|2022-07-15
hCoV-19/New_Zealand/22ZA0388/2022|EPI_ISL_14633713|2022-07-15
hCoV-19/New_Zealand/22ZA0425/2022|EPI_ISL_14635899|2022-07-16
hCoV-19/New_Zealand/22ZA0457/2022|EPI_ISL_14635923|2022-07-17
hCoV-19/New_Zealand/22ZA0467/2022|EPI_ISL_14635761|2022-07-17
hCoV-19/New_Zealand/22ZA0507/2022|EPI_ISL_14635946|2022-07-18
hCoV-19/New_Zealand/22ZA0512/2022|EPI_ISL_14635952|2022-07-19
hCoV-19/New_Zealand/22ZA0520/2022|EPI_ISL_14635956|2022-07-19
hCoV-19/New_Zealand/22ZA0534/2022|EPI_ISL_14633759|2022-07-18
hCoV-19/New_Zealand/22ZA0594/2022|EPI_ISL_14634902|2022-07-18
hCoV-19/New_Zealand/22ZA0601/2022|EPI_ISL_14634898|2022-07-18
hCoV-19/New_Zealand/22ZA0603/2022|EPI_ISL_14634892|2022-07-18
hCoV-19/New_Zealand/22ZA0612/2022|EPI_ISL_14635768|2022-07-18
hCoV-19/New_Zealand/22ZA0621/2022|EPI_ISL_14635960|2022-07-19
hCoV-19/New_Zealand/22ZA0624/2022|EPI_ISL_14635965|2022-07-19
hCoV-19/New_Zealand/22ZA0628/2022|EPI_ISL_14635962|2022-07-19
hCoV-19/New_Zealand/22ZA0643/2022|EPI_ISL_14635748|2022-07-19
hCoV-19/New_Zealand/22ZA0649/2022|EPI_ISL_14633806|2022-07-18
hCoV-19/New_Zealand/22ZA0657/2022|EPI_ISL_14633111|2022-07-19
hCoV-19/New_Zealand/22ZA0658/2022|EPI_ISL_14633112|2022-07-20
hCoV-19/New_Zealand/22ZA0663/2022|EPI_ISL_14633815|2022-07-19
hCoV-19/New_Zealand/22ZA0671/2022|EPI_ISL_14633782|2022-07-18
hCoV-19/New_Zealand/22ZA0677/2022|EPI_ISL_14633818|2022-07-19
hCoV-19/New_Zealand/22ZA0681/2022|EPI_ISL_14633850|2022-07-20
hCoV-19/New_Zealand/22ZA0701/2022|EPI_ISL_14634938|2022-07-19
hCoV-19/New_Zealand/22ZA0705/2022|EPI_ISL_14634944|2022-07-19
hCoV-19/New_Zealand/22ZA0765/2022|EPI_ISL_14635781|2022-07-20
hCoV-19/New_Zealand/22ZA0769/2022|EPI_ISL_14637204|2022-07-19
hCoV-19/New_Zealand/22ZA0770/2022|EPI_ISL_14637210|2022-07-20
hCoV-19/New_Zealand/22ZA0807/2022|EPI_ISL_14633854|2022-07-20
hCoV-19/New_Zealand/22ZA0814/2022|EPI_ISL_14635994|2022-07-22
hCoV-19/New_Zealand/22ZA0818/2022|EPI_ISL_14635985|2022-07-23
hCoV-19/New_Zealand/22ZA0881/2022|EPI_ISL_14636149|2022-07-25
hCoV-19/New_Zealand/22ZA0894/2022|EPI_ISL_14633915|2022-07-23
hCoV-19/New_Zealand/22ZA0940/2022|EPI_ISL_14633230|2022-07-20
hCoV-19/New_Zealand/22ZA0949/2022|EPI_ISL_14633218|2022-07-20
hCoV-19/New_Zealand/22ZA0970/2022|EPI_ISL_14633942|2022-07-24
hCoV-19/New_Zealand/22ZA0986/2022|EPI_ISL_14633122|2022-07-25
hCoV-19/New_Zealand/22ZA0992/2022|EPI_ISL_14633895|2022-07-23
hCoV-19/New_Zealand/22ZA0996/2022|EPI_ISL_14633944|2022-07-25
hCoV-19/New_Zealand/22ZA1050/2022|EPI_ISL_14634013|2022-07-25
hCoV-19/New_Zealand/22ZA1091/2022|EPI_ISL_14636015|2022-07-26
hCoV-19/New_Zealand/22ZA1096/2022|EPI_ISL_14636185|2022-07-26

hCoV-19/New_Zealand/22ZA1099/2022|EPI_ISL_14636179|2022-07-26
hCoV-19/New_Zealand/22ZA1102/2022|EPI_ISL_14636198|2022-07-27
hCoV-19/New_Zealand/22ZA1112/2022|EPI_ISL_14636195|2022-07-27
hCoV-19/New_Zealand/22ZA1152/2022|EPI_ISL_14633997|2022-07-25
hCoV-19/New_Zealand/22ZA1156/2022|EPI_ISL_14634026|2022-07-25
hCoV-19/New_Zealand/22ZA1182/2022|EPI_ISL_14634044|2022-07-26
hCoV-19/New_Zealand/22ZA1193/2022|EPI_ISL_14635035|2022-07-26
hCoV-19/New_Zealand/22ZA1299/2022|EPI_ISL_14636238|2022-07-31
hCoV-19/New_Zealand/22ZA1327/2022|EPI_ISL_14634132|2022-07-30
hCoV-19/New_Zealand/22ZA1341/2022|EPI_ISL_14636892|2022-07-30
hCoV-19/New_Zealand/22ZA1379/2022|EPI_ISL_14636243|2022-07-31
hCoV-19/New_Zealand/22ZA1381/2022|EPI_ISL_14636260|2022-07-31
hCoV-19/New_Zealand/22ZA1390/2022|EPI_ISL_14636264|2022-07-31
hCoV-19/New_Zealand/22ZA1397/2022|EPI_ISL_14636272|2022-08-01
hCoV-19/New_Zealand/22ZA1406/2022|EPI_ISL_14633315|2022-07-31
hCoV-19/New_Zealand/22ZA1429/2022|EPI_ISL_14634160|2022-08-01
hCoV-19/New_Zealand/22ZA1456/2022|EPI_ISL_14636285|2022-08-02
hCoV-19/New_Zealand/22ZA1470/2022|EPI_ISL_14637385|2022-07-31
hCoV-19/New_Zealand/22ZA1485/2022|EPI_ISL_14634189|2022-08-01
hCoV-19/New_Zealand/22ZA1530/2022|EPI_ISL_14636060|2022-08-02
hCoV-19/New_Zealand/22ZA1553/2022|EPI_ISL_14636309|2022-08-04
hCoV-19/New_Zealand/22ZA1564/2022|EPI_ISL_14636045|2022-08-03
hCoV-19/New_Zealand/22ZA1579/2022|EPI_ISL_14635195|2022-08-02
hCoV-19/New_Zealand/22ZA1590/2022|EPI_ISL_14635212|2022-08-02
hCoV-19/New_Zealand/22ZA1603/2022|EPI_ISL_14634227|2022-08-02
hCoV-19/New_Zealand/22ZA1630/2022|EPI_ISL_14637112|2022-08-04
hCoV-19/New_Zealand/22ZA1641/2022|EPI_ISL_14634262|2022-08-02
hCoV-19/New_Zealand/22ZA1649/2022|EPI_ISL_14634276|2022-08-03
hCoV-19/New_Zealand/22ZA1660/2022|EPI_ISL_14636320|2022-08-06
hCoV-19/New_Zealand/22ZA1665/2022|EPI_ISL_14636325|2022-08-06
hCoV-19/New_Zealand/22ZA1684/2022|EPI_ISL_14636338|2022-08-07
hCoV-19/New_Zealand/22ZA1705/2022|EPI_ISL_14634279|2022-08-03
hCoV-19/New_Zealand/22ZA1719/2022|EPI_ISL_14634170|2022-08-01
hCoV-19/New_Zealand/22ZA1745/2022|EPI_ISL_14636349|2022-08-07
hCoV-19/New_Zealand/22ZA1762/2022|EPI_ISL_14634346|2022-08-07
hCoV-19/New_Zealand/22ZA1764/2022|EPI_ISL_14634343|2022-08-07
hCoV-19/New_Zealand/22ZA1785/2022|EPI_ISL_14636372|2022-08-08
hCoV-19/New_Zealand/22ZA1798/2022|EPI_ISL_14636088|2022-08-08
hCoV-19/New_Zealand/22ZA1800/2022|EPI_ISL_14634386|2022-08-08
hCoV-19/New_Zealand/22ZA1860/2022|EPI_ISL_14636096|2022-08-09
hCoV-19/New_Zealand/22ZA1911/2022|EPI_ISL_14635284|2022-08-09
hCoV-19/New_Zealand/22ZA1936/2022|EPI_ISL_14636391|2022-08-10
hCoV-19/New_Zealand/22ZA1947/2022|EPI_ISL_14636394|2022-08-11

hCoV-19/New_Zealand/22ZA1992/2022|EPI_ISL_14634450|2022-08-10
hCoV-19/New_Zealand/22ZA1998/2022|EPI_ISL_14634430|2022-08-09
hCoV-19/New_Zealand/22ZA2006/2022|EPI_ISL_14636401|2022-08-12
hCoV-19/New_Zealand/22ZA2031/2022|EPI_ISL_14634487|2022-08-12
hCoV-19/New_Zealand/22ZA2049/2022|EPI_ISL_14637323|2022-08-13
hCoV-19/New_Zealand/22ZA2076/2022|EPI_ISL_14636419|2022-08-14
hCoV-19/New_Zealand/22ZA2101/2022|EPI_ISL_14634446|2022-08-09
hCoV-19/New_Zealand/22ZA2104/2022|EPI_ISL_14634488|2022-08-12
hCoV-19/New_Zealand/22ZA2130/2022|EPI_ISL_14636443|2022-08-15
hCoV-19/New_Zealand/22ZA2142/2022|EPI_ISL_14636121|2022-08-15
hCoV-19/New_Zealand/22ZA2145/2022|EPI_ISL_14636115|2022-08-15
hCoV-19/New_Zealand/22ZA2157/2022|EPI_ISL_14634555|2022-08-15
hCoV-19/New_Zealand/22ZA2173/2022|EPI_ISL_14636457|2022-08-17
hCoV-19/New_Zealand/22ZA2204/2022|EPI_ISL_14634546|2022-08-15
hCoV-19/New_Zealand/22ZA2224/2022|EPI_ISL_15276294|2022-08-16
hCoV-19/New_Zealand/22ZA2241/2022|EPI_ISL_15277447|2022-08-16
hCoV-19/New_Zealand/22ZA2288/2022|EPI_ISL_15278375|2022-08-16
hCoV-19/New_Zealand/22ZA2332/2022|EPI_ISL_15276300|2022-08-16
hCoV-19/New_Zealand/22ZA2344/2022|EPI_ISL_15276345|2022-08-18
hCoV-19/New_Zealand/22ZA2363/2022|EPI_ISL_15276338|2022-08-17
hCoV-19/New_Zealand/22ZA2402/2022|EPI_ISL_15277462|2022-08-22
hCoV-19/New_Zealand/22ZA2403/2022|EPI_ISL_15277463|2022-08-22
hCoV-19/New_Zealand/22ZA2455/2022|EPI_ISL_15276903|2022-08-23
hCoV-19/New_Zealand/22ZA2468/2022|EPI_ISL_15276390|2022-08-22
hCoV-19/New_Zealand/22ZA2500/2022|EPI_ISL_15276382|2022-08-22
hCoV-19/New_Zealand/22ZA2516/2022|EPI_ISL_15277330|2022-08-24
hCoV-19/New_Zealand/22ZA2523/2022|EPI_ISL_15278088|2022-08-23
hCoV-19/New_Zealand/22ZA2526/2022|EPI_ISL_15278090|2022-08-23
hCoV-19/New_Zealand/22ZA2528/2022|EPI_ISL_15278095|2022-08-23
hCoV-19/New_Zealand/22ZA2553/2022|EPI_ISL_15275837|2022-08-24
hCoV-19/New_Zealand/22ZA2558/2022|EPI_ISL_15275838|2022-08-24
hCoV-19/New_Zealand/22ZA2566/2022|EPI_ISL_15276440|2022-08-23
hCoV-19/New_Zealand/22ZA2580/2022|EPI_ISL_15275842|2022-08-27
hCoV-19/New_Zealand/22ZA2590/2022|EPI_ISL_15277585|2022-08-27
hCoV-19/New_Zealand/22ZA2618/2022|EPI_ISL_15277589|2022-08-28
hCoV-19/New_Zealand/22ZA2652/2022|EPI_ISL_15277601|2022-08-30
hCoV-19/New_Zealand/22ZA2682/2022|EPI_ISL_15278130|2022-08-29
hCoV-19/New_Zealand/22ZA2733/2022|EPI_ISL_15276552|2022-08-30
hCoV-19/New_Zealand/22ZA2746/2022|EPI_ISL_15277607|2022-08-31
hCoV-19/New_Zealand/22ZA2747/2022|EPI_ISL_15277508|2022-08-31
hCoV-19/New_Zealand/22ZA2763/2022|EPI_ISL_15277618|2022-09-04
hCoV-19/New_Zealand/22ZA2885/2022|EPI_ISL_15276674|2022-09-08
hCoV-19/New_Zealand/22ZA2904/2022|EPI_ISL_15277665|2022-09-11

hCoV-19/New_Zealand/22ZA2907/2022|EPI_ISL_15276662|2022-09-07
hCoV-19/New_Zealand/22ZA2947/2022|EPI_ISL_15276715|2022-09-12
hCoV-19/New_Zealand/22ZA2987/2022|EPI_ISL_15277535|2022-09-17
hCoV-19/New_Zealand/22ZA3032/2022|EPI_ISL_15277790|2022-09-21
hCoV-19/New_Zealand/22ZA3106/2022|EPI_ISL_15322800|2022-09-27
hCoV-19/New_Zealand/22ZA3121/2022|EPI_ISL_15322839|2022-09-28
hCoV-19/New_Zealand/22ZA3133/2022|EPI_ISL_15322872|2022-10-01
hCoV-19/New_Zealand/22ZA3140/2022|EPI_ISL_15323507|2022-10-02
hCoV-19/New_Zealand/22ZA3191/2022|EPI_ISL_15958143|2022-10-07
hCoV-19/New_Zealand/22ZA3220/2022|EPI_ISL_15956313|2022-10-08
hCoV-19/New_Zealand/22ZA3280/2022|EPI_ISL_15956343|2022-10-11
hCoV-19/New_Zealand/22ZA3319/2022|EPI_ISL_15956377|2022-10-13
hCoV-19/New_Zealand/22ZA3351/2022|EPI_ISL_15958087|2022-10-17
hCoV-19/New_Zealand/22ZA3425/2022|EPI_ISL_15958496|2022-10-17
hCoV-19/New_Zealand/22ZA3443/2022|EPI_ISL_15958201|2022-10-19
hCoV-19/New_Zealand/22ZA3446/2022|EPI_ISL_15958194|2022-10-19
hCoV-19/New_Zealand/22ZA3489/2022|EPI_ISL_15958097|2022-10-21
hCoV-19/New_Zealand/22ZA3497/2022|EPI_ISL_15958226|2022-10-22
hCoV-19/New_Zealand/22ZA3502/2022|EPI_ISL_15958229|2022-10-23
hCoV-19/New_Zealand/22ZA3503/2022|EPI_ISL_15958230|2022-10-23
hCoV-19/New_Zealand/22ZA3511/2022|EPI_ISL_15958235|2022-10-23
hCoV-19/New_Zealand/22ZA3544/2022|EPI_ISL_15956512|2022-10-23
hCoV-19/New_Zealand/22ZA3551/2022|EPI_ISL_15958080|2022-10-25
hCoV-19/New_Zealand/22ZA3575/2022|EPI_ISL_15956470|2022-10-20
hCoV-19/New_Zealand/22ZA3579/2022|EPI_ISL_15956479|2022-10-20
hCoV-19/New_Zealand/22ZA3618/2022|EPI_ISL_15958263|2022-10-26
hCoV-19/New_Zealand/22ZA3653/2022|EPI_ISL_15958274|2022-10-28
hCoV-19/New_Zealand/22ZA3660/2022|EPI_ISL_15958283|2022-10-29
hCoV-19/New_Zealand/22ZA3661/2022|EPI_ISL_15958284|2022-10-29
hCoV-19/New_Zealand/22ZA3689/2022|EPI_ISL_15956567|2022-10-27
hCoV-19/New_Zealand/22ZA3694/2022|EPI_ISL_15956573|2022-10-27
hCoV-19/New_Zealand/22ZA3724/2022|EPI_ISL_15955854|2022-10-29
hCoV-19/New_Zealand/22ZA3734/2022|EPI_ISL_15958305|2022-11-01
hCoV-19/New_Zealand/22ZA3752/2022|EPI_ISL_15956603|2022-10-28
hCoV-19/New_Zealand/22ZA3768/2022|EPI_ISL_15956617|2022-10-30
hCoV-19/New_Zealand/22ZA3834/2022|EPI_ISL_15956677|2022-11-02
hCoV-19/New_Zealand/22ZA3837/2022|EPI_ISL_15956673|2022-11-01
hCoV-19/New_Zealand/22ZA3863/2022|EPI_ISL_15958348|2022-11-05
hCoV-19/New_Zealand/22ZA3870/2022|EPI_ISL_15958074|2022-11-05
hCoV-19/New_Zealand/22ZA3877/2022|EPI_ISL_15956686|2022-11-02
hCoV-19/New_Zealand/22ZA3915/2022|EPI_ISL_15956720|2022-11-04
hCoV-19/New_Zealand/22ZA3970/2022|EPI_ISL_15956749|2022-11-06
hCoV-19/New_Zealand/22ZA3978/2022|EPI_ISL_15958365|2022-11-08

hCoV-19/New_Zealand/22ZA4044/2022|EPI_ISL_15958388|2022-11-11
hCoV-19/New_Zealand/22ZA4083/2022|EPI_ISL_15956819|2022-11-10
hCoV-19/New_Zealand/22ZA4095/2022|EPI_ISL_15958393|2022-11-13
hCoV-19/New_Zealand/22ZA4098/2022|EPI_ISL_15958398|2022-11-14
hCoV-19/New_Zealand/22ZA4099/2022|EPI_ISL_15958396|2022-11-14
hCoV-19/New_Zealand/22ZA4111/2022|EPI_ISL_15956831|2022-11-10
hCoV-19/New_Zealand/22ZA4125/2022|EPI_ISL_15956848|2022-11-11
hCoV-19/New_Zealand/22ZA4134/2022|EPI_ISL_15956855|2022-11-12
hCoV-19/New_Zealand/22ZA4159/2022|EPI_ISL_15956868|2022-11-13
hCoV-19/New_Zealand/22ZA4254/2022|EPI_ISL_15955891|2022-11-17
hCoV-19/New_Zealand/22ZA4383/2022|EPI_ISL_15956975|2022-11-21
hCoV-19/New_Zealand/22ZA4392/2022|EPI_ISL_15955792|2022-11-16
hCoV-19/New_Zealand/22ZA4439/2022|EPI_ISL_15956984|2022-11-21
hCoV-19/New_Zealand/22ZA4465/2022|EPI_ISL_16050615|2022-11-23
hCoV-19/New_Zealand/22ZA4479/2022|EPI_ISL_16050328|2022-11-22
hCoV-19/New_Zealand/22ZA4493/2022|EPI_ISL_16050248|2022-11-20
hCoV-19/New_Zealand/22ZA4513/2022|EPI_ISL_16050370|2022-11-24
hCoV-19/New_Zealand/22ZA4535/2022|EPI_ISL_16050629|2022-11-25
hCoV-19/New_Zealand/22ZA4542/2022|EPI_ISL_16050631|2022-11-25
hCoV-19/New_Zealand/22ZA4555/2022|EPI_ISL_16050645|2022-11-27
hCoV-19/New_Zealand/22ZA4585/2022|EPI_ISL_16050222|2022-11-25
hCoV-19/New_Zealand/22ZA4600/2022|EPI_ISL_16050211|2022-11-25
hCoV-19/New_Zealand/22ZA4615/2022|EPI_ISL_16050262|2022-11-25
hCoV-19/New_Zealand/22ZA4627/2022|EPI_ISL_16050662|2022-11-28
hCoV-19/New_Zealand/22ZA4634/2022|EPI_ISL_16050671|2022-11-29
hCoV-19/New_Zealand/22ZA4794/2022|EPI_ISL_16435879|2022-12-01
hCoV-19/New_Zealand/22ZA4822/2022|EPI_ISL_16434324|2022-11-30
hCoV-19/New_Zealand/22ZA4830/2022|EPI_ISL_16434274|2022-11-28
hCoV-19/New_Zealand/22ZA4862/2022|EPI_ISL_16435685|2022-12-03
hCoV-19/New_Zealand/22ZA4885/2022|EPI_ISL_16434340|2022-12-01
hCoV-19/New_Zealand/22ZA4907/2022|EPI_ISL_16434388|2022-12-03
hCoV-19/New_Zealand/22ZA4958/2022|EPI_ISL_16434364|2022-12-02
hCoV-19/New_Zealand/22ZA4999/2022|EPI_ISL_16434422|2022-12-05
hCoV-19/New_Zealand/22ZA5030/2022|EPI_ISL_16433973|2022-12-06
hCoV-19/New_Zealand/22ZA5032/2022|EPI_ISL_16435939|2022-12-06
hCoV-19/New_Zealand/22ZA5051/2022|EPI_ISL_16435322|2022-12-05
hCoV-19/New_Zealand/22ZA5154/2022|EPI_ISL_16435731|2022-12-08
hCoV-19/New_Zealand/22ZA5177/2022|EPI_ISL_16434536|2022-12-08
hCoV-19/New_Zealand/22ZA5193/2022|EPI_ISL_16434558|2022-12-10
hCoV-19/New_Zealand/22ZA5210/2022|EPI_ISL_16434167|2022-12-08
hCoV-19/New_Zealand/22ZA5221/2022|EPI_ISL_16434179|2022-12-09
hCoV-19/New_Zealand/22ZA5271/2022|EPI_ISL_16435701|2022-12-12
hCoV-19/New_Zealand/22ZA5279/2022|EPI_ISL_16435709|2022-12-12

hCoV-19/New_Zealand/22ZA5283/2022|EPI_ISL_16435326|2022-12-08
hCoV-19/New_Zealand/22ZA5296/2022|EPI_ISL_16434547|2022-12-09
hCoV-19/New_Zealand/22ZA5332/2022|EPI_ISL_16433836|2022-12-10
hCoV-19/New_Zealand/22ZA5347/2022|EPI_ISL_16435758|2022-12-13
hCoV-19/New_Zealand/22ZA5373/2022|EPI_ISL_16436100|2022-12-08
hCoV-19/New_Zealand/22ZA5385/2022|EPI_ISL_16436107|2022-12-09
hCoV-19/New_Zealand/22ZA5427/2022|EPI_ISL_16433997|2022-12-13
hCoV-19/New_Zealand/22ZA5429/2022|EPI_ISL_16434000|2022-12-13
hCoV-19/New_Zealand/22ZA5461/2022|EPI_ISL_16434607|2022-12-12
hCoV-19/New_Zealand/22ZA5480/2022|EPI_ISL_16434597|2022-12-12
hCoV-19/New_Zealand/22ZA5498/2022|EPI_ISL_16433995|2022-12-13
hCoV-19/New_Zealand/22ZA5547/2022|EPI_ISL_16434667|2022-12-14
hCoV-19/New_Zealand/22ZA5551/2022|EPI_ISL_16434662|2022-12-14
hCoV-19/New_Zealand/22ZA5595/2022|EPI_ISL_16434011|2022-12-15
hCoV-19/New_Zealand/22ZA5604/2022|EPI_ISL_16435810|2022-12-15
hCoV-19/New_Zealand/22ZA5611/2022|EPI_ISL_16435812|2022-12-16
hCoV-19/New_Zealand/22ZA5622/2022|EPI_ISL_16434696|2022-12-14
hCoV-19/New_Zealand/22ZA5674/2022|EPI_ISL_16435669|2022-12-17
hCoV-19/New_Zealand/22ZA5699/2022|EPI_ISL_16434030|2022-12-18
hCoV-19/New_Zealand/22ZA5706/2022|EPI_ISL_16434698|2022-12-14
hCoV-19/New_Zealand/22ZA5715/2022|EPI_ISL_16434703|2022-12-15
hCoV-19/New_Zealand/22ZA5725/2022|EPI_ISL_16434736|2022-12-16
hCoV-19/New_Zealand/22ZA5727/2022|EPI_ISL_16434734|2022-12-16
hCoV-19/New_Zealand/22ZA5747/2022|EPI_ISL_16434021|2022-12-16
hCoV-19/New_Zealand/22ZA5769/2022|EPI_ISL_16436216|2022-12-20
hCoV-19/New_Zealand/22ZA5783/2022|EPI_ISL_16952669|2022-12-19
hCoV-19/New_Zealand/22ZA5793/2022|EPI_ISL_16952678|2022-12-20
hCoV-19/New_Zealand/22ZA5799/2022|EPI_ISL_16952680|2022-12-20
hCoV-19/New_Zealand/23XA0007/2022|EPI_ISL_16954428|2022-12-30
hCoV-19/New_Zealand/23XA0083/2022|EPI_ISL_16953095|2022-12-22
hCoV-19/New_Zealand/23XA0222/2022|EPI_ISL_16954404|2022-12-24
hCoV-19/New_Zealand/23XA0328/2022|EPI_ISL_16952658|2022-12-23
hCoV-19/New_Zealand/23XA0575/2022|EPI_ISL_16952522|2022-12-28
hCoV-19/New_Zealand/23YA0016/2022|EPI_ISL_16952721|2022-12-29
hCoV-19/New_Zealand/23YA0044/2022|EPI_ISL_16952684|2022-12-22
hCoV-19/New_Zealand/23YA0070/2022|EPI_ISL_16952830|2022-12-30
hCoV-19/New_Zealand/23YA0128/2022|EPI_ISL_16952794|2022-12-25
hCoV-19/New_Zealand/23YA0133/2022|EPI_ISL_16952799|2022-12-26
hCoV-19/New_Zealand/23YA0142/2022|EPI_ISL_16952807|2022-12-27
hCoV-19/New_Zealand/23YA0195/2022|EPI_ISL_16952782|2022-12-22
hCoV-19/New_Zealand/23ZA0006/2022|EPI_ISL_16953067|2022-12-30
hCoV-19/New_Zealand/23ZA0090/2022|EPI_ISL_16952657|2022-12-31
hCoV-19/New_Zealand/23ZA0177/2022|EPI_ISL_16952645|2022-12-22

hCoV-19/New_Zealand/23ZA0190/2022|EPI_ISL_16952967|2022-12-26
hCoV-19/New_Zealand/23ZA0225/2022|EPI_ISL_16952702|2022-12-26
hCoV-19/New_Zealand/23ZA0242/2022|EPI_ISL_16952505|2022-12-22
hCoV-19/New_Zealand/23ZA0252/2022|EPI_ISL_16952528|2022-12-31
hCoV-19/New_Zealand/23ZA0483/2022|EPI_ISL_16952652|2022-12-28
hCoV-19/New_Zealand/23ZA0518/2022|EPI_ISL_16952713|2022-12-28
hCoV-19/New_Zealand/23ZA0544/2022|EPI_ISL_16952955|2022-12-28
hCoV-19/New_Zealand/23ZA0561/2022|EPI_ISL_16953107|2022-12-28
hCoV-19/Nigeria/ISTH-E0195/2020|EPI_ISL_14205933|2020-12-18
hCoV-19/Northern_Ireland/QEUH-965194/2020|EPI_ISL_531134|2020-08-13
hCoV-19/Norway/10252/2022|EPI_ISL_11262873|2022-02-17
hCoV-19/Norway/12251/2022|EPI_ISL_13181820|2022-03-02
hCoV-19/Norway/12761/2022|EPI_ISL_11323933|2022-03-02
hCoV-19/Norway/12974/2022|EPI_ISL_13181325|2022-03-01
hCoV-19/Norway/13028/2022|EPI_ISL_11661008|2022-03-02
hCoV-19/Norway/13198/2022|EPI_ISL_11661091|2022-03-02
hCoV-19/Norway/13695/2022|EPI_ISL_12982076|2022-03-01
hCoV-19/Norway/15277/2022|EPI_ISL_12540286|2022-03-17
hCoV-19/Norway/16461/2022|EPI_ISL_12439667|2022-03-17
hCoV-19/Norway/16571/2022|EPI_ISL_12360788|2022-03-17
hCoV-19/Norway/18191/2021|EPI_ISL_17206949|2021-08-15
hCoV-19/Norway/19481/2022|EPI_ISL_12449719|2022-04-12
hCoV-19/Norway/20133/2022|EPI_ISL_12809744|2022-04-12
hCoV-19/Norway/21644/2021|EPI_ISL_17207647|2021-09-16
hCoV-19/Norway/21719/2022|EPI_ISL_12983129|2022-05-02
hCoV-19/Norway/21720/2022|EPI_ISL_12983130|2022-05-02
hCoV-19/Norway/23207/2022|EPI_ISL_13406808|2022-05-27
hCoV-19/Norway/23446/2022|EPI_ISL_13328013|2022-05-27
hCoV-19/Norway/2386/2020|EPI_ISL_449791|2020-04-04
hCoV-19/Norway/23884/2021|EPI_ISL_17207184|2021-10-15
hCoV-19/Norway/24027/2022|EPI_ISL_13636983|2022-05-27
hCoV-19/Norway/24278/2022|EPI_ISL_13636886|2022-06-09
hCoV-19/Norway/26158/2021|EPI_ISL_17207693|2021-11-02
hCoV-19/Norway/30134/2022|EPI_ISL_14892153|2022-08-16
hCoV-19/Norway/3112/2020|EPI_ISL_549135|2020-08-05
hCoV-19/Norway/33633/2021|EPI_ISL_17206788|2021-12-10
hCoV-19/Norway/5580/2022|EPI_ISL_10351488|2022-02-04
hCoV-19/Norway/5708/2022|EPI_ISL_10351524|2022-02-05
hCoV-19/Norway/6266/2022|EPI_ISL_10583442|2022-02-04
hCoV-19/Norway/6587/2022|EPI_ISL_10764231|2022-02-05
hCoV-19/Norway/6980/2022|EPI_ISL_10583370|2022-02-01
hCoV-19/Norway/7158/2020|EPI_ISL_17207201|2020-12-14
hCoV-19/Norway/7331/2020|EPI_ISL_17207690|2020-12-26

hCoV-19/Norway/7543/2022|EPI_ISL_12649867|2022-02-01
hCoV-19/Norway/8205/2021|EPI_ISL_17206897|2021-03-15
hCoV-19/Norway/9361/2022|EPI_ISL_10849645|2022-02-17
hCoV-19/Norway/Ahus-4146/2022|EPI_ISL_14146019|2022-07-18
hCoV-19/Norway/OUS-20734/2022|EPI_ISL_9747233|2022-02-01
hCoV-19/Norway/OUS-22001/2022|EPI_ISL_11067797|2022-03-01
hCoV-19/Norway/OUS-22015/2022|EPI_ISL_11067808|2022-03-01
hCoV-19/Norway/OUS-25210/2022|EPI_ISL_12983055|2022-05-03
hCoV-19/Pakistan/KRISS0890/2021|EPI_ISL_15172228|2021-03-26
hCoV-19/Panama/CDED23068-GMI/2022|EPI_ISL_16027399|2022-05-28
hCoV-19/Panama/CDED23100-GMI/2022|EPI_ISL_16027401|2022-05-30
hCoV-19/Panama/CDED23898-GMI/2022|EPI_ISL_16027404|2022-08-10
hCoV-19/Panama/CDED23911-GMI/2022|EPI_ISL_16027405|2022-08-13
hCoV-19/Panama/M220569-GMI/2022|EPI_ISL_11859503|2022-03-16
hCoV-19/Paraguay/469967/2022|EPI_ISL_14854416|2022-06-10
hCoV-19/Peru/CAL-CITBM-C18m08/2021|EPI_ISL_15572305|2021-10-14
hCoV-19/Philippines/PGCV_0030_2785/2022|EPI_ISL_15636296|2022-08-18
hCoV-19/Philippines/PGCV_0031_3049/2022|EPI_ISL_15636308|2022-08-22
hCoV-19/Philippines/PGCV_0032_3204/2022|EPI_ISL_15636314|2022-08-29
hCoV-19/Philippines/PH-PGC-110497/2022|EPI_ISL_13170859|2022-02-01
hCoV-19/Philippines/PH-VUI-140746/2022|EPI_ISL_16019717|2022-10-27
hCoV-19/Philippines/PH-VUI-140758/2022|EPI_ISL_16019710|2022-10-27
hCoV-19/Poland/22SNR2704_wsserze/2022|EPI_ISL_12812405|2022-05-03
hCoV-19/Poland/PL_P1328/2022|EPI_ISL_12614709|2022-02-16
hCoV-19/Poland/PL_P791/2022|EPI_ISL_9981872|2022-02-02
hCoV-19/Poland/PZH-GUM-12034/2022|EPI_ISL_10115059|2022-02-03
hCoV-19/Poland/PZH-GUM-12451/2022|EPI_ISL_10720036|2022-02-01
hCoV-19/Poland/PZH-GUM-12502/2022|EPI_ISL_10720045|2022-02-01
hCoV-19/Poland/PZH-GUM-12579/2022|EPI_ISL_10720067|2022-02-02
hCoV-19/Poland/PZH-GUM-12615/2022|EPI_ISL_10720076|2022-02-01
hCoV-19/Poland/PZH-GUM-12617/2022|EPI_ISL_10720077|2022-02-03
hCoV-19/Poland/PZH-GUM-13659/2022|EPI_ISL_10505242|2022-02-03
hCoV-19/Poland/PZH-GUM-13965/2022|EPI_ISL_10505833|2022-02-17
hCoV-19/Poland/PZH-GUM-14461/2022|EPI_ISL_10847305|2022-02-16
hCoV-19/Poland/PZH-GUM-15290/2022|EPI_ISL_10957706|2022-02-16
hCoV-19/Poland/PZH-UMB-18025/2022|EPI_ISL_14605037|2022-08-09
hCoV-19/Portugal/PT0261/2020|EPI_ISL_453975|2020-04-03
hCoV-19/Portugal/PT1762/2020|EPI_ISL_731996|2020-11-15
hCoV-19/Portugal/PT32522/2022|EPI_ISL_12289711|2022-04-12
hCoV-19/Portugal/PT40987/2022|EPI_ISL_14576588|2022-08-09
hCoV-19/Portugal/PT44394/2022|EPI_ISL_16073725|2022-11-26
hCoV-19/Puerto_Rico/PR-UPRRP-527/2020|EPI_ISL_15592454|2020-12-16
hCoV-19/Puerto_Rico/PR-UPRRP-603/2020|EPI_ISL_15592632|2020-12-11

hCoV-19/Qatar/QA.QU_18.51.A11/2022|EPI_ISL_16076568|2022-06-18
hCoV-19/Qatar/QA.QU_18.51.A9/2022|EPI_ISL_16076565|2022-06-18
hCoV-19/Qatar/QA.QU_18.51.B2/2022|EPI_ISL_16076570|2022-06-18
hCoV-19/Qatar/QA.QU_18.51.E8/2022|EPI_ISL_16076572|2022-06-19
hCoV-19/Qatar/QU.18.64.F7/2022|EPI_ISL_15922739|2022-09-21
hCoV-19/Qatar/QU.18.65.F2/2022|EPI_ISL_15922810|2022-09-28
hCoV-19/Qatar/QU.18.65.G10/2022|EPI_ISL_15922803|2022-09-28
hCoV-19/Qatar/QU.18.65.G8/2022|EPI_ISL_15922814|2022-09-28
hCoV-19/Qatar/QU.18.68.C9/2022|EPI_ISL_15922937|2022-10-18
hCoV-19/Qatar/QU.18.68.H5/2022|EPI_ISL_15922931|2022-10-17
hCoV-19/Reunion/CHU722052633703/2022|EPI_ISL_13767487|2022-05-28
hCoV-19/Reunion/ChuReu-722052633703/2022|EPI_ISL_13909186|2022-05-28
hCoV-19/Reunion/ChuReu-722060317001/2022|EPI_ISL_13909471|2022-05-25
hCoV-19/Reunion/ChuReu-722060318201/2022|EPI_ISL_13909675|2022-05-25
hCoV-19/Reunion/ChuReu-722060324401/2022|EPI_ISL_13909482|2022-05-27
hCoV-19/Reunion/ChuReu-722061747501/2022|EPI_ISL_13910295|2022-06-08
hCoV-19/Reunion/ChuReu-722061802401/2022|EPI_ISL_13838131|2022-06-09
hCoV-19/Reunion/ChuReu-722061815101/2022|EPI_ISL_13910432|2022-06-11
hCoV-19/Reunion/ChuReu-722062009101/2022|EPI_ISL_13838342|2022-06-10
hCoV-19/Reunion/ChuReu-722091252901/2022|EPI_ISL_15777978|2022-08-30
hCoV-19/Reunion/LRE-ChuReu-722031778101/2022|EPI_ISL_11540310|2022-03-17
hCoV-19/Reunion/LRE-ChuReu-722031779001/2022|EPI_ISL_11540232|2022-03-17
hCoV-19/Reunion/PIMIT_10604/2021|EPI_ISL_3154181|2021-05-22
hCoV-19/Romania/B-16467/2022|EPI_ISL_12603339|2022-03-17
hCoV-19/Romania/IS-20642/2022|EPI_ISL_15488978|2022-05-27
hCoV-19/Romania/IS-22971/2022|EPI_ISL_15489109|2022-07-19
hCoV-19/Romania/MS-16704/2022|EPI_ISL_12603317|2022-03-16
hCoV-19/Romania/MS-24545/2022|EPI_ISL_15489596|2022-08-05
hCoV-19/Romania/MS-25027/2022|EPI_ISL_15489648|2022-08-12
hCoV-19/Romania/MS-26080/2022|EPI_ISL_15489743|2022-09-02
hCoV-19/Romania/MS-26096/2022|EPI_ISL_15489759|2022-09-05
hCoV-19/Russia/AL-RII-MH117472S/2022|EPI_ISL_16053192|2022-09-09
hCoV-19/Russia/LIP-CRIE-7785738572/2022|EPI_ISL_15895449|2022-10-25
hCoV-19/Russia/MOS-RII-MH116083S/2022|EPI_ISL_16008489|2022-11-18
hCoV-19/Russia/MOW-CRIE-7765194914/2022|EPI_ISL_14423670|2022-07-27
hCoV-19/Russia/MOW-CSP-KOM-148_S67/2020|EPI_ISL_8904859|2020-09-08
hCoV-19/Russia/MOW-CSP-SK-566_S67/2020|EPI_ISL_8908162|2020-10-19
hCoV-19/Russia/MOW-PMVL-DZ-133534800/2021|EPI_ISL_15859105|2021-08-04
hCoV-19/Russia/MOW-PMVL-DZ-24052565493/2022|EPI_ISL_15862920|2022-07-25
hCoV-19/Russia/MOW-PMVL-DZ-29833283788/2022|EPI_ISL_15862535|2022-07-21
hCoV-19/Russia/MOW-PMVL-DZ-36763307163/2021|EPI_ISL_15868891|2021-07-31
hCoV-19/Russia/MOW-PMVL-DZ-3905172/2021|EPI_ISL_15858526|2021-06-28
hCoV-19/Russia/MOW-PMVL-DZ-9795670434/2021|EPI_ISL_15874955|2021-11-24

hCoV-19/Russia/MOW-RII-MH116092S/2022|EPI_ISL_16008497|2022-11-18
hCoV-19/Russia/MOW-RII-MH117155S/2022|EPI_ISL_16053009|2022-11-28
hCoV-19/Russia/MOW-RII-MH117192S/2022|EPI_ISL_16053027|2022-11-28
hCoV-19/Russia/PRI-6033/2021|EPI_ISL_16641835|2021-02-11
hCoV-19/Russia/PRI-8266/2021|EPI_ISL_17562483|2021-10-26
hCoV-19/Russia/SAM-RII-MH116280S/2022|EPI_ISL_16008588|2022-11-21
hCoV-19/Russia/SAM-RII-MH116283S/2022|EPI_ISL_16008589|2022-11-22
hCoV-19/Russia/SAM-RII-MH116287S/2022|EPI_ISL_16008593|2022-11-21
hCoV-19/Russia/SAM-RII-MH116294S/2022|EPI_ISL_16008598|2022-11-18
hCoV-19/Russia/SAM-RII-MH117169S/2022|EPI_ISL_16053014|2022-11-26
hCoV-19/Russia/SPE-CSP-154_S20/2020|EPI_ISL_8908239|2020-08-18
hCoV-19/Russia/SPE-RII-MH70940S/2022|EPI_ISL_14596219|2022-07-14
hCoV-19/Russia/SPE-RII-MH71235S/2022|EPI_ISL_14596274|2022-07-19
hCoV-19/Russia/SPE-RII-MH72002S/2022|EPI_ISL_14909167|2022-07-21
hCoV-19/Russia/SVE-RII-MH116318S/2022|EPI_ISL_16008602|2022-10-13
hCoV-19/Russia/SVE-RII-MH116320S/2022|EPI_ISL_16008604|2022-10-09
hCoV-19/Russia/SVE-RII-MH116351S/2022|EPI_ISL_16008609|2022-10-28
hCoV-19/Russia/SVE-RII-MH116360S/2022|EPI_ISL_16008608|2022-10-10
hCoV-19/Saudi_Arabia/KAUST489/2021|EPI_ISL_16675001|2021-12-30
hCoV-19/Scotland/EDB3846/2020|EPI_ISL_439337|2020-05-01
hCoV-19/Scotland/EDB4453/2020|EPI_ISL_453129|2020-05-05
hCoV-19/Scotland/EDB578/2020|EPI_ISL_433202|2020-04-01
hCoV-19/Scotland/QEUH-37794BB/2022|EPI_ISL_10337626|2022-02-17
hCoV-19/Scotland/QEUH-89DB2D/2020|EPI_ISL_532697|2020-07-28
hCoV-19/Scotland/QEUH-960C10/2020|EPI_ISL_531979|2020-08-06
hCoV-19/Scotland/QEUH-9C8760/2020|EPI_ISL_576017|2020-09-15
hCoV-19/Shaoxing/02/2020|EPI_ISL_463894|2020-01-30
hCoV-19/Sichuan/SC-NC-071/2020|EPI_ISL_451330|2020-01-29
hCoV-19/Sichuan/SC-WCH1-005/2020|EPI_ISL_451376|2020-01-28
hCoV-19/Sichuan/SC-WCH3-255/2020|EPI_ISL_451384|2020-01-27
hCoV-19/Sierra_Leone/nmimr-SLE--SLSEQ067/2021|EPI_ISL_15283215|2021-01-28
hCoV-19/Singapore/1214/2022|EPI_ISL_9596884|2022-02-01
hCoV-19/Singapore/14058/2022|EPI_ISL_15593912|2022-10-26
hCoV-19/Singapore/1449/2022|EPI_ISL_9847967|2022-02-05
hCoV-19/Singapore/2838/2022|EPI_ISL_11250682|2022-03-01
hCoV-19/Singapore/87/2020|EPI_ISL_435692|2020-04-21
hCoV-19/Singapore/8728/2022|EPI_ISL_14016440|2022-07-15
hCoV-19/Singapore/9442/2022|EPI_ISL_14358964|2022-07-30
hCoV-19/Singapore/9450/2022|EPI_ISL_14358972|2022-07-30
hCoV-19/Slovakia/BA_22_00020170/2022|EPI_ISL_11798970|2022-03-16
hCoV-19/Slovenia/152795/2022|EPI_ISL_11546349|2022-02-01
hCoV-19/Slovenia/17-033985-CE/2020|EPI_ISL_7242685|2020-10-21
hCoV-19/Slovenia/170840/2022|EPI_ISL_11612494|2022-03-01

hCoV-19/Slovenia/177983/2022|EPI_ISL_12285166|2022-03-16
hCoV-19/Slovenia/178009/2022|EPI_ISL_12284913|2022-03-16
hCoV-19/Slovenia/21-8537/2020|EPI_ISL_9464115|2020-09-29
hCoV-19/Slovenia/211601/2022|EPI_ISL_14962052|2022-07-14
hCoV-19/Slovenia/211747/2022|EPI_ISL_14962073|2022-07-14
hCoV-19/Slovenia/212379/2022|EPI_ISL_14962166|2022-07-17
hCoV-19/Slovenia/212909/2022|EPI_ISL_14962240|2022-07-19
hCoV-19/Slovenia/213091/2022|EPI_ISL_14962264|2022-07-20
hCoV-19/Slovenia/213131/2022|EPI_ISL_14962270|2022-07-20
hCoV-19/Slovenia/215032/2022|EPI_ISL_14962563|2022-07-30
hCoV-19/Slovenia/215680/2022|EPI_ISL_14951496|2022-08-02
hCoV-19/Slovenia/215816/2022|EPI_ISL_14951512|2022-08-03
hCoV-19/Slovenia/215942/2022|EPI_ISL_14951526|2022-08-04
hCoV-19/Slovenia/216271/2022|EPI_ISL_14951554|2022-08-06
hCoV-19/Slovenia/217260/2022|EPI_ISL_14953464|2022-08-11
hCoV-19/Slovenia/217557/2022|EPI_ISL_14953471|2022-08-13
hCoV-19/Slovenia/218997/2022|EPI_ISL_14952029|2022-08-21
hCoV-19/Slovenia/220263/2022|EPI_ISL_14962705|2022-08-27
hCoV-19/Slovenia/220267/2022|EPI_ISL_14962706|2022-08-27
hCoV-19/Slovenia/279182/2020|EPI_ISL_14724859|2020-12-17
hCoV-19/South_Africa/KRISP-012/2020|EPI_ISL_421576|2020-04-01
hCoV-19/South_Africa/KRISP-CC00689995/2020|EPI_ISL_736934|2020-11-25
hCoV-19/South_Africa/KRISP-K002210/2020|EPI_ISL_515607|2020-07-24
hCoV-19/South_Africa/NCV1365/2020|EPI_ISL_12651479|2020-09-02
hCoV-19/South_Africa/NCV1709/2020|EPI_ISL_16064201|2020-07-28
hCoV-19/South_Africa/NHLS-UCT-GS-8235/2020|EPI_ISL_16438056|2020-12-17
hCoV-19/South_Africa/NICD-A265-003-210/2021|EPI_ISL_16078530|2021-08-16
hCoV-19/South_Africa/NICD-K212-001-190/2021|EPI_ISL_16078626|2021-06-08
hCoV-19/South_Africa/VIDA-KRISP-CP157502837/2020|EPI_ISL_1132711|2020-12-15
hCoV-19/South_Korea/Chosun-CoV15_NP/2020|EPI_ISL_1007669|2020-02-25
hCoV-19/South_Korea/GJ-HERI-5138/2021|EPI_ISL_15016225|2021-10-07
hCoV-19/South_Korea/KCDC2042/2020|EPI_ISL_471448|2020-02-19
hCoV-19/South_Korea/KCDC2725/2020|EPI_ISL_522504|2020-07-13
hCoV-19/South_Korea/KDCA0424/2020|EPI_ISL_760208|2020-11-14
hCoV-19/South_Korea/KDCA112656/2022|EPI_ISL_14855592|2022-08-19
hCoV-19/South_Korea/KDCA130635/2022|EPI_ISL_15351736|2022-09-21
hCoV-19/South_Korea/KDCA163892/2022|EPI_ISL_15979523|2022-11-11
hCoV-19/South_Korea/KDCA163894/2022|EPI_ISL_15979525|2022-11-11
hCoV-19/South_Korea/KDCA163906/2022|EPI_ISL_15979537|2022-11-11
hCoV-19/South_Korea/KDCA163907/2022|EPI_ISL_15979538|2022-11-11
hCoV-19/South_Korea/KDCA164122/2022|EPI_ISL_15979750|2022-11-11
hCoV-19/South_Korea/KDCA164129/2022|EPI_ISL_15979757|2022-11-11
hCoV-19/South_Korea/KDCA164130/2022|EPI_ISL_15979758|2022-11-11

hCoV-19/South_Korea/KDCA164136/2022|EPI_ISL_15979762|2022-11-11
hCoV-19/South_Korea/KDCA164137/2022|EPI_ISL_15979763|2022-11-11
hCoV-19/South_Korea/KDCA164427/2022|EPI_ISL_15980022|2022-11-13
hCoV-19/South_Korea/KDCA164429/2022|EPI_ISL_15980024|2022-11-13
hCoV-19/South_Korea/KDCA164433/2022|EPI_ISL_15980028|2022-11-13
hCoV-19/South_Korea/KDCA164434/2022|EPI_ISL_15980029|2022-11-13
hCoV-19/South_Korea/KDCA164435/2022|EPI_ISL_15980030|2022-11-13
hCoV-19/South_Korea/KDCA164648/2022|EPI_ISL_15980241|2022-11-13
hCoV-19/South_Korea/KDCA164650/2022|EPI_ISL_15980243|2022-11-13
hCoV-19/South_Korea/KDCA164651/2022|EPI_ISL_15980244|2022-11-13
hCoV-19/South_Korea/KDCA164654/2022|EPI_ISL_15980247|2022-11-13
hCoV-19/South_Korea/KDCA164655/2022|EPI_ISL_15980248|2022-11-13
hCoV-19/South_Korea/KDCA34780/2022|EPI_ISL_12129714|2022-03-16
hCoV-19/South_Korea/KDCA39231/2022|EPI_ISL_12414769|2022-04-12
hCoV-19/South_Korea/KDCA71808/2022|EPI_ISL_13353202|2022-05-25
hCoV-19/South_Korea/KDCA72014/2022|EPI_ISL_13353397|2022-05-26
hCoV-19/South_Korea/KDCA72079/2022|EPI_ISL_13353456|2022-05-27
hCoV-19/South_Korea/KDCA76204/2022|EPI_ISL_13389088|2022-05-25
hCoV-19/South_Korea/KDCA81331/2022|EPI_ISL_13571917|2022-06-09
hCoV-19/South_Korea/KDCA81368/2022|EPI_ISL_13571951|2022-06-10
hCoV-19/South_Korea/KDCA86647/2022|EPI_ISL_13690247|2022-06-10
hCoV-19/South_Korea/KDCA92635/2022|EPI_ISL_14137041|2022-07-13
hCoV-19/South_Korea/KDCA94470/2022|EPI_ISL_14255115|2022-07-13
hCoV-19/South_Korea/KDCA94496/2022|EPI_ISL_14255138|2022-07-14
hCoV-19/South_Korea/KDCA94734/2022|EPI_ISL_14255346|2022-07-17
hCoV-19/South_Korea/KDCA94778/2022|EPI_ISL_14255383|2022-07-18
hCoV-19/Spain/AN-CBA-00856/2021|EPI_ISL_15629942|2021-02-25
hCoV-19/Spain/AN-CBA-03579/2021|EPI_ISL_15649542|2021-04-12
hCoV-19/Spain/AN-CBA-07387/2021|EPI_ISL_15681367|2021-07-20
hCoV-19/Spain/AN-CBA-08512/2021|EPI_ISL_15741973|2021-03-25
hCoV-19/Spain/AN-CBA-08548/2021|EPI_ISL_15742002|2021-06-22
hCoV-19/Spain/AN-CBA-08863/2021|EPI_ISL_15685405|2021-07-28
hCoV-19/Spain/AN-CBA-10589/2021|EPI_ISL_15742359|2021-07-25
hCoV-19/Spain/AN-CBA-10891/2021|EPI_ISL_15698073|2021-07-23
hCoV-19/Spain/AN-CBA-13411/2021|EPI_ISL_15713522|2021-09-25
hCoV-19/Spain/AN-CBA-14901/2021|EPI_ISL_16126147|2021-10-31
hCoV-19/Spain/AN-CBA-17199/2021|EPI_ISL_16177104|2021-11-29
hCoV-19/Spain/AS-252252535/2022|EPI_ISL_14148258|2022-07-17
hCoV-19/Spain/AS-252252538/2022|EPI_ISL_14148259|2022-07-17
hCoV-19/Spain/AS-252255721/2022|EPI_ISL_14244319|2022-07-24
hCoV-19/Spain/AS-252260932/2022|EPI_ISL_14480701|2022-08-04
hCoV-19/Spain/AS-252269420/2022|EPI_ISL_15022734|2022-08-29
hCoV-19/Spain/AS-252269763/2022|EPI_ISL_15022739|2022-08-29

hCoV-19/Spain/AS-252276179/2022|EPI_ISL_15287450|2022-09-22

hCoV-19/Spain/CL-28062/2022|EPI_ISL_12351738|2022-03-17

hCoV-19/Spain/CL-32037/2022|EPI_ISL_14729307|2022-05-27

hCoV-19/Spain/CL-32314/2022|EPI_ISL_14729365|2022-06-08

hCoV-19/Spain/CL-32459/2022|EPI_ISL_14728967|2022-06-09

hCoV-19/Spain/CL-32486/2022|EPI_ISL_14729414|2022-06-08

hCoV-19/Spain/CL-33181/2022|EPI_ISL_14727566|2022-05-27

hCoV-19/Spain/CL-COV31777/2022|EPI_ISL_13301024|2022-05-25

hCoV-19/Spain/CT-HUB06452/2022|EPI_ISL_15021544|2022-07-14

hCoV-19/Spain/CT-LabRefCat-7001648/2022|EPI_ISL_12628082|2022-05-01

hCoV-19/Spain/GA-CHUVI-19069153/2020|EPI_ISL_16894290|2020-12-03

hCoV-19/Spain/GA-CHUVI-19269221c-2/2021|EPI_ISL_17621823|2021-04-14

hCoV-19/Spain/GA-CHUVI-19369236c-2/2021|EPI_ISL_17621844|2021-06-29

hCoV-19/Spain/GA-CHUVI-19533461/2022|EPI_ISL_12130743|2022-03-17

hCoV-19/Spain/GA-CHUVI-19992004c-2/2021|EPI_ISL_17621868|2021-01-05

hCoV-19/Spain/GA-CHUVI-33643400/2020|EPI_ISL_16579975|2020-12-26

hCoV-19/Spain/GA-CHUVI-33838795c-2/2021|EPI_ISL_17621872|2021-03-26

hCoV-19/Spain/GA-CHUVI-34979511/2022|EPI_ISL_15728914|2022-09-24

hCoV-19/Spain/MD-HGUGM-6718636/2022|EPI_ISL_16013799|2022-11-21

hCoV-19/Spain/MD-HGUGM-6719497/2022|EPI_ISL_16013800|2022-11-25

hCoV-19/Spain/MD-ISCIII-222544/2022|EPI_ISL_15156947|2022-05-26

hCoV-19/Spain/MD-ISCIII-222584/2022|EPI_ISL_15156949|2022-05-29

hCoV-19/Spain/ML-ISCIII-222559/2022|EPI_ISL_15156781|2022-05-26

hCoV-19/Spain/PV-HUB-56592455/2020|EPI_ISL_489834|2020-06-22

hCoV-19/Spain/PV-HUD-70124295/2022|EPI_ISL_14844882|2022-08-16

hCoV-19/Spain/PV-HUG267/2022|EPI_ISL_15021937|2022-08-03

hCoV-19/Spain/VC-CVA_ALIC-113069/2022|EPI_ISL_15030518|2022-07-20

hCoV-19/Spain/VC-CVA_ALIC-113095/2022|EPI_ISL_15030543|2022-07-18

hCoV-19/Spain/VC-CVA_ALIC-113186/2022|EPI_ISL_15030632|2022-08-05

hCoV-19/Spain/VC-CVA_ALIC-113207/2022|EPI_ISL_15030652|2022-07-23

hCoV-19/Spain/VC-CVA_ALIC-113218/2022|EPI_ISL_15030663|2022-08-09

hCoV-19/Spain/VC-CVA_ALIC-113291/2022|EPI_ISL_15030726|2022-08-17

hCoV-19/Spain/VC-CVA_ALIC-114497/2022|EPI_ISL_15317879|2022-08-27

hCoV-19/Spain/VC-CVA_ALIC-115499/2022|EPI_ISL_15975385|2022-10-23

hCoV-19/Spain/VC-CVA_ALIC-115501/2022|EPI_ISL_15975387|2022-11-02

hCoV-19/Spain/VC-CVA_ALIC-115503/2022|EPI_ISL_15975389|2022-11-03

hCoV-19/Spain/VC-CVA_ALIC-115505/2022|EPI_ISL_15975391|2022-11-03

hCoV-19/Spain/VC-CVA_ALIC-115507/2022|EPI_ISL_15975393|2022-11-04

hCoV-19/Spain/VC-CVA_CAST-113829/2022|EPI_ISL_15032090|2022-08-04

hCoV-19/Spain/VC-CVA_CAST-113847/2022|EPI_ISL_15032108|2022-08-09

hCoV-19/Spain/VC-CVA_CLIN-114903/2022|EPI_ISL_15618498|2022-08-03

hCoV-19/Spain/VC-CVA_GRAL-113681/2022|EPI_ISL_15031861|2022-08-10

hCoV-19/Spain/VC-CVA_GRAL-113752/2022|EPI_ISL_15032206|2022-07-24

hCoV-19/Spain/VC-CVA_GRAL-114391/2022|EPI_ISL_15388120|2022-08-26
hCoV-19/Spain/VC-CVA_GRAL-114409/2022|EPI_ISL_15388081|2022-09-02
hCoV-19/Spain/VC-CVA_GRAL-114968/2022|EPI_ISL_15688086|2022-10-18
hCoV-19/Spain/VC-CVA_GRAL-115605/2022|EPI_ISL_15993535|2022-11-07
hCoV-19/Spain/VC-CVA_GRAL-115608/2022|EPI_ISL_15993537|2022-11-08
hCoV-19/Spain/VC-CVA_GRAL-115613/2022|EPI_ISL_15993539|2022-11-09
hCoV-19/Spain/VC-CVA_GRAL-115615/2022|EPI_ISL_15993541|2022-11-09
hCoV-19/Spain/VC-CVA_GRAL-115618/2022|EPI_ISL_15993543|2022-11-11
hCoV-19/Spain/VC-CVA_LAFE-113420/2022|EPI_ISL_15031648|2022-07-14
hCoV-19/Spain/VC-FISABIO-109143/2022|EPI_ISL_15498427|2022-05-29
hCoV-19/Spain/VC-FISABIO-109631/2022|EPI_ISL_15498479|2022-06-09
hCoV-19/Spain/VC-FISABIO-112189/2022|EPI_ISL_15030264|2022-07-22
hCoV-19/Spain/VC-FISABIO-112226/2022|EPI_ISL_15030299|2022-07-25
hCoV-19/Spain/VC-FISABIO-112903/2022|EPI_ISL_15287416|2022-08-03
hCoV-19/Spain/VC-FISABIO-113908/2022|EPI_ISL_15287366|2022-08-17
hCoV-19/Spain/VC-FISABIO-115103/2022|EPI_ISL_16100273|2022-10-31
hCoV-19/Sweden/101510276/2021|EPI_ISL_2981010|2021-04-06
hCoV-19/Sweden/20-07908/2020|EPI_ISL_452238|2020-04-27
hCoV-19/Sweden/21-20712/2020|EPI_ISL_2408182|2020-05-23
hCoV-19/Sweden/2898195413/2021|EPI_ISL_3157299|2021-05-06
hCoV-19/Sweden/4021089758VN/2022|EPI_ISL_16046268|2022-11-02
hCoV-19/Sweden/4021097974VN/2022|EPI_ISL_16046585|2022-10-31
hCoV-19/Sweden/DA10202700/2022|EPI_ISL_15423606|2022-09-28
hCoV-19/Switzerland/AG-UZH-IMV-3ba5db21/2022|EPI_ISL_9881442|2022-02-04
hCoV-19/Switzerland/GE-DIA-011612938801/2022|EPI_ISL_16013890|2022-11-07
hCoV-19/Switzerland/GE-DIA-011614236801/2022|EPI_ISL_16013907|2022-11-18
hCoV-19/Switzerland/JU-Risch-1012R14094/2022|EPI_ISL_15434786|2022-10-12
hCoV-19/Switzerland/JU-Risch-1125R14976/2022|EPI_ISL_16027486|2022-11-25
hCoV-19/Switzerland/LU-Risch-1128R15115/2022|EPI_ISL_16114630|2022-11-28
hCoV-19/Switzerland/un-UHG-H3007/2020|EPI_ISL_14688199|2020-03-17
hCoV-19/Switzerland/VD-CHUV-GEN11140/2022|EPI_ISL_11528051|2022-03-16
hCoV-19/Switzerland/VD-CHUV-GEN14274/2022|EPI_ISL_16046787|2022-11-16
hCoV-19/Switzerland/VS-ETHZ-38003306/2022|EPI_ISL_14755216|2022-08-12
hCoV-19/Switzerland/ZH-ETHZ-481687_SR0098_F2/2020|EPI_ISL_14210657|2020-10-26
hCoV-19/Switzerland/ZH-UZH-IMV-3ba5d5ff/2022|EPI_ISL_9882019|2022-02-01
hCoV-19/Switzerland/ZH-UZH-IMV-3ba5d7cd/2022|EPI_ISL_9880861|2022-02-02
hCoV-19/Switzerland/ZH-UZH-IMV-3ba5d7db/2022|EPI_ISL_9880948|2022-02-02
hCoV-19/Switzerland/ZH-UZH-IMV-3ba5d9f6/2022|EPI_ISL_9881823|2022-02-03
hCoV-19/Switzerland/ZH-UZH-IMV-46211155/2022|EPI_ISL_9881429|2022-02-01
hCoV-19/Switzerland/ZH-UZH-IMV-4634d4d5/2022|EPI_ISL_11013238|2022-03-02
hCoV-19/Thailand/CONI-3818/2022|EPI_ISL_16003411|2022-09-27
hCoV-19/Thailand/CU-A211078-NT/2021|EPI_ISL_17623599|2021-12-20
hCoV-19/Thailand/MTM_01_224/2022|EPI_ISL_14669064|2022-05-02

hCoV-19/Thailand/MTM_01_261/2022|EPI_ISL_14669089|2022-05-28

hCoV-19/Thailand/NIC_BKK_FRS810/2022|EPI_ISL_11449570|2022-03-01

hCoV-19/Thailand/NIC_BKK_FRS813/2022|EPI_ISL_11449571|2022-03-01

hCoV-19/Thailand/NIC_UTT_L22-28250/2022|EPI_ISL_11251424|2022-02-01

hCoV-19/Thailand/TUC-L22-53780/2022|EPI_ISL_15363585|2022-08-13

hCoV-19/Thailand/TUC-L22-61384/2022|EPI_ISL_16030147|2022-10-26

hCoV-19/Thailand/TUC-L22-61455/2022|EPI_ISL_16030214|2022-11-08

hCoV-19/Thailand/TUC-L22-61456/2022|EPI_ISL_16030215|2022-11-08

hCoV-19/Thailand/TUC-L22-61464/2022|EPI_ISL_16030222|2022-11-09

hCoV-19/Thailand/TUC-L22-61485/2022|EPI_ISL_16030242|2022-11-01

hCoV-19/Thailand/TUC-L22-61514/2022|EPI_ISL_16030268|2022-11-02

hCoV-19/Tunisia/S-1732/2021|EPI_ISL_16398494|2021-12-16

hCoV-19/Tunisia/Sfax/S-0682/2021|EPI_ISL_16944335|2021-03-14

hCoV-19/Turkey/HSGM-G2105/2022|EPI_ISL_10858380|2022-02-17

hCoV-19/Turkey/HSGM-GS9394/2022|EPI_ISL_15724607|2022-10-28

hCoV-19/Uganda/UG020/2020|EPI_ISL_451202|2020-05-01

hCoV-19/United_Arab_Emirates/BTC-5878/2021|EPI_ISL_16802320|2021-06-17

hCoV-19/United_Arab_Emirates/BTC-6364/2021|EPI_ISL_16802546|2021-07-08

hCoV-19/United_Arab_Emirates/BTC-6738/2021|EPI_ISL_16801359|2021-07-22

hCoV-19/United_Arab_Emirates/BTC-7678/2021|EPI_ISL_16804925|2021-08-26

hCoV-19/United_Arab_Emirates/BTC-7872/2021|EPI_ISL_16815587|2021-09-02

hCoV-19/United_Arab_Emirates/BTC-9243/2021|EPI_ISL_16814511|2021-10-21

hCoV-19/United_Arab_Emirates/BTC-9733/2021|EPI_ISL_16800858|2021-11-29

hCoV-19/United_Arab_Emirates/BTC-9824/2021|EPI_ISL_16800989|2021-12-07

hCoV-19/USA/AK-PHL22852/2020|EPI_ISL_15796362|2020-12-16

hCoV-19/USA/AL-UAB-GX222/2021|EPI_ISL_2993509|2021-04-23

hCoV-19/USA/AR-B011499/2021|EPI_ISL_17133311|2021-10-06

hCoV-19/USA/AR-B012097/2021|EPI_ISL_17133665|2021-11-15

hCoV-19/USA/AR-B06938/2021|EPI_ISL_17136453|2021-08-05

hCoV-19/USA/AR-V01578/2021|EPI_ISL_17141954|2021-08-24

hCoV-19/USA/AZ-ASPHL-1570/2020|EPI_ISL_2537014|2020-06-29

hCoV-19/USA/AZ-ASU90230/2022|EPI_ISL_15121085|2022-08-29

hCoV-19/USA/AZ-ASU92301/2020|EPI_ISL_15292583|2020-06-19

hCoV-19/USA/AZ-CDC-LC0711133/2022|EPI_ISL_13525659|2022-06-09

hCoV-19/USA/CA-ALSR-1090-SAN/2020|EPI_ISL_483517|2020-06-15

hCoV-19/USA/CA-CCPHL-3969/2022|EPI_ISL_15967751|2022-10-26

hCoV-19/USA/CA-CDC-LC0607544/2022|EPI_ISL_12656522|2022-05-01

hCoV-19/USA/CA-CDC-LC0609117/2022|EPI_ISL_12656461|2022-05-01

hCoV-19/USA/CA-CDC-LC0688331/2022|EPI_ISL_13307851|2022-05-25

hCoV-19/USA/CA-CDC-LC0691440/2022|EPI_ISL_13545091|2022-05-27

hCoV-19/USA/CA-CDC-LC0693009/2022|EPI_ISL_13544983|2022-05-27

hCoV-19/USA/CA-CDC-LC0710741/2022|EPI_ISL_13525583|2022-06-08

hCoV-19/USA/CA-CDC-LC0779388/2022|EPI_ISL_14055621|2022-07-13

hCoV-19/USA/CA-CDC-LC0785621/2022|EPI_ISL_14106350|2022-07-15
hCoV-19/USA/CA-CDC-LC0820634/2022|EPI_ISL_14501649|2022-08-02
hCoV-19/USA/CA-CDC-LC0822772/2022|EPI_ISL_14501058|2022-08-02
hCoV-19/USA/CA-CDC-LC0848853/2022|EPI_ISL_14737202|2022-08-13
hCoV-19/USA/CA-CDC-LC0896992/2022|EPI_ISL_15399907|2022-09-24
hCoV-19/USA/CA-CDC-LC0925522/2022|EPI_ISL_15744748|2022-10-31
hCoV-19/USA/CA-CDC-LC0949538/2022|EPI_ISL_16130457|2022-11-26
hCoV-19/USA/CA-CDPH-3000298376/2022|EPI_ISL_12921248|2022-01-03
hCoV-19/USA/CA-CDPH-3000298447/2022|EPI_ISL_12921266|2022-01-04
hCoV-19/USA/CA-CDPH-3000298472/2022|EPI_ISL_12921066|2022-01-04
hCoV-19/USA/CA-CDPH-3000328495/2022|EPI_ISL_13816711|2022-02-02
hCoV-19/USA/CA-CDPH-3000328541/2022|EPI_ISL_13816716|2022-02-02
hCoV-19/USA/CA-CDPH-3000328591/2022|EPI_ISL_13816585|2022-02-02
hCoV-19/USA/CA-CDPH-7000011243/2022|EPI_ISL_13036725|2022-02-05
hCoV-19/USA/CA-CDPH-7000014231/2022|EPI_ISL_14609922|2022-06-08
hCoV-19/USA/CA-Curative-432469/2022|EPI_ISL_14598016|2022-08-14
hCoV-19/USA/CA-CZB-14516/2020|EPI_ISL_739561|2020-11-11
hCoV-19/USA/CA-SEARCH-121852/2022|EPI_ISL_13527487|2022-05-02
hCoV-19/USA/CA-Stanford-83_S13/2022|EPI_ISL_13289738|2022-05-26
hCoV-19/USA/CA-USC-HW8248/2020|EPI_ISL_17067913|2020-12-30
hCoV-19/USA/CA-USC-VI9997/2020|EPI_ISL_17067787|2020-12-22
hCoV-19/USA/CO-CDC-LC0711096/2022|EPI_ISL_13525687|2022-06-09
hCoV-19/USA/CO-CDPHE-2101131135/2021|EPI_ISL_3159886|2021-05-19
hCoV-19/USA/CO-CDPHE-2101239058/2021|EPI_ISL_3089257|2021-05-31
hCoV-19/USA/CO-CDPHE-2103612135/2022|EPI_ISL_15101959|2022-08-16
hCoV-19/USA/CT-CDC-LC0675563/2022|EPI_ISL_13219102|2022-05-25
hCoV-19/USA/CT-CDC-LC0701403/2022|EPI_ISL_13555901|2022-06-10
hCoV-19/USA/CT-CDC-LC0885603/2022|EPI_ISL_15159124|2022-09-13
hCoV-19/USA/CT-DPH-1087199001/2022|EPI_ISL_13155795|2022-05-25
hCoV-19/USA/CT-Yale-18953/2022|EPI_ISL_12296746|2022-04-12
hCoV-19/USA/DC-CDC-LC0577296/2022|EPI_ISL_12304073|2022-04-12
hCoV-19/USA/DC-CDC-LC0669006/2022|EPI_ISL_13162996|2022-05-25
hCoV-19/USA/DC-CDC-LC0697936/2022|EPI_ISL_13553603|2022-06-08
hCoV-19/USA/FL-BPHL-21550/2021|EPI_ISL_17369402|2021-08-04
hCoV-19/USA/FL-BPHL-3176/2020|EPI_ISL_17186481|2020-06-27
hCoV-19/USA/FL-BPHL-3256/2020|EPI_ISL_17608189|2020-07-07
hCoV-19/USA/FL-CDC-LC0676919/2022|EPI_ISL_13220019|2022-05-27
hCoV-19/USA/FL-CDC-LC0677137/2022|EPI_ISL_13219246|2022-05-25
hCoV-19/USA/FL-CDC-LC0702993/2022|EPI_ISL_13555833|2022-06-10
hCoV-19/USA/FL-CDC-LC0711680/2022|EPI_ISL_13540892|2022-06-10
hCoV-19/USA/FL-CDC-LC0715591/2022|EPI_ISL_13541324|2022-06-12
hCoV-19/USA/FL-CDC-LC0940555/2022|EPI_ISL_16021117|2022-11-22
hCoV-19/USA/FL-CDC-LC0941387/2022|EPI_ISL_16021506|2022-11-23

hCoV-19/USA/GA-CDC-LC0611571/2022|EPI_ISL_12757728|2022-05-02
hCoV-19/USA/GA-CDC-LC0702874/2022|EPI_ISL_13554334|2022-06-08
hCoV-19/USA/GA-EHC-4671Q/2022|EPI_ISL_13864569|2022-05-28
hCoV-19/USA/GA-EHC-4778T/2022|EPI_ISL_13813492|2022-06-09
hCoV-19/USA/GA-EHC-5508V/2022|EPI_ISL_14982237|2022-07-15
hCoV-19/USA/GA-EHC-6081K/2022|EPI_ISL_15166731|2022-08-09
hCoV-19/USA/HI-H2210910/2022|EPI_ISL_12932599|2022-05-01
hCoV-19/USA/HI-H2214084/2022|EPI_ISL_14285146|2022-07-26
hCoV-19/USA/HI-H2214356/2022|EPI_ISL_14442336|2022-07-18
hCoV-19/USA/HI-H228781/2022|EPI_ISL_10952364|2022-03-02
hCoV-19/USA/IA-SHL-946506/2020|EPI_ISL_15026509|2020-03-25
hCoV-19/USA/IA-SHL-948633/2020|EPI_ISL_15026608|2020-03-28
hCoV-19/USA/ID-CDC-LC0620547/2022|EPI_ISL_12769992|2022-05-03
hCoV-19/USA/ID-IVREF-811188/2020|EPI_ISL_12037034|2020-07-09
hCoV-19/USA/IL-606014_S10-MN908947-3/2022|EPI_ISL_13435194|2022-05-27
hCoV-19/USA/IL-606133_S29-MN908947-3/2022|EPI_ISL_13435263|2022-05-26
hCoV-19/USA/IL-616041/2022|EPI_ISL_13524138|2022-06-09
hCoV-19/USA/IL-C22WGS4851/2022|EPI_ISL_14279360|2022-07-16
hCoV-19/USA/IL-C22WGS5309/2022|EPI_ISL_14258281|2022-07-18
hCoV-19/USA/IL-CDC-LC0608976/2022|EPI_ISL_12656455|2022-05-01
hCoV-19/USA/IL-CDC-LC0618102/2022|EPI_ISL_12757116|2022-05-02
hCoV-19/USA/IL-RIPHL_11595_Y/2020|EPI_ISL_16102151|2020-11-04
hCoV-19/USA/IL-RUSH_MTculture_d31/2020|EPI_ISL_14900780|2020-12-19
hCoV-19/USA/IL-RUSH-00649/2020|EPI_ISL_14380093|2020-03-18
hCoV-19/USA/KY-GD_03487/2021|EPI_ISL_15516235|2021-10-11
hCoV-19/USA/KY-GD_03887/2021|EPI_ISL_15526918|2021-10-20
hCoV-19/USA/KY-GD_04693/2022|EPI_ISL_16002925|2022-08-15
hCoV-19/USA/LA-BIE-CAT00605/2022|EPI_ISL_15370783|2022-08-31
hCoV-19/USA/LA-BIE-LSUH003456/2022|EPI_ISL_14372792|2022-07-26
hCoV-19/USA/LA-OD-D-0222041857/2022|EPI_ISL_14578603|2022-08-10
hCoV-19/USA/MA-NEIDL-01626/2021|EPI_ISL_15466688|2021-01-26
hCoV-19/USA/MA-NEIDL-04777/2022|EPI_ISL_11250855|2022-02-17
hCoV-19/USA/MA-NEIDL-05314/2022|EPI_ISL_11797589|2022-03-16
hCoV-19/USA/MA-NEIDL-06617/2022|EPI_ISL_13135909|2022-05-02
hCoV-19/USA/MA-NEIDL-06650/2022|EPI_ISL_13135942|2022-05-03
hCoV-19/USA/MA-NEIDL-06657/2022|EPI_ISL_13135949|2022-05-02
hCoV-19/USA/MD-CDC-LC0670155/2022|EPI_ISL_13163145|2022-05-25
hCoV-19/USA/MD-CDC-LC0677377/2022|EPI_ISL_13260053|2022-05-25
hCoV-19/USA/MD-HP07031-PIDMOCQTIE/2020|EPI_ISL_2885116|2020-05-04
hCoV-19/USA/MD-HP29350-PIDENOGWDM/2020|EPI_ISL_11963222|2020-03-18
hCoV-19/USA/MD-HP42001-PIDUIXNAQU/2020|EPI_ISL_16431309|2020-12-03
hCoV-19/USA/ME-HETL-J15529/2022|EPI_ISL_13350478|2022-05-01
hCoV-19/USA/ME-HETL-J18244/2020|EPI_ISL_17617484|2020-10-14

hCoV-19/USA/MI-UM-10048164563/2022|EPI_ISL_15815497|2022-10-31
hCoV-19/USA/MI-UM-L359542418/2022|EPI_ISL_16046867|2022-11-23
hCoV-19/USA/MI-UM-L359626278/2022|EPI_ISL_16046906|2022-11-28
hCoV-19/USA/MN-CDC-LC0707876/2022|EPI_ISL_13453140|2022-06-11
hCoV-19/USA/MN-Mayo-14308/2020|EPI_ISL_13598354|2020-09-16
hCoV-19/USA/MN-Mayo21154/2021|EPI_ISL_13684631|2021-06-18
hCoV-19/USA/MN-Mayo21702/2020|EPI_ISL_13687004|2020-04-11
hCoV-19/USA/MN-Mayo2832/2020|EPI_ISL_13680382|2020-07-30
hCoV-19/USA/MN-Mayo324/2020|EPI_ISL_13681737|2020-10-01
hCoV-19/USA/MN-Mayo4008/2020|EPI_ISL_13684313|2020-06-08
hCoV-19/USA/MN-Mayo4934/2020|EPI_ISL_13687956|2020-08-19
hCoV-19/USA/MN-Mayo6960/2020|EPI_ISL_13679765|2020-08-25
hCoV-19/USA/MN-Mayo7078/2020|EPI_ISL_13680147|2020-06-10
hCoV-19/USA/MN-Mayo7339/2020|EPI_ISL_13681061|2020-08-04
hCoV-19/USA/MN-Mayo8664/2021|EPI_ISL_13685477|2021-02-14
hCoV-19/USA/MN-MDH-1720/2020|EPI_ISL_576229|2020-09-16
hCoV-19/USA/MS-MSPHL-1726/2022|EPI_ISL_16014692|2022-11-29
hCoV-19/USA/MT-Chang-62119/2020|EPI_ISL_9275689|2020-10-08
hCoV-19/USA/MT-Walk-1950/2020|EPI_ISL_14099127|2020-10-26
hCoV-19/USA/MT-WL-2262/2020|EPI_ISL_12059771|2020-10-13
hCoV-19/USA/NC-CDC-LC0670777/2022|EPI_ISL_13163248|2022-05-26
hCoV-19/USA/NC-CDC-LC0670853/2022|EPI_ISL_13163280|2022-05-26
hCoV-19/USA/NC-CDC-LC0680068/2022|EPI_ISL_13308739|2022-05-29
hCoV-19/USA/NC-CDC-LC0699296/2022|EPI_ISL_13554977|2022-06-09
hCoV-19/USA/NC-CDC-LC0700847/2022|EPI_ISL_13555410|2022-06-10
hCoV-19/USA/NC-CORVASEQ-1085-6934/2022|EPI_ISL_14946046|2022-09-04
hCoV-19/USA/NC-CORVASEQ-CLT-008589/2022|EPI_ISL_13289494|2022-05-27
hCoV-19/USA/NC-CORVASEQ-CLT-008624/2022|EPI_ISL_13289527|2022-05-27
hCoV-19/USA/NC-CORVASEQ-CLT-008661/2022|EPI_ISL_13289561|2022-05-27
hCoV-19/USA/NC-CORVASEQ-CLT-008666/2022|EPI_ISL_13289566|2022-05-29
hCoV-19/USA/NC-CORVASEQ-CLT-008670/2022|EPI_ISL_13289570|2022-05-29
hCoV-19/USA/NC-CORVASEQ-R221093/2022|EPI_ISL_13227190|2022-05-26
hCoV-19/USA/NC-CORVASEQ-R221542/2022|EPI_ISL_14954699|2022-07-23
hCoV-19/USA/NC-ECU-CORVASEQ-111443327/2022|EPI_ISL_8509416|2022-01-02
hCoV-19/USA/NC-ECU-CORVASEQ-111454524/2022|EPI_ISL_8509383|2022-01-03
hCoV-19/USA/NC-ECU-CORVASEQ-111976453/2022|EPI_ISL_10206288|2022-02-02
hCoV-19/USA/NC-MCPH-CLT-008297/2022|EPI_ISL_13166608|2022-05-26
hCoV-19/USA/NC-MCPH-CLT-008513/2022|EPI_ISL_13289419|2022-05-27
hCoV-19/USA/NC-MCPH-CLT-008565/2022|EPI_ISL_13289470|2022-05-29
hCoV-19/USA/NC-MCPH-CLT-012335/2022|EPI_ISL_15258124|2022-09-22
hCoV-19/USA/NC-MCPH-CLT-012344/2022|EPI_ISL_15258133|2022-09-22
hCoV-19/USA/NC-MCPH-CLT-012594/2022|EPI_ISL_15305988|2022-09-29
hCoV-19/USA/NC-NCSU-111121R22C11/2021|EPI_ISL_16355729|2021-11-11

hCoV-19/USA/ND-NDDH-21317/2021|EPI_ISL_16066875|2021-11-18
hCoV-19/USA/ND-NDDH-23460/2021|EPI_ISL_17603035|2021-11-15
hCoV-19/USA/ND-NDDH-23522/2021|EPI_ISL_17603055|2021-12-29
hCoV-19/USA/ND-USAFSAM-S19351/2022|EPI_ISL_14726769|2022-08-08
hCoV-19/USA/NE-NCOV20-13872/2020|EPI_ISL_6124002|2020-05-11
hCoV-19/USA/NJ-CDC-LC0565095/2022|EPI_ISL_11530505|2022-03-16
hCoV-19/USA/NJ-CDC-LC0614037/2022|EPI_ISL_12758818|2022-05-03
hCoV-19/USA/NJ-CDC-LC0620145/2022|EPI_ISL_12758627|2022-05-03
hCoV-19/USA/NJ-CDC-LC0671995/2022|EPI_ISL_13220097|2022-05-27
hCoV-19/USA/NJ-CDC-LC0674968/2022|EPI_ISL_13219110|2022-05-25
hCoV-19/USA/NJ-CDC-LC0675261/2022|EPI_ISL_13220040|2022-05-27
hCoV-19/USA/NJ-CDC-LC0677231/2022|EPI_ISL_13260122|2022-05-26
hCoV-19/USA/NJ-CDC-LC0677550/2022|EPI_ISL_13260270|2022-05-28
hCoV-19/USA/NJ-CDC-LC0698684/2022|EPI_ISL_13553875|2022-06-08
hCoV-19/USA/NJ-CDC-LC0700202/2022|EPI_ISL_13554096|2022-06-08
hCoV-19/USA/NJ-CDC-LC0700311/2022|EPI_ISL_13554108|2022-06-08
hCoV-19/USA/NJ-CDC-LC0700469/2022|EPI_ISL_13554113|2022-06-08
hCoV-19/USA/NJ-CDC-LC0788766/2022|EPI_ISL_14184382|2022-07-19
hCoV-19/USA/NJ-CDC-LC0940825/2022|EPI_ISL_16020953|2022-11-20
hCoV-19/USA/NJ-MSHSPSP-PV65878/2022|EPI_ISL_13350710|2022-05-29
hCoV-19/USA/NM-NCGR-182041_IvcfC/2020|EPI_ISL_12698033|2020-05-08
hCoV-19/USA/NM-UNM-ED_4017/2022|EPI_ISL_16069661|2022-11-14
hCoV-19/USA/NM-UNM-ED_4063/2022|EPI_ISL_16069685|2022-11-16
hCoV-19/USA/NM-UNM-ED_4094/2022|EPI_ISL_16069704|2022-11-22
hCoV-19/USA/NM-UNM-ED_4102/2022|EPI_ISL_16069710|2022-11-27
hCoV-19/USA/NM-UNM-TRL_0522_007/2022|EPI_ISL_15969729|2022-04-24
hCoV-19/USA/NM-UNM-TRL_0522_014/2022|EPI_ISL_15969732|2022-04-26
hCoV-19/USA/NM-UNM-TRL_0622_217/2022|EPI_ISL_15455816|2022-05-25
hCoV-19/USA/NM-UNM-TRL_0622_240/2022|EPI_ISL_15455802|2022-05-28
hCoV-19/USA/NM-UNM-TRL_0822_151/2022|EPI_ISL_15200160|2022-08-03
hCoV-19/USA/NV-ASC-210989058/2022|EPI_ISL_16069783|2022-11-12
hCoV-19/USA/NY-ASC-210989037/2022|EPI_ISL_16069765|2022-11-12
hCoV-19/USA/NY-CDC-LC0565134/2022|EPI_ISL_11530471|2022-03-16
hCoV-19/USA/NY-CDC-LC0609766/2022|EPI_ISL_12757812|2022-05-02
hCoV-19/USA/NY-CDC-LC0700426/2022|EPI_ISL_13554148|2022-06-08
hCoV-19/USA/NY-MSHSPSP-PV52161/2022|EPI_ISL_12567394|2022-04-13
hCoV-19/USA/NY-MSHSPSP-PV57691/2022|EPI_ISL_12422517|2022-04-12
hCoV-19/USA/NY-MSHSPSP-PV57700/2022|EPI_ISL_12422520|2022-04-12
hCoV-19/USA/NY-MSHSPSP-PV57707/2022|EPI_ISL_12422526|2022-04-12
hCoV-19/USA/NY-MSHSPSP-PV57711/2022|EPI_ISL_12422527|2022-04-13
hCoV-19/USA/NY-MSHSPSP-PV59695/2022|EPI_ISL_13350624|2022-05-29
hCoV-19/USA/NY-MSK-1905/2020|EPI_ISL_14556360|2020-03-24
hCoV-19/USA/NY-MSK-2597/2020|EPI_ISL_15047409|2020-03-20

hCoV-19/USA/NY-NYULH6198/2022|EPI_ISL_12061382|2022-03-17
hCoV-19/USA/NY-NYULH6539/2022|EPI_ISL_12329835|2022-04-13
hCoV-19/USA/NY-NYUMC393/2020|EPI_ISL_430420|2020-04-13
hCoV-19/USA/NY-PRL-0506_00D11/2022|EPI_ISL_12707231|2022-05-03
hCoV-19/USA/NY-PRL-0506_01A11/2022|EPI_ISL_12707397|2022-05-03
hCoV-19/USA/NY-PRL-2021_0430_00C09/2021|EPI_ISL_2965125|2021-04-29
hCoV-19/USA/NY-PRL-2022_0320_01C13/2022|EPI_ISL_11472382|2022-03-16
hCoV-19/USA/NY-PRL-2022_0320_01C15/2022|EPI_ISL_11472383|2022-03-17
hCoV-19/USA/NY-PRL-2022_0320_01G23/2022|EPI_ISL_11472457|2022-03-17
hCoV-19/USA/NY-PRL-2022_0413_00A15/2022|EPI_ISL_12255392|2022-04-12
hCoV-19/USA/NY-PRL-2022_0413_00C14/2022|EPI_ISL_12255428|2022-04-12
hCoV-19/USA/NY-PRL-2022_0413_00D21/2022|EPI_ISL_12255455|2022-04-12
hCoV-19/USA/NY-PRL-2022_0413_01G20/2022|EPI_ISL_12255700|2022-04-12
hCoV-19/USA/NY-PRL-2022_0506_01F17/2022|EPI_ISL_12707559|2022-05-03
hCoV-19/USA/NY-PRL-2022_0506_02E04/2022|EPI_ISL_12707723|2022-05-03
hCoV-19/USA/NY-PRL-2022_0506_02H23/2022|EPI_ISL_12707789|2022-05-02
hCoV-19/USA/NY-PRL-2022_0506_02K12/2022|EPI_ISL_12707823|2022-05-03
hCoV-19/USA/NY-PRL-2022_0506_03D07/2022|EPI_ISL_12707874|2022-05-03
hCoV-19/USA/NY-PRL-220526_02D11/2022|EPI_ISL_13122602|2022-05-25
hCoV-19/USA/NY-PRL-220527_00K01/2022|EPI_ISL_13364260|2022-05-25
hCoV-19/USA/NY-PRL-220527_01E24/2022|EPI_ISL_13255274|2022-05-25
hCoV-19/USA/NY-PRL-220527_01J01/2022|EPI_ISL_13255343|2022-05-25
hCoV-19/USA/NY-PRL-220527_02D07/2022|EPI_ISL_13364347|2022-05-26
hCoV-19/USA/NY-PRL-220527_03D10/2022|EPI_ISL_13364503|2022-05-26
hCoV-19/USA/NY-PRL-220527_03L16/2022|EPI_ISL_13364545|2022-05-26
hCoV-19/USA/NY-PRL-220530_00C20/2022|EPI_ISL_13364610|2022-05-28
hCoV-19/USA/NY-PRL-220530_00D11/2022|EPI_ISL_13364622|2022-05-27
hCoV-19/USA/NY-PRL-220530_00E01/2022|EPI_ISL_13364635|2022-05-28
hCoV-19/USA/NY-PRL-220530_00F23/2022|EPI_ISL_13364670|2022-05-29
hCoV-19/USA/NY-PRL-220530_00G12/2022|EPI_ISL_13364680|2022-05-29
hCoV-19/USA/NY-PRL-220530_00G23/2022|EPI_ISL_13364689|2022-05-29
hCoV-19/USA/NY-PRL-220530_00K05/2022|EPI_ISL_13364740|2022-05-27
hCoV-19/USA/NY-PRL-220530_01A05/2022|EPI_ISL_13364770|2022-05-29
hCoV-19/USA/NY-PRL-220530_01E23/2022|EPI_ISL_13364867|2022-05-27
hCoV-19/USA/NY-PRL-220530_01J07/2022|EPI_ISL_13364939|2022-05-26
hCoV-19/USA/NY-PRL-220530_01J11/2022|EPI_ISL_13364942|2022-05-28
hCoV-19/USA/NY-PRL-220530_01L12/2022|EPI_ISL_13364968|2022-05-29
hCoV-19/USA/NY-PRL-220530_02C12/2022|EPI_ISL_13255410|2022-05-28
hCoV-19/USA/NY-PRL-220530_02C13/2022|EPI_ISL_13255411|2022-05-27
hCoV-19/USA/NY-PRL-220530_03B01/2022|EPI_ISL_13255611|2022-05-29
hCoV-19/USA/NY-PRL-220530_03B15/2022|EPI_ISL_13255622|2022-05-29
hCoV-19/USA/NY-PRL-220530_03E13/2022|EPI_ISL_13255682|2022-05-29
hCoV-19/USA/NY-PRL-220530_03J06/2022|EPI_ISL_13255760|2022-05-29

hCoV-19/USA/NY-PRL-220530_04A12/2022|EPI_ISL_13255810|2022-05-28
hCoV-19/USA/NY-PRL-220530_04D17/2022|EPI_ISL_13255878|2022-05-27
hCoV-19/USA/NY-PRL-220610_00E16/2022|EPI_ISL_13365609|2022-06-08
hCoV-19/USA/NY-PRL-220610_00E23/2022|EPI_ISL_13365614|2022-06-08
hCoV-19/USA/NY-PRL-220610_00H15/2022|EPI_ISL_13365656|2022-06-08
hCoV-19/USA/NY-PRL-220613_01E09/2022|EPI_ISL_13366373|2022-06-12
hCoV-19/USA/NY-PRL-220613_01E16/2022|EPI_ISL_13366378|2022-06-12
hCoV-19/USA/NY-PRL-220613_01F23/2022|EPI_ISL_13366400|2022-06-10
hCoV-19/USA/NY-PRL-220613_01G08/2022|EPI_ISL_13366407|2022-06-11
hCoV-19/USA/NY-PRL-220613_01I04/2022|EPI_ISL_13366443|2022-06-10
hCoV-19/USA/NY-PRL-220613_02K24/2022|EPI_ISL_13366700|2022-06-09
hCoV-19/USA/NY-PRL-220613_03N20/2022|EPI_ISL_13366785|2022-06-10
hCoV-19/USA/NY-PRL-220719_03M12/2022|EPI_ISL_14030152|2022-07-15
hCoV-19/USA/NY-PRL-220817_01I18/2022|EPI_ISL_14684749|2022-08-15
hCoV-19/USA/NY-PRL-220824_00E03/2022|EPI_ISL_14807909|2022-08-20
hCoV-19/USA/NY-PRL-220831_01H04/2022|EPI_ISL_14914249|2022-08-29
hCoV-19/USA/NY-PRL-221102_00D04/2022|EPI_ISL_15749411|2022-10-31
hCoV-19/USA/NY-PRL-221128_03B16/2022|EPI_ISL_16001597|2022-11-22
hCoV-19/USA/NY-PRL-221128_03B24/2022|EPI_ISL_16001603|2022-11-22
hCoV-19/USA/NY-PRL-221128_03E18/2022|EPI_ISL_16001655|2022-11-22
hCoV-19/USA/NY-PRL-221128_03F03/2022|EPI_ISL_16001663|2022-11-23
hCoV-19/USA/NY-PRL-221128_03F20/2022|EPI_ISL_16001678|2022-11-22
hCoV-19/USA/NY-PRL-221128_03G06/2022|EPI_ISL_16001683|2022-11-23
hCoV-19/USA/NY-PRL-221128_03J10/2022|EPI_ISL_16001739|2022-11-22
hCoV-19/USA/NY-Wadsworth-22022777-01/2022|EPI_ISL_13436133|2022-05-03
hCoV-19/USA/NY-Wadsworth-22034348-01/2022|EPI_ISL_14440826|2022-07-13
hCoV-19/USA/NY-Wadsworth-22035201-01/2022|EPI_ISL_14987771|2022-07-13
hCoV-19/USA/NY-Wadsworth-22038901-01/2022|EPI_ISL_14987811|2022-08-03
hCoV-19/USA/NY-Wadsworth-22038907-01/2022|EPI_ISL_14987814|2022-08-03
hCoV-19/USA/NY-Wadsworth-22040870-01/2022|EPI_ISL_15023115|2022-08-19
hCoV-19/USA/NY-Wadsworth-277962-01/2020|EPI_ISL_754606|2020-11-22
hCoV-19/USA/OH-CDC-QDX37502564/2022|EPI_ISL_13290125|2022-05-27
hCoV-19/USA/OK-OKPHL0017612/2022|EPI_ISL_15556111|2022-05-26
hCoV-19/USA/OK-OKPHLCOV0002184/2021|EPI_ISL_15568914|2021-09-14
hCoV-19/USA/OR_UO_MAP005339/2022|EPI_ISL_15048996|2022-07-29
hCoV-19/USA/OR_UO_MW000850_S32_L001/2022|EPI_ISL_10499520|2022-02-04
hCoV-19/USA/OR_UO_MW001050_S86_L001/2022|EPI_ISL_12626181|2022-03-02
hCoV-19/USA/OR-OHSU-1565/2020|EPI_ISL_525979|2020-07-20
hCoV-19/USA/PA-CDC-2029000/2021|EPI_ISL_17533617|2021-02-20
hCoV-19/USA/PA-CDC-2851900/2021|EPI_ISL_17532718|2021-03-03
hCoV-19/USA/RI-RISHL-E01400/2021|EPI_ISL_17602548|2021-07-21
hCoV-19/USA/RI-RISHL-E01848/2021|EPI_ISL_17602614|2021-08-31
hCoV-19/USA/SC-004DED7BEEL/2021|EPI_ISL_17136675|2021-04-19

hCoV-19/USA/SC-0628/2021|EPI_ISL_17137515|2021-02-23
hCoV-19/USA/SC-2668/2021|EPI_ISL_17113918|2021-07-22
hCoV-19/USA/SC-3290_T28/2020|EPI_ISL_17141380|2020-09-27
hCoV-19/USA/SC-3854/2021|EPI_ISL_17114749|2021-08-06
hCoV-19/USA/SC-4015/2021|EPI_ISL_17114860|2021-09-01
hCoV-19/USA/SC-5684/2021|EPI_ISL_17115680|2021-10-21
hCoV-19/USA/SC-7198/2021|EPI_ISL_17138318|2021-12-22
hCoV-19/USA/SC-MD-0000009/2021|EPI_ISL_17116664|2021-09-26
hCoV-19/USA/SC-MUSC-07012/2022|EPI_ISL_12830594|2022-03-17
hCoV-19/USA/SC-REDDI-257F8AA87PB/2022|EPI_ISL_15892454|2022-10-17
hCoV-19/USA/SC-REDDI-7B539FFFDPU-2/2021|EPI_ISL_15636708|2021-07-30
hCoV-19/USA/SC-REDDI-FE77A2A2CMU/2022|EPI_ISL_15393700|2022-07-28
hCoV-19/USA/TN-6-88483-2/2020|EPI_ISL_15532849|2020-12-30
hCoV-19/USA/TN-CDC-2-3693660/2020|EPI_ISL_747062|2020-11-06
hCoV-19/USA/TN-SPHL-3352/2021|EPI_ISL_14931504|2021-01-07
hCoV-19/USA/TX-CDC-LC0717247/2022|EPI_ISL_13540536|2022-06-09
hCoV-19/USA/TX-CDC-LC0950153/2022|EPI_ISL_16130518|2022-11-28
hCoV-19/USA/TX-HHD-12214397994/2020|EPI_ISL_7505889|2020-07-04
hCoV-19/USA/TX-HMH-M-106862/2022|EPI_ISL_14028302|2022-07-14
hCoV-19/USA/TX-HMH-M-107460/2022|EPI_ISL_14179220|2022-07-15
hCoV-19/USA/TX-HMH-M-107526/2022|EPI_ISL_14179300|2022-07-17
hCoV-19/USA/TX-HMH-M-107808/2022|EPI_ISL_14179687|2022-07-18
hCoV-19/USA/TX-HMH-M-107924/2022|EPI_ISL_14179801|2022-07-19
hCoV-19/USA/TX-HMH-M-108101/2022|EPI_ISL_14179576|2022-07-19
hCoV-19/USA/TX-HMH-M-108874/2022|EPI_ISL_14180044|2022-07-22
hCoV-19/USA/TX-HMH-M-109745/2022|EPI_ISL_14313592|2022-07-27
hCoV-19/USA/TX-HMH-M-111697/2022|EPI_ISL_14552490|2022-08-09
hCoV-19/USA/TX-HMH-M-112206/2022|EPI_ISL_14608822|2022-08-12
hCoV-19/USA/TX-HMH-M-112545/2022|EPI_ISL_14608092|2022-08-15
hCoV-19/USA/TX-HMH-M-112733/2022|EPI_ISL_14608281|2022-08-16
hCoV-19/USA/TX-HMH-M-114329/2022|EPI_ISL_14954350|2022-08-29
hCoV-19/USA/TX-HMH-M-118976/2022|EPI_ISL_16113588|2022-11-28
hCoV-19/USA/TX-HMH-M-119074/2022|EPI_ISL_16113681|2022-11-28
hCoV-19/USA/TX-HMH-M-119089/2022|EPI_ISL_16113618|2022-11-28
hCoV-19/USA/TX-HMH-M-119102/2022|EPI_ISL_16113605|2022-11-28
hCoV-19/USA/TX-HMH-M-119119/2022|EPI_ISL_16113646|2022-11-29
hCoV-19/USA/TX-HMH-MCoV-116133/2022|EPI_ISL_15306165|2022-09-19
hCoV-19/USA/TX-HMH-MCoV-19448/2020|EPI_ISL_783846|2020-11-30
hCoV-19/USA/TX-HMH-MCoV-99413/2022|EPI_ISL_13269937|2022-05-26
hCoV-19/USA/TX-PHH-SPID1004433151/2022|EPI_ISL_16020567|2022-10-31
hCoV-19/USA/TX-PHH-SPID1004433178/2022|EPI_ISL_16020581|2022-10-29
hCoV-19/USA/TX-TAMGHRC-GHRC_MKM43/2022|EPI_ISL_10271784|2022-02-05
hCoV-19/USA/TX-TAMGHRC-L-73824-22R/2022|EPI_ISL_12086390|2022-02-01

hCoV-19/USA/TX-TAMGHRC-SHS_83277/2022|EPI_ISL_15754213|2022-09-28
hCoV-19/USA/TX-TAMGHRC-SHS-79746/2022|EPI_ISL_14865977|2022-07-18
hCoV-19/USA/TX-TAMGHRC-SHS-80752/2022|EPI_ISL_14866025|2022-08-22
hCoV-19/USA/TX-TAMGHRC-SHS-81395/2022|EPI_ISL_15195036|2022-08-30
hCoV-19/USA/UT-UPHL-08762/2020|EPI_ISL_525348|2020-08-14
hCoV-19/USA/UT-UPHL-220624595350/2022|EPI_ISL_13492924|2022-05-26
hCoV-19/USA/UT-UPHL-220818426376/2022|EPI_ISL_14582879|2022-07-26
hCoV-19/USA/UT-UPHL-221115472146/2022|EPI_ISL_15881847|2022-10-28
hCoV-19/USA/VA-CDC-LC0609980/2022|EPI_ISL_12756916|2022-05-01
hCoV-19/USA/VA-CDC-LC0698751/2022|EPI_ISL_13555069|2022-06-09
hCoV-19/USA/WA-CDC-4190831/2021|EPI_ISL_17580880|2021-04-23
hCoV-19/USA/WA-CDC-LC0617066/2022|EPI_ISL_12758205|2022-05-03
hCoV-19/USA/WA-CDC-LC0706400/2022|EPI_ISL_13558757|2022-06-10
hCoV-19/USA/WA-CDC-LC0706746/2022|EPI_ISL_13558795|2022-06-10
hCoV-19/USA/WA-CDC-UW22031649410/2022|EPI_ISL_11439121|2022-03-16
hCoV-19/USA/WA-CDC-UW22031708656/2022|EPI_ISL_11439087|2022-03-17
hCoV-19/USA/WA-CDC-UW22060801306/2022|EPI_ISL_13356347|2022-06-08
hCoV-19/USA/WA-CDC-UW22060879479/2022|EPI_ISL_13356370|2022-06-08
hCoV-19/USA/WA-CDC-UW22060902569/2022|EPI_ISL_13465741|2022-06-09
hCoV-19/USA/WA-FHCRC-1109/2020|EPI_ISL_17578385|2020-03-16
hCoV-19/USA/WA-FHCRC-1732/2020|EPI_ISL_17578147|2020-03-21
hCoV-19/USA/WA-FHCRC-3718/2020|EPI_ISL_17578377|2020-03-25
hCoV-19/USA/WA-FHCRC-4742/2020|EPI_ISL_17578005|2020-03-27
hCoV-19/USA/WA-FHCRC-9368/2020|EPI_ISL_17578689|2020-05-16
hCoV-19/USA/WA-PHL-029257/2020|EPI_ISL_15462513|2020-03-26
hCoV-19/USA/WA-PHL-029281/2020|EPI_ISL_15462536|2020-12-16
hCoV-19/USA/WA-S19935/2022|EPI_ISL_12710783|2022-05-03
hCoV-19/USA/WA-UW-13300/2020|EPI_ISL_501134|2020-07-03
hCoV-19/USA/WA-UW-2115/2020|EPI_ISL_17578770|2020-03-25
hCoV-19/USA/WA-UW-22061236236/2022|EPI_ISL_13466927|2022-06-12
hCoV-19/USA/WA-UW-22061250115/2022|EPI_ISL_13466965|2022-06-12
hCoV-19/USA/WA-UW-22071742558/2022|EPI_ISL_14250036|2022-07-17
hCoV-19/USA/WA-UW-22080494610/2022|EPI_ISL_14624702|2022-08-04
hCoV-19/USA/WA-UW-22082208298/2022|EPI_ISL_14992318|2022-08-22
hCoV-19/USA/WA-UW-22082795649/2022|EPI_ISL_15016366|2022-08-27
hCoV-19/USA/WA-UW122/2020|EPI_ISL_416660|2020-03-10
hCoV-19/USA/WA-UW170/2020|EPI_ISL_416708|2020-03-14
hCoV-19/USA/WA1-F6/2020|EPI_ISL_407215|2020-01-25
hCoV-19/USA/WI-CDC-4063380/2020|EPI_ISL_17577655|2020-12-29
hCoV-19/USA/WI-CDC-4063433/2021|EPI_ISL_17577622|2021-03-03
hCoV-19/USA/WI-CDC-LC0949412/2022|EPI_ISL_16130952|2022-11-29
hCoV-19/USA/WI-MHDL-100037/2022|EPI_ISL_15313938|2022-08-31
hCoV-19/USA/WI-MHDL-110106/2022|EPI_ISL_15747741|2022-10-28

hCoV-19/USA/WI-UW-1046/2020|EPI_ISL_516472|2020-07-29

hCoV-19/USA/WI-UW-258/2020|EPI_ISL_428732|2020-04-06

hCoV-19/USA/WI-UW-608/2020|EPI_ISL_484983|2020-06-23

hCoV-19/USA/WV-CDC-LC0617137/2022|EPI_ISL_12757290|2022-05-02

hCoV-19/USA/WV-WV091501/2021|EPI_ISL_17117022|2021-11-30

hCoV-19/USA/WV-WV127825/2021|EPI_ISL_17118349|2021-12-01

hCoV-19/USA/WY-UNM-21093406/2021|EPI_ISL_17616758|2021-09-14

hCoV-19/USA/WY-WYPHL-20074665/2020|EPI_ISL_14355194|2020-09-14

hCoV-19/Vietnam/HCMC_Alpha_02/2021|EPI_ISL_16828680|2021-03-29

hCoV-19/Vietnam/PIHCM-0001/2020|EPI_ISL_17454568|2020-01-20

hCoV-19/Wales/LSPA-3E5241B/2022|EPI_ISL_13377011|2022-06-09

hCoV-19/Wales/PHW2/2020|EPI_ISL_413556|2020-03-04

hCoV-19/Wales/PHWC-16A06D/2020|EPI_ISL_535193|2020-09-06

hCoV-19/Wales/PHWC-16B837/2020|EPI_ISL_573908|2020-09-15

hCoV-19/Wales/PHWC-29E16/2020|EPI_ISL_432181|2020-04-04

hCoV-19/Wales/PHWC-4AE686/2020|EPI_ISL_822345|2020-11-08

hCoV-19/Wuhan/0125-A168/2020|EPI_ISL_493159|2020-01-25

hCoV-19/Wuhan/0125-A192/2020|EPI_ISL_493166|2020-01-25

hCoV-19/Wuhan/HBCDC-HB-02/2020|EPI_ISL_412978|2020-01-17

hCoV-19/Zhejiang/ZPCDCP-02/2021|EPI_ISL_17261504|2021-07-18

Wuhan-Hu-1|2020-01-05

Supplementary Table 2. Meta data for household participants.

Participant	Gender	Age	Number of vaccine doses	Household ID	Case	RT-PCR result	Variant	Date first positive swab
1	Male	2	0	HH 1	Index	Positive	BA.2	29/03/2022
2	Female	4	0	HH 1	Secondary	Positive	BA.2	29/03/22
3	Female	31	3	HH 1	Secondary	Positive	BA.2	1/04/22
4	Male	35	2	HH 1		Negative		
5	Male	2	0	HH 2	Secondary	Positive	BA.2	21/03/22
6	Male	7	1	HH 2	Secondary	Positive	BA.2	21/03/22
7	Male	9	0	HH 2	Secondary	Positive	BA.2	21/03/22
8	Female	12	2	HH 2	Index	Positive	BA.2	21/03/2022
9	Female	14	2	HH 2	Secondary	Positive	BA.2	21/03/2022
10	Female	34	3	HH 2	Secondary	Positive	BA.2	21/03/22
11	Female	2	0	HH 3	Index	Positive	BA.5	15/07/22
12	Female	39	3	HH 3	Secondary	Positive	BA.5	18/07/22
13	Male	49	2	HH 3	Secondary	Positive	BA.5	18/07/22
14	Female	2	0	HH 4	Index	Positive	BA.2	11/04/22
15	Female	4	0	HH 4	Secondary	Positive	BA.2	14/04/22
16	Male	33	3	HH 4	Secondary	Positive	BA.2	14/04/22
17	Female	33	3	HH 4	Secondary	Positive	BA.2	14/04/22
18	Female	2	0	HH 5		Negative		
19	Female	3	0	HH 5		Negative		
20	Female	11	0	HH 5		Negative		
21	Female	14	2	HH 5		Negative		
22	Male	30	2	HH 5	Index	Positive	BA.2	18/05/22
23	Female	31	2	HH 5		Negative		
24	Female	1	0	HH 6	Secondary	Positive	BA.5	21/07/22
25	Female	6	0	HH 6	Secondary	Positive	BA.5	21/07/22
26	Female	35	3	HH 6	Secondary	Positive	BA.5	21/07/22
27	Male	46	2	HH 6	Index	Positive	BA.5	18/07/2022
28	Female	2	0	HH 7		Negative		
29	Male	4	0	HH 7	Secondary	Positive	BA.2	4/05/22
30	Male	34	3	HH 7	Index	Positive	BA.2	04/05/2022
31	Female	34	3	HH 7	Secondary	Positive	BA.2	04/05/2022
32	Female	1	0	HH 8	Secondary	Positive	BA.2	2/03/22
33	Female	5	1	HH 8	Secondary	Positive	BA.2	2/03/22
34	Male	7	1	HH 8	Index	Positive	BA.2	01/03/2022
35	Female	36	2	HH 8	Secondary	Positive	BA.2	5/03/22
36	Male	39	2	HH 8	Secondary	Positive	BA.2	02/03/2022
37	Female	1	0	HH 9	Secondary	Positive	BA.2	20/04/22
38	Female	3	0	HH 9	Index	Positive	BA.2	11/04/22
39	Female	31	3	HH 9	Secondary	Positive	BA.2	17/04/22
40	Male	37	3	HH 9	Secondary	Positive	BA.2	20/04/22
41	Female	1	0	HH 10		Negative		
42	Female	34	3	HH 10		Negative		
43	Male	35	3	HH 10	Index	Positive	BA.2	27/04/2022
44	Female	64	3	HH 10		Negative		
45	Female	1	0	HH 11	Secondary	Positive	BA.2	17/03/22
46	Male	3	0	HH 11	Secondary	Positive	BA.2	14/03/22
47	Female	5	0	HH 11	Secondary	Positive	BA.2	16/03/22

48	Female	6	0	HH 11	Secondary	Positive	BA.2	16/03/22
49	Female	31	2	HH 11	Secondary	Positive	BA.2	16/03/22
50	Male	33	2	HH 11	Index	Positive	BA.2	16/03/22
				HH 12[a]		Negative		
51	Female	1	0	HH 12[b]		Negative		
				HH 12[a]		Negative		
52	Female	34	3	HH 12[b]	Index	Positive	BA.2	14/06/2022
				HH 12[a]	Primari[a]	Positive	BA.2	14/03/2022
53	Male	34	3	HH 12[b]		Negative		
54	Female	1	0	HH 13	Secondary	Positive	BA.2	7/05/22
55	Female	5	2	HH 13	Secondary	Positive		10/05/2022
56	Female	7	2	HH 13	Secondary	Positive	BA.2	10/05/22
57	Female	36	3	HH 13	Secondary	Positive	BA.2	10/05/22
58	Male	38	3	HH 13	Index	Positive	BA.2	05/05/2022
59	Female	1	0	HH 14		Negative		
60	Female	28	3	HH 14	Index	Positive	BA.2	21/03/2022
61	Male	29	3	HH 14		Negative		
62	Female	14	2	HH 15	Index	Positive		14/03/2022
63	Female	34	3	HH 15		Negative		
64	Female	11	1	HH 16	Index	Positive	BA.2	08/03/2022
65	Female	13	2	HH 16	Secondary	Positive	BA.2	8/03/22
66	Female	39	3	HH 16	Secondary	Positive	BA.2	16/03/22
67	Male	42	3	HH 16	Secondary	Positive	BA.2	13/03/22
68	Female	67	3	HH 16	Secondary	Positive	BA.2	13/03/22
69	Female	0	0	HH 17	Secondary	Positive	BA.2	24/03/22
70	Female	4	0	HH 17	Secondary	Positive	BA.2	24/03/2022
71	Male	8	0	HH 17	Secondary	Positive	BA.2	24/03/22
72	Male	10	0	HH 17	Secondary	Positive	BA.2	24/03/22
73	Male	12	1	HH 17	Index	Positive	BA.2	21/03/2022
74	Male	35	2	HH 17	Secondary	Positive	BA.2	21/03/22
75	Female	35	2	HH 17	Secondary	Positive	BA.2	24/03/22
				HH 18[a]		Negative		
76	Female	12	2	HH 18[b]		Negative		
				HH 18[a]		Negative		
77	Female	12	2	HH 18[b]		Negative		
				HH 18[a]	Index[a]	Positive	BA.1	14/03/2022
78	Female	13	2	HH 18[b]		Negative		
				HH 18[a]	Secondary	Positive		14/03/2022
79	Female	45	3	HH 18[b]	Secondary	Positive	BA.4	26/07/2022
				HH 18[a]		Negative		
80	Male	54	3	HH 18[b]	Index[b]	Positive	BA.4	26/07/22
				HH 19[a]		Negative		
81	Male	12	2	HH 19[b]		Negative		
				HH 19[a]	Index[a]	Positive	BA.2	07/03/2022
82	Male	13	2	HH 19[b]		Negative		
				HH 19[a]	Secondary	Positive		18/03/2022
83	Female	16	2	HH 19[b]		Negative		
				HH 19[a]		Negative		
84	Female	71	3	HH 19[b]	Secondary	Positive	BA.2	18/05/22
85	Male	60	3	HH 19[a]		Negative		

				HH 19[b]	Index[b]	Positive	BA.2	19/05/2022
86	Female	15	0	HH 20	Index	Positive	BA.2	8/07/22
87	Male	19	2	HH 20	Secondary	Positive	BA.2	
88	Female	37	3	HH 20	Secondary	Positive	BA.2	14/07/22
89	Male	2	0	HH 21	Secondary	Positive	BA.5	5/09/22
90	Female	40	2	HH 21	Index	Positive	BA.5	16/08/2022
91	Male	51	2	HH 21	Secondary	Positive	BA.5	2/09/22
92	Female	1	0	HH 22	Index	Positive	BA.2	07/03/2022
93	Female	42	3	HH 22	Secondary	Positive	BA.2	12/03/22
94	Male	50	3	HH 22	Secondary	Positive		12/03/2022
95	Male	7	1	HH 23	Index	Positive	BA.2	09/03/2022
96	Female	13	2	HH 23		Negative		
97	Female	42	3	HH 23	Secondary	Positive		13/03/2022
98	Male	43	3	HH 23		Negative		
99	Other	64	3	HH 23		Negative		
	Female			HH 24[a]		Negative		
100	Female	13	2	HH 24[b]	Index[b]	Positive	BA.2	11/05/22
	Male			HH 24[a]	Index[a]	Positive	BA.1	23/02/2022
101	Male	16	2	HH 24[b]	Secondary	Positive		04/06/2022
	Female			HH 24[a]		Negative		
102	Female	48	3	HH 24[b]	Secondary	Positive	BA.2	11/05/22
	Male			HH 24[a]		Negative		
103	Male	54	3	HH 24[b]	Secondary	Positive	BA.2	17/05/22
104	Male	19	3	HH 24[b]	Secondary	Positive	BA.2	14/05/22
105	Female	11	1	HH 25	Index	Positive	BA.2	14/03/22
106	Female	15	2	HH 25	Secondary	Positive	BA.2	14/03/2022
107	Female	49	2	HH 25	Secondary	Positive	BA.2	14/03/22
108	Male	76	3	HH 25	Secondary	Positive	BA.2	14/03/22
109	Female	12	2	HH 26	Index	Positive	BA.2	14/03/2022
110	Male	16	2	HH 26		Negative		
111	Female	46	3	HH 26		Negative		
112	Male	46	3	HH 26		Negative		
113	Female	16	2	HH 27	Index	Positive	BA.2	10/03/2022
114	Female	44	3	HH 27		Negative		
115	Male	50	3	HH 27		Negative		
116	Male	16	2	HH 28	Secondary	Positive	BA.2	24/03/22
117	Female	50	3	HH 28	Index	Positive	BA.2	07/03/2022
118	Female	11	1	HH 29	Secondary	Positive	BA.2	11/03/22
119	Male	14	2	HH 29	Secondary	Positive	BA.2	11/03/22
120	Male	17	2	HH 29	Index	Positive		11/03/2022
121	Male	46	3	HH 29		Negative		
122	Female	46	3	HH 29		Negative		
123	Male	13	2	HH 30		Negative		
124	Female	15	2	HH 30	Index	Positive	BA.2	29/03/22
125	Male	18	2	HH 30		Negative		
126	Female	49	3	HH 30	Secondary	Positive	BA.2	29/03/2022
127	Male	50	3	HH 30	Secondary	Positive	BA.2	1/04/22
128	Male	16	2	HH 31	Index	Positive	BA.2	17/05/2022
129	Male	18	3	HH 31	Secondary	Positive	BA.2	20/05/22
130	Female	51	3	HH 31	Secondary	Positive	BA.2	23/05/22

131	Male	52	3	HH 31	Secondary	Positive	BA.2	29/05/22
132	Male	13	2	HH 32	Secondary	Positive	BA.2	18/05/22
133	Female	16	3	HH 32	Secondary	Positive	BA.2	21/05/22
134	Female	52	3	HH 32	Secondary	Positive	BA.2	21/05/22
135	Male	52	3	HH 32	Index	Positive	BA.2	19/05/2022
136	Male	1	0	HH 33	Index	Positive	BA.2	31/03/22
137	Male	35	3	HH 33		Negative		
138	Female	36	3	HH 33	Secondary	Positive	BA.2	31/03/2022
139	Male	13	2	HH 34	Index	Positive	BA.2	28/06/22
140	Female	46	2	HH 34	Secondary	Positive	BA.2	30/06/22
141	Female	6	0	HH 35	Index	Positive	BA.2	8/07/22
142	Female	8	0	HH 35	Secondary	Positive	BA.2	14/07/22
143	Female	45	3	HH 35	Secondary	Positive	BA.2	14/07/22
144	Male	48	3	HH 35	Secondary	Positive	BA.2	8/07/22
145	Female	70	2	HH 35	Index	Positive	BA.5	8/07/22
146	Male	7	0	HH 36	Secondary	Positive	BA.2	10/03/22
147	Female	42	2	HH 36	Index	Positive	BA.2	02/03/2022
148	Male	43	2	HH 36	Secondary	Positive	BA.2	4/03/22
149	Male	3	0	HH 37	Index	Positive	BA.2	28/03/2022
150	Male	34	3	HH 37	Secondary	Positive	BA.2	28/03/2022
151	Female	42	3	HH 37		Negative		
152	Female	5	1	HH 38	Index	Positive	BA.2	11/04/22
153	Male	7	1	HH 38	Index	Positive	BA.1	18/03/2022
154	Female	10	1	HH 38	Secondary	Positive	BA.1	18/03/22
155	Female	45	3	HH 38	Secondary	Positive	BA.2	14/04/22
156	Male	51	3	HH 38	Secondary	Positive	BA.1	21/03/22
157	Female	2	0	HH 39		Negative		
158	Female	4	0	HH 39	Secondary	Positive	BA.2	11/03/22
159	Male	6	0	HH 39	Secondary	Positive	BA.2	14/03/22
160	Female	10	0	HH 39		Negative		
161	Female	33	2	HH 39	Index	Positive	BA.2	09/03/2022
162	Male	34	2	HH 39	Secondary	Positive	BA.2	09/03/2022
163	Male	7	2	HH 40	Secondary	Positive	BA.2	5/04/22
164	Female	9	2	HH 40	Secondary	Positive	BA.2	2/04/22
165	Female	12	2	HH 40	Index	Positive	BA.2	30/03/22
166	Female	44	3	HH 40	Secondary	Positive	BA.2	2/04/22
167	Male	50	3	HH 40	Secondary	Positive	BA.2	2/04/22
168	Male	11	0	HH 41		Negative		
169	Male	14	2	HH 41	Index	Positive	BA.2	09/05/2022
170	Female	50	2	HH 41		Negative		
171	Male	50	2	HH 41		Negative		
172	Female	2	0	HH 42		Negative		
173	Female	33	3	HH 42	Index	Positive	BA.2	24/05/2022
174	Male	41	3	HH 42		Negative		
175	Female	6	1	HH 43	Index	Positive	BA.1	28/03/22
176	Female	21	3	HH 43	Secondary	Positive	BA.1	28/03/22
177	Female	38	3	HH 43	Secondary	Positive	BA.1	31/03/22
178	Female	11	2	HH 44	Index	Positive		21/03/2022
179	Female	43	3	HH 44		Negative		
180	Male	43	3	HH 44		Negative		

181	Male	9	0	HH 45	Index	Positive		9/05/22
182	Female	15	2	HH 45	Secondary	Positive	BA.2	13/05/22
183	Female	40	3	HH 45	Secondary	Positive	BA.2	13/05/22
184	Male	45	3	HH 45	Secondary	Positive	BA.2	13/05/22
185	Female	12	2	HH 46	Index	Positive	BA.2	16/05/2022
186	Male	12	2	HH 46		Negative		
187	Female	16	2	HH 46		Negative		
188	Female	44	3	HH 46		Negative		
189	Male	50	3	HH 46		Negative		
190	Female	63	3	HH 46		Negative		
191	Male	8	1	HH 47	Secondary	Positive	BA.2	18/03/22
192	Female	12	2	HH 47		Negative		
193	Male	14	2	HH 47		Negative		
194	Male	43	3	HH 47	Index	Positive	BA.2	18/03/2022
195	Female	43	3	HH 47	Secondary	Positive	BA.2	18/03/22
196	Female	1	0	HH 48	Secondary	Positive	BA.2	05/04/2022
197	Male	9	1	HH 48	Secondary	Positive	BA.2	5/04/22
198	Female	13	2	HH 48	Index	Positive	BA.2	5/04/22
199	Female	15	2	HH 48	Secondary	Positive	BA.2	8/04/22
200	Female	34	3	HH 48	Secondary	Positive	BA.2	05/04/2022
201	Male	49	3	HH 48	Secondary	Positive	BA.2	11/04/22
202	Female	1	0	HH 49	Secondary	Positive	BA.2	11/03/2022
203	Female	8	1	HH 49	Index	Positive	BA.2	11/03/2022
204	Female	9	1	HH 49	Secondary	Positive	BA.2	11/03/2022
205	Female	31	2	HH 49	Secondary	Positive	BA.2	11/03/22
206	Male	36	2	HH 49	Secondary	Positive	BA.2	11/03/22
207	Male	0	0	HH 50	Secondary	Positive	BA.1	19/03/22
208	Male	3	0	HH 50	Secondary	Positive	BA.1	16/03/2022
209	Female	8	0	HH 50	Secondary	Positive	BA.1	16/03/22
210	Male	34	0	HH 50	Index	Positive	BA.1	15/03/2022
211	Female	35	0	HH 50	Secondary	Positive	BA.1	19/03/22
212	Female	4	0	HH 51	Secondary	Positive	BA.2	24/03/22
213	Male	6	1	HH 51	Secondary	Positive	BA.2	24/03/22
214	Male	8	1	HH 51	Secondary	Positive	BA.2	24/03/22
215	Female	39	3	HH 51	Index	Positive	BA.2	21/03/22
216	Male	39	3	HH 51	Secondary	Positive	BA.2	21/03/2022
217	Male	1	0	HH 52		Negative		
218	Male	3	0	HH 52		Negative		
219	Female	34	3	HH 52	Secondary	Positive	BA.2	30/05/22
220	Male	37	3	HH 52	Index	Positive	BA.2	26/05/2022
221	Male	1	0	HH 53		Negative		
222	Male	35	2	HH 53	Index	Positive	BA.2	03/05/2022
223	Female	35	3	HH 53		Negative		
224	Female	3	0	HH 54	Secondary	Positive	BA.2	5/04/22
225	Male	4	0	HH 54	Secondary	Positive	BA.2	2/04/22
226	Male	6	2	HH 54	Index	Positive	BA.2	29/03/22
227	Male	37	3	HH 54	Secondary	Positive	BA.2	5/04/22
228	Female	40	3	HH 54	Secondary	Positive	BA.2	2/04/22
229	Female	0	0	HH 55	Index	Positive	BA.2	01/04/2022
230	Female	4	0	HH 55		Negative		
231	Female	6	2	HH 55		Negative		

232	Female	39	3	HH 55		Negative		
233	Female	43	3	HH 55		Negative		
234	Female	17	2	HH 56	Index	Positive	BA.2	10/03/22
235	Female	18	2	HH 56		Negative		
236	Female	48	3	HH 56		Negative		
237	Male	0	0	HH 57		Negative		
238	Female	3	0	HH 57		Negative		
239	Female	5	0	HH 57		Negative		
240	Male	16	0	HH 57		Negative		
241	Female	33	0	HH 57	Index	Positive	BA.5	16/08/22
242	Male	37	0	HH 57		Negative		
243	Male	1	0	HH 58	Secondary	Positive	BA.5	27/06/22
244	Female	33	0	HH 58	Index	Positive	BA.5	23/06/22
245	Female	37	0	HH 58	Secondary	Positive	BA.5	30/06/22
246	Male	0	0	HH 59		Negative		
247	Female	12	0	HH 59	Index	Positive		30/03/2022
248	Male	32	3	HH 59		Negative		
249	Female	40	3	HH 59		Negative		
250	Female	15	2	HH 60	Secondary	Positive	BA.2	3/06/22
251	Female	17	2	HH 60	Secondary	Positive	BA.2	25/05/22
252	Female	48	3	HH 60	Index	Positive	BA.2	20/05/2022
253	Male	51	3	HH 60	Secondary	Positive	BA.2	28/05/22
254	Male	2	0	HH 61	Secondary	Positive	BA.2	1/08/22
255	Male	12	0	HH 61		Negative		
256	Male	31	0	HH 61	Index	Positive	BA.2	01/08/2022
257	Female	32	0	HH 61	Secondary	Positive	BA.2	1/08/22
258	Male	7	0	HH 62		Negative		
259	Male	10	0	HH 62		Negative		
260	Female	37	0	HH 62	Index	Positive	BA.5	13/07/2022
261	Male	39	0	HH 62		Negative		
262	Female	0	0	HH 63		Negative		
263	Male	32	3	HH 63	Index	Positive	BA.2	07/04/2022
264	Female	34	3	HH 63		Negative		
265	Female	0	0	HH 64	Index	Positive	BA.2	30/03/2022
266	Female	37	3	HH 64		Negative		
267	Male	38	3	HH 64		Negative		
268	Male	0	0	HH 65	Secondary	Positive	BA.2	10/04/22
269	Female	3	0	HH 65	Index	Positive	BA.2	4/04/22
270	Male	34	3	HH 65	Secondary	Positive	BA.2	4/04/22
271	Female	34	3	HH 65	Secondary	Positive	BA.2	7/04/22
272	Female	1	0	HH 66	Secondary	Positive	BA.5	16/08/22
273	Female	30	0	HH 66	Index	Positive	BA.5	15/08/22
274	Male	31	0	HH 66	Secondary	Positive	BA.5	16/08/22
275	Female	11	2	HH 67		Negative		
276	Female	15	2	HH 67	Secondary	Positive	BA.2	6/05/22
277	Female	47	3	HH 67	Index	Positive	BA.2	04/05/2022
278	Male	16	3	HH 68	Index	Positive		08/06/2022
279	Male	19	2	HH 68	Secondary	Positive	BA.2	8/06/22
280	Female	48	3	HH 68	Secondary	Positive	BA.2	8/06/22
281	Male	9	0	HH 69	Index	Positive	BA.5	25/07/22

282	Male	39	0	HH 69	Secondary	Positive	BA.5	28/07/22
283	Female	39	0	HH 69	Secondary	Positive	BA.5	31/07/2022
284	Male	11	0	HH 70	Index	Positive	BA.2	4/04/22
285	Male	35	3	HH 70	Secondary	Positive	BA.2	8/04/22
286	Female	42	2	HH 70	Secondary	Positive	BA.2	8/04/22
287	Male	0	0	HH 71	Secondary	Positive	BA.2	14/05/22
288	Female	33	2	HH 71	Index	Positive		09/05/2022
289	Male	35	3	HH 71	Secondary	Positive	BA.2	14/05/22

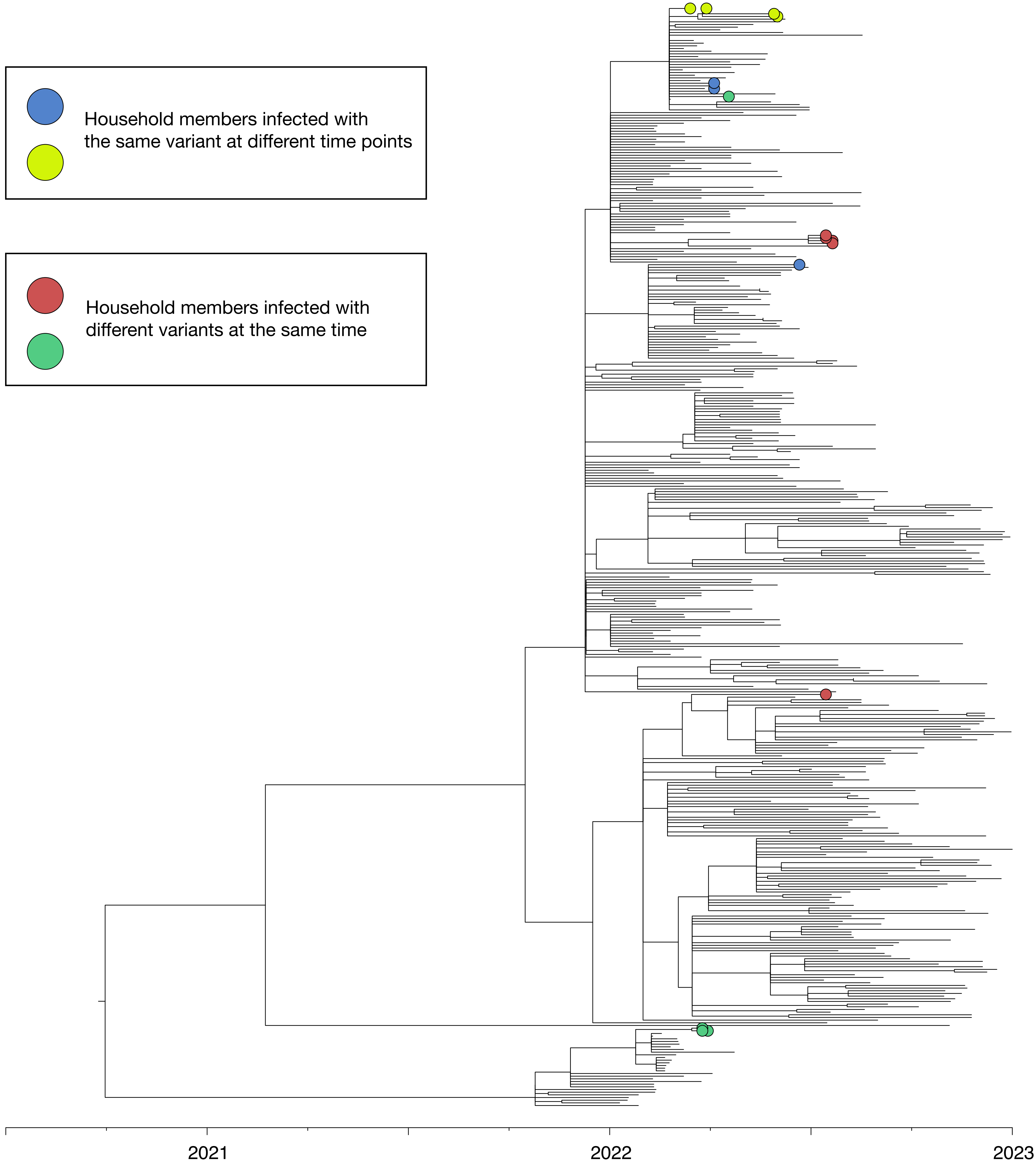

Supplementary Figure 1. Maximum likelihood time-calibrated phylogenetic tree showing the phylogenetic position of genomes from four households: two with members of the same household that were infected with the same Omicron subvariant at different time points during this study (blue and yellow) and two with members of the same holdhouse infected with different subvariants at the same time.
